# Supplementary material for: Industry funding of patient organisations in the UK: a retrospective study of commercial determinants, funding concentration and disease prevalence
Source: BMJ Open. 2023 Jun 27;13(6):e071138. doi: 10.1136/bmjopen-2022-071138 (PMC10410975; doi:10.1136/bmjopen-2022-071138)
Supplement: Supplementary data [file bmjopen-2022-071138supp001.pdf]

# Supplemental Material

## Data collection

### <u>Payments</u>

We retrieved data on 2020 payments from pharmaceutical companies to patient organisations from the following sources:

1) **<u>Companies' websites</u>**. Disclosing payments to patient organisations is a requirement of Clause 29 of the Association of British Pharmaceutical Industry (ABPI) Code of Practice.[1] Specifically, the ABPI requires companies to keep a public record of any payment made to patient organisations on their website for a minimum of three years following the payment.[1] Therefore, companies' website were our primary data source on payments to patient organisations.
2) **<u>Disclosure UK HCOs database</u>**. In light of a recent study unveiling that payments to patient organisations were misreported in the Disclosure UK database of payments to healthcare organisations (HCOs),[2] we also screened the 2020 Disclosure UK HCOs database for payments to patient organisations.

If payments were not disclosed in the company's website nor in the Disclosure UK HCOs database, we assumed that the company did not make any payments to patient organisations in 2020, as commonly assumed in the literature.[3]

One investigator (AG) extracted payment disclosures from the companies' websites. These comprised the name of the patient organisation, the year when the payment was made, the reason for the payment and its value in the currency reported by the disclosing company. The 2020 Disclosure UK HCOs database was also screened, and recipients were matched to standardised patient organisations names. To ensure the data's accuracy, the final database was scanned for duplicates, but no such instances were found. When reported in different currencies, such as United States Dollars (USD), Swiss Franc (CHF), Swedish Krona (SEK), Norwegian Krone (NKK) and Danish Krone (DKK), the value of the payment was converted to Great British Pounds (GBP), using the ONS historical yearly conversion rates. [4,5] Two in-kind payments with a monetary value of zero were excluded from the analysis. Further details on variables' cleaning and coding can be found in the Supplemental Material.

### <u>Therapeutic areas</u>

Patient organisations' websites were also screened to understand the condition(s) they focused on. For example, in the case of *Blood Cancer UK*, their mission is to "*beat blood cancer"*, therefore, the condition supported was coded as blood cancer.

After being identified as described above, conditions were further classified into rare and non-rare.



Gentilini A, Parvanova I. *BMJ Open* 2023; 13:e071138. doi: 10.1136/bmjopen-2022-071138

Conditions were considered rare if they appeared in the Orphanet database of rare diseases regardless of their classification level (group of disorders, disorders or subtypes of disorders).[6] For example, multiple myeloma appears in the Orphanet database of rare diseases, therefore a patient organisation focusing this condition would be categorised as rare-focused. When condition sub-types appeared in the Orphanet database, the patient organisation's website was screened to check whether its focus was on rare conditions. For example, *Metabolic Support UK's* motto is "*Your rare condition. Our common fight"* and was therefore assumed to be rare disease-focused. Conversely, should a patient organisation focus on a broader condition such as blood cancer with no sole focus on rare conditions, the organisation would be conservatively considered non-rare. While this approach was preferred as it did not require further assumptions, it entails that only more specialised patient organisation are considered as rare. Such approach might have led to the number and overall value of payments from pharmaceutical companies to rare diseases-focused patient organisations being underestimated, as these organisaitons are expected to get less payments than more generalist ones (e.g. multiple myeloma vs blood cancer).

A third category (*unclear)* was created for non-disease-specific patient organisations, such as coalition of charities or organisations focused on palliative care for terminally ill patients. This category was excluded from the main analyses, but sub-group analyses are reported at the end of the Supplemental Material.

**Companies' interest**

We developed a methodology to assess the extent to which a pharmaceutical company holds an interest in the disease supported by a patient organisation. For the purpose of this analysis, we adapted the definition of interest provided by NICE.[7] An interest is when there is, or could be perceived to be, an opportunity for a pharmaceutical company to benefit in the disease area where the patient organisation operates. This could include situations where the pharmaceutical company has a drug developed or in development for a condition supported by the patient organisation, or where a drug in the company's portfolio or pipeline is restricted to a specific population affected by the disease supported by the patient organisation.

As first step, the conditions supported by patient organisations were translated into ICD-11 codes using the online ICD-11 database.[8]

ICD-11 codes are mutually exclusive, exhaustive and are arranged as a single hierarchical tree. This means that specific diseases are nested within broader classifications. An example for multiple myeloma is shown in Table 1 below.

**Table 1. Example of ICD-11 classification, Multiple myeloma**

| Hierarchy level | Condition | ICD-11 code |
|---|---|---|
| Level 1 | Neoplasms | 2 |
| Level 2 | Neoplasms of haematopoietic or lymphoid tissues | 2A |
| Level 3 | Mature B-cell neoplasms | 2A8 |
| Level 4 | Plasma cell neoplasms | 2A83 |
| Level 5 | Plasma cell myeloma | 2A83.1 |



Gentilini A, Parvanova I. *BMJ Open* 2023; 13:e071138. doi: 10.1136/bmjopen-2022-071138

In this example, multiple myeloma is nested within *Plasma cell myeloma*, who is in its turn nested within *Plasma cell neoplasms* and so on up to *Neoplasms*.

Subsequently, companies' annual reports, website and the ClinicalTrials.gov database were searched to assess whether the each company had an interest in the condition supported by the patient organisation receiving the payment. The diagram in the main document (Figure 1) schematically illustrates the approach taken to understand whether the company definitely, probably or did not have an interest in the condition. Figure 1 below illustrates the source of companies' interests.

For example, if *Company X* reports in its annual report having a drug in development for multiple myeloma and transferred a sum of money to *Blood Cancer UK*, this would be coded as *probably yes,* as the company has a product in its pipeline or portfolio associated with a condition supported by the patient organisation. In this case, the ICD-11 level would be 2, *Neoplasms of haematopoietic or lymphoid tissue*, under which multiple myeloma is nested. Conversely, should *Company X* have made a payment to *Myeloma UK,* this would have been coded as *definitely yes,* as there is perfect alignment between the condition supported by the patient organisation and by *Company X's* drug.

Situations where a company's interest in a certain condition could not be identified indicate an impossibility of identifying such link, rather than the lack thereof.

**Figure 1. Source of companies interests**

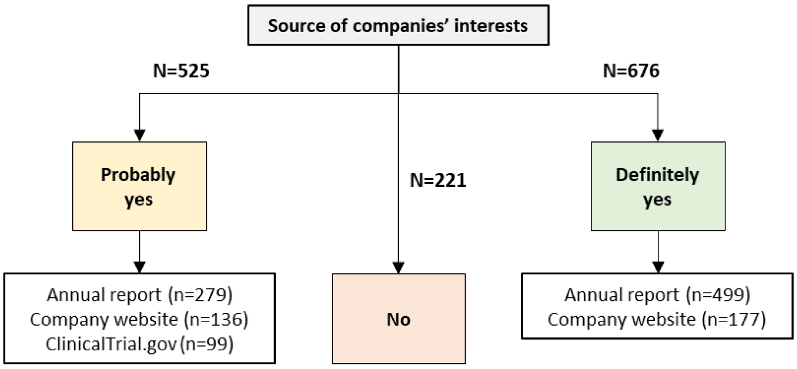

## Variables cleaning and coding

**Table 2. Description of key variables in payment database**

| Variables name | Description | Details |
|---|---|---|
| **Company** | Standardised company name | Company name as reported on company website and/or on HCOs database.<br>Two mergers involving companies included in our analysis—BMS and Celgene, and Takeda and Shire—were completed prior to 2020. Although these companies had merged, we treated them as separate entities because their disclosures were reported separately even after the acquisition. |
| **ABPI member** | ABPI membership of company; *source: ABPI full members list* | 0 = not ABPI member, 1 = ABPI member |
| **Company_condition** | Concatenation of company name and disease area targeted by the patient organisation | Concatenation used for coding and analysis purposes |
| **Company interest** | Whether the company hold an interest* in the condition targeted by the patient organsiation | - **Definitely yes**: the company's annual report or website list a product for the condition targeted by the patient organisation in its portfolio/pipeline (ICD-11 level 4 or above)<br>- **Probably yes**: the company's annual report or website list a product for the condition targeted by the patient organisation in its portfolio/pipeline OR a clinical trial for which the company is sponsor is listed for the disease targeted by the patient organisation OR a drug in the company's pipeline/portfolio is restricted to a specific population affected by the disease targeted by the patient organisation (ICD-11 level 3 or below)<br>- **No** : None of the above |
| **Source** | Source of company interest variable | Annual report, company website, ClinicalTrials.gov, none |
| **Name of PO** | Name of patient organization as reported by companies in disclosure report | - |
| **Standardised PO name** | Standardised name of patient organization to avoid duplicates and inconsistencies | For coding purposes, names of patient organisations were standardised. The following steps were taken:<br>1. Patient organisations' names for typos, abbreviations, spelling mistakes and duplicated within the companies' disclosures (e.g. Crohn's & Colitis UK and CCUK would both be standardized to Crohn's and Colitis UK);<br>2. If the patient organisation changed name over time, the latest name on record was used; |

| | | |
|---|---|---|
| | | 3. If the two patient organisations merged over the study period, the name of the merged entity was used (e.g. the British Lung Foundation and Asthma UK merged into Asthma + Lung UK);<br>4. Separate entries were made for patient organisations under the same umbrella but focusing on different geographical entities (e.g. Alzheimer UK vs Alzheimer Scotland) |
| **Reason for exclusion** | Reason why the organisation was excluded from the analysis | - Not UK organisation (not aligned with geographical scope e.g. Irish, US-based);<br>- For profit company (not aligned with definition of patient organization used in the study);<br>- Missing information (organisations for whose nature is unclear i.e. patient organisation website could not be identified) |
| **ICD-11** | Classification of disease targeted by the patient organisation according to the WHO ICD-11; *source: ICD WHO website* | General classification (ICD-11 chapters)<br>*See Excel file, Inputs tab* |
| **Condition** | Condition targeted by patient organisation as reported on website | e.g. Blood Cancer UK would fall under ICD-11 code 02 Neoplasms, with *blood cancer* being the condition |
| **Charity number (if any)** | Charity number as reported in the organization website or as reported in the England and Wales Charity Commission website | When both England/Wales and Scotland or Northern Ireland charity numbers were provided, the former was chosen. Scotland and Northern Ireland charity numbers were reported only when those for England/Wales were missing |
| **Company number (if charity number missing)** | Company number as reported in the organization website or as reported in the Government Company Information Service wesbite if the patient organization cannot be found in the charity commission database (e.g. limited by guarantee company) | When both England/Wales and Scotland or Northern Ireland charity numbers were provided, the former was chosen. Scotland and Northern Ireland charity numbers were reported only when those for England/Wales were missing |
| **Link** | Link of patient organisation website | - |
| **Rare disease** | Whether the condition or one of the conditions targeted by the patient organisation is considered as rare | A condition was considered as rare if it under at least one of the following criteria:<br>1. The condition is listed in Orphanet list of rare diseases regardless of its ICD-11 level classification;<br>2. In their website, the patient organisation explicitly describe the disease they target as rare (e.g. *Metabolic Support UK's* motto is "*Your rare condition. Our common fight"* and was therefore assumed to be rare disease-focused) |



Gentilini A, Parvanova I. *BMJ Open* 2023; 13:e071138. doi: 10.1136/bmjopen-2022-071138

| | | |
|---|---|---|
| **Details of payment** | Details of payment as reported by companies in disclosure report | - |
| **Country** | Country of payment | The country considered for the entire database is the UK |
| **Year** | Year of payment | 2020 |
| **Currency** | Currency of payment | Currency the payment is reported in the disclosure reports (i.e. EUR, GBP, USD, CHF, SEK, NKK) |
| **Currency_year** | Concatenation of currency and year of payment for conversion purposes | - |
| **Value of payment** | Value of payment in original currency as reported by companies in disclosure report | In-kind payments were removed from the analysis as no monetary value could be associated to such payments |
| **Value in 2020 pounds** | GBP converted value of payment | See details in *Inputs* sheet |

*An interest is when there is, or could be perceived to be, an opportunity for a pharmaceutical company to benefit in the disease area where the patient organisation operates.



Gentilini A, Parvanova I. *BMJ Open* 2023; 13:e071138. doi: 10.1136/bmjopen-2022-071138

## Disclosure details

**Table 3. Reporting of payments to patient organizations by pharmaceutical companies: comparison of company websites and Disclosure UK HCOs database**

| Company | Company website only | HCOs database only | Both |
|---|---|---|---|
| Abbvie | X | | |
| Alexion | X | | |
| Almirall | X | | |
| Alnylam | | | X |
| Amgen | | | X |
| Amryt | X | | |
| Astellas | | | X |
| AstraZeneca | | | X |
| BMS | | | X |
| Bayer | | | X |
| Bial | | X | |
| BioMarin | | | X |
| Biogen | X | | |
| BlueBird | X | | |
| Boehringer Ingelheim | | | X |
| Britannia | | | X |
| CSL Behring | X | | |
| Camurus | | | X |
| Celgene | | | X |
| Chiesi | | | X |
| Chugai | X | | |
| Clinuvel | X | | |
| Daiichi Sankyo | | | X |
| Diurnal | X | | |
| Eisai | | | X |
| Eli Lilly | | | X |
| Ever | | | X |
| Ferring | | X | |
| Flynn | | X | |
| GSK | | | X |
| GW | | | X |
| Gilead | | X | |
| Grünenthal | | | X |
| Guerbet | | X | |
| HRA | | X | |
| Immedica | X | | |
| Indivior | X | | |
| Intercept | X | | |
| Ipsen | | X | |
| Janssen | | | X |



Gentilini A, Parvanova I. *BMJ Open* 2023; 13:e071138. doi: 10.1136/bmjopen-2022-071138

| | | | |
|---|---|---|---|
| **LEO** | X | | |
| **Lundbeck** | | | X |
| **Lupin** | X | | |
| **MSD** | | | X |
| **Merck** | | | X |
| **Merz** | | | X |
| **Napp** | | | X |
| **Norgine** | | X | |
| **Novartis** | | | X |
| **Novo Nordisk** | | | X |
| **Octapharma** | | X | |
| **PTC** | X | | |
| **Pfizer** | | | X |
| **Pharmasure** | | X | |
| **Pierre Fabre** | | | X |
| **Recordati** | X | | |
| **Roche** | | | X |
| **Rosemont** | | | X |
| **Sandoz** | | X | |
| **Sanofi** | | | X |
| **Santen** | X | | |
| **Seqirus** | X | | |
| **Servier** | X | | |
| **Shionogi** | | X | |
| **Shire** | | | X |
| **Sobi** | X | | |
| **Takeda** | | | X |
| **Teva** | | X | |
| **Tillotts** | X | | |
| **UCB** | | | X |
| **Valneva** | X | | |
| **Veriton** | | X | |
| **Vifor** | | | X |
| **Zogenix** | X | | |
| **Total (n;%)** | 24; 32% | 14; 19% | 36; 49% |

**Table 4. Reporting of payments to patient organizations by pharmaceutical companies: payments disclosed on company websites and Disclosure UK HCOs database**

| Company | Payments reported on company website (£) | Payments reported on HCOs database (£) | Total |
|---|---|---|---|
| **Abbvie** | £ 371,503 | £ - | £ 371,503 |
| **Alexion** | £ 168,925 | £ - | £ 168,925 |
| **Almirall** | £ 9,775 | £ - | £ 9,775 |
| **Alnylam** | £ 51,559 | £ 14,050 | £ 65,609 |
| **Amgen** | £ 347,757 | £ 68,845 | £ 416,602 |



Gentilini A, Parvanova I. *BMJ Open* 2023; 13:e071138. doi: 10.1136/bmjopen-2022-071138

| | | | |
|---|---|---|---|
| **Amryt** | £ 45,413 | £ - | £ 45,413 |
| **Astellas** | £ 94,583 | £ 13,071 | £ 107,654 |
| **AstraZeneca** | £ 326,201 | £ 88,175 | £ 414,376 |
| **BMS** | £ 517,082 | £ 17,750 | £ 534,832 |
| **Bayer** | £ 171,758 | £ 9,098 | £ 180,856 |
| **Bial** | £ - | £ 5,500 | £ 5,500 |
| **BioMarin** | £ 411,912 | £ 310,455 | £ 722,367 |
| **Biogen** | £ 663,142 | £ - | £ 663,142 |
| **BlueBird** | £ 94,000 | £ - | £ 94,000 |
| **Boehringer Ingelheim** | £ 79,762 | £ 30,000 | £ 109,762 |
| **Britannia** | £ 35,000 | £ 2,030 | £ 37,030 |
| **CSL Behring** | £ 152,192 | £ - | £ 152,192 |
| **Camurus** | £ 13,168 | £ 6,500 | £ 19,668 |
| **Celgene** | £ 310,329 | £ 640 | £ 310,969 |
| **Chiesi** | £ 602,259 | £ 60,000 | £ 662,259 |
| **Chugai** | £ 62,092 | £ - | £ 62,092 |
| **Clinuvel** | £ 1,000 | £ - | £ 1,000 |
| **Daiichi Sankyo** | £ 57,879 | £ 329,385 | £ 387,264 |
| **Diurnal** | £ 6,000 | £ - | £ 6,000 |
| **Eisai** | £ 476,271 | £ 183,207 | £ 659,478 |
| **Eli Lilly** | £ 874,288 | £ 62,690 | £ 936,978 |
| **Ever** | £ 18,934 | £ 18,934 | £ 37,867 |
| **Ferring** | £ - | £ 38,000 | £ 38,000 |
| **Flynn** | £ - | £ 8,002 | £ 8,002 |
| **GSK** | £ 325,410 | £ 159,064 | £ 484,474 |
| **GW** | £ 98,788 | £ 303 | £ 99,091 |
| **Gilead** | £ - | £ 417,448 | £ 417,448 |
| **Grünenthal** | £ 4,200 | £ 1,000 | £ 5,200 |
| **Guerbet** | £ - | £ 17,000 | £ 17,000 |
| **HRA** | £ - | £ 10,000 | £ 10,000 |
| **Immedica** | £ 19,954 | £ - | £ 19,954 |
| **Indivior** | £ 1,200 | £ - | £ 1,200 |
| **Intercept** | £ 71,712 | £ - | £ 71,712 |
| **Ipsen** | £ - | £ 50,050 | £ 50,050 |
| **Janssen** | £ 1,170,768 | £ 10,000 | £ 1,180,768 |
| **LEO** | £ 78,633 | £ - | £ 78,633 |
| **Lundbeck** | £ 89,400 | £ 40,309 | £ 129,709 |
| **Lupin** | £ 24,000 | £ - | £ 24,000 |
| **MSD** | £ 537,632 | £ 225,287 | £ 762,919 |
| **Merck** | £ 763,885 | £ 1,000 | £ 764,885 |
| **Merz** | £ 31,114 | £ 5,789 | £ 36,903 |
| **Napp** | £ 8,000 | £ 18,020 | £ 26,020 |
| **Norgine** | £ - | £ 1,240 | £ 1,240 |
| **Novartis** | £ 1,442,037 | £ 46,812 | £ 1,488,849 |
| **Novo Nordisk** | £ 452,113 | £ 1,411,598 | £ 1,863,711 |

| | | | |
|---|---|---|---|
| **Octapharma** | £ - | £ 2,995 | £ 2,995 |
| **PTC** | £ 151,433 | £ - | £ 151,433 |
| **Pfizer** | £ 1,360,510 | £ 509,793 | £ 1,870,303 |
| **Pharmasure** | £ - | £ 6,000 | £ 6,000 |
| **Pierre Fabre** | £ 50,010 | £ 34,096 | £ 84,106 |
| **Recordati** | £ 14,500 | £ - | £ 14,500 |
| **Roche** | £ 1,169,578 | £ 101,395 | £ 1,270,973 |
| **Rosemont** | £ 200 | £ 200 | £ 400 |
| **Sandoz** | £ - | £ 20,000 | £ 20,000 |
| **Sanofi** | £ 1,262,802 | £ 3,825 | £ 1,266,627 |
| **Santen** | £ 38,170 | £ - | £ 38,170 |
| **Seqirus** | £ 105,000 | £ - | £ 105,000 |
| **Servier** | £ 17,163 | £ - | £ 17,163 |
| **Shionogi** | £ - | £ 17,000 | £ 17,000 |
| **Shire** | £ 555,244 | £ 53,980 | £ 609,224 |
| **Sobi** | £ 132,988 | £ - | £ 132,988 |
| **Takeda** | £ 420,549 | £ 17,270 | £ 437,819 |
| **Teva** | £ - | £ 51,410 | £ 51,410 |
| **Tillotts** | £ 830 | £ - | £ 830 |
| **UCB** | £ 1,493,896 | £ 35,378 | £ 1,529,274 |
| **Valneva** | £ 59,512 | £ - | £ 59,512 |
| **Veriton** | £ - | £ 15,000 | £ 15,000 |
| **Vifor** | £ 58,083 | £ 12,000 | £ 70,083 |
| **Zogenix** | £ 43,625 | £ - | £ 43,625 |
| **Total (£;%)** | £18,015,722; 80% | £4,561,593; 20% | £22,577,314; 100% |

**Table 5. Companies' commercial interests by ICD-11 codes according to 2020 payments**

| | ICD-11 | | | | | | | | | | | | | | | | | | |
|---|---|---|---|---|---|---|---|---|---|---|---|---|---|---|---|---|---|---|---|
| **Company** | **01** | **02** | **03** | **04** | **05** | **06** | **08** | **09** | **11** | **12** | **13** | **14** | **15** | **16** | **18** | **19** | **20** | **22** | **Other** |
| **Abbvie** | 1 | 1 | 0 | 0 | 0 | 0 | 1 | 0 | 0 | 0 | 1 | 1 | 1 | 0 | 0 | 0 | 0 | 0 | 0 |
| **Alexion** | 0 | 0 | 1 | 0 | 1 | 0 | 0 | 0 | 0 | 0 | 0 | 0 | 0 | 0 | 0 | 0 | 0 | 0 | 0 |
| **Almirall** | 0 | 0 | 0 | 0 | 0 | 0 | 0 | 0 | 0 | 0 | 0 | 1 | 0 | 0 | 0 | 0 | 0 | 0 | 0 |
| **Alnylam** | 0 | 0 | 0 | 0 | 1 | 0 | 0 | 0 | 0 | 0 | 0 | 0 | 0 | 0 | 0 | 0 | 0 | 0 | 0 |
| **Amgen** | 0 | 1 | 1 | 0 | 0 | 0 | 0 | 0 | 0 | 0 | 1 | 1 | 1 | 0 | 0 | 0 | 0 | 0 | 0 |
| **Amryt** | 0 | 0 | 0 | 0 | 1 | 0 | 0 | 0 | 0 | 0 | 0 | 0 | 0 | 0 | 0 | 0 | 0 | 0 | 0 |
| **Astellas** | 0 | 1 | 0 | 0 | 0 | 0 | 0 | 0 | 0 | 0 | 0 | 0 | 0 | 0 | 0 | 0 | 0 | 0 | 0 |
| **AstraZeneca** | 0 | 1 | 0 | 0 | 1 | 0 | 0 | 0 | 1 | 0 | 0 | 0 | 0 | 1 | 0 | 0 | 0 | 0 | 0 |
| **BMS** | 0 | 1 | 0 | 0 | 0 | 0 | 1 | 0 | 1 | 0 | 0 | 0 | 1 | 0 | 0 | 0 | 0 | 0 | 0 |
| **Bayer** | 0 | 1 | 0 | 0 | 0 | 0 | 0 | 1 | 1 | 0 | 0 | 0 | 0 | 0 | 0 | 0 | 0 | 0 | 0 |
| **Bial** | 0 | 0 | 0 | 0 | 0 | 0 | 1 | 0 | 0 | 0 | 0 | 0 | 0 | 0 | 0 | 0 | 0 | 0 | 0 |
| **BioMarin** | 0 | 0 | 1 | 0 | 1 | 0 | 0 | 0 | 0 | 0 | 0 | 0 | 0 | 0 | 0 | 0 | 0 | 0 | 0 |
| **Biogen** | 0 | 0 | 0 | 0 | 0 | 0 | 1 | 1 | 0 | 0 | 0 | 0 | 1 | 0 | 0 | 0 | 0 | 0 | 0 |
| **BlueBird** | 0 | 0 | 1 | 0 | 0 | 0 | 1 | 0 | 0 | 0 | 0 | 0 | 0 | 0 | 0 | 0 | 0 | 0 | 0 |
| **Boehringer Ingelheim** | 0 | 0 | 0 | 1 | 0 | 0 | 0 | 0 | 0 | 1 | 0 | 0 | 0 | 0 | 0 | 0 | 0 | 0 | 0 |
| **Britannia** | 0 | 0 | 0 | 0 | 0 | 0 | 1 | 0 | 0 | 0 | 0 | 0 | 0 | 0 | 0 | 0 | 0 | 0 | 0 |
| **CSL Behring** | 1 | 0 | 1 | 1 | 0 | 0 | 0 | 0 | 0 | 0 | 0 | 0 | 0 | 0 | 0 | 0 | 0 | 0 | 0 |
| **Camurus** | 0 | 0 | 0 | 0 | 0 | 0 | 0 | 0 | 0 | 0 | 0 | 0 | 0 | 0 | 0 | 0 | 0 | 0 | 0 |
| **Celgene** | 0 | 1 | 0 | 0 | 0 | 0 | 0 | 0 | 0 | 0 | 0 | 0 | 0 | 0 | 0 | 0 | 0 | 0 | 0 |
| **Chiesi** | 0 | 0 | 1 | 0 | 1 | 0 | 0 | 0 | 0 | 1 | 0 | 0 | 0 | 0 | 0 | 0 | 0 | 0 | 0 |
| **Chugai** | 0 | 0 | 1 | 0 | 0 | 0 | 0 | 0 | 0 | 0 | 0 | 0 | 1 | 0 | 0 | 0 | 0 | 0 | 0 |
| **Clinuvel** | 0 | 0 | 0 | 0 | 1 | 0 | 0 | 0 | 0 | 0 | 0 | 0 | 0 | 0 | 0 | 0 | 0 | 0 | 0 |
| **Daiichi Sankyo** | 0 | 1 | 0 | 0 | 0 | 0 | 0 | 0 | 1 | 0 | 0 | 0 | 0 | 0 | 0 | 0 | 0 | 0 | 0 |
| **Diurnal** | 0 | 0 | 0 | 0 | 1 | 0 | 0 | 0 | 0 | 0 | 0 | 0 | 0 | 0 | 0 | 0 | 0 | 0 | 0 |

| | | | | | | | | | | | | | | | | | | | |
|---|---|---|---|---|---|---|---|---|---|---|---|---|---|---|---|---|---|---|---|
| **Eisai** | 0 | 1 | 0 | 0 | 0 | 1 | 1 | 0 | 0 | 0 | 0 | 0 | 0 | 0 | 0 | 0 | 0 | 0 | 0 |
| **Eli Lilly** | 0 | 1 | 0 | 0 | 1 | 0 | 1 | 0 | 0 | 0 | 0 | 1 | 1 | 0 | 0 | 0 | 0 | 0 | 0 |
| **Ever** | 0 | 0 | 0 | 0 | 0 | 0 | 1 | 0 | 0 | 0 | 0 | 0 | 0 | 0 | 0 | 0 | 0 | 0 | 0 |
| **Ferring** | 0 | 1 | 0 | 0 | 0 | 0 | 0 | 0 | 0 | 0 | 0 | 0 | 0 | 1 | 0 | 0 | 0 | 0 | 0 |
| **Flynn** | 0 | 0 | 0 | 0 | 0 | 1 | 0 | 0 | 0 | 0 | 0 | 0 | 0 | 0 | 0 | 0 | 0 | 0 | 0 |
| **GSK** | 1 | 1 | 0 | 1 | 0 | 0 | 0 | 0 | 1 | 0 | 0 | 0 | 0 | 0 | 0 | 0 | 0 | 0 | 0 |
| **GW** | 0 | 0 | 0 | 0 | 0 | 0 | 0 | 0 | 0 | 0 | 0 | 0 | 0 | 0 | 0 | 0 | 0 | 0 | 0 |
| **Gilead** | 1 | 1 | 0 | 0 | 0 | 0 | 0 | 0 | 0 | 0 | 1 | 0 | 0 | 0 | 0 | 0 | 0 | 0 | 0 |
| **Grünenthal** | 0 | 0 | 0 | 0 | 0 | 0 | 0 | 0 | 0 | 0 | 0 | 0 | 0 | 0 | 0 | 0 | 0 | 0 | 0 |
| **Guerbet** | 0 | 0 | 0 | 0 | 0 | 0 | 0 | 0 | 0 | 0 | 0 | 0 | 0 | 1 | 0 | 0 | 0 | 0 | 0 |
| **HRA** | 0 | 0 | 0 | 0 | 0 | 0 | 0 | 0 | 0 | 0 | 0 | 0 | 0 | 0 | 0 | 0 | 0 | 0 | 0 |
| **Immedica** | 0 | 0 | 0 | 0 | 0 | 0 | 0 | 0 | 0 | 0 | 0 | 0 | 0 | 0 | 0 | 0 | 0 | 0 | 0 |
| **Indivior** | 0 | 0 | 0 | 0 | 0 | 0 | 0 | 0 | 0 | 0 | 0 | 0 | 0 | 0 | 0 | 0 | 0 | 0 | 0 |
| **Intercept** | 0 | 0 | 0 | 0 | 0 | 0 | 0 | 0 | 0 | 0 | 1 | 0 | 0 | 0 | 0 | 0 | 0 | 0 | 0 |
| **Ipsen** | 0 | 1 | 0 | 0 | 0 | 0 | 1 | 0 | 0 | 0 | 0 | 0 | 0 | 0 | 0 | 0 | 0 | 0 | 0 |
| **Janssen** | 1 | 1 | 0 | 0 | 0 | 0 | 0 | 0 | 1 | 0 | 1 | 1 | 0 | 0 | 0 | 0 | 0 | 0 | 0 |
| **LEO** | 0 | 0 | 0 | 0 | 0 | 0 | 0 | 0 | 1 | 0 | 0 | 1 | 0 | 0 | 0 | 0 | 0 | 0 | 0 |
| **Lundbeck** | 0 | 0 | 0 | 0 | 0 | 1 | 1 | 0 | 0 | 0 | 0 | 0 | 0 | 0 | 0 | 0 | 0 | 0 | 0 |
| **Lupin** | 0 | 0 | 0 | 0 | 0 | 0 | 1 | 0 | 0 | 0 | 0 | 0 | 0 | 0 | 0 | 0 | 0 | 0 | 0 |
| **MSD** | 1 | 1 | 0 | 0 | 0 | 0 | 0 | 0 | 0 | 0 | 0 | 0 | 0 | 0 | 0 | 0 | 0 | 0 | 0 |
| **Merck** | 0 | 1 | 0 | 0 | 0 | 0 | 1 | 0 | 0 | 0 | 0 | 0 | 0 | 1 | 0 | 0 | 0 | 0 | 0 |
| **Merz** | 0 | 0 | 0 | 0 | 0 | 0 | 0 | 0 | 0 | 0 | 0 | 0 | 0 | 0 | 0 | 0 | 0 | 0 | 0 |
| **Napp** | 0 | 0 | 0 | 0 | 1 | 0 | 0 | 0 | 0 | 0 | 0 | 0 | 0 | 0 | 0 | 0 | 0 | 0 | 0 |
| **Norgine** | 0 | 0 | 0 | 0 | 0 | 0 | 0 | 0 | 0 | 0 | 0 | 0 | 0 | 0 | 0 | 0 | 0 | 0 | 0 |
| **Novartis** | 0 | 1 | 1 | 0 | 0 | 0 | 1 | 1 | 1 | 0 | 0 | 1 | 1 | 0 | 0 | 0 | 0 | 0 | 0 |
| **Novo Nordisk** | 0 | 0 | 1 | 0 | 1 | 0 | 0 | 0 | 0 | 0 | 0 | 0 | 0 | 0 | 0 | 0 | 1 | 0 | 0 |
| **Octapharma** | 0 | 0 | 0 | 0 | 0 | 0 | 0 | 0 | 0 | 0 | 0 | 0 | 0 | 0 | 0 | 0 | 0 | 0 | 0 |
| **PTC** | 0 | 0 | 0 | 0 | 0 | 0 | 1 | 0 | 0 | 0 | 0 | 0 | 0 | 0 | 0 | 0 | 0 | 0 | 0 |
| **Pfizer** | 1 | 1 | 1 | 0 | 1 | 0 | 1 | 0 | 1 | 0 | 1 | 0 | 1 | 0 | 0 | 0 | 1 | 0 | 0 |
| **Pharmasure** | 0 | 0 | 0 | 0 | 0 | 0 | 0 | 0 | 0 | 0 | 0 | 0 | 0 | 1 | 0 | 0 | 0 | 0 | 0 |

| | | | | | | | | | | | | | | | | | | | |
|---|---|---|---|---|---|---|---|---|---|---|---|---|---|---|---|---|---|---|---|
| **Pierre Fabre** | 0 | 1 | 0 | 0 | 0 | 0 | 0 | 0 | 0 | 0 | 0 | 1 | 0 | 0 | 0 | 0 | 0 | 0 | 0 |
| **Recordati** | 0 | 0 | 0 | 0 | 1 | 0 | 0 | 0 | 0 | 0 | 0 | 0 | 0 | 0 | 0 | 0 | 0 | 0 | 0 |
| **Roche** | 0 | 1 | 0 | 0 | 0 | 0 | 1 | 0 | 0 | 1 | 1 | 0 | 0 | 0 | 1 | 0 | 0 | 0 | 0 |
| **Rosemont** | 0 | 0 | 0 | 0 | 0 | 0 | 0 | 0 | 0 | 0 | 0 | 0 | 0 | 0 | 0 | 0 | 0 | 0 | 0 |
| **Sandoz** | 0 | 0 | 0 | 0 | 0 | 0 | 0 | 0 | 0 | 0 | 0 | 0 | 0 | 0 | 0 | 0 | 0 | 0 | 0 |
| **Sanofi** | 1 | 1 | 1 | 1 | 1 | 0 | 1 | 0 | 1 | 0 | 0 | 1 | 1 | 1 | 0 | 0 | 0 | 0 | 0 |
| **Santen** | 0 | 0 | 0 | 1 | 0 | 0 | 0 | 1 | 0 | 0 | 0 | 0 | 0 | 0 | 0 | 0 | 0 | 0 | 0 |
| **Seqirus** | 0 | 0 | 0 | 0 | 0 | 0 | 0 | 0 | 0 | 0 | 0 | 0 | 0 | 0 | 0 | 0 | 0 | 0 | 0 |
| **Servier** | 0 | 1 | 0 | 0 | 0 | 0 | 0 | 0 | 0 | 0 | 0 | 0 | 0 | 0 | 0 | 0 | 0 | 0 | 0 |
| **Shionogi** | 0 | 0 | 0 | 0 | 0 | 0 | 0 | 0 | 0 | 0 | 0 | 0 | 0 | 0 | 0 | 0 | 0 | 0 | 0 |
| **Shire** | 0 | 0 | 1 | 1 | 1 | 1 | 0 | 0 | 0 | 0 | 0 | 0 | 0 | 0 | 0 | 0 | 0 | 0 | 0 |
| **Sobi** | 0 | 1 | 1 | 0 | 1 | 0 | 0 | 0 | 0 | 0 | 0 | 0 | 0 | 0 | 0 | 0 | 0 | 0 | 0 |
| **Takeda** | 0 | 1 | 0 | 0 | 1 | 0 | 0 | 0 | 0 | 0 | 1 | 0 | 0 | 0 | 0 | 0 | 0 | 0 | 0 |
| **Teva** | 0 | 0 | 0 | 0 | 0 | 0 | 0 | 0 | 0 | 0 | 0 | 0 | 0 | 0 | 0 | 0 | 0 | 0 | 0 |
| **Tillotts** | 0 | 0 | 0 | 0 | 0 | 0 | 0 | 0 | 0 | 0 | 1 | 0 | 0 | 0 | 0 | 0 | 0 | 0 | 0 |
| **UCB** | 0 | 0 | 1 | 0 | 0 | 0 | 1 | 0 | 0 | 0 | 0 | 1 | 1 | 0 | 0 | 0 | 0 | 0 | 0 |
| **Valneva** | 1 | 0 | 0 | 0 | 0 | 0 | 0 | 0 | 0 | 0 | 0 | 0 | 0 | 0 | 0 | 0 | 0 | 0 | 0 |
| **Veriton** | 0 | 0 | 0 | 0 | 0 | 0 | 0 | 0 | 0 | 0 | 0 | 0 | 0 | 0 | 0 | 0 | 0 | 0 | 0 |
| **Vifor** | 0 | 0 | 0 | 0 | 0 | 0 | 0 | 0 | 0 | 0 | 0 | 0 | 0 | 0 | 0 | 0 | 0 | 0 | 0 |
| **Zogenix** | 0 | 0 | 0 | 0 | 0 | 0 | 1 | 0 | 0 | 0 | 0 | 0 | 0 | 0 | 0 | 0 | 0 | 0 | 0 |

Notes: This table reflects whether companies had a definite or probable interest in the ICD-11 code based on their pipeline or portfolio (1 = yes, 0 = no). Please note that companies' interests were opportunistically screened only in disease areas where they made a payment to a specific patient organisation, and therefore this table should not be considered exhaustive. The table refers payments made in 2020 only.

Legend: 01 Certain infectious or parasitic diseases; 02 Neoplasms; 03 Diseases of the blood or blood-forming organs; 04 Diseases of the immune system; 05 Endocrine, nutritional or metabolic diseases; 06 Mental, behavioural or neurodevelopmental disorders; 08 Diseases of the nervous system; 09 Diseases of the visual system; 11 Diseases of the circulatory system; 12 Diseases of the respiratory system; 13 Diseases of the digestive system; 14 Diseases of the skin; 15 Diseases of the musculoskeletal system or connective tissue; 16 Diseases of the genitourinary system; 18 Pregnancy, childbirth or the puerperium; 19 Certain conditions originating in the perinatal period; 20 Developmental anomalies; 22 Injury, poisoning or certain other consequences of external causes; Other. Other indicates disease areas where patient organisations operate that could not be classified as any ICD-11 codes.

**Table 6. List of patient organisations receiving payments in 2020**

| Standardised name | Charity number | Link |
|---|---|---|
| Acacia Mews Care Home | 1174346 | https://www.nhs.uk/services/Careproviders/Overview/DefaultView.aspx?id=47011 |
| Action Bladder Cancer UK | 1164374 | https://actionbladdercanceruk.org/ |
| Action for Pulmonary Fibrosis | 1152399 | https://www.actionpf.org/ |
| Action On Pre-Eclampsia | 1013557 | https://action-on-pre-eclampsia.org.uk/ |
| Action on Smoking and Health - Wales | 1120834 | https://ash.wales/ |
| Action Duchenne | 1101971 | https://www.actionduchenne.org/ |
| Adfam | 1067428 | https://adfam.org.uk/ |
| Africa Advocacy Foundation | 1164778 | https://www.africadvocacy.org/ |
| African-Caribbean Leukaemia Trust | 1119516 | https://aclt.org/ |
| Age UK | 1128267 | https://www.ageuk.org.uk/ |
| Alex - The Leukodystrophy Charity | 1106008 | https://www.alextlc.org/ |
| ALK Positive Lung Cancer | 1181171 | https://www.alkpositive.org.uk/ |
| Alkaptonuria Society | 1101052 | https://akusociety.org/ |
| Allergy UK | 1094231 | https://www.allergyuk.org/ |
| Alliance for Heart Failure | N/A | https://allianceforheartfailure.org/ |
| Alzheimer Scotland | SC022315 | https://www.alzscot.org/ |
| Alzheimer’s Support | 1048314 | https://www.alzheimerswiltshire.org.uk/ |
| Alzheimer's Research UK | 1077089 | https://www.alzheimersresearchuk.org/ |
| Alzheimer's Society | 296645 | https://www.alzheimers.org.uk/ |
| Amyloidosis Patients Association | 1183624 | https://register-of-charities.charitycommission.gov.uk/charity-details/?regid=1183624&subid=0 |
| Anthony Nolan | 803716 | https://www.anthonynolan.org/ |
| Anticoagulation UK | 1090250 | https://register-of-charities.charitycommission.gov.uk/charity-details/?regid=1090250&subid=0 |
| AOFAC Foundation | 1162155 | https://aofacfoundation.org/ |
| Aplastic Anaemia Trust | 1107539 | https://www.theaat.org.uk/ |
| APS Support UK | 1138116 | https://aps-support.org.uk/ |
| Arthritis and Musculoskeletal Alliance | 1108851 | http://arma.uk.net/ |
| Aspens | 1171446 | https://www.aspens.org.uk/ |
| Association for Glycogen Storage Disease | 1132271 | https://agsd.org.uk/ |
| Asthma + Lung UK | 326730 | https://www.asthma.org.uk/ |
| Astriid | 1176645 | https://astriid.org/ |
| Atrial Fibrillation Association | 1122442 | Supporting children terminally ill |
| Axial Spondylitis International Federation | 1173902 | https://asif.info/ |
| Baby Lifeline | 1006457 | https://www.babylifeline.org.uk/ |
| Bath Institute for Rheumatic Diseases | 1040650 | https://www.birdbath.org.uk/ |

| | | |
|---|---|---|
| Batten Disease Family Association | 1084908 | http://www.bdfa-uk.org.uk/ |
| Bipolar UK | 293340 | https://www.bipolaruk.org/ |
| Bladder Health UK | 1149973 | https://bladderhealthuk.org/ |
| Bliss | 1002973 | https://www.bliss.org.uk/ |
| Blood Cancer Alliance | N/A | https://www.bloodcanceralliance.org/ |
| Blood Cancer UK | 216032 | https://bloodcancer.org.uk/ |
| BME Cancer Communities | 1182806 | https://www.bmecancer.com/ |
| Bowel Cancer UK | 1071038 | https://www.bowelcanceruk.org.uk/ |
| Brains Trust | 1114634 | https://brainstrust.org.uk/ |
| Breast Cancer Haven (The Haven) | 3291851 | https://www.breastcancerhaven.org.uk/ |
| Breast Cancer Now | 1160558 | https://breastcancernow.org/ |
| British Association ofr the Study of the Liver | 1106320 | https://www.basl.org.uk/ |
| British Heart Foundation | 225971 | https://www.bhf.org.uk/ |
| British Inherited Metabolic Disease Group | 1184024 | https://www.bimdg.org.uk/site/index.asp |
| British Liver Trust | 298858 | https://britishlivertrust.org.uk/ |
| British Paediatric Neurology Association | 1159115 | https://bpna.org.uk/ |
| British Porphyria Association | 1089609 | http://porphyria.org.uk/ |
| British Skin Foundation | 1171373 | https://www.britishskinfoundation.org.uk/ |
| British Society for Heart Failure | 1075720 | https://www.bsh.org.uk/ |
| British Society of Echocardiography | 1093808 | https://www.bsecho.org/ |
| British Thyroid Foundation | 1006391 | https://www.btf-thyroid.org/ |
| Cambridge Rare Disease Network | 1166365 | https://www.camraredisease.org/ |
| Cancer 52 | 7994413 | https://www.cancer52.org.uk/ |
| Cancer Black Care | 1086465 | https://www.cancerblackcare.org.uk/ |
| Cancer Focus Northern Ireland | 101307 | https://cancerfocusni.org/ |
| Cancer Research UK | 1089464 | https://www.cancerresearchuk.org/ |
| Cancer Support Scotland | SC012867 | https://www.cancersupportscotland.org/ |
| Cancer Support UK | 1105703 | https://cancersupportuk.org/ |
| CancerCare | 1120048 | https://cancercare.org.uk/ |
| Cara Trust | 328124 | https://www.madtrust.org.uk/project/the-cara-trust/ |
| Cardiomyopathy UK | 1164263 | https://www.cardiomyopathy.org/ |
| Carers UK | N/A | https://www.carersuk.org/ |
| Changing Faces | 1011222 | https://www.changingfaces.org.uk/ |
| Child Growth Foundation | 1172807 | https://childgrowthfoundation.org/ |
| Childhood Trust | 1154032 | https://www.childhoodtrust.org.uk/ |
| Children’s Cancer and Leukaemia Group | 1182637 | https://www.cclg.org.uk/ |
| Children’s HIV Association | 1122356 | https://www.chiva.org.uk/ |
| Children’s Trust | 288018 | https://www.thechildrenstrust.org.uk/ |
| Children's Burns Trust | 1082084 | https://www.cbtrust.org.uk/ |

| | | |
|---|---|---|
| Cholangiocarcinoma Charity | 1091915 | https://ammf.org.uk/ |
| Chronic Lymphocytic Leukaemia Support Association | 1178482 | https://www.cllsupport.org.uk/ |
| Coalition for Life-Course Immunisation | 1182662 | https://www.cl-ci.org/ |
| Confederation of Meningitis Organisations | 1091105 | https://www.comomeningitis.org/ |
| Contact a Family | 284912 | https://contact.org.uk/ |
| Crohn's and Colitis UK | 1117148 | https://www.crohnsandcolitis.org.uk/ |
| Cystic Fibrosis Trust | 1079049 | https://www.cysticfibrosis.org.uk/ |
| Dementia UK | 1039404 | https://www.dementiauk.org/ |
| Dementia Club UK | 1168397 | https://dementiaclubuk.org.uk/ |
| Diabetes UK | 215199 | https://www.diabetes.org.uk/ |
| Diana Award | 1117288 | https://diana-award.org.uk/ |
| DMD Pathfinders | 1155884 | https://www.pathfindersalliance.org.uk/ |
| Down Syndrome International | 1091843 | https://www.ds-int.org/ |
| Downs Syndrome Association | 1061474 | https://www.downs-syndrome.org.uk/ |
| Dravet Syndrome UK | 1128289 | https://www.dravet.org.uk/ |
| DrugFAM | 1123316 | https://www.drugfam.co.uk/# |
| Duchenne UK | 1147094 | https://www.duchenneuk.org/ |
| Dystonia UK | 1062595 | https://www.dystonia.org.uk/ |
| East North Hertfordshire NHS Trust | 1053338 | https://www.enherts-tr.nhs.uk/ |
| East Sussex Healthcare NHS Trust | 1058599 | https://www.esht.nhs.uk/ |
| Ecancer | 1176307 | https://ecancer.org/en/ |
| Eczema Outreach Support | SC042392 | https://www.eos.org.uk/ |
| Encephalitis Society | 1087843 | https://www.encephalitis.info/ |
| Epilepsy Action | 234343 | https://www.epilepsy.org.uk/?gclid=CjwKCAiAsNKQBhAPEiwAB-I5zXsMWEMg1x_J-blYzK3HQGZujp-zoejjkEA_sYpKqYxct5LuE_sV6hoC1t8QAvD_BwE |
| Epilepsy Consortium Scotland | N/A | http://www.epilepsyconsortiumscotland.co.uk/ |
| Epilepsy Research UK | 1100394 | https://epilepsyresearch.org.uk/ |
| Epilepsy Scotland | SC000067 | https://www.epilepsyscotland.org.uk/ |
| Epilepsy Society | 206186 | https://epilepsysociety.org.uk/ |
| Errol Mckellar Foundation | 1181574 | https://www.theerrolmckellarfoundation.com/ |
| European Parkinson's Disease Association | 1163211 | https://www.epda.eu.com/ |
| Eve Appeal | 1091708 | https://eveappeal.org.uk/ |
| Familial Hypercholesterolaemia Network | 1170731 | https://fheurope.org/ |
| FareShare | 1100051 | https://fareshare.org.uk/ |
| Favor UK | N/A | https://www.facesandvoicesofrecoveryuk.org/ |

| | | |
|---|---|---|
| Fertility Network UK | 1099960 | https://fertilitynetworkuk.org/ |
| Fight Bladder Cancer | 1157763 | https://www.fightbladdercancer.co.uk/ |
| Fight for Sight UK | 1111438 | https://www.fightforsight.org.uk/ |
| Findacure | 1149646 | https://www.rarebeacon.org/about-us/our-journey/ |
| Gauchers Association | 1095657 | https://www.gaucher.org.uk/ |
| Gene People | 1141583 | https://genepeople.org.uk/ |
| Genetic Alliance UK | 1114195 | https://geneticalliance.org.uk/ |
| GetYourBellyOut | 11276246 | https://getyourbellyout.org.uk/ |
| GIST Cancer UK | 1129219 | https://www.gistcancer.org.uk/ |
| Global Action on Men's Health | 1183428 | https://gamh.org/ |
| GO Girls | 1179108 | https://www.gogirlssupport.org/ |
| Gorlin Syndrome Group | 1197282 | https://gorlingroup.org/ |
| Guts UK | 1137029 | https://gutscharity.org.uk/ |
| Haemachromatosis UK | 1001307 | https://www.haemochromatosis.org.uk/ |
| Haemophilia Scotland | SC044298 | https://haemophilia.scot/ |
| Haemophilia Society | 288260 | https://haemophilia.org.uk/ |
| Headway East London | 1083910 | https://headwayeastlondon.org/ |
| Heart UK | 1003904 | https://www.heartuk.org.uk/ |
| Heartburn Cancer UK | 1136413 | https://www.heartburncanceruk.org/ |
| Helen & Douglas House | 1085951 | https://www.helenanddouglas.org.uk/ |
| Hepatitis C Coalition | N/A | http://www.hepc-coalition.uk/ |
| Hepatitis C Trust | 1104279 | http://hepctrust.org.uk/ |
| Hereditary Angioedema UK | 1152591 | https://www.haeuk.org/ |
| Hidradenitis Suppurativa Trust | 1177819 | https://painuk.org/members/charities/hidradenitis-suppurativa-trust/ |
| Histiocytosis UK | 1158789 | https://www.histiouk.org/ |
| HIV i-Base | 1081905 | https://i-base.info/ |
| HIV Scotland | SC033951 | https://www.hiv.scot/ |
| Human Story Theatre | 1173504 | https://humanstorytheatre.com/about-us/ |
| Huntington's Disease Association | 296453 | https://www.hda.org.uk/ |
| Huntington's Disease Youth Organization | 1145781 | https://en.hdyo.org/ |
| Immune Deficiency Patient Group of Wales | N/A | https://www.facebook.com/tommy.browne.idpgw/ |
| Immune Thrombocytopenia Support Association | 1064480 | https://www.itpsupport.org.uk/index.php/en/ |
| Independent Cancer Patients' Voice | 1138456 | http://www.independentcancerpatientsvoice.org.uk/ |
| Intensive Care Society | 1039236 | https://www.ics.ac.uk/ |
| International Alliance of Patients' Organizations | 1155577 | https://www.iapo.org.uk/ |
| International Brain Tumour Alliance | N/A | https://theibta.org/ |
| International Gaucher Alliance | 6653373 | https://gaucheralliance.org/home |
| International Headache Society | 1042574 | https://ihs-headache.org/en/ |
| International Longevity Centre UK | 1080496 | https://ilcuk.org.uk/ |

| | | |
|---|---|---|
| International Niemann-Pick Disease Alliance | 1150256 | https://www.inpda.org/ |
| International Patient Organisation for Primary Immunodeficiencies | 1058005 | https://ipopi.org/ |
| Invisible Cafe | N/A | https://theinvisiblecafe.co.uk/ |
| Isabel Hospice Limited | 1046826 | https://www.isabelhospice.org.uk/ |
| Jo's Cervical Cancer Trust | 1133542 | https://www.jostrust.org.uk/ |
| Juvenile Diabetes Research Foundation | 295716 | https://jdrf.org.uk/ |
| Karen Clifford Skcin cancer charity | 1150048 | https://www.skcin.org/ |
| Kent Autistic Trust | 801965 | https://www.kentautistictrust.org/ |
| Kent MS Therapy Centre | 801382 | https://kentmstc.org.uk/ |
| Kidney Cancer Support Network | 1164238 | https://actionkidneycancer.org/ |
| Kidney Cancer UK | 1120146 | https://www.kcuk.org.uk/ |
| Kidney Care UK | 270288 | https://www.kidneycareuk.org/ |
| Kidney Research UK | 252892 | https://www.kidneyresearchuk.org/ |
| Leukaemia CARE | 1183890 | https://www.leukaemiacare.org.uk/ |
| Leukaemia UK | 1154856 | https://www.leukaemiauk.org.uk/ |
| Liver4Life | 1152618 | https://www.liver4life.org.uk/ |
| Lupus UK | 1051610 | https://www.lupusuk.org.uk/ |
| Lymphoma Action | 1068395 | https://lymphoma-action.org.uk/about-us |
| Macmillan Cancer Support | 261017 | https://www.macmillan.org.uk/ |
| Macular Society | 2177039 | https://www.macularsociety.org/ |
| Maggie's Centres | SC024414 | https://www.maggies.org/ |
| Maypole Project | 1120163 | https://www.themaypoleproject.co.uk/ |
| MDS UK Support Group | 1145214 | https://mdspatientsupport.org.uk/ |
| Meath Epilepsy Charity | 200359 | https://www.meath.org.uk/ |
| Medics 4 Rare Diseases | 1183996 | https://www.m4rd.org/history/ |
| Melanoma Focus | 1124716 | https://melanomafocus.org/ |
| Melanoma Fund | 1085969 | https://www.melanoma-fund.co.uk/ |
| Melanoma UK | 1157635 | https://www.melanomauk.org.uk/ |
| Memorylane Eastbourne | 1163541 | https://www.memorylaneeastbourne.co.uk/ |
| Meningitis Now | 803016 | https://www.meningitisnow.org/ |
| Meningitis Research Foundation | 1091105 | https://www.meningitis.org/ |
| Menopause Support | N/A | https://menopausesupport.co.uk/ |
| Mental Health UK | 1170815 | https://mentalhealth-uk.org/ |
| Mersey Region Epilepsy Association | 504366 | https://www.epilepsymersey.org.uk/ |
| Mesothelioma UK | 1177039 | https://www.mesothelioma.uk.com/ |
| Metabolic Support UK | 1089588 | https://www.metabolicsupportuk.org/ |
| Migraine Trust | 1081300 | https://migrainetrust.org/ |
| Motor Neurone Disease Association | 294354 | https://www.mndassociation.org/ |
| Mouth Cancer Foundation | 1109298 | https://www.mouthcancerfoundation.org/ |
| MPN Voice | 1160316 | https://www.mpnvoice.org.uk/ |

| | | |
|---|---|---|
| Multiple Sclerosis International Federation | 1105321 | https://www.msif.org/ |
| Multiple Sclerosis Society UK | 1139257 | https://www.mssociety.org.uk/ |
| Multiple Sclerosis Therapy Centres | 1031690 | https://www.msntc.org.uk/ |
| Multiple Sclerosis Trust | 1088353 | https://mstrust.org.uk/ |
| Muscular Dystrophy UK | 205395 | https://www.musculardystrophyuk.org/ |
| My Name'5 Doddie Foundation | SC047871 | https://www.myname5doddie.co.uk/ |
| Myeloma UK | SC026116 | https://www.myeloma.org.uk/ |
| National AIDS Map | 1011220 | https://www.aidsmap.com/ |
| National AIDS Trust | 297977 | https://www.nat.org.uk/ |
| National Attention Deficit Disorder Information and Support Service | N/A | https://www.nhs.uk/services/service-directory/the-national-attention-deficit-disorder-information-and-support-service-addiss/N10498901 |
| National Axial Spondyloarthritis Society | 1183175 | https://nass.co.uk/ |
| National Cancer Research Institute | 1160609 | https://www.ncri.org.uk/ |
| National Eczema Society | 1009671 | https://eczema.org/ |
| National Federation of Prostate Cancer Support Groups | 1163152 | https://tackleprostate.org/ |
| National Kidney Federation | 1106735 | https://www.kidney.org.uk/ |
| National Rheumatoid Arthritis Society | 1134859 | https://nras.org.uk/ |
| National Voices | 1057711 | https://www.nationalvoices.org.uk/ |
| NAZ | 1014056 | https://www.naz.org.uk/ |
| Neuroendocrine Cancer UK | 1092386 | https://www.neuroendocrinecancer.org.uk/ |
| Neurological Alliance | 1039034 | https://www.neural.org.uk/ |
| New Life Counselling | NI005568 | https://www.amh.org.uk/ |
| NHS Charities Together | 1186569 | https://nhscharitiestogether.co.uk/ |
| Nicole & Jessica Rich Foundation | N/A | https://thenicolerichfoundation.org.uk/ |
| Niemann-Pick UK | 1144406 | https://www.npuk.org/ |
| North Bristol NHS Trust | 1055900 | https://www.nbt.nhs.uk/ |
| Oral Health Foundation | 263198 | https://www.dentalhealth.org/ |
| Orchid | 1080540 | https://orchid-cancer.org.uk/ |
| Osteoporosis Dorse | 1023507 | https://www.osteodorset.org.uk/ |
| Ovacome | 1159682 | https://www.ovacome.org.uk/ |
| Ovarian Cancer Action | 1109743 | https://ovarian.org.uk/ |
| Over the Wall | 1075361 | https://www.otw.org.uk/ |
| Pain Concern | SC023559 | https://painconcern.org.uk/ |
| Pancreatic Cancer Action | 1137689 | https://pancreaticcanceraction.org/ |
| Pancreatic Cancer UK | 1112708 | https://www.pancreaticcancer.org.uk/ |
| Parathyroid UK | N/A | https://parathyroiduk.org/ |
| Parkinson’s UK | 258197 | https://www.parkinsons.org.uk/ |
| Patient Information Forum | N/A | https://pifonline.org.uk/ |
| Patients Association | 1006733 | https://www.patients-association.org.uk/ |



Gentilini A, Parvanova I. *BMJ Open* 2023; 13:e071138. doi: 10.1136/bmjopen-2022-071138

| | | |
|---|---|---|
| Patients On Intravenous and Nasogastric Nutrition Therapy | 1157655 | https://pinnt.com/Home.aspx |
| Paula Carr Diabetes Trust | 801596 | https://www.paulacarrdiabetestrust.co.uk/ |
| PBC Foundation UK | SC025619 | https://www.pbcfoundation.org.uk/ |
| Pilgrims Hospice | 293968 | https://www.pilgrimshospices.org/ |
| Pituitary Foundation | 1058968 | https://www.pituitary.org.uk/ |
| Platelet Society | 1172202 | https://plateletsociety.co.uk/ |
| Police Community Clubs of Great Britain | N/A | https://www.policecommunityclubs.org/ |
| Polycystic Kidney Disease Charity | 1160970 | https://pkdcharity.org.uk/ |
| Pompe Support Network | 1186383 | https://pompe.uk/ |
| Positively UK | 1007685 | https://positivelyuk.org/ |
| Primary Immunodeficiency UK | 1193166 | http://www.immunodeficiencyuk.org/ |
| Progress Educational Trust | 1139856 | https://www.progress.org.uk/ |
| Progressive Supranuclear Palsy Association | 1037087 | https://pspassociation.org.uk/ |
| Prostate Cancer UK | 1005541 | https://prostatecanceruk.org/ |
| Psoriasis Association | 1180666 | https://www.psoriasis-association.org.uk/ |
| Pulmonary Hypertension Association UK | 1120756 | https://www.phauk.org/ |
| Pumping Marvellous Foundation | 1151848 | https://www.pumpingmarvellous.org/ |
| Rain Trust | N/A | https://www.nhs.uk/services/service-directory/rain-trust/N10972097 |
| Rainbow Trust Children's Charity | 1070532 | https://www.rainbowtrust.org.uk/ |
| Rapid Effective Assistance For Children With Potentially Terminal Illness | 802440 | https://reactcharity.org/ |
| Red Rose Recovery | 1152474 | https://redroserecovery.org.uk/ |
| Release | 801118 | https://www.release.org.uk/ |
| Rethink Mental Illness | 271028 | https://www.rethink.org/ |
| Retina UK | 1153851 | https://retinauk.org.uk/about/ |
| Revive Multiple Sclerosis Support | SC022886 | https://www.revivemssupport.org.uk/ |
| Roy Castle Lung Cancer Foundation | 1046854 | https://roycastle.org/ |
| Royal Free Charity | 1165672 | https://royalfreecharity.org/ |
| Royal National Institute of Blind People | 226227 | https://www.rnib.org.uk/ |
| Royal Osteoporosis Society | 1102712 | https://theros.org.uk/ |
| Ruth Strauss Foundation | 1183221 | https://ruthstraussfoundation.com/ |
| Salivary Gland Cancer UK | 1182762 | https://www.salivaryglandcancer.uk/ |
| SANE | 296572 | https://www.sane.org.uk/ |
| Sarcoma UK | 1139869 | https://sarcoma.org.uk/ |
| Scleroderma and Raynauds UK | 1161828 | https://www.sruk.co.uk/ |
| Scottish Drugs Forum | SC008075 | https://www.sdf.org.uk/ |



Gentilini A, Parvanova I. *BMJ Open* 2023; 13:e071138. doi: 10.1136/bmjopen-2022-071138

| | | |
|---|---|---|
| Scottish Families Affected by Alcohol & Drugs | N/A | https://www.sfad.org.uk/ |
| Scottish Huntington's Association | SC010985 | https://hdscotland.org/ |
| Shift.MS | 1117194 | https://shift.ms/ |
| Shine Cancer Support | 1146902 | https://shinecancersupport.org/ |
| Sickle Cell Society | 1046631 | https://www.sicklecellsociety.org/ |
| Skin Conditions Campaign Sco tland | SC030004 | https://www.disabilityscot.org.uk/organisat ion/skin-conditions-campaign-scotland/ |
| Society for Mucopolysaccharide Diseases | 1143472 | https://www.mpssociety.org.uk/ |
| Somerville Foundation | 1138088 | https://sfhearts.org.uk/ |
| Sophia Forum | 1131629 | https://sophiaforum.net/ |
| Spinal Muscular Atrophy Support UK | 1106815 | https://smauk.org.uk/ |
| St Elizabeths Centre | 1176777 | https://www.stelizabeths.org.uk/ |
| Stroke Association | 211015 | https://www.stroke.org.uk/ |
| Swallows Head and Neck Cancer Charity | 1149794 | https://www.theswallows.org.uk/ |
| Target Ovarian Cancer | 1125038 | https://targetovariancancer.org.uk/ |
| Tenovus Cancer Care | 1054015 | https://www.tenovuscancercare.org.uk/ |
| Terrence Higgins Trust | 288527 | https://www.tht.org.uk/ |
| Thrombosis UK | 1090540 | https://thrombosisuk.org/news/post.php?s= 2021-10-11-thrombosis-uk-winner-of-activity-of-the-year-award-2021 |
| Tiny Tickers | 1078114 | https://www.tinytickers.org/ |
| Together for Short Lives | 1144022 | https://www.togetherforshortlives.org.uk/ |
| TRACTion Cancer Support | SCO048145 | https://www.tractioncancersupport.org/ |
| Trekstock | 1132421 | https://www.trekstock.com/ |
| Trevi | 1075433 | https://trevi.org.uk/ |
| Tuberous Sclerosis Association | 1039549 | https://tuberous-sclerosis.org/ |
| Turner Syndrome Support Society | 1080507 | https://tss.org.uk/ |
| Twins Trust | 1076478 | https://twinstrust.org/ |
| UK Breast Cancer Group | 1177296 | https://ukbcg.org/ |
| UK Lung Cancer Coalition | N/A | https://www.uklcc.org.uk/ |
| UK Primary Immune-deficiency Patient Support | 1148789 | https://ukpips.org.uk/ |
| UK Thalassaemia Society | 275107 | https://ukts.org/ |
| University of Newcastle Institute of Neuroscience | N/A | https://www.ncl.ac.uk/medical-sciences/research/research-themes/neuroscience/ |
| Urology Cancer Research and Education | 1120887 | http://www.ucare-oxford.org.uk/ |
| Versus Arthritis | 207711 | https://www.versusarthritis.org/ |
| Waldenstrom's Macroglobulinaemia UK | 1187121 | https://wmuk.org.uk/ |
| White Chapel Mission | 227905 | https://whitechapel.org.uk/ |
| Working with Cancer | 9092152 | https://workingwithcancer.co.uk/ |
| Young Epilepsy | 311877 | https://www.youngepilepsy.org.uk/ |



Gentilini A, Parvanova I. *BMJ Open* 2023; 13:e071138. doi: 10.1136/bmjopen-2022-071138

**Inclusion/exclusion of patient organisations**

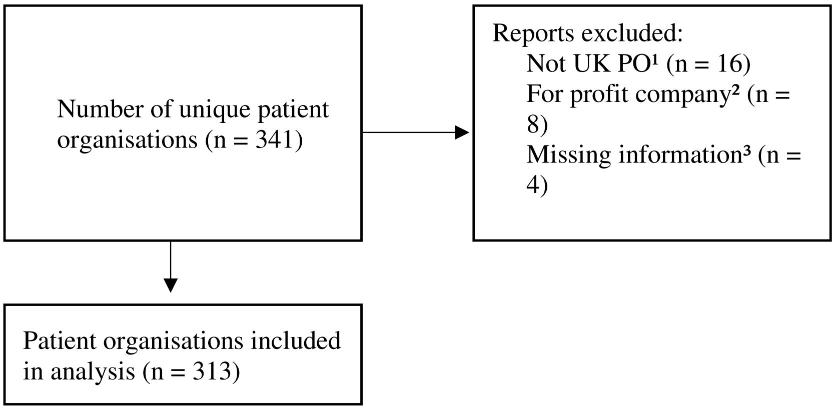


[1]Not aligned with geographical scope e.g. Irish, US-based
[2]Not aligned with EFPIA's definition of patient organisation
[3]Organisations for whose nature is unclear i.e. patient organisation website could not be identified

## Additional tables and figures

**Figure 2. Histogram of unique companies funding patient organisations in 2020, broken down by rarity of disease**

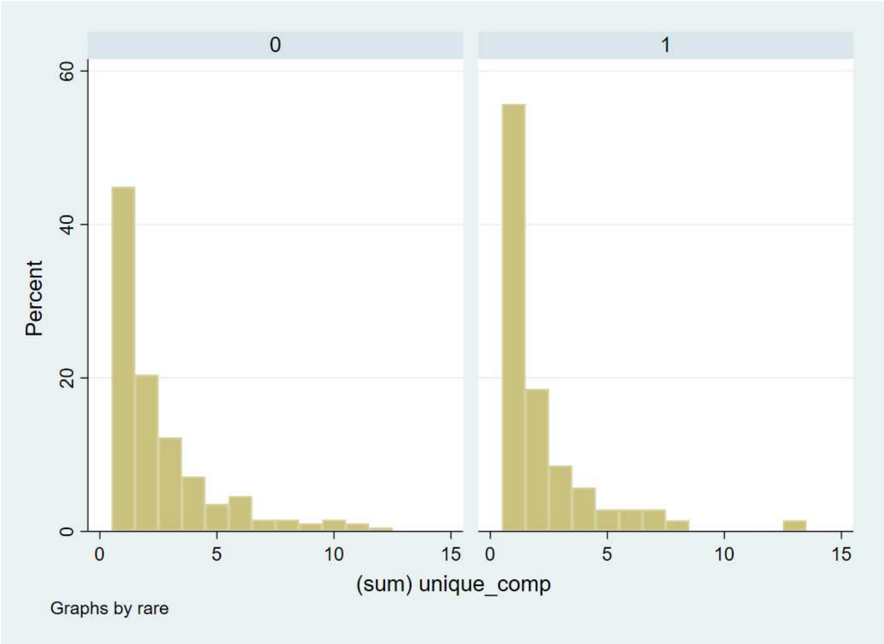


**Figure 3. Histogram of share of overall industry funding to patient organisations coming from each contributing company in 2020, broken down by rarity of disease**

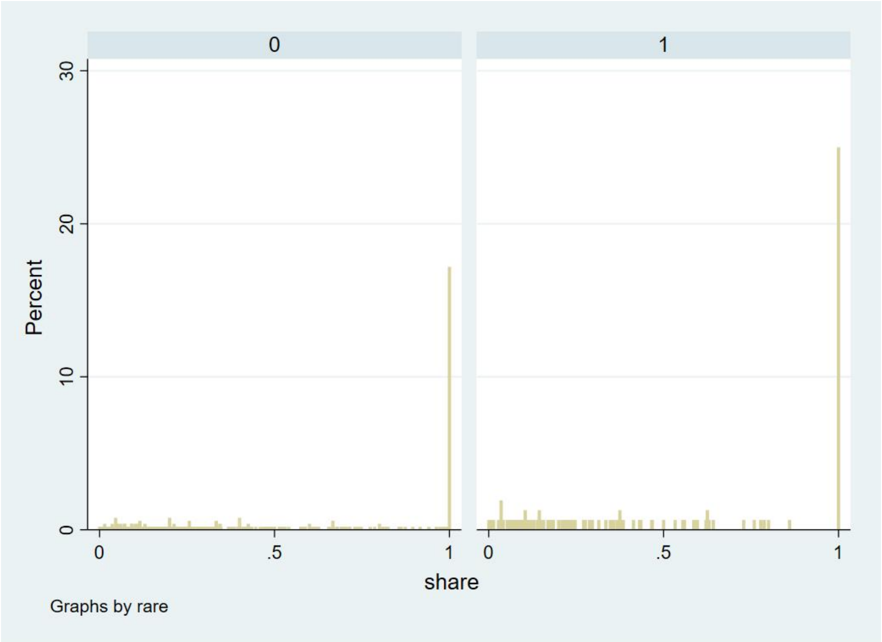




Gentilini A, Parvanova I. *BMJ Open* 2023; 13:e071138. doi: 10.1136/bmjopen-2022-071138

**Figure 4. Histogram of share of industry funding of each organisation comprised by the single highest payment in 2020, broken down by rarity of disease**

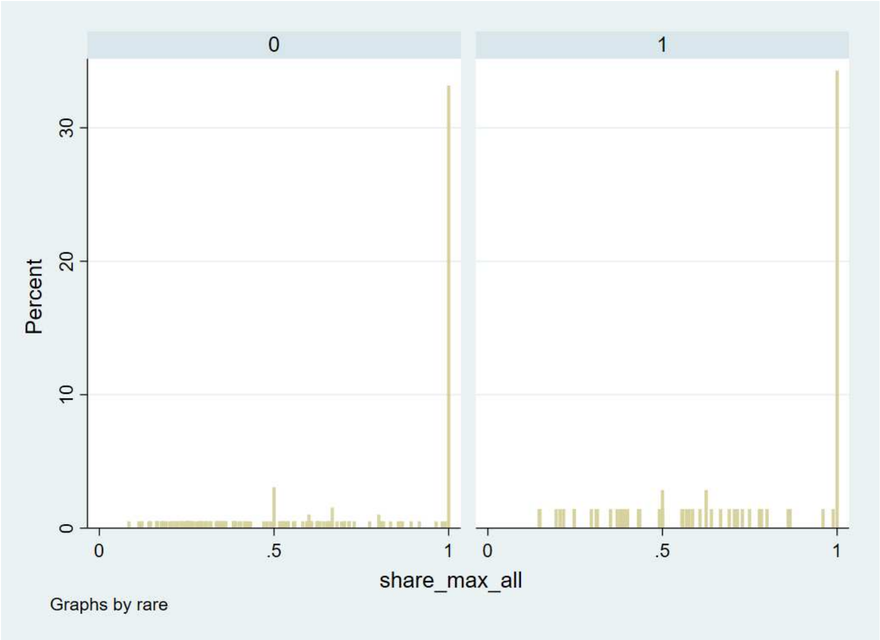




Gentilini A, Parvanova I. *BMJ Open* 2023; 13:e071138. doi: 10.1136/bmjopen-2022-071138

## Sub-group analyses

### <u>Excluded patient organisations</u>

66 payments made 28 to patient organisaitons were excluded from the analysis as they did not match EFPIA's definition of *"not-for-profit organisations, mainly composed of patients and/or caregivers, that represent and/or support the needs of patients and/or caregivers"*.

Figure 5 illustrates the reasons for patient organisations exclusion. Most of the excluded patient organisations were for profit organisations (47%; n=31), followed by not UK-based (42%; n=28) and organisations for which no information could be found online (11%; n=7).

Non-UK patient organisations mostly comprised international alliances of patient organisations, European or Irish organisations. We classified organisations as for-profit if they appeared in the UK government repository of companies[1] as *private limited companies.* Care homes, consultancies and rehabilitation clinics were the most prominent in this category.

Overall, payments to excluded patient organisations amounted to £869,677, about 4% of the included payments (Figure 6).

**Figure 5. Excluded patient organisations by reason of exclusion**

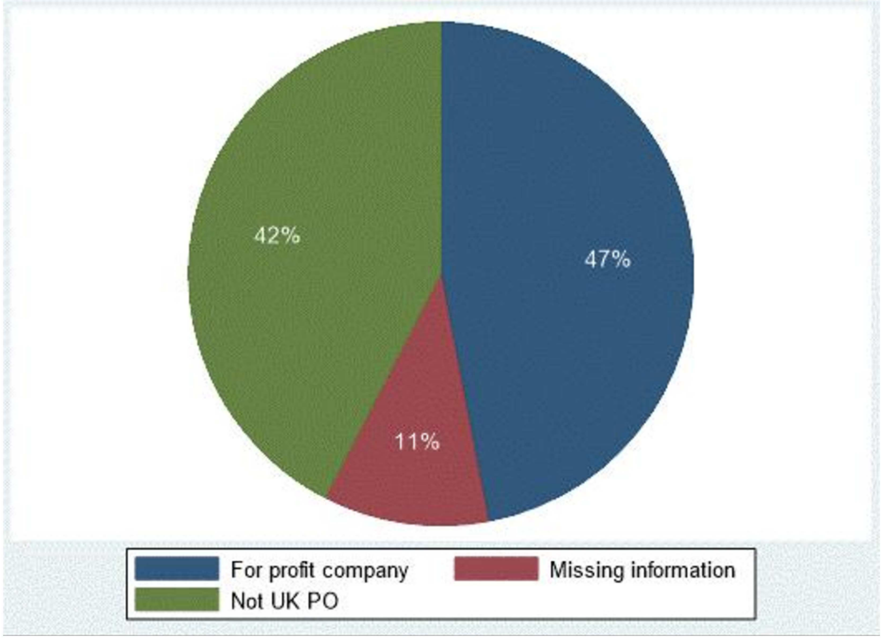


[1] https://find-and-update.company-information.service.gov.uk/



Gentilini A, Parvanova I. *BMJ Open* 2023; 13:e071138. doi: 10.1136/bmjopen-2022-071138

**Figure 6. Payments to included and excluded patient organisations**

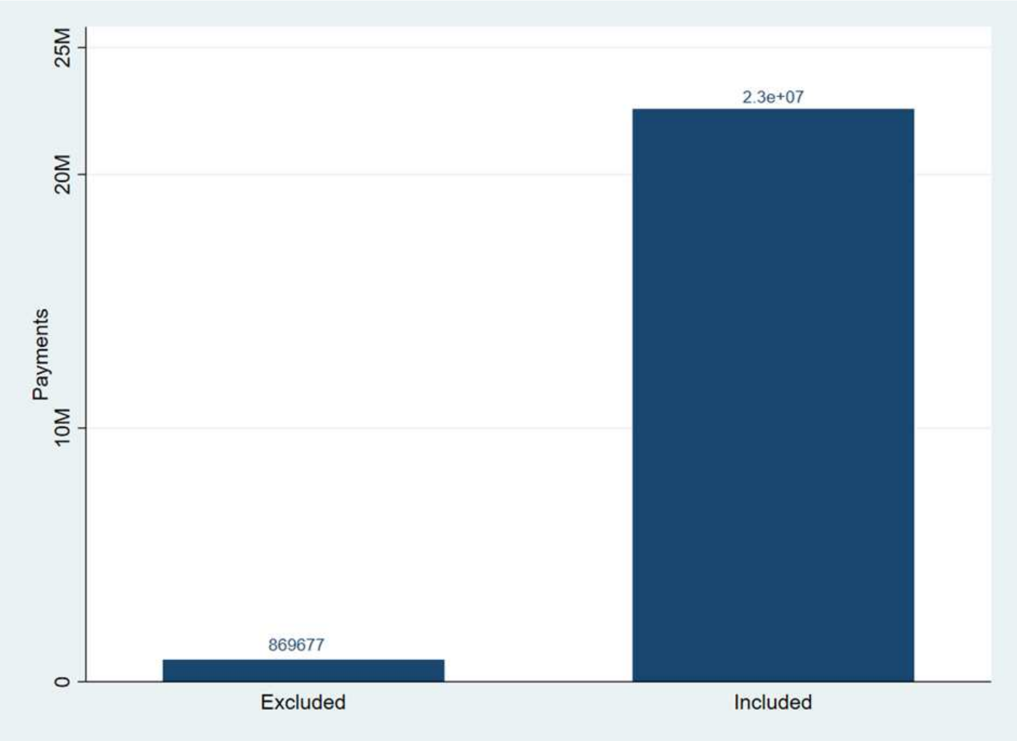

## References

1. PMCPA. ABPI Code of Practice 2021 [Available from: https://www.pmcpa.org.uk/the-code/2021-interactive-abpi-code-of-practice/.
2. Rickard E, Carmel E, Ozieranski P. Comparing pharmaceutical company payments in the four UK countries: a cross-sectional and social network analysis. *BMJ Open* 2023;13(3):e061591. doi: 10.1136/bmjopen-2022-061591
3. Ozieranski P, Rickard E, Mulinari, Shai. Exposing drug industry funding of UK patient organisations. *BMJ* 2019;365:l1806. doi: 10.1136/bmj.l1806
4. HMRC. HMRC yearly average and spot rates: HM Revenue and Customs;  [Available from: https://www.gov.uk/government/publications/exchange-rates-for-customs-and-vat-yearly.
5. HMRC. HMRC yearly average and spot rates: HM Revenue and Customs; 2022 [Available from: https://www.gov.uk/government/publications/exchange-rates-for-customs-and-vat-yearly.
6. Orphanet. The portal for rare diseases and orphan drugs 2022 [Available from: https://www.orpha.net/consor/cgi-bin/Disease_Search_Simple.php?lng=EN.
7. NICE. Policy on declaring and managing interests for NICE advisory committees, 2018.
8. WHO. ICD-11 for Mortality and Morbidity Statistics 2022 [Available from: https://icd.who.int/browse11/l-m/en#/http://id.who.int/icd/entity/465177735?view=G0.
