# Supplementary material for: Industry funding of patient organisations in the UK: a retrospective study of commercial determinants, funding concentration and disease prevalence
Source: BMJ Open. 2023 Jun 27;13(6):e071138. doi: 10.1136/bmjopen-2022-071138 (PMC10410975; doi:10.1136/bmjopen-2022-071138)
Supplement: Author's manuscript [file bmjopen-2022-071138.draft_revisions.pdf]

BMJ Open is committed to open peer review. As part of this commitment we make the peer review history of every article we publish publicly available.

When an article is published we post the peer reviewers' comments and the authors' responses online. We also post the versions of the paper that were used during peer review. These are the versions that the peer review comments apply to.

The versions of the paper that follow are the versions that were submitted during the peer review process. They are not the versions of record or the final published versions. They should not be cited or distributed as the published version of this manuscript.

BMJ Open is an open access journal and the full, final, typeset and author-corrected version of record of the manuscript is available on our site with no access controls, subscription charges or pay-per-view fees (http://bmjopen.bmj.com).

If you have any questions on BMJ Open's open peer review process please email info.bmjopen@bmj.com

Industry funding of patient organisations in the United Kingdom: A retrospective study of commercial determinants, funding concentration and disease prevalence

Journal:	*BMJ Open*
Manuscript ID	bmjopen-2022-071138
Article Type:	Original research
Date Submitted by the Author:	20-Dec-2022
Complete List of Authors:	Gentilini, Arianna; The London School of Economics and Political Science, Department of Health Policy<br>Parvanova, Iva; The London School of Economics and Political Science, Department of Health Policy
Keywords:	Health policy < HEALTH SERVICES ADMINISTRATION & MANAGEMENT, PUBLIC HEALTH, Health Equity

SCHOLARONE™
Manuscripts

I, the Submitting Author has the right to grant and does grant on behalf of all authors of the Work (as defined in the below author licence), an exclusive licence and/or a non-exclusive licence for contributions from authors who are: i) UK Crown employees; ii) where BMJ has agreed a CC-BY licence shall apply, and/or iii) in accordance with the terms applicable for US Federal Government officers or employees acting as part of their official duties; on a worldwide, perpetual, irrevocable, royalty-free basis to BMJ Publishing Group Ltd ("BMJ") its licensees and where the relevant Journal is co-owned by BMJ to the co-owners of the Journal, to publish the Work in this journal and any other BMJ products and to exploit all rights, as set out in our licence.

The Submitting Author accepts and understands that any supply made under these terms is made by BMJ to the Submitting Author unless you are acting as an employee on behalf of your employer or a postgraduate student of an affiliated institution which is paying any applicable article publishing charge ("APC") for Open Access articles. Where the Submitting Author wishes to make the Work available on an Open Access basis (and intends to pay the relevant APC), the terms of reuse of such Open Access shall be governed by a Creative Commons licence – details of these licences and which Creative Commons licence will apply to this Work are set out in our licence referred to above.

Other than as permitted in any relevant BMJ Author's Self Archiving Policies, I confirm this Work has not been accepted for publication elsewhere, is not being considered for publication elsewhere and does not duplicate material already published. I confirm all authors consent to publication of this Work and authorise the granting of this licence.

For peer review only

Industry funding of patient organisations in the United Kingdom: A retrospective study of commercial determinants, funding concentration and disease prevalence

Arianna Gentilini and Iva Parvanova

Department of Health Policy, London School of Economics and Political Science, London, UK

Corresponding author

Arianna Gentilini

Department of Health Policy
Cowdray House, COW.4.09
London School of Economics
Houghton street
London WC2A 2AE
England
Email: a.gentilini@lse.ac.uk

For peer review only - http://bmjopen.bmj.com/site/about/guidelines.xhtml

Industry funding of patient organisations in the United Kingdom: A retrospective study of commercial determinants, funding concentration and disease prevalence

Abstract

Objectives – To assess the relationship between UK-based patient organisation funding and companies' commercial interests in rare and non-rare diseases from 2018 to 2020.

Design – Retrospective analysis of the value and volume of payments from pharmaceutical companies to patient organisations in the UK matched with data on the conditions supported by patient organisations and drugs in companies' approved portfolios and research and development pipelines.

Setting – UK.

Participants – 60 pharmaceutical companies making financial transfers to 483 UK-based patient organisations.

Main outcome measures – Alignment between the commercial interests of pharmaceutical companies and the disease area focus of patient organisations; difference in the volume and value of transfers to patient organisations broken down by prevalence of conditions; industry funding concentration, measured as the number of companies funding each patient organisations, the share of overall industry funding coming from each contributing company and the share of industry funding of each organisation comprised by the single highest transfers.

Results – 3,155 transfers were made by 60 companies to 429 patient organisations. Almost all funds (97%) from pharmaceutical companies were directed to patient organisations that are aligned with companies' approved drug portfolios and research and development pipelines. Despite rare diseases affecting less than 5% of the UK population, 25% of all transfers were directed to patient organisations which target such conditions. Patient organisations focusing on rare diseases relied on transfers from fewer companies (*p-value* = 0.008) compared to organisations focusing on non-rare diseases.

Conclusions – Companies predominantly funded patient organisations operating in therapeutic areas relevant to companies' portfolio or drug development pipeline. Patient organisations focusing on rare diseases received more funding relative to the number of patients affected by these conditions and relied more heavily on transfers from fewer companies compared to organisations targeting non-rare diseases. Increased independence of patient organisations could help avoiding conflicts of interest.

For peer review only - http://bmjopen.bmj.com/site/about/guidelines.xhtml

Strengths and limitations of this study

- We develop a methodology to determine the concordance between commercial interests of pharmaceutical companies and disease areas supported by patient organisations
- We present a comparative analysis of industry funding to patient organisations depending on the prevalence of the disease(s) they support.
- Our analysis focuses on a recent time period which might differ from historical trends.
- Financial transfers from pharmaceutical companies to patient organisations might be underreported. However, underreporting is expected to impact the absolute value of financial transfers rather than the relative difference.

For peer review only

Introduction

Patient organisations, which represent and support the needs of patients, play an important role in the development, regulatory review, and adoption of new drugs. During research and development, patient organisations effectively advocate for resources to be directed to conditions where unmet need is highest.[1 2] Patient organisations support research design and planning, helping to identify patient-relevant study endpoints.[2] Patient organisations also represent patient views and preferences at the time of regulatory review and health technology assessment of new drugs.[3 4] For example, during technology appraisals conducted by the National Institute for Health and Care Excellence (NICE), which makes funding recommendations for the English National Health Service (NHS), patients, and organisations representing the interests of patients, provide testimonies of their first-hand experiences on how the disease affects them and those around them.[5] Finally, when drugs are launched, patient organisations contribute to dissemination of research results to patient community and clinicians, and offer support and information on therapies available.[2 6]

Given the role of patient organisations across all stages of drug development, approval and access, it is vital to understand their financial ties with pharmaceutical companies. Previous studies documented the large number and high value of payments from pharmaceutical companies to patient organisations,[6-9] the uneven distribution between and within therapeutic areas,[7 9] the concentration of payments coming from a small number of pharmaceutical firms.[6-11]

What remains unknown is the alignment between the commercial interests of pharmaceutical companies and patient organisations' activities. Previous literature has shown that industry prioritises commercially attractive conditions.[7] Moreover, research in different settings suggested that having a drug marketed for a certain disease is associated with an increase in industry funding to patient organisations operating in the same area.[9] However, the question of whether companies fund patient organisations operating in therapeutic areas relevant to companies' approved drug portfolios and research and development pipelines remains unanswered.

Another gap in the literature relates to the dynamics between the pharmaceutical industry and patient organisations supporting rare vs. non-rare conditions. Patient organisations focusing on rare conditions serve different purposes than those focusing on non-rare diseases. First, patient organisations focusing on rare diseases are mostly made up of patients, their families and carers.[12] This makes them uniquely placed to share first-hand experiences that helps steering research and approval decisions.[13 14] For example, in appraisals for extremely rare diseases, NICE places particular importance on patients' testimonies, as they help with defining target populations and determining treatment benefits.[15] Second, patient organisations targeting rare diseases support recruitment and enrolment of patients in clinical trials.[13] Third, such organisations have been instrumental in advocating for scientific support and economic incentives to stimulate innovation in rare diseases, which ultimately led to the enactment of legislation in multiple settings, such as the EU Regulation on Orphan Medicinal Products.[16 17]

We evaluated the concordance between the commercial interests of pharmaceutical companies and patient organisations' activities. We also sought to characterise the financial relations

For peer review only - http://bmjopen.bmj.com/site/about/guidelines.xhtml

between the pharmaceutical industry and patient organisations focusing on rare versus non-rare diseases in the UK using publicly available data on transfers of value between 2018 and 2020. We analysed the volume, value of transfers to patient organisations according to their disease area of interest and its rarity. Lastly, we examined the concentration of industry funding, namely how many companies funded each patient organisation and the extent to which organisations might have been reliant on funding from a single company.

For peer review only

For peer review only - http://bmjopen.bmj.com/site/about/guidelines.xhtml

Methods

Data on industry payments

We used the Disclosure UK patient organisation gateway (in January 2022) to retrieve data on transfers from the pharmaceutical industry to patient organisations from 2018 to 2020.[18] The gateway was launched in 2020 and is a collection of hyperlinks to companies' disclosure of transfers to patient organisations. Disclosing transfers to patient organisations is a requirement of Clause 29 of the Association of British Pharmaceutical Industry (ABPI) Code of Practice.[19] Companies that sign up to abide by the ABPI Code accept the jurisdiction of the Prescription Medicines Code of Practice Authority (code regulator), which extends beyond those who are ABPI members. This requirement therefore affects virtually all pharmaceutical companies operating in the UK. Companies might be sanctioned by the ABPI if they do not disclose their transfers.[19] We screened the websites of all pharmaceutical companies listed in the Disclosure UK database to ensure all transfers were captured. If transfers were not disclosed in Disclosure UK nor in the company's website, we assumed the company was did not make any transfers to patient organisations in a given year.

One investigator (AG) extracted transfer disclosures from the companies' websites. These comprised the name of the patient organisation, the year when the transfer was made, the reason for the transfer and its value in the currency reported by the disclosing company. All transfers were first adjusted for inflation using the ONS Consumer Price Index.[20] When reported in different currencies, such as United States Dollars (USD), Swiss Franc (CHF), Swedish Krona (SEK), Norwegian Krone (NKK) and Danish Krone (DKK), the value of the transfer was converted to Great British Pounds (GBP), using the ONS historical yearly conversion rates. [21] [22] We reported all transfers in 2020 GBP. Two in-kind transfers with a monetary value of zero were excluded from the analysis.

Data on patient organisations

We retrieved data on patient organisations from their websites. Details on the therapeutic area they advocated for – proxied by International Classification of Diseases Version 11 (ICD-11) codes – and whether the condition(s) was rare or non-rare were also extracted. Conditions were considered rare if they appeared in the Orphanet database of rare diseases.[24] Orphanet is a unique platform and repository of data on rare diseases and orphan drugs. Patient organisations that were not disease specific, such as hospital charities, carers organisations and hospices, or that did not match the European Federation of Pharmaceutical Industries and Associations (EFPIA) definition of what constitutes a patient organisation were excluded from the analysis. We chose the EFPIA's definition for the following reasons. First, other commonly used definitions, such as the one from the European Medicines Agency (EMA), refer to the structure of patient organisations' governing bodies, which have to consist of over 50% patients.[25] Considering the high number of patient organisations included in our analysis, this requirement was challenging – if not impossible – to verify. Second, EFPIA's definition indicates what the pharmaceutical industry considers to be a patient organisation. Therefore, it helped us minimize selection bias issues as it includes a wide range of organisations. We excluded excluding 181 transfers to patient organisations that did not match EFPIA's definition. Sub-group analyses on excluded organisations can be found in the Supplemental Material.

For peer review only - http://bmjopen.bmj.com/site/about/guidelines.xhtml

Determining commercial interests

We assessed whether – and the extent to which – a pharmaceutical company holds an interest in the disease supported by a patient organisation. We adapted the definition of 'interest' provided by NICE [26]. An interest is when there is, or could be perceived to be, an opportunity for a pharmaceutical company to benefit in the disease area where the patient organisation operates. This could include cases where the pharmaceutical company has a drug developed or in development for a condition targeted by the patient organisation, or where a drug in the company's portfolio or pipeline is restricted to a specific population affected by the disease supported by the patient organisation.

To establish whether an interest existed or not, we first classified the conditions targeted by patient organisations to ICD-11 codes using the online ICD-11 database.[27] ICD-11 codes are mutually exclusive, exhaustive and are arranged as a single hierarchical tree, from level one (most general e.g., *neoplasms*) to five (most specific, e.g. *plasma cell myeloma*). This means that specific diseases are nested within broader classifications.

We then searched companies' annual reports, websites and the ClinicalTrials.gov registry to determine whether each company had an interest in the condition targeted by the patient organisation receiving the transfer. Figure 1 schematically illustrates the approach taken to understand whether – and the degree to which – a company has an interest in the conditions (*definitely yes, probably yes, no*). For example, if *Company X* declares in its annual report having a drug in development for multiple myeloma and made a transfer to *Blood Cancer UK*, this would be coded as *probably yes,* as the company has a product in its pipeline or portfolio nested within a broader class of conditions targeted by the patient organisation. Conversely, should *Company X* have made a transfer to *Myeloma UK,* this would have been coded as *definitely yes,* as there is perfect alignment between the condition targeted by the patient organisation and by *Company X's* drug. Cases in which a company's interest in a certain condition could not be identified were coded as *no.* These, however, were due to limitations in data availability and therefore did not indicate that there was no company interest. Data on pharmaceutical companies' portfolio and pipeline were retrieved from their latest annual reports, company websites and ClinicalTrials.gov.[23]

One investigator (AG) initially coded all data, while the other (IP) blindly re-coded a 30% random sample of transfers to validate the data collection process and minimise the risk of reporting errors. Any disagreement was discussed until consensus was reached.

Analysis of industry funding concentration

We assessed the concentration of industry funding received by patient organisations. In particular, we calculated (1) the number of companies funding each patient organisations, (2) the share of overall industry funding coming from each contributing company and (3) the share of industry funding of each organisation comprised by the single highest transfers.

The Supplemental Material provides further details on the data collection and how the outcomes were constructed. Descriptive statistics and tests, such as ranges and K-sample tests, were presented in the analysis. These statistics were preferred over the mean in light of the

For peer review only - http://bmjopen.bmj.com/site/about/guidelines.xhtml

skewed distribution of the data analysed. All analyses and data visualisations were performed using Stata 17 and RStudio (*ggplot2* package), respectively.

Patient and public involvement

Patients were not involved in this study as our analyses focused on patient organisations as institutional actors rather than single patients with specific conditions. We plan to disseminate key findings from our analysis to patients and members of the public.

For peer review only

For peer review only - http://bmjopen.bmj.com/site/about/guidelines.xhtml

Results

Between 2018 and 2020, 60 companies made 3,155 transfers to 429 patient organisations in the study period, amounting to £42 million. The value of the transfers rose significantly over time, from £10.3 million in 2018 to £16.8 million in 2020.

Overall, *diseases of the nervous system* (£8.2 million) was the most funded therapeutic area over time, followed by *neoplasms* (£7.9 million) and *endocrine, nutritional or metabolic diseases* (£5.3 million). About 50% of the transfers made to organisations targeting *diseases of the nervous system* were made in 2020 alone. Sanofi, Novartis, Pfizer, UCB and Janssen were the top five funders over the study period (Figure 2). These companies contributed to between 37% and 44% of all transfers, decreasing over time.

Table 1 in the Supplemental Material summarises the number and value of transfers to patient organisations.

Companies' interest in financial transfers to patient organisations

Between 2018-2020, 96% of the transfers were directed to patient organisations that were judged to be aligned with their portfolio and pipeline. Only 4% of transfers were made to organisations that focused on conditions that could not be linked to a product in the funder's portfolio or pipeline. Table 1 shows the volume and value of transfers, broken down by the company's interest variable and whether patient organisations targeted a rare or non-rare disease. Transfers to patient organisations targeting a disease for which the company has a product developed or in development (*definitely yes*) made up around 55% regardless of the rarity of the condition targeted.

The monetary value of transfers coded as *definitely yes* accounted for 69% of the overall transfer value for patient organisations targeting rare diseases versus 63% for organisations focusing on non-rare conditions. When transfers coded as *probably yes* were included, this share increased to 97% for both patient organisations focusing on rare and non-rare diseases.

For peer review only

Table 1. Volume and value of transfers by company interests

		**Volume**; n(%)				**Value**: £(%)			
**Patient organisation type**	**Company's interest**	*2018*	*2019*	*2020*	*Overall*	*2018*	*2019*	*2020*	*Overall*
Rare	Definitely yes	79 (53%)	125 (58%)	136 (54%)	340 (54%)	£1,602,340 (69%)	£2,372,533 (72%)	£2,750,425 (66%)	£6,725,300 (69%)
	Probably yes	59 (40%)	79 (38%)	124 (45%)	262 (41%)	£635,393 (27%)	£781,688 (24%)	£1,296,449 (31%)	£2,713,531 (28%)
	No	10 (7%)	11 (5%)	13 (5%)	34 (5%)	£91,282 (4%)	£126,779 (4%)	£134,015 (3%)	£352,078 (4%)
Non-rare	Definitely yes	408 (56%)	425 (54%)	443 (55%)	1,276 (55%)	£5,350,194 (67%)	£5,921,218 (65%)	£7,850,393 (62%)	£19,121,806 (62%)
	Probably yes	304 (42%)	339 (43%)	334 (41%)	977 (42%)	£2,409,093 (31%)	£3,032,911 (33%)	£4,385,282 (35%)	£9,827,287 (35%)
	No	17 (2%)	24 (3%)	30 (4%)	71 (3%)	£231,784 (3%)	£155,331 (2%)	£334,352 (3%)	£721,468 (3%)

Notes: *Definitely yes* indicates transfers directed to patient organisations that operated in a disease area (ICD-11 level 4 or higher) for which the company has a product in its portfolio or pipeline. *Probably yes* indicates directed to patient organisations that operated in a disease area (ICD-11 level 3 or lower) for which the company has a product in its portfolio or pipeline. *No* refers to directed to patient organisations that operated in a disease area for which no link could be found to the company's portfolio or pipeline. The higher the ICD-11, the more specific the condition. For example, if the ICD-11 level 4 is *Plasma cell neoplasms,* level 2 would be *Neoplasms of hematopoietic or lymphoid tissues.* Further details on how this variable was constructed can be found in the Supplemental Material.

For peer review only - http://bmjopen.bmj.com/site/about/guidelines.xhtml

Industry funding of patient organisations focusing on rare vs. non-rare conditions

Of the £42 million in transfers from industry to patient organisations, £9.8 million (23%; n=635) were directed to organisations focusing on rare diseases while £29.7 million (71%; n=2,323) to organisations supporting non-rare conditions. The remaining 6% were directed to non-disease-specific patient organisations, which were excluded from the main analysis.

From 2018 to 2020, the transfer to patient organisations targeting rare diseases increased more compared to those focusing on more prevalent conditions (80% vs 57%). Median transfers received by patient organisations were significantly different ($p<0.001$) depending on the rarity of the disease they focused on, with rare patient organisations receiving higher transfers.

Among the top five recipients overall in 2018 and 2019, two focused on rare diseases (Myeloma UK and the Cystic Fibrosis Trust). In 2020 no organisation targeting rare conditions appeared in the top five recipients. Figure 3 shows therapeutic areas in order from most to least funded, broken down by rarity of disease targeted. In the case of organisations focusing on rare diseases, *neoplasms* and *endocrine, nutritional or metabolic disease* received most funds across years. Together, the top three most funded disease areas represented more than half of overall funding. When looking at the conditions that attracted most funding, multiple sclerosis was first (£4.1 million), followed by diabetes (£2.4 million) and epilepsy (£1.7 million). Cystic fibrosis and multiple myeloma were the only rare diseases that were among the top ten most funded conditions overall, attracting £1.3 and £1.2 million, respectively (Table 2).

For peer review only - http://bmjopen.bmj.com/site/about/guidelines.xhtml

1 **Table 2. Number of funding companies, top funder and highest transfers for the top five receiving patient organisations**

**Patient organisations**	**Number of funding companies**	**Top funder**	**Overall funding**	**Highest transfer**	**Share highest transfer/ overall funding**	**Top funder interest**
**Rare**						
Cystic Fibrosis Trust	1	Chiesi	£ 1,305,512	£ 1,305,512	100%	*Definitely yes*
Myeloma UK	8	Celgene	£ 1,243,519	£ 425,495	34%	*Definitely yes*
Genetic Alliance UK	15	Alexion	£ 613,006	£ 153,002	25%	*Definitely yes*
International Patient Organisation for Primary Immunodeficiencies	5	Shire	£ 556,357	£ 222,100	40%	*Definitely yes*
Society for Mucopolysaccharide Diseases	6	Sanofi	£ 651,097	£ 293,095	45%	*Definitely yes*
**Non-rare**						
Diabetes UK	9	Novo Nordisk	£ 2,389,423	£ 1,071,507	45%	*Definitely yes*
Epilepsy Society	2	UCB	£ 1,539,749	£ 1,534,236	100%	*Definitely yes*
Shift.MS	5	Sanofi	£ 1,315,328	£ 341,019	26%	*Definitely yes*
Multiple Sclerosis International Federation	6	Sanofi	£ 1,279,214	£ 482,082	38%	*Definitely yes*
Asthma + Lung UK	11	Seqirus	£ 994,842	£ 160,369	16%	*Definitely yes*

For peer review only

Industry funding concentration

On average, each patient organisation received transfers from approximately two companies, with 1.97 (SD:1.74) and 2.21 (SD:1.91) companies funding patient organisations targeting rare and non-rare diseases, respectively. However this difference was not statistically significant ($\chi^2 = 0.197$, *p-value* = 0.657).

In our sample, the median transfer of a company to a patient organisation comprised 33% of the overall industry transfers per organisation (IQR: 0.112-1). When looking at patient organisations focusing on rare diseases, the median company contribution was as high as 42% (IQR: 0.145-1) versus 31% (IQR: 0.116-0.997) for non-rare conditions ($\chi^2 = 7.141$, *p-value* = 0.008).

Finally, the share of industry funding comprised by the single highest transfer per organisation amounted to an average of 73% (SD: 0.29) for the entire sample, ranging from a minimum of 10% to a maximum of 100%. This percentage slightly decreased annually over the study period. The highest value transfer in the case of patient organisations targeting rare diseases made up a larger share of the overall industry funding (median: 86%, IQR: 0.527-1), despite not significant, compared to those focusing on more prevalent conditions (median: 79%, IQR: 0.428-1).

For peer review only

Discussion

In this study, we evaluated the financial links between the pharmaceutical industry and patient organisations in the UK between 2018 and 2020. This is the first study to document the almost-perfect concordance of pharmaceutical company interests and patient organisation funding. Almost all industry transfers during our study period – in terms of both volume (96%) and value (97%) – were to patient organisations aligned with pharmaceutical companies' portfolios and pipelines. Approximately a quarter of industry funding to patient organisations from 2018 to 2020 was directed towards organisations focusing on rare diseases (£9.8 million / £42 million). Finally, we found that patient organisations targeting rare diseases relied on transfers from fewer companies but of higher value compared to organisations focusing on non-rare diseases.

The almost-perfect concordance between industry interests and patient organisation activities likely reflect the commercial attractiveness of conditions targeted by pharmaceutical companies.[28-30] Such close alignment between the interests between companies and patient organisations might undermine the credibility of patient organisations as perceived by the general public and might raise questions about patient organisations' inputs in regulatory and health technology appraisals. A recent study found that during NICE appraisal meetings fewer than 25% of all relevant financial ties between patient organisations and pharmaceutical companies were disclosed.[31]

Our findings make an important contribution to the existing body of literature on industry funding of patient organisations. Ozieranski et al found that industry donated over £57 million to UK patient organisations from 2012 to 2016, an average of £11.5 million per year.[7] The authors also observed that payments were concentrated in commercially attractive therapeutic areas, with organisations focusing on cancer receiving more than 36% of overall payments.[7] However, the study did not examine whether companies were more likely to fund organisations that target diseases for which they have already developed or are currently developing products. Another earlier study examined transfers to Swedish patient organisations and found an association between drug commercialisation and industry funding.[9] The authors did not take into account products in the companies' pipelines nor drugs that might had not yet launched in Sweden. Considering that patient organisations have an important role not only in the post-commercialisation phase but also in the R&D and approval stages, this might have led to an underestimate of the companies' interest in some conditions. We therefore developed a robust, hierarchical matching algorithm to determine whether transfers from companies were directed at organisations that were aligned with their portfolios and pipelines.

Patient organisations focusing on rare diseases can drive both supply of and demand for medicinal products due to their research, advocacy and education role. [2 17] As a result of their close ties with patients, these organisations have the credibility and power to educate patient communities, advocate for access to available therapies and raise awareness on the unmet need of certain conditions.[2 13 32] Although a large share of both the value and number of transfers were directed to patient organisations focusing on rare diseases, most funds targeted commercially attractive rare conditions, such as multiple myeloma and cystic fibrosis, where the unmet need is relatively low compared to other rare conditions. These are diseases that have

For peer review only - http://bmjopen.bmj.com/site/about/guidelines.xhtml

relatively high prevalence and for which 10 and 29 treatments, respectively, are currently approved for use in Europe.[24 33] This poses the risk of widening already existing health inequities, where severe and debilitating rare conditions that affect a small number of patients do not receive the resources they need and have to rely on limited public grants.[34]

Finally, our analysis showed that patient organisations focusing on rare diseases are funded by very few companies, relying on a single transfer for over 80% of their industry-reported income. Despite the share of industry contributions among the overall patient organisation's income remains unknown, this increases the risk of pursuing the company's commercial interests rather than objectively representing a patient body.[11]

These findings have important implications for policy and practice. To minimise conflicts of interests, patient organisations should not accept payments from companies whose products they have endorsed a year before and after this endorsement.[31] One way of avoiding potential conflicts of interest is through increased transparency. Despite considerable progress on this front, especially in terms of reporting the monetary value of industry payments, there are still gaps in reporting.[35] Furthermore, financial independence of patient organisation is fundamental for making sure that patients' interest is at the forefront of the organisations' agenda. In the long term, policymakers should make sure that patient organisations receive adequate public funding regardless of whether they focus on conditions that are profitable for the industry. Such public funding is particularly important for patient organisations supporting rare diseases, as relatively few companies have financial links with patient organisations focusing on rare diseases, potentially creating high reliance on few high-value transfers.

This study had limitations. First, companies may have underreported their financial transfers to patient organisations.[36] However, as underreporting is expected to affect all patient organisations equally, we do not expect this to affect the difference across disease areas or between rare and non-rare diseases investigated in our analysis. Second, in our assessment of company interests, we made a conversative assumption that only patient organisations which target relatively narrow conditions were eligible to be coded as *definitely ye*s. Despite this assumption, we concluded that more than half of transfers were in therapeutic areas in which companies had a clear interest. Finally, our analysis focused on a recent time period (2018-2020). While previous publications show similar trends,[7 9] conferring robustness to the findings, whether these trends hold over time and their generalisability to other periods is unclear.

There are several avenues which can be explored further to build on this analysis. While some of the previous literature on the topic has focused on the financial dependency of patient organisations' budgets from pharmaceutical funding,[10] whether this differs depending on the rarity of the disease targeted has not been explored. Due to the small number of patients affected by rare conditions, patient organisations that target such conditions may be less well-equipped to finance their activities via charitable events and may rely more heavily on contributions from pharmaceutical companies. Lastly, while our analysis did not evaluate the effect of Covid-19 on the financial dynamics between pharmaceutical companies and patient organisations, we expect that the pandemic had a substantial effect on the type, value and

For peer review only - http://bmjopen.bmj.com/site/about/guidelines.xhtml

distribution of transfers. Future research should examine the impact of Covid-19 on industry funding of patient organisations.

Conclusions

Almost all industry funding of patient organisations between 2018 and 2020 was in areas that were aligned with companies' approved drug portfolios and research and development pipelines. Pharmaceutical companies spent a larger amount on patient organisations focusing on rare diseases and that such organisations relied on a small of companies for their funding.

For peer review only

For peer review only - http://bmjopen.bmj.com/site/about/guidelines.xhtml

Acknowledgements: We thank Dr Huseyin Naci and Dr Olivier Wouters, both from the Department of Health Policy at the London School of Economics and Political Science, for providing comments on earlier drafts of this paper. We also acknowledge Dr Mylene Lagarde and Dr Panos Kanavos for the support provided when finalising the manuscript.

Contributors: AG developed a preliminary version of the study and developed it further with IP. AG collected the data. AG and IP did the analysis, wrote and reviewed the manuscript. Both authors had full access to all of the data (including statistical reports and tables) in the study and can take responsibility for the integrity of the data and the accuracy of the data analysis. The corresponding author attests that all listed authors meet authorship criteria and that no others meeting the criteria have been omitted. AG is the guarantor.

Funding: This research received no specific grant from any funding agency in the public, commercial or not-for-profit sectors. AG is funded by the ESRC for her doctoral research (Project reference: 2480077) and is employed by Imperial College London. IP is funded by the ESRC for her doctoral research (Project reference: 2311285) and is employed by Imperial College London.

Competing interest statement: All authors have completed the Unified Competing Interest form (available on request from the corresponding author) and declare: no support from any organisation for the submitted work; no financial relationships with any organisations that might have an interest in the submitted work in the previous three years, no other relationships or activities that could appear to have influenced the submitted work.

Ethical approval: This study does not involve human participants and ethical approval was not required.

Data sharing: A dataset of all publicly available data used in the study is available from the corresponding author at a.gentilini@lse.ac.uk.

Transparency declaration: The lead author affirms that the manuscript is an honest, accurate, and transparent account of the study being reported; that no important aspects of the study have been omitted; and that any discrepancies from the study as planned (and, if relevant, registered) have been explained.

The Corresponding Author has the right to grant on behalf of all authors and does grant on behalf of all authors, an exclusive licence (or non-exclusive for government employees) on a worldwide basis to the BMJ Publishing Group Ltd to permit this article (if accepted) to be published in BMJ editions and any other BMJPGL products and sublicences such use and exploit all subsidiary rights, as set out in our licence.

For peer review only

For peer review only - http://bmjopen.bmj.com/site/about/guidelines.xhtml

References

1. Polich GR. Rare disease patient groups as clinical researchers. *Drug Discovery Today* 2012;17(3):167-72. doi: https://doi.org/10.1016/j.drudis.2011.09.020
2. Geissler J, Ryll B, di Priolo SL, et al. Improving Patient Involvement in Medicines Research and Development::A Practical Roadmap. *Therapeutic Innovation & Regulatory Science* 2017;51(5):612-19. doi: 10.1177/2168479017706405
3. MHRA. Patient Involvement Strategy 2021-25: Medicines and Healthcare products Regulatory Agency 2020.
4. MHRA. Putting patients first: A new era for our agency. Delivery Plan 2021-2023: Medicines and Healthcare products Regulatory Agency 2020.
5. NICE. Public Involvement Programme - Overview of technology appraisals: A factsheet for patient and carer organisations: National Institute for Health and Care Excellence, 2014
6. Fabbri A, Parker L, Colombo C, et al. Industry funding of patient and health consumer organisations: systematic review with meta-analysis. *BMJ* 2020;368:l6925. doi: 10.1136/bmj.l6925
7. Ozieranski P, Rickard E, Mulinari, Shai. Exposing drug industry funding of UK patient organisations. *BMJ* 2019;365:l1806. doi: 10.1136/bmj.l1806
8. Rose SL, Highland J, Karafa MT, et al. Patient Advocacy Organizations, Industry Funding, and Conflicts of Interest. *JAMA Intern Med* 2017;177(3):344-50. doi: 10.1001/jamainternmed.2016.8443
9. Mulinari S, Vilhelmsson A, Rickard E, et al. Five years of pharmaceutical industry funding of patient organisations in Sweden: Cross-sectional study of companies, patient organisations and drugs. *PLoS One* 2020;15(6):e0235021. doi: 10.1371/journal.pone.0235021 [published Online First: 20200624]
10. Ozieranski P, Pitter JG, Rickard E, et al. A 'patient-industry complex'? Investigating the financial dependency of UK patient organisations on drug company funding. *Sociol Health Illn* 2022;44(1):188-210. doi: 10.1111/1467-9566.13409 [published Online First: 20211207]
11. Rose SL. Patient advocacy organizations: institutional conflicts of interest, trust, and trustworthiness. 2014(1748-720X (Electronic))
12. Halley MC. From "Ought" to "Is": Surfacing Values in Patient and Family Advocacy in Rare Diseases. 2021(1536-0075 (Electronic))
13. Aymé S, Kole A, Groft S. Empowerment of patients: lessons from the rare diseases community. *Lancet* 2008;371(9629):2048-51. doi: 10.1016/s0140-6736(08)60875-2
14. Dunkle M, Pines W, Saltonstall PL. Advocacy Groups and Their Role in Rare Diseases Research. In: Posada de la Paz M, Groft SC, eds. Rare Diseases Epidemiology. Dordrecht: Springer Netherlands 2010:515-25.
15. Gentic Alliance. NICE and patient involvement in Highly Specialised Technologies 2018 [Available from: https://geneticalliance.org.uk/wp-content/uploads/2019/01/How-to-work-with-NICE_webinar.pdf.
16. European Commission. Regulation (EC) No 141/2000 of the European Parliament and of the Council of 16 December 1999 on Orphan Medicinal Products, 2000.
17. Mavris M, Le Cam Y. Involvement of patient organisations in research and development of orphan drugs for rare diseases in europe. 2012(1661-8769 (Print))
18. Disclosure UK. ABPI Patient Organisations database 2021 [Available from: https://search.disclosureuk.org.uk/.
19. PMCPA. ABPI Code of Practice 2021 [Available from: https://www.pmcpa.org.uk/the-code/2021-interactive-abpi-code-of-practice/.
20. ONS. Consumer price inflation time series: Office for National Statistics; 2022 [Available from: https://www.ons.gov.uk/economy/inflationandpriceindices/timeseries/l55o/mm23.
21. HMRC. HMRC yearly average and spot rates: HM Revenue and Customs; [Available from: https://www.gov.uk/government/publications/exchange-rates-for-customs-and-vat-yearly.
22. HMRC. HMRC yearly average and spot rates: HM Revenue and Customs; 2022 [Available from: https://www.gov.uk/government/publications/exchange-rates-for-customs-and-vat-yearly.

For peer review only - http://bmjopen.bmj.com/site/about/guidelines.xhtml

23. NIH U.S. National Library of Medicine. ClinicalTrials.gov [Available from: https://clinicaltrials.gov/2022.
24. Orphanet. The portal for rare diseases and orphan drugs 2022 [Available from: https://www.orpha.net/consor/cgi-bin/Disease_Search_Simple.php?lng=EN.
25. European Medicines Agency. Criteria to be fulfilled by patient, consumer and healthcare professional organisations involved in European Medicines Agency (EMA) activities 2018 [Available from: https://www.ema.europa.eu/en/documents/regulatory-procedural-guideline/criteria-be-fulfilled-patient-consumer-healthcare-professional-organisations-involved-european_en.pdf.
26. NICE. Policy on declaring and managing interests for NICE advisory committees, 2018.
27. WHO. ICD-11 for Mortality and Morbidity Statistics 2022 [Available from: https://icd.who.int/browse11/l-m/en#/http://id.who.int/icd/entity/465177735?view=G0.
28. Roos JC, Hyry Hi Fau - Cox TM, Cox TM. Orphan drug pricing may warrant a competition law investigation. (1756-1833 (Electronic))
29. Hughes DA, Poletti-Hughes J. Profitability and Market Value of Orphan Drug Companies: A Retrospective, Propensity-Matched Case-Control Study. 2016(1932-6203 (Electronic))
30. Luzzatto L, Hyry HI, Schieppati A, et al. Outrageous prices of orphan drugs: a call for collaboration. 2018(1474-547X (Electronic))
31. Mandeville KL, Barker R, Packham A, et al. Financial interests of patient organisations contributing to technology assessment at England's National Institute for Health and Care Excellence: policy review. *BMJ* 2019;364:k5300. doi: 10.1136/bmj.k5300
32. Bedlington N, Geissler J, Houyez F, et al. Role of Patient Organisations. In: Facey KM, Ploug Hansen H, Single ANV, eds. Patient Involvement in Health Technology Assessment. Singapore: Springer Singapore 2017:401-10.
33. European Medicines Agency. European public assessment reports (EPAR), 2022.
34. Baggott R, Jones K. The Big Society in an age of austerity: threats and opportunities for Health Consumer and Patients' Organizations in England. 2015(1369-7625 (Electronic))
35. Lexchin J. Association between commercial funding of Canadian patient groups and their views about funding of medicines: An observational study. *PLOS ONE* 2019;14(2):e0212399. doi: 10.1371/journal.pone.0212399
36. Ozieranski P, Csanádi M, Rickard E, et al. Under-reported relationship: a comparative study of pharmaceutical industry and patient organisation payment disclosures in the UK (2012–2016). *BMJ Open* 2020;10(9):e037351. doi: 10.1136/bmjopen-2020-037351

For peer review only

For peer review only - http://bmjopen.bmj.com/site/about/guidelines.xhtml

Figure legend

Figure 1. Hierarchical algorithm to determine company interests in patient organisation funding

Note: An interest is when there is, or could be perceived to be, an opportunity for a pharmaceutical company to benefit in the disease area where the patient organisation operates.

Figure 2. Cumulative value of transfers by receiving patient organisation and funding company from 2018-2020

Figure 3. Cumulative value of transfers by patient organisation type and therapeutic area from 2018-2020

For peer review only

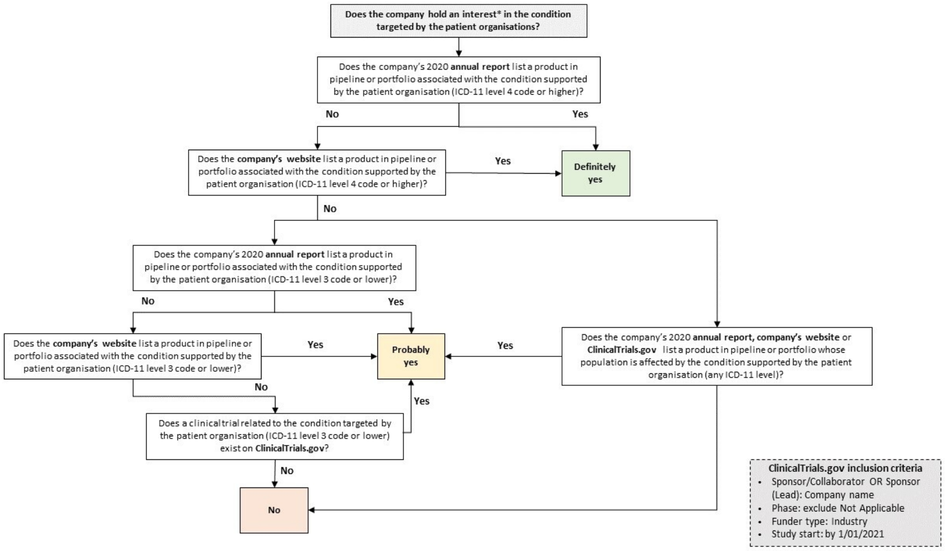

figure 1

108x60mm (300 x 300 DPI)

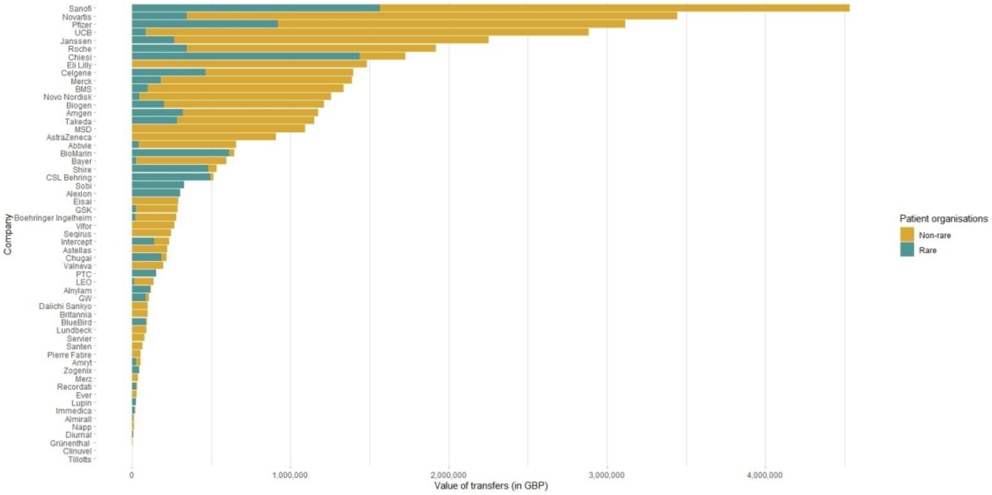

figure 2

130x64mm (300 x 300 DPI)

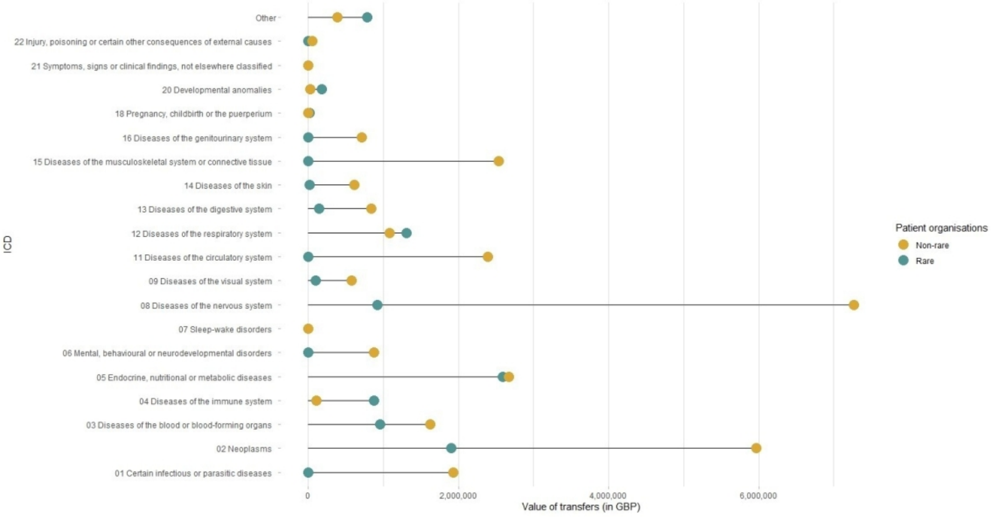

figure 3

124x64mm (300 x 300 DPI)

Supplemental Material

Data collection

Transfers of value

Data on transfers from pharmaceutical companies to POs from 2018 to 2020 were retrieved in January 2022 from the Disclosure UK patient organisation gateway.1 The gateway was launched in 2020 and is a collection of hyperlinks to companies' disclosure of transfers to patient organisations. Disclosing financial transfers to patient organisations is a requirement of Clause 29 of the Association of British Pharmaceutical Industry (ABPI) Code of Practice.2 However, companies signed up to abide by the ABPI Code, accepting the jurisdiction of the Prescription Medicines Code of Practice Authority (code regulator) extends beyond those who are ABPI members and is expected to include most pharmaceutical companies operative in the UK. The websites of all pharmaceutical companies appearing in the Disclosure UK database were screened to ensure all transfers were captured. If transfers were not disclosed in Disclosure UK nor in the company's website,
transfer to patient organisations in that year(s).

AG extracted transfer disclosures from companies' websites, comprising of the name of the patient organisation, the year in which the transfer was made, the reason why it was made and its value. Given that a consolidated database of transfers was not available and transfers needed to be manually compiled from each individual
of transfers to validate the data collection process and minimise the risk of reporting errors.

All transfers were first adjusted for inflation using the ONS Consumer Price Index [3] and then converted to British Pounds (GBP), using the ONS historical yearly conversion rates.[4 5] All transfers are in 2020 GBP. Data on pharmaceutical companies' portfolio and pi
retrieved from their latest annual report, company website and ClinicalTrials.gov,[6] in order of screening.

Therapeutic areas

Patient organisations' websites were also sc
on. For example, in the case of *Blood Cancer UK*, their mission is to “*beat blood cancer*”,
therefore, the condition supported was coded as blood cancer.

After being identified as described above, conditions were further classified into rare and non-rare.

Conditions were considered rare if they appeared in the Orphanet database of rare diseases regardless of their classification level (group of disorders, disorders or subtypes of disorders).[9] For example, multiple myeloma appears in the Orphanet database of rare diseases, therefore a patient organisation focusing this condition would be categorised as rare-focused. When condition sub-types appeared in the Orphanet databas
screened to check whether its focus was on rare conditions. For example, *Metabolic Support UK's* motto is “*Your rare condition. Our common fight”* and was therefore assumed to be rare

disease-focused. Conversely, should a patient organisation focus on a broader condition such as blood cancer with no sole focus on rare conditions, the organisation would be conservatively considered non-rare. While this approach was preferred as it did not require further assumptions, it entails that only more specialised patient organisation are considered as rare. Such approach might have led to the number and overall value of transfers from pharmaceutical companies to rare diseases-focused patient organisations being underestimated, as these organisaitons are expected to get less transfers than more generalist ones (e.g. multiple myeloma vs blood cancer).

A third category (*unclear)* was created for non-disease-specific patient organisations, such as coalition of charities or organisations focused on palliative care for terminally ill patients. This category was excluded from the main analyses, but sub-group analyses are reported at the end of the Supplemental Material.

Companies' interest

We developed a methodology to assess the extent to which a pharmaceutical company holds an interest in the disease supported by a patient organisation. For the purpose of this analysis, we adapted the definition of interest provided by NICE.[11] An interest is when there is, or could be perceived to be, an opportunity for a pharmaceutical company to benefit in the disease area where the patient organisation operates. This could include situations where the pharmaceutical company has a drug developed or in development for a condition supported by the patient organisation, or where a drug in the company [illegible]
population affected by the disease supported by the patient organisation.

As first step, the conditions supported by patient organisations were translated into ICD-11 codes using the online ICD-11 database.[12]

ICD-11 codes are mutually exclusive, exhaustive and are arranged as a single hierarchical tree. This means that specific diseases are nested within broader classifications. An example for multiple myeloma is shown in Table 1 below.

Table 1. Example of ICD-11 classification, Multiple myeloma

Hierarchy level	Condition	ICD-11 code
Level 1	Neoplasms	2
Level 2	Neoplasms of haematopoietic or lymphoid tissues	2A
Level 3	Mature B-cell neoplasms	2A8
Level 4	Plasma cell neoplasms	2A83
Level 5	Plasma cell myeloma	2A83.1

In this example, multiple myeloma is nested within *Plasma cell myeloma*, who is in its turn nested within *Plasma cell neoplasms* and so on up to *Neoplasms*.

Subsequently, companies' annual reports, we[illegible]
searched to assess whether the each company had an interest in the condition supported by the patient organisation receiving the transfer. The diagram in the main document (Figure 1)

For peer review only

schematically illustrates the approach taken to understand whether the company definitely, maybe or did not have an interest in the condition.

For example, if *Company X* reports in its annual report having a drug in development for multiple myeloma and transferred a sum of money to *Blood Cancer UK*, this would be coded as *maybe yes*, as the company has a product in its pipeline or portfolio associated with a condition supported by the patient organisation. In this case, the ICD-11 level would be 2, *Neoplasms of haematopoietic or lymphoid tissue*, under which multiple myeloma is nested. Conversely, should *Company X* have made a transfer to *Myeloma UK*, this would have been coded as *definitely yes*, as there is perfect alignment between the condition supported by the patient organisation and by *Company X's* drug.

Situations where a company's interest in a c [illegible] impossibility of identifying such link, rather than the lack thereof.

For peer review only

Inclusion/exclusion of patient organisations

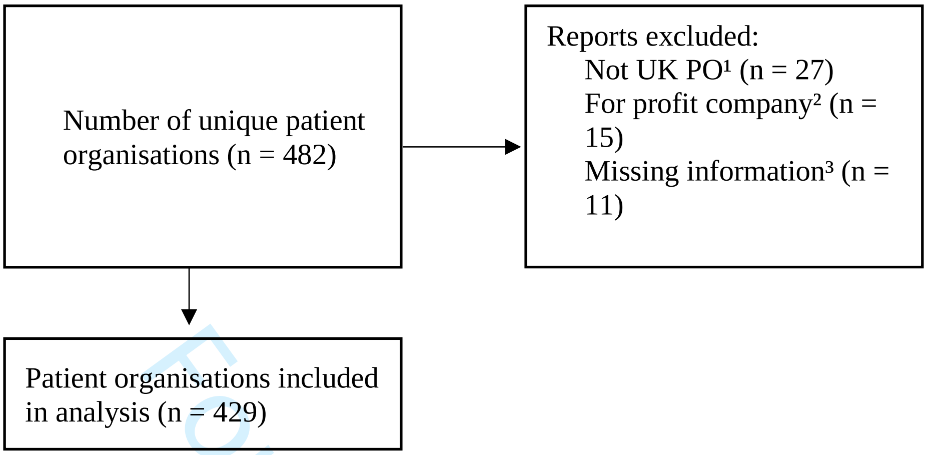

[1]Not aligned with geographical scope e.g. Irish, US-based
[2]Not aligned with EFPIA's definition of patient organisation
[3]Organisations for whose nature is unclear i.e. patient organisation website could not be identified

For peer review only

Additional tables and figures

Table 2. Number and value of transfers from the pharmaceutical industry to UK patient organisations broken down rarity of diseases from 2018 to 2020

	Rare-focused patient organisaitons	Non-rare-focused patient organisaitons	Overall
**Number of TOVs**	636	2,324	2,960
**Mean TOV**	£15,395	£12,767	£13,331
**Median TOV**	£7,000	£5,085	£5,136
**Max. TOV**	£440,229	£946,300	£946,300
**Min. TOV**	£17	£7	£7
**SD**	£35,478	£31,654	£32,525
**TOVs 2018**	£2,329,017	£7,991,072	£10,320,089
**TOVs 2019**	£3,281,001	£9,109,462	£12,390,463
**TOVs 2020**	£4,180,892	£12,570,028	£16,750,919
**Overall TOVs**	£9,790,909	£29,670,562	£39,461,472

Abbreviations: SD (standard deviation); TOV (transfer of value).

For peer review only

Figure 1 Value of transfers by receiving patient organisation and funding company, broken down by year

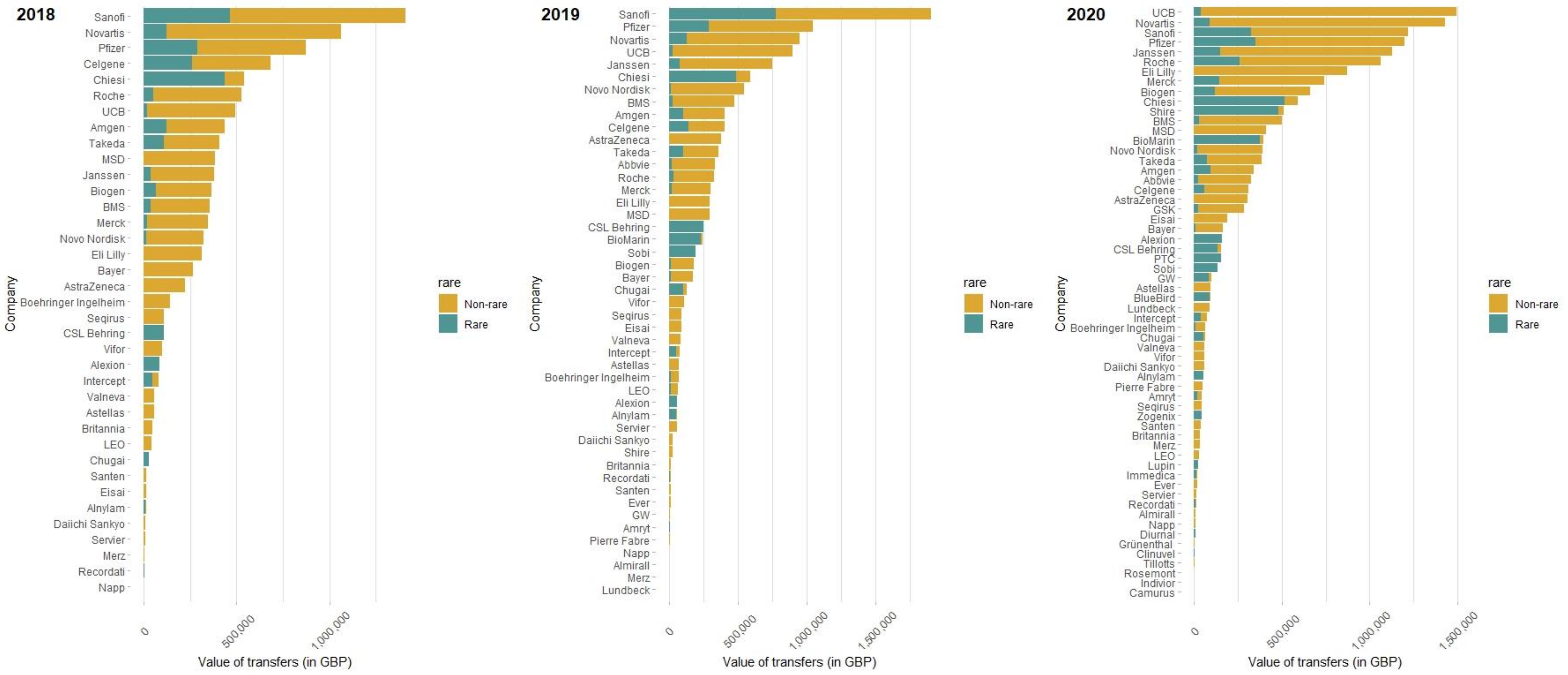

Figure 2. Value of transfers by patient organisation type, therapeutic area and year

A)

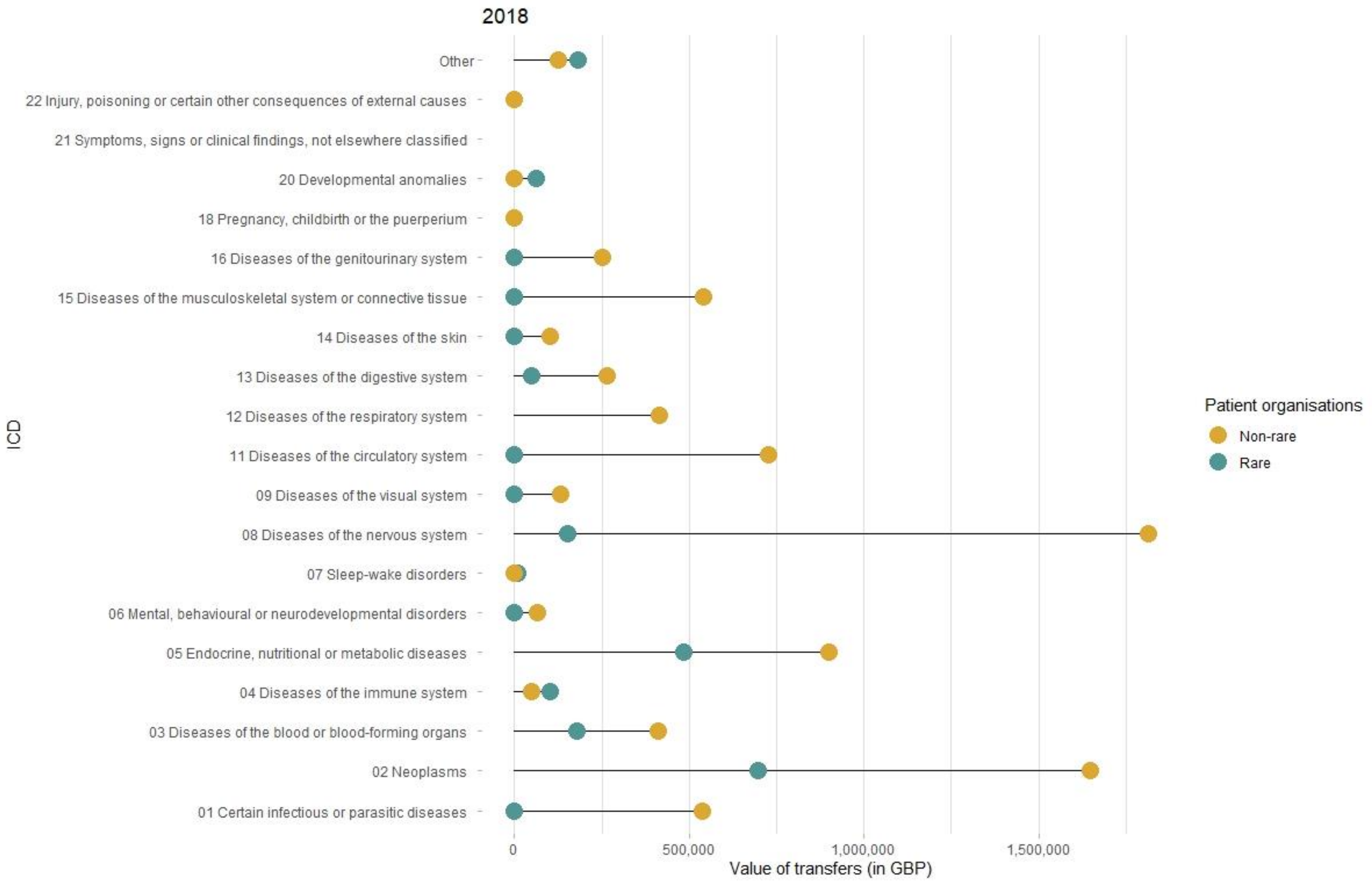

B)

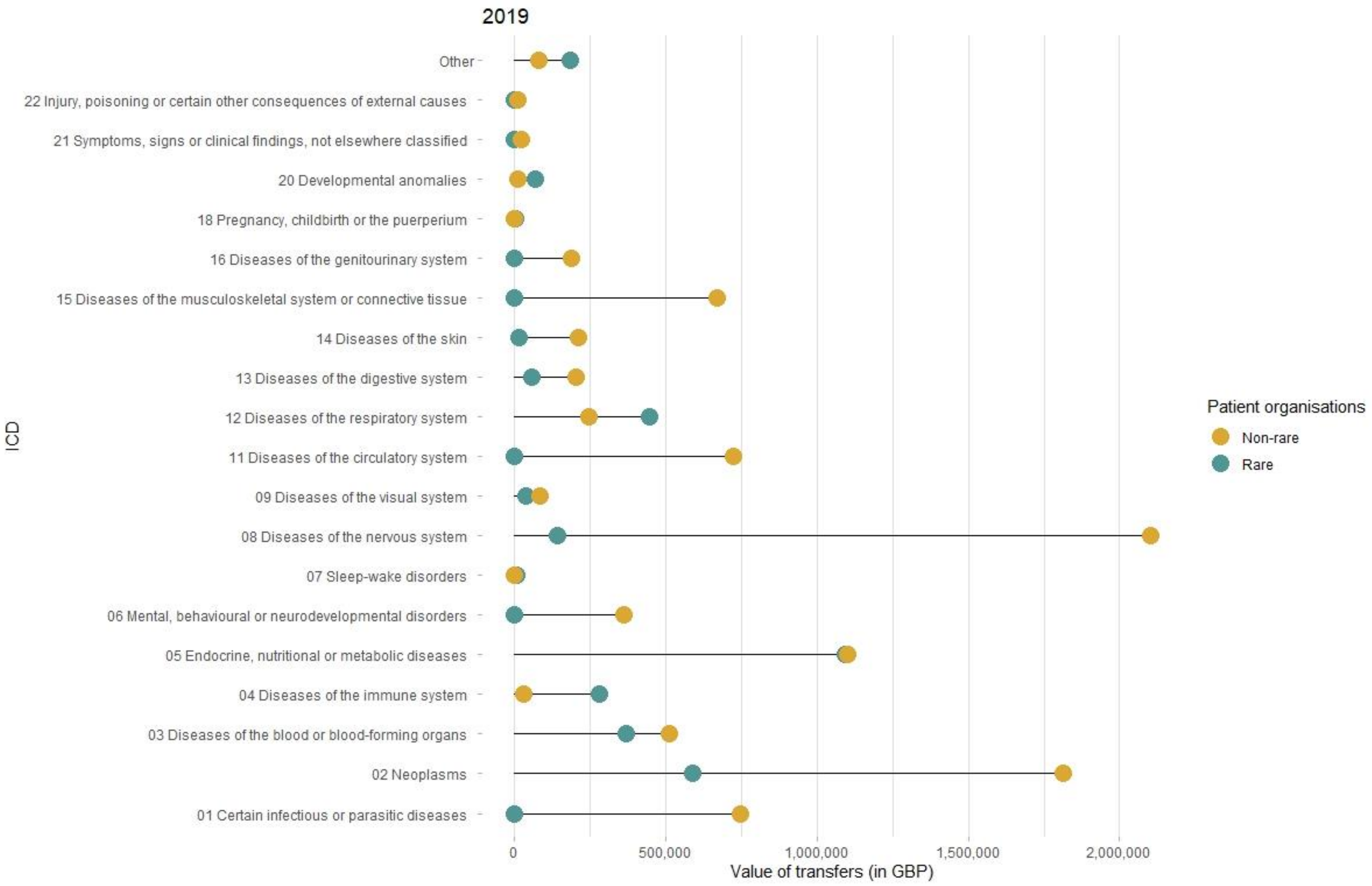
2019
Other
22 Injury, poisoning or certain other consequences of external causes
21 Symptoms, signs or clinical findings, not elsewhere classified
20 Developmental anomalies
18 Pregnancy, childbirth or the puerperium
16 Diseases of the genitourinary system
15 Diseases of the musculoskeletal system or connective tissue
14 Diseases of the skin
13 Diseases of the digestive system
12 Diseases of the respiratory system
11 Diseases of the circulatory system
09 Diseases of the visual system
08 Diseases of the nervous system
07 Sleep-wake disorders
06 Mental, behavioural or neurodevelopmental disorders
05 Endocrine, nutritional or metabolic diseases
04 Diseases of the immune system
03 Diseases of the blood or blood-forming organs
02 Neoplasms
01 Certain infectious or parasitic diseases
ICD
0
500,000
1,000,000
1,500,000
2,000,000
Value of transfers (in GBP)
Patient organisations
Non-rare
Rare

C)

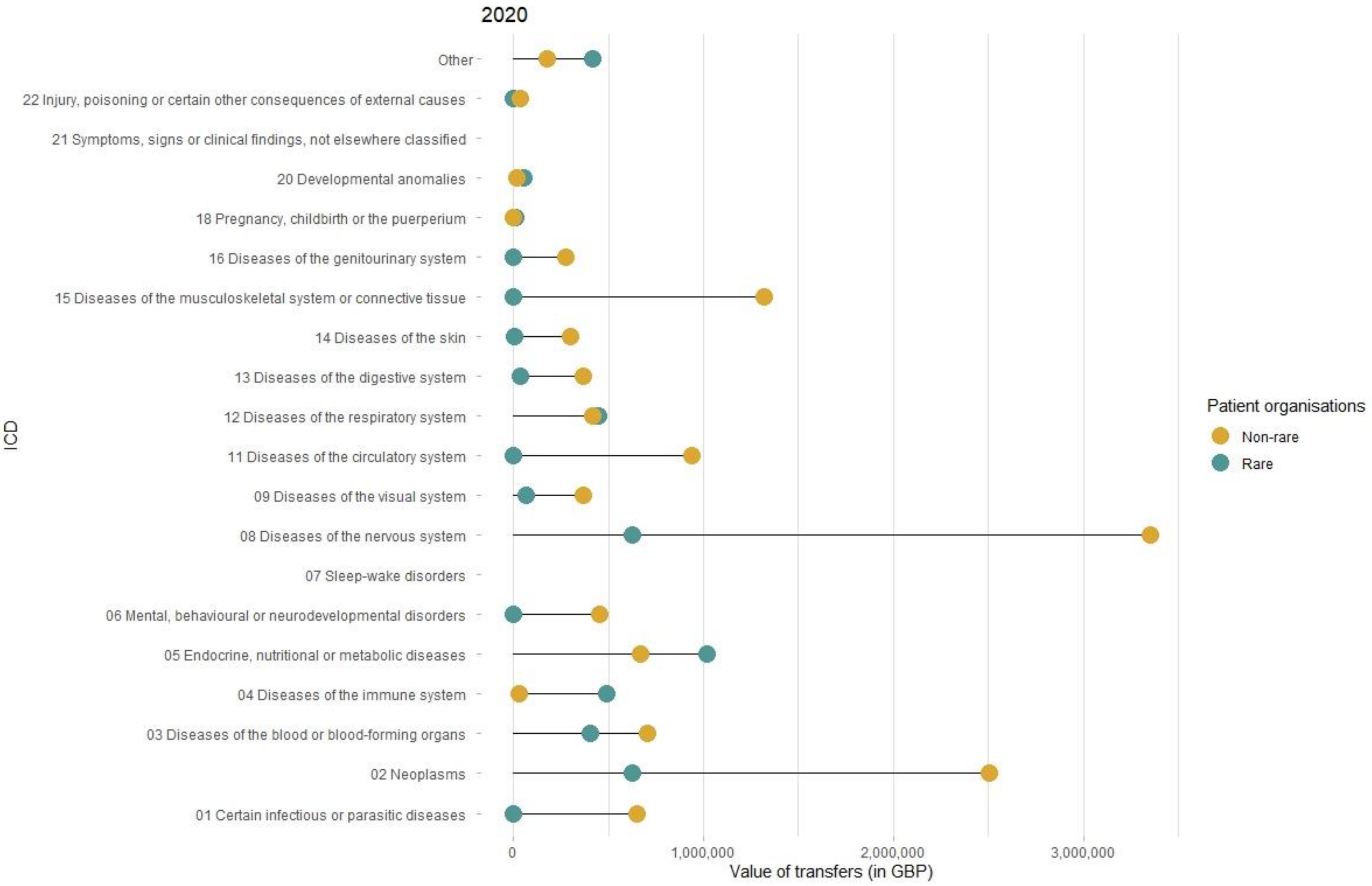
2020
Other
22 Injury, poisoning or certain other consequences of external causes
21 Symptoms, signs or clinical findings, not elsewhere classified
20 Developmental anomalies
18 Pregnancy, childbirth or the puerperium
16 Diseases of the genitourinary system
15 Diseases of the musculoskeletal system or connective tissue
14 Diseases of the skin
13 Diseases of the digestive system
12 Diseases of the respiratory system
11 Diseases of the circulatory system
09 Diseases of the visual system
08 Diseases of the nervous system
07 Sleep-wake disorders
06 Mental, behavioural or neurodevelopmental disorders
05 Endocrine, nutritional or metabolic diseases
04 Diseases of the immune system
03 Diseases of the blood or blood-forming organs
02 Neoplasms
01 Certain infectious or parasitic diseases
ICD
0
1,000,000
2,000,000
3,000,000
Value of transfers (in GBP)
Patient organisations
Non-rare
Rare

Sub-group analyses

Excluded patient organisations

181 transfers made 53 to patient organisaitons were excluded from the analysis as they did not match EFPIA's *"not-for-profit organisation, mainly composed of patients and/or caregivers, that represent and/or support the needs of patients and/or caregivers"*.

Figure 3 illustrates the reasons for patient organisations exclusion. Most of the excluded patient organisations were not UK-based (56%; n=101), followed by for profit organisations (36%; n=66) and organisations for which no information could be found online (8%; n=14).

Non-UK patient organisations mostly comprised international alliances of patient organisations, European or Irish organisations. We classified organisations as for-profit if they appeared in the UK government repository of companies[1] as *private limited companies.* Care homes, consultancies and rehabilitation clinics were the most prominent in this category.

Overall, transfers to excluded patient organisations amounted to £2,279,445, about 5% of the included transfers (Figure 4).

Figure 3. Excluded patient organisations by reason of exclusion

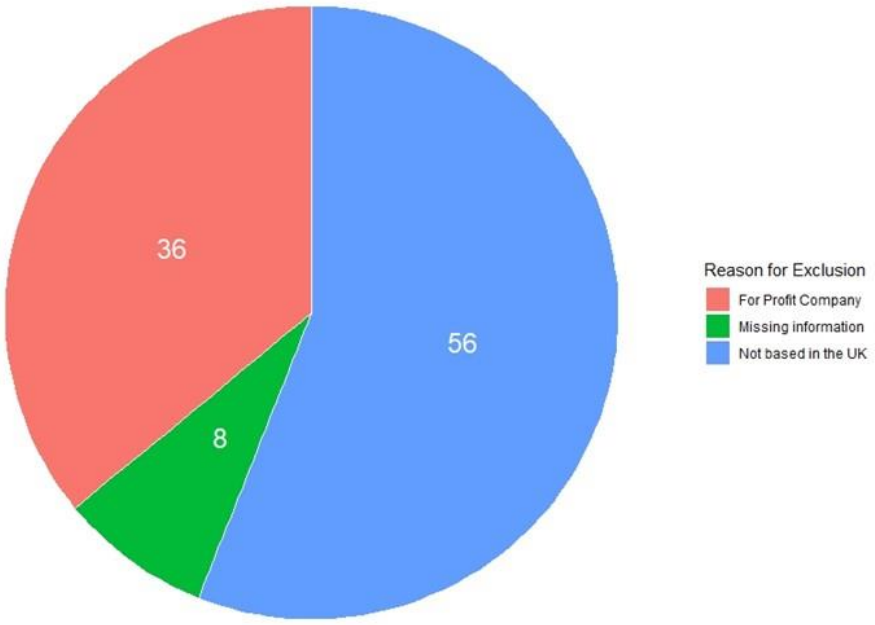

[1] https://find-and-update.company-information.service.gov.uk/

Figure 4. Transfers to included and excluded patient organisations

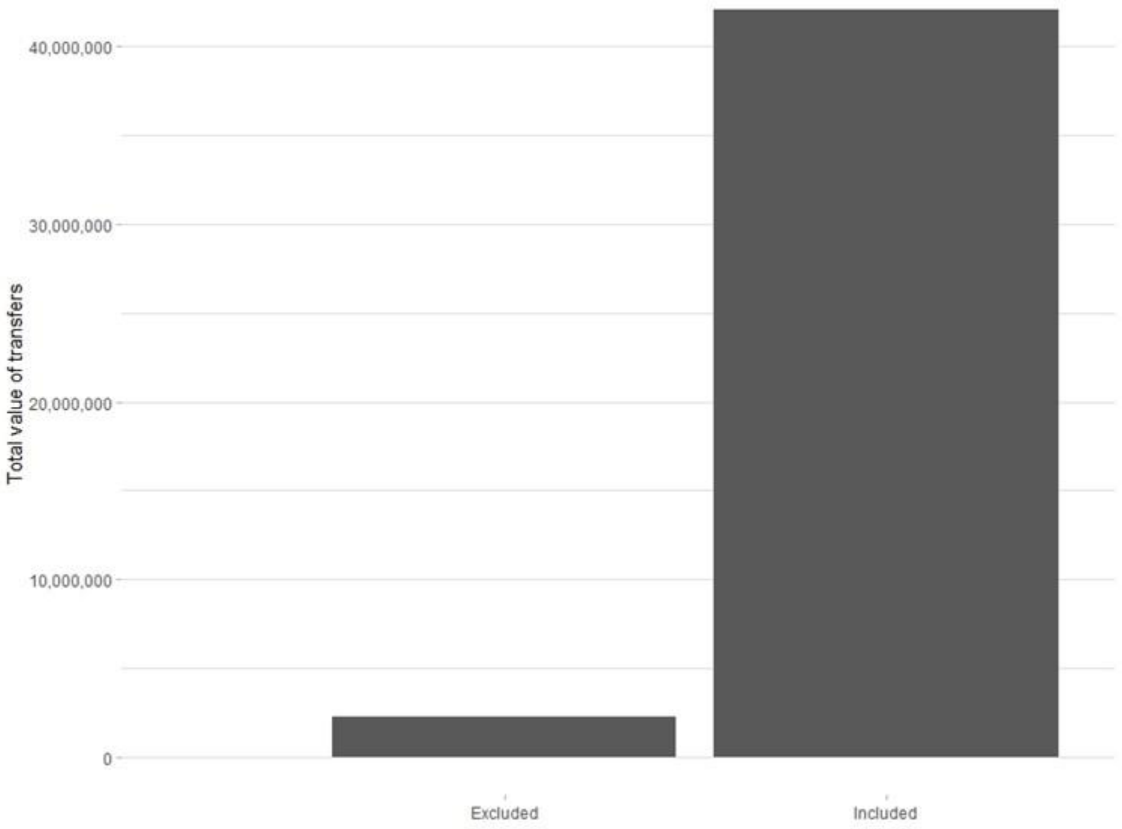

<u>Non-disease-specific organisations</u>

Overall, 378 transfers were made to non-disease-specific organisations. Of those, 181 were excluded due to the recipient organisation not meeting the necessary condition to be classified as a patient organisation (as per the analysis presented above). 197 transfers were made to 63 non-disease-specific patient organisations. These included hospital charities, carers organisations and hospices.

Transfers to non-disease-specific organisations amounted to £ 2,534,044, about 6% of the included disease-specific transfers (Figure 5).

For peer review only - http://bmjopen.bmj.com/site/about/guidelines.xhtml

Figure 5. Transfers to disease and non-disease-specific patient organisations

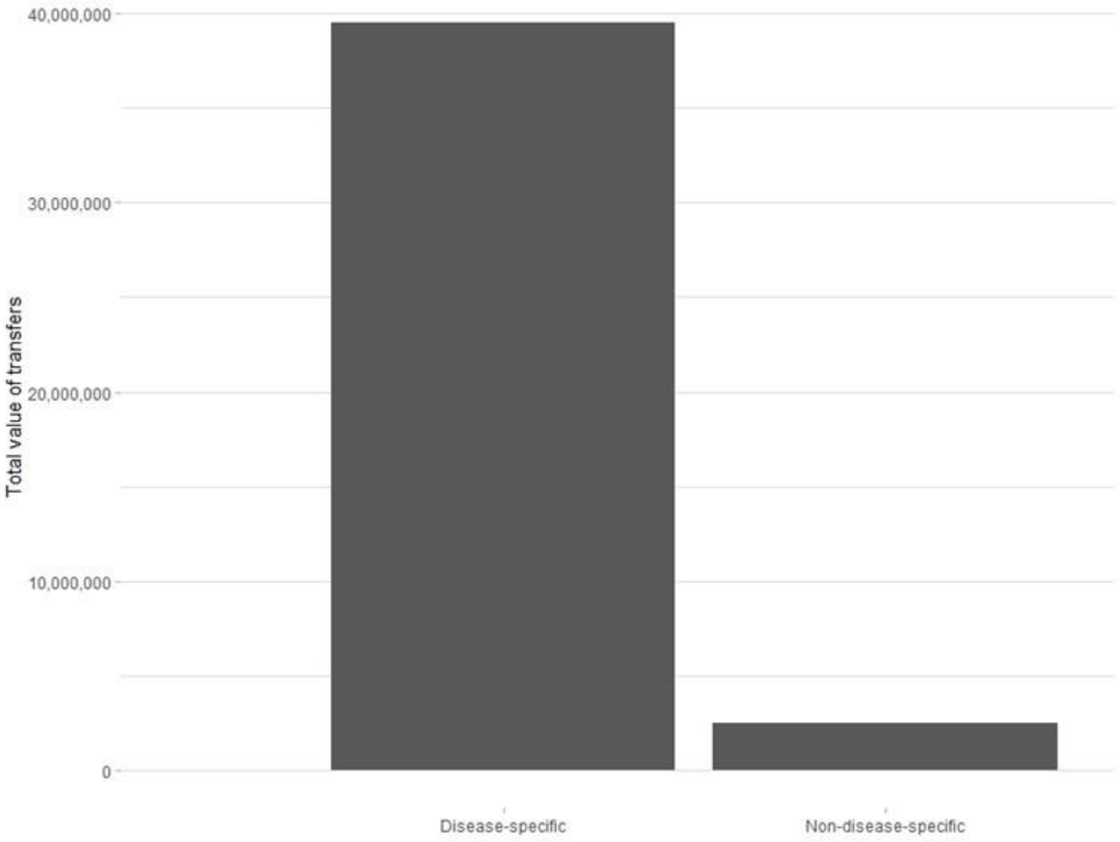

review only

For peer review only - http://bmjopen.bmj.com/site/about/guidelines.xhtml

References

1. Disclosure UK. ABPI Patient Organisations database 2021 [Available from: https://search.disclosureuk.org.uk/.
2. PMCPA. ABPI Code of Practice 2021 [Available from: https://www.pmcpa.org.uk/the-code/2021-interactive-abpi-code-of-practice/.
3. ONS. Consumer price inflation time series: Office for National Statistics; 2022 [Available from: https://www.ons.gov.uk/economy/inflationandpriceindices/timeseries/l55o/mm23.
4. HMRC. HMRC yearly average and spot rates: HM Revenue and Customs; [Available from: https://www.gov.uk/government/publications/exchange-rates-for-customs-and-vat-yearly.
5. HMRC. HMRC yearly average and spot rates: HM Revenue and Customs; 2022 [Available from: https://www.gov.uk/government/publications/exchange-rates-for-customs-and-vat-yearly.
6. NIH U.S. National Library of Medicine. ClinicalTrials.gov [Available from: https://clinicaltrials.gov/2022.
7. EFPIA. EFPIA Code of Practice on the Relationships between the Pharmaceutical Industry and Patient Organisations: European Federation of Pharmaceutical Industries and Associations, 2011.
8. Ozieranski P, Rickard E, Mulinari, Shai. Exposing drug industry funding of UK patient organisations. *BMJ* 2019;365:l1806. doi: 10.1136/bmj.l1806
9. Orphanet. The portal for rare diseases and orphan drugs 2022 [Available from: https://www.orpha.net/consor/cgi-bin/Disease_Search_Simple.php?lng=EN.
10. Mulinari S, Vilhelmsson A, Rickard E, et al. Five years of pharmaceutical industry funding of patient organisations in Sweden: Cross-sectional study of companies, patient organisations and drugs. *PLoS One* 2020;15(6):e0235021. doi: 10.1371/journal.pone.0235021 [published Online First: 20200624]
11. NICE. Policy on declaring and managing interests for NICE advisory committees, 2018.
12. WHO. ICD-11 for Mortality and Morbidity Statistics 2022 [Available from: https://icd.who.int/browse11/l-m/en#/http://id.who.int/icd/entity/465177735?view=G0.

For peer review only - http://bmjopen.bmj.com/site/about/guidelines.xhtml

BMJ Open

Industry funding of patient organisations in the United Kingdom: A retrospective study of commercial determinants, funding concentration and disease prevalence

Journal:	*BMJ Open*
Manuscript ID	bmjopen-2022-071138.R1
Article Type:	Original research
Date Submitted by the Author:	08-Mar-2023
Complete List of Authors:	Gentilini, Arianna; The London School of Economics and Political Science, Department of Health Policy<br>Parvanova, Iva; The London School of Economics and Political Science, Department of Health Policy
**Primary Subject Heading**:	Health policy
Secondary Subject Heading:	Health policy
Keywords:	Health policy < HEALTH SERVICES ADMINISTRATION & MANAGEMENT, PUBLIC HEALTH, Health Equity

SCHOLARONE™ Manuscripts

I, the Submitting Author has the right to grant and does grant on behalf of all authors of the Work (as defined in the below author licence), an exclusive licence and/or a non-exclusive licence for contributions from authors who are: i) UK Crown employees; ii) where BMJ has agreed a CC-BY licence shall apply, and/or iii) in accordance with the terms applicable for US Federal Government officers or employees acting as part of their official duties; on a worldwide, perpetual, irrevocable, royalty-free basis to BMJ Publishing Group Ltd ("BMJ") its licensees and where the relevant Journal is co-owned by BMJ to the co-owners of the Journal, to publish the Work in this journal and any other BMJ products and to exploit all rights, as set out in our licence.

The Submitting Author accepts and understands that any supply made under these terms is made by BMJ to the Submitting Author unless you are acting as an employee on behalf of your employer or a postgraduate student of an affiliated institution which is paying any applicable article publishing charge ("APC") for Open Access articles. Where the Submitting Author wishes to make the Work available on an Open Access basis (and intends to pay the relevant APC), the terms of reuse of such Open Access shall be governed by a Creative Commons licence – details of these licences and which Creative Commons licence will apply to this Work are set out in our licence referred to above.

Other than as permitted in any relevant BMJ Author's Self Archiving Policies, I confirm this Work has not been accepted for publication elsewhere, is not being considered for publication elsewhere and does not duplicate material already published. I confirm all authors consent to publication of this Work and authorise the granting of this licence.

For peer review only

Industry funding of patient organisations in the United Kingdom: A retrospective study of commercial determinants, funding concentration and disease prevalence

Arianna Gentilini and Iva Parvanova

Department of Health Policy, London School of Economics and Political Science, London, UK

Corresponding author

Arianna Gentilini

Department of Health Policy
Cowdray House, COW.4.09
London School of Economics
Houghton street
London WC2A 2AE
England
Email: a.gentilini@lse.ac.uk

For peer review only

Industry funding of patient organisations in the United Kingdom: A retrospective study of commercial determinants, funding concentration and disease prevalence

Abstract

Objectives – To assess the relationship between UK-based patient organisation funding and companies' commercial interests in rare and non-rare diseases from 2018 to 2020.

Design – Retrospective analysis of the value and volume of payments from pharmaceutical companies to patient organisations in the UK matched with data on the conditions supported by patient organisations and drugs in companies' approved portfolios and research and development pipelines.

Setting – UK.

Participants – 60 pharmaceutical companies making payments to 483 UK-based patient organisations.

Main outcome measures – Alignment between the commercial interests of pharmaceutical companies and the disease area focus of patient organisations; difference in the volume and value of payments to patient organisations broken down by prevalence of conditions; industry funding concentration, measured as the number of companies funding each patient organisations, the share of overall industry funding coming from each contributing company and the share of industry funding of each organisation comprised by the single highest payments.

Results – 3,155 payments were made by 60 companies to 429 patient organisations. Almost all funds (92%) from pharmaceutical companies were directed to patient organisations that are aligned with companies' approved drug portfolios and research and development pipelines. Despite rare diseases affecting less than 5% of the UK population, 25% of all payments were directed to patient organisations which target such conditions. Patient organisations focusing on rare diseases relied on payments from fewer companies (*p-value* = 0.008) compared to organisations focusing on non-rare diseases.

Conclusions – Companies predominantly funded patient organisations operating in therapeutic areas relevant to companies' portfolio or drug development pipeline. Patient organisations focusing on rare diseases received more funding relative to the number of patients affected by these conditions and relied more heavily on payments from fewer companies compared to organisations targeting non-rare diseases. Increased independence of patient organisations could help avoiding conflicts of interest.

For peer review only

Strengths and limitations of this study

- We develop a methodology to determine the concordance between commercial interests of pharmaceutical companies and disease areas supported by patient organisations.
- We present a comparative analysis of industry funding to patient organisations depending on the prevalence of the disease(s) they support.
- Our analysis focuses on a recent time period which might differ from historical trends.
- The sample size of pharmaceutical companies making payments to patient organisations was not constant over time. However this is expected to have a limited impact, as payment values were similar for companies that consistently disclosed payments.

For peer review only

Introduction

Patient organisations, which represent and support the needs of patients, play an important role in the development, regulatory review, and adoption of new drugs. They are defined as not-for-profit organisations, mainly composed of patients and/or caregivers that represent and/or support the needs of patients or caregivers.[1 2] During research and development, patient organisations effectively advocate for resources to be directed to conditions where unmet need is highest.[3 4] Patient organisations support research design and planning, helping to identify patient-relevant study endpoints.[4] Patient organisations also represent patient views and preferences at the time of regulatory review and health technology assessment of new drugs.[5 6] For example, during technology appraisals conducted by the National Institute for Health and Care Excellence (NICE), which makes funding recommendations for the English National Health Service (NHS), patients, and organisations representing the interests of patients, provide testimonies of their first-hand experiences on how the disease affects them and those around them.[7] Finally, when drugs are launched, patient organisations contribute to dissemination of research results to patient community and clinicians, and offer support and information on therapies available.[4 8]

Given the role of patient organisations across many stages of drug development, approval and access, it is vital to understand their financial ties with pharmaceutical companies. Previous studies documented the large number and high value of payments from pharmaceutical companies to patient organisations, [2 8-10] the uneven distribution between and within therapeutic areas,[2 10] and the concentration of payments coming from a small number of pharmaceutical firms across multiple jurisdictions.[2 8-15]

What remains unknown is the alignment between the commercial interests of pharmaceutical companies and UK patient organisations' activities. Prior research has demonstrated that industry tends to prioritize commercially attractive conditions, and there is evidence to suggest that the marketing of a drug for a particular disease is associated with increased industry funding to patient organizations operating in that area. [2 10] However, such studies have typically been conducted in different geographic settings and have focused solely on marketed drugs, rather than examining the entire research and development pipeline of pharmaceutical companies. This is especially important given the lengthy timeline for drugs to reach the market,[16] as failure to consider drugs currently undergoing clinical trials may result in an incomplete picture.

Another gap in the literature relates to the dynamics between the pharmaceutical industry and patient organisations supporting rare vs. non-rare conditions.

The fragmented nature of rare diseases, coupled with the lack of interest from policymakers and manufacturers, who often prioritize more profitable and prevalent diseases, has necessitated the formation of patient organizations to advocate for the needs of rare disease patients.[17 18] The National Organization for Rare Disorders (NORD), serves as the umbrella organization for rare disease patients in the United States (US) and has been instrumental in lobbying for scientific support and economic incentives to stimulate innovation in rare diseases.[19] This advocacy ultimately led to the passing of the Orphan Drug Act in 1983 in the USA and the EU Regulation on Orphan Medicinal Products in Europe in 2000.[20 21]

For peer review only - http://bmjopen.bmj.com/site/about/guidelines.xhtml

Moreover, the limited availability and complexity of medical knowledge regarding rare diseases have also fostered patients and families affected by these conditions to come together to provide each other with support and medical expertise.[17 22] Patient organisations, which are primarily composed of patients and their caregivers, are in a unique position to share first-hand experiences that can inform research and regulatory decisions.[23] While this is true also for non-rare conditions, patient organisations' input in regulatory and health technology appraisals is particularly important in the context of rare diseases due to scarce evidence. For example, the Scottish Medicines Consortium (SMC) provides opportunities for patient groups and clinicians to have a stronger voice in the decision-making process for drugs used to treat rare and end-of-life conditions.[24] Similarly, three members of patient organisations sit in the Committee for Orphan Medicinal Products (COMP) within the European Medicines Agency (EMA), the body responsible for granting orphan designations to drugs. Patient organisation-led registries that collect real-world data on disease progression can de-risk drug development for rare diseases.[17] While observational studies are common in non-rare diseases, they usually do not require the support of patient organisations' networks as patients are easier to identify and recruit.[3]

Finally, there has been limited exploration of the concentration of industry funding for patient organizations. A recent study by Mulinari and colleagues (2022) examined the average number of pharmaceutical companies making payments to Danish patient organizations,[15] while only one study has investigated the share of industry funding and the top drug company donor's share in UK patient organizations' income.[11] However, no study has specifically focused on the number of companies funding UK patient organizations, nor have they explored whether organisations' industry funding differs based on disease rarity.

Our paper aims to contribute to and expand on existing literature by examining the concordance between the commercial interests of pharmaceutical companies and patient organizations' activities in the UK. Using publicly available data on payments between 2018 and 2020, we analysed the volume, value of payments to patient organisations according to their disease area of interest, with the objective of examining whether there are differences in funding patterns between rare and non-rare diseases. Lastly, we examined the concentration of industry funding, namely how many companies funded each patient organisation and the extent to which organisations might have been reliant on funding from a single company. Based on the reviewed literature, we formulated the following hypotheses:

- *Hypothesis 1*: With respect to value and volume of industry payments to patient organisations, we expect similar overall funding patterns to those reported in the existing literature – namely an increase over time;[2]
- *Hypothesis 2*: Regarding the concordance between the commercial interests of pharmaceutical companies and patient organisations' activities, we expect no difference between rare and non-rare patient organisations, under the assumption that companies are unlikely to invest in such organisations out of altruistic motives;
- *Hypothesis 3*: Furthermore, we hypothesise that patient organizations targeting rare diseases would receive less overall funding due to their low prevalence;
- *Hypothesis 4*: Considering the limited availability of drugs for rare diseases from a handful of manufacturers, we expect organizations focusing on these conditions to rely

For peer review only - http://bmjopen.bmj.com/site/about/guidelines.xhtml

on payments of higher value and from fewer companies compared to those targeting more prevalent conditions.

For peer review only

For peer review only - http://bmjopen.bmj.com/site/about/guidelines.xhtml

Methods

Data on industry payments

We used the Disclosure UK patient organisation gateway (in January 2022) as well as companies' websites to retrieve data on payments from the pharmaceutical industry to UK patient organisations from 2018 to 2020.[25] The gateway was launched in 2020 and is a collection of hyperlinks to companies' disclosure of payments to patient organisations. Disclosing payments to patient organisations is a requirement of Clause 29 of the Association of British Pharmaceutical Industry (ABPI) Code of Practice.[26] Companies that sign up to abide by the ABPI Code accept the jurisdiction of the Prescription Medicines Code of Practice Authority (PMCPA, code regulator), which extends beyond those who are ABPI members.[26] This requirement therefore affects virtually all pharmaceutical companies operating in the UK. Companies might be sanctioned by the PMCPA if they do not disclose their payments.[26] We screened the websites of all pharmaceutical companies abiding by the ABPI Code, most of which provided a link in the Disclosure UK database, and retrieved payments information companies' websites to ensure all payments were captured. If payments were not disclosed in Disclosure UK nor in the company's website, we assumed the company was did not make any payments to patient organisations in a given year which is commonly assumed in the literature.[2]

One investigator (AG) extracted payment disclosures from the companies' websites. These comprised the name of the patient organisation, the year when the payment was made, the reason for the payment and its value in the currency reported by the disclosing company. All payments were first adjusted for inflation using the ONS Consumer Price Index.[27] When reported in different currencies, such as United States Dollars (USD), Swiss Franc (CHF), Swedish Krona (SEK), Norwegian Krone (NKK) and Danish Krone (DKK), the value of the payment was converted to Great British Pounds (GBP), using the ONS historical yearly conversion rates. [28 29] We reported all payments in 2020 GBP. Two in-kind payments with a monetary value of zero were excluded from the analysis.

Data on patient organisations

We retrieved data on patient organisations from their websites. Details on the therapeutic area they advocated for – proxied by International Classification of Diseases Version 11 (ICD-11) codes – and whether the condition(s) was rare or non-rare were also extracted. Conditions were considered rare if they appeared in the Orphanet database of rare diseases.[30] Orphanet is a unique platform and repository of data on rare diseases and orphan drugs. Patient organisations that were not disease specific, such as hospital charities, carers organisations and hospices, or that did not match the European Federation of Pharmaceutical Industries and Associations (EFPIA) definition of what constitutes a patient organisation were excluded from the analysis. We chose the EFPIA's definition for the following reasons. First, this corresponds the definition used in the wider peer-reviewed literature.[2 31] Second, other commonly used definitions, such as the one from the EMA, refer to the structure of patient organisations' governing bodies, which have to consist of over 50% patients.[32] Considering the high number of patient organisations included in our analysis, this requirement was challenging – if not impossible – to verify. Second, EFPIA's definition indicates what the pharmaceutical industry considers to be a patient organisation. Therefore, it helped us minimize selection bias issues as

For peer review only - http://bmjopen.bmj.com/site/about/guidelines.xhtml

it includes a wide range of organisations. We excluded excluding 181 payments to patient organisations that did not match EFPIA's definition. Sub-group analyses on excluded organisations can be found in the Supplemental Material.

Determining commercial interests

We assessed whether – and the extent to which – a pharmaceutical company holds an interest in the disease supported by a patient organisation. We adapted the definition of 'interest' provided by NICE [33]. An interest is when there is, or could be perceived to be, an opportunity for a pharmaceutical company to benefit in the disease area where the patient organisation operates. This could include cases where the pharmaceutical company has a drug developed or in development for a condition targeted by the patient organisation, or where a drug in the company's portfolio or pipeline is restricted to a specific population affected by the disease supported by the patient organisation. We define portfolio as a group of drugs that a pharmaceutical company has already developed, gained regulatory approval for, and is actively marketing or selling. Conversely, pipeline refers to the collection of drug candidates being developed by a pharmaceutical company, at various stages of development, from preclinical research to clinical trials.

To establish whether an interest existed or not, we first classified the conditions targeted by patient organisations to ICD-11 codes using the online ICD-11 database.[34] ICD-11 codes are mutually exclusive, exhaustive and are arranged as a single hierarchical tree, from level one (most general e.g., *neoplasms*) to five (most specific, e.g. *plasma cell myeloma*). This means that specific diseases are nested within broader classifications.

We then searched companies' annual reports, websites and the ClinicalTrials.gov registry to determine whether each company had an interest in the condition targeted by the patient organisation receiving the payment. Figure 1 schematically illustrates the approach taken to understand whether – and the degree to which – a company has an interest in the conditions (*definitely yes, probably yes, no*). For example, if *Company X* declares in its annual report having a drug in development for multiple myeloma and made a payment to *Blood Cancer UK*, this would be coded as *probably yes,* as the company has a product in its pipeline or portfolio nested within a broader class of conditions targeted by the patient organisation. Conversely, should *Company X* have made a payment to *Myeloma UK,* this would have been coded as *definitely yes,* as there is perfect alignment between the condition targeted by the patient organisation and by *Company X's* drug. Cases in which a company's interest in a certain condition could not be identified were coded as *no.* These, however, were due to limitations in data availability and therefore did not indicate that there was no company interest. Data on pharmaceutical companies' portfolio and pipeline were retrieved from their latest annual reports, company websites and ClinicalTrials.gov.[35]

One investigator (AG) initially coded all data, while the other (IP) blindly re-coded a 30% random sample of payments to validate the data collection process and minimise the risk of reporting errors. We followed this process when validating all data sources described above. Any disagreement was discussed until consensus was reached.

For peer review only - http://bmjopen.bmj.com/site/about/guidelines.xhtml

Analysis of industry funding concentration

We assessed the concentration of industry funding received by patient organisations. In a prior study, Ozieranski and colleagues examined funding disparities among healthcare organizations in the UK in 2015, using the Gini coefficient to assess the distribution of funding.[36] However, the authors acknowledged that the data preparation process presented challenges, limiting the analysis to payments from a single year. While this methodology has its advantages, we found that the time-consuming process of reshaping the data outweighed the benefits over using descriptive statistics. In particular, we calculated (1) the number of companies funding each patient organisations, (2) the share of all industry funding to each patient organisations coming from each contributing company and (3) the share of industry funding of each organisation comprised by the single highest payment.

The Supplemental Material provides further details on the data collection and how the outcomes were constructed. Descriptive statistics and tests, such as ranges and K-sample tests, were presented in the analysis. These statistics were preferred over the mean in light of the skewed distribution of the data analysed. All analyses and data visualisations were performed using Stata 17 and RStudio (*ggplot2* package), respectively.

Patient and public involvement

Patients were not involved in this study as our analyses focused on patient organisations as institutional actors rather than single patients with specific conditions. We plan to disseminate key findings from our analysis to patients and members of the public.

For peer review only - http://bmjopen.bmj.com/site/about/guidelines.xhtml

Results

Between 2018 and 2020, 60 companies made 3,155 payments to 429 patient organisations in the study period, amounting to £42 million. The value of the payments rose substantially over time, from £10.9 million in 2018 to £18 million in 2020. While this is partially due to the different sample of contributing companies across years (see Supplemental Materials), similar upward trends are observed among the 37/60 companies that consistently disclose payments for all year in the analysis (10.9 million in 2018 vs 15.5 million in 2020). These results confirm our expectations of increasing industry funding as expressed in *Hypothesis 1.*

Overall, *diseases of the nervous system* (£8.2 million) was the most funded therapeutic area over time, followed by *neoplasms* (£7.9 million) and *endocrine, nutritional or metabolic diseases* (£5.3 million). About 50% of the payments made to organisations targeting *diseases of the nervous system* were made in 2020 alone. Sanofi, Novartis, Pfizer, UCB and Janssen were the top five funders over the study period (Figure 2). These companies contributed to between 37% and 44% of all payments.

Table 1 summarises the number and value of payments to patient organisations.

Companies' interest in payments to patient organisations

Between 2018-2020, 92% of the payments were directed to patient organisations that were judged to be aligned with their portfolio and pipeline. Only 8% of payments were made to organisations that focused on conditions that could not be linked to a product in the funder's portfolio or pipeline. Table 2 shows the volume and value of payments, broken down by the company's interest variable, overall and whether patient organisations targeted a rare or non-rare disease. Payments to patient organisations targeting a disease for which the company has a product developed or in development (*definitely yes*) made up around 52% regardless of the rarity of the condition targeted as anticipated in *Hypothesis 2.*

The monetary value of payments coded as *definitely yes* accounted for 62% of the overall payment value. However, this was as high as 69% for patient organisations targeting rare diseases, versus 62% for organisations focusing on non-rare conditions. When payments coded as *probably yes* were included, this share increased to 97% for both patient organisations focusing on rare and non-rare diseases.

For peer review only - http://bmjopen.bmj.com/site/about/guidelines.xhtml

Table 1. Number and value of payments from the pharmaceutical industry to UK patient organisations broken down by year and rarity of diseases

	2018	2019	2020	All years (2018-2020)
**Number of payments**	924	1,063	1,168	3,155
**Median payment (IQR; overall)**	£5,136 (£678 - £12,756)	£5,085 (£636 - £12,680)	£9,000 (£1,894 - £15,205)	£5,400 (£921 - £15,000)
**Median payment (IQR; rare)**	£7,190 (£1,249 - £15,408)	£5,085 (£1,236 - £12,204)	£8,500 (£2,500 - £15,000)	£7,000 (£1,777 - £15,000)
**Median payment (IQR; non-rare)**	£3,082 (£616 - £11,468)	£4,800 (£508 - £12,712)	£9,120 (£1,540 - £16,175)	£5,085 (£740 - £14,880)
**Value of payments (£; overall)**	£10,933,715	£13,046,079	£18,015,722	£41,995,516
**Value of payments (£; rare)**	£2,329,017	£3,281,001	£4,180,892	£9,790,909
**Value of payments (£; non-rare)**	£7,991,072	£9,109,462	£12,570,027	£29,670,563
**Number of pharmaceutical companies**	37	47	60	60
**Number of patient organisations**	221	268	294	429

Abbreviations: IQR (Interquartile range).
Notes: All payments are expressed in 2020 GBP. The Supplemental Materials detail the inflation multipliers and conversion rates used, both retrieved from the Office of National Statistics (ONS) website. Further details on how patient organisation data were cleaned and coded, please see the Supplemental Materials . Please note that the number of pharmaceutical companies and patient organisations making and receiving payments across the study period (2018-2020) refers to companies and organisations that made or received at least one payment, respectively.

Table 2. Volume and value of payments by company interests across all years

**PO type**	**Company's interest**	**Volume**; n (%) *All years (2018-2020)*	**Value**: £ (%) *All years (2018-2020)*
Overall†	Definitely yes	1,627 (52%)	£26,002,527 (62%)
	Probably yes	1,265 (40%)	£12,724,965 (30%)
	No*	263 (8%)	£3,262,205 (8%)
Rare	Definitely yes	339 (54%)	£6,725,300 (69%)
	Probably yes	262 (41%)	£2,713,531 (28%)
	No*	34 (5%)	£352,078 (4%)
Non-rare	Definitely yes	1,276 (55%)	£19,121,806 (62%)
	Probably yes	977 (42%)	£9,827,287 (35%)
	No*	71 (3%)	£721,468 (3%)

For peer review only

Notes: *Definitely yes* indicates payments directed to patient organisations that operated in a disease area (ICD-11 level 4 or higher) for which the company has a product in its portfolio or pipeline. *Probably yes* indicates directed to patient organisations that operated in a disease area (ICD-11 level 3 or lower) for which the company has a product in its portfolio or pipeline. *No* refers to directed to patient organisations that operated in a disease area for which no link could be found to the company's portfolio or pipeline. The higher the ICD-11, the more specific the condition. For example, if the ICD-11 level 4 is *Plasma cell neoplasms,* level 2 would be *Neoplasms of hematopoietic or lymphoid tissues.* Further details on how this variable was constructed can be found in the Supplemental Material.

*Please note that the *No* category of interest conservatively includes also interests that were considered as unclear.

†Please note that the *Overall* results are not a sum of the *Rare* and *Non-rare* results, as they also include patient organisations that could not be classified in either group.

For peer review only

Industry funding of patient organisations focusing on rare vs. non-rare conditions

Of the £42 million in payments from industry to patient organisations, £9.8 million (23%; n=635) were directed to organisations focusing on rare diseases while £29.7 million (71%; n=2,323) to organisations supporting non-rare conditions. The remaining 6% were directed to non-disease-specific patient organisations, which were excluded from the analysis.

From 2018 to 2020, the payments to patient organisations targeting rare diseases increased more compared to those focusing on more prevalent conditions (80% vs 57%). Median payments received by patient organisations were significantly different ($p<0.001$) depending on the rarity of the disease they focused on, with rare patient organisations receiving higher payments. Linking these results to *Hypothesis 3,* we can see that while patient organisations supporting rare diseases received less funding in the period, there was a significantly higher increase in payment value.

Among the top five recipients overall in 2018 and 2019, two focused on rare diseases (Myeloma UK and the Cystic Fibrosis Trust). In 2020 no organisation targeting rare conditions appeared in the top five recipients. Figure 3 shows therapeutic areas in order from most to least funded, broken down by rarity of disease targeted. In the case of organisations focusing on rare diseases, *neoplasms* and *endocrine, nutritional or metabolic disease* received most funds across years. Together, the top three most funded disease areas represented more than half of overall funding. When looking at the conditions that attracted most funding, multiple sclerosis was first (£4.1 million), followed by diabetes (£2.4 million) and epilepsy (£1.7 million). Cystic fibrosis and multiple myeloma were the only rare diseases that were among the top ten most funded conditions overall, attracting £1.3 and £1.2 million, respectively.

For peer review only

Industry funding concentration

On average, each patient organisation received payments from approximately two companies every year, with 1.97 (SD:1.74) and 2.21 (SD:1.91) companies funding patient organisations targeting rare and non-rare diseases, respectively. However this difference was not statistically significant ($\chi^2 = 0.197$, *p-value* = 0.657).

In our sample, the median yearly payment of a company to a patient organisation comprised 33% of the its overall industry payments (IQR: 11.2%-100%). When looking at patient organisations focusing on rare diseases, the median company contribution was as high as 42% (IQR: 14.5%-100%) versus 31% (IQR: 11.6%-99.7%) for non-rare conditions ($\chi^2 = 7.141$, *p-value* = 0.008).

Finally, the share of industry funding comprised by the single highest payment per organisation amounted to an average of 73% (SD: 0.29) for all years, ranging from a minimum of 10% to a maximum of 100%. When broken down by year, this percentage slightly decreased over time. The highest value payment in the case of patient organisations targeting rare diseases made up a larger share of the overall industry funding (median: 86%, IQR: 0.527-1), despite not significant, compared to those focusing on more prevalent conditions (median: 79%, IQR: 0.428-1). While there was not a significant difference in the number of funding companies between patient organisations supporting rare and non-rare diseases as stated in *Hypothesis 4*, the former relied on larger payments.

For peer review only

Discussion

In this study, we evaluated the financial links between the pharmaceutical industry and patient organisations in the UK between 2018 and 2020. This is the first study to document the almost-perfect concordance of pharmaceutical company interests and patient organisation funding in the UK. Almost all industry payments during our study period – in terms of both volume (92%) and value (92%) – were to patient organisations aligned with pharmaceutical companies' portfolios and pipelines. Approximately a quarter of industry funding to patient organisations from 2018 to 2020 was directed towards organisations focusing on rare diseases (£9.8 million / £42 million). Finally, we found that patient organisations targeting rare diseases relied on payments from fewer companies but of higher value compared to organisations focusing on non-rare diseases.

The almost-perfect concordance between industry interests and patient organisation activities likely reflect the commercial attractiveness of conditions targeted by pharmaceutical companies.[2 37] Such close alignment between the interests of companies and patient organisations might undermine the credibility of patient organisations as perceived by the general public and might raise questions about patient organisations' inputs in regulatory and health technology appraisals.[9 38 39] A recent study found that during NICE appraisal meetings fewer than 25% of all relevant financial ties between patient organisations and pharmaceutical companies were disclosed.[40] As discussed by the Mandeville and colleagues, this lack of transparency increases the risk of conflict of interest.

Our findings make an important contribution to the existing body of literature on industry funding of patient organisations. Ozieranski et al found that industry donated over £57 million to UK patient organisations from 2012 to 2016, an average of £11.5 million per year.[2] The authors also observed that payments were concentrated in commercially attractive therapeutic areas, with organisations focusing on cancer receiving more than 36% of overall payments.[2] However, the study did not examine whether companies were more likely to fund organisations that target diseases for which they have already developed or are currently developing products. Another earlier study examined payments to Swedish patient organisations and found an association between drug commercialisation and industry funding.[10] The authors did not take into account products in the companies' pipelines nor drugs that might had not yet launched in Sweden. Considering that patient organisations have an important role not only in the post-commercialisation phase but also in the R&D and approval stages. We therefore developed a replicable classification model to determine whether payments from companies were directed at organisations that were aligned with their portfolios and pipelines.

Patient organisations focusing on rare diseases can drive both supply of and demand for medicinal products due to their research, advocacy and education role. [4 41] As a result of their close ties with patients, these organisations have the credibility and power to educate patient communities, advocate for access to available therapies and raise awareness on the unmet need of certain conditions.[4 17 42] Although a large share of both the value and number of payments were directed to patient organisations focusing on rare diseases, most funds targeted commercially attractive rare conditions, such as multiple myeloma and cystic fibrosis, where the unmet need is relatively low compared to other rare conditions. These are diseases that have

For peer review only - http://bmjopen.bmj.com/site/about/guidelines.xhtml

relatively high prevalence and for which 10 and 29 treatments, respectively, are currently approved for use in Europe.[30 43] This poses the risk of widening already existing health inequities, where severe and debilitating rare conditions that affect a small number of patients do not receive the resources they need and have to rely on limited public grants.[44]

Finally, our analysis showed that patient organisations focusing on rare diseases are funded by very few companies, relying on a single payment for over 80% of their industry-reported income. Despite the share of industry contributions among the overall patient organisation's income was found to be low in the literature,[11] this increases the risk of pursuing the company's commercial interests rather than objectively representing a patient body.[12] On average, patient organisations received payments from 2.1 (SD:1.8) pharmaceutical companies, ranging from 1 to a maximum of 13, which was recorded in 2020 for Genetic Alliance UK, a national charity and an alliance of over 200 patient organisations, supporting those affected by rare genetic conditions. This is aligned with findings from a recent study investigating the distribution of payments from industry to Danish patient organisations, which found that on average, most organisations were funded by 2.6 (SD:2.1) on average.[15]

These findings have important implications for policy and practice. To minimise conflicts of interests and maintain the integrity of patient organisations, particular attention should be paid to funding from companies in the immediate period before or after a patient organisation has endorsed this company's product.[40] One way of avoiding potential conflicts of interest is through increased transparency. Despite considerable progress on this front, especially in terms of reporting the monetary value of industry payments, there are still gaps in reporting.[45] Furthermore, financial independence of patient organisation is fundamental for making sure that patients' interest is at the forefront of the organisations' agenda. This is exemplified by the opposition of AbbVie-sponsored patient organizations to biosimilar switching in various countries, which underscores the potential harm of financial dependency on public health priorities.[15] In the long term, policymakers should make sure that patient organisations receive adequate public funding regardless of whether they focus on conditions that are profitable for the industry. Such public funding is particularly important for patient organisations supporting rare diseases, as relatively few companies have financial links with patient organisations focusing on rare diseases, potentially creating high reliance on few high-value payments.

This study had limitations. First, the lack of mandatory reporting of payments to patient organizations by companies that do not comply with the ABPI Code is a major limitation of our analysis.[46] For example, our dataset does not include payments by Vertex, a company with a rare-focused portfolio and a strong presence in cystic fibrosis.[47] Even for companies that are signatories of the ABPI Code, underreporting of payments to patient organizations and removal of disclosure reports from the public domain has been observed.[13 48 49] Although the ABPI Code requires companies to disclose their payments to patient organizations annually, it does not mandate the publication of disclosure reports from previous years on their websites.[26] As a result, our findings should be interpreted with caution given the incomplete nature of the available data. Linked to this we have assumed that companies which disclosed no payments in a given year, made no payments in that year. Second, the sample size of pharmaceutical companies making payments to patient organisations was not constant over time. In fact, we

For peer review only - http://bmjopen.bmj.com/site/about/guidelines.xhtml

recorded payments from 37 companies in 2018 versus 60 companies in 2020. While this might bias our results, the impact of this was considered to be limited. Most notably, despite the differences in sample size, absolute values of payments are very similar when considering only companies that consistently disclosed payments across years (n=37). For example, in 2020, payments from those companies that disclosed consistently across the study period amounted to £15.5 million versus £18 million when any payment disclosed in that year is considered (86%). Third, in our assessment of company interests, we made a conversative assumption that only patient organisations which target relatively narrow conditions were eligible to be coded as *definitely ye*s. Despite this assumption, we concluded that more than half of payments were in therapeutic areas in which companies had a clear interest. Finally, our analysis focused on a recent time period (2018-2020). While previous publications show similar trends in terms of the most funded diseases and absolute value of payments,[2 10] lending credibility to our analysis and underlying data, it is still unclear whether these trends hold over time and their generalisability to other periods.

There are several avenues which can be explored further to build on this analysis. While some of the previous literature on the topic has focused on the financial dependency of patient organisations' budgets from pharmaceutical funding,[11] whether this differs depending on the rarity of the disease targeted has not been explored. Due to the small number of patients affected by rare conditions, patient organisations that target such conditions may be less well-equipped to finance their activities via charitable events and may rely more heavily on contributions from pharmaceutical companies. Lastly, while our analysis did not evaluate the effect of Covid-19 on the financial dynamics between pharmaceutical companies and patient organisations, we expect that the pandemic had a substantial effect on the type, value and distribution of payments. Future research should examine the impact of Covid-19 on industry funding of patient organisations.

Conclusions

Almost all industry funding of UK patient organisations between 2018 and 2020 was in areas that were aligned with companies' approved drug portfolios and research and development pipelines. Pharmaceutical companies spent a larger amount on patient organisations focusing on rare diseases and these organisations relied on a small of companies for their funding.

For peer review only - http://bmjopen.bmj.com/site/about/guidelines.xhtml

Acknowledgements: We thank Dr Huseyin Naci and Dr Olivier Wouters, both from the Department of Health Policy at the London School of Economics and Political Science, for providing comments on earlier drafts of this paper. We also acknowledge Dr Mylene Lagarde and Dr Panos Kanavos for the support provided when finalising the manuscript.

Contributors: AG developed a preliminary version of the study and developed it further with IP. AG collected the data. AG and IP did the analysis, wrote and reviewed the manuscript. Both authors had full access to all of the data (including statistical reports and tables) in the study and can take responsibility for the integrity of the data and the accuracy of the data analysis. The corresponding author attests that all listed authors meet authorship criteria and that no others meeting the criteria have been omitted. AG is the guarantor.

Funding: This research received no specific grant from any funding agency in the public, commercial or not-for-profit sectors. AG is funded by the ESRC for her doctoral research (Project reference: 2480077) and is employed by Imperial College London. IP is funded by the ESRC for her doctoral research (Project reference: 2311285) and is employed by Imperial College London.

Competing interest statement: All authors have completed the Unified Competing Interest form (available on request from the corresponding author) and declare: no support from any organisation for the submitted work; no financial relationships with any organisations that might have an interest in the submitted work in the previous three years, no other relationships or activities that could appear to have influenced the submitted work.

Ethical approval: This study does not involve human participants and ethical approval was not required.

Data sharing: A dataset of all publicly available data used in the study is available from the corresponding author at a.gentilini@lse.ac.uk.

Transparency declaration: The lead author affirms that the manuscript is an honest, accurate, and transparent account of the study being reported; that no important aspects of the study have been omitted; and that any discrepancies from the study as planned (and, if relevant, registered) have been explained.

The Corresponding Author has the right to grant on behalf of all authors and does grant on behalf of all authors, an exclusive licence (or non-exclusive for government employees) on a worldwide basis to the BMJ Publishing Group Ltd to permit this article (if accepted) to be published in BMJ editions and any other BMJPGL products and sublicences such use and exploit all subsidiary rights, as set out in our licence.

For peer review only - http://bmjopen.bmj.com/site/about/guidelines.xhtml

References

1. EFPIA. EFPIA Code of Practice on the Relationships between the Pharmaceutical Industry and Patient Organisations: European Federation of Pharmaceutical Industries and Associations, 2011.
2. Ozieranski P, Rickard E, Mulinari, Shai. Exposing drug industry funding of UK patient organisations. *BMJ* 2019;365:l1806. doi: 10.1136/bmj.l1806
3. Polich GR. Rare disease patient groups as clinical researchers. *Drug Discovery Today* 2012;17(3):167-72. doi: https://doi.org/10.1016/j.drudis.2011.09.020
4. Geissler J, Ryll B, di Priolo SL, et al. Improving Patient Involvement in Medicines Research and Development::A Practical Roadmap. *Therapeutic Innovation & Regulatory Science* 2017;51(5):612-19. doi: 10.1177/2168479017706405
5. MHRA. Patient Involvement Strategy 2021-25: Medicines and Healthcare products Regulatory Agency 2020.
6. MHRA. Putting patients first: A new era for our agency. Delivery Plan 2021-2023: Medicines and Healthcare products Regulatory Agency 2020.
7. NICE. Public Involvement Programme - Overview of technology appraisals: A factsheet for patient and carer organisations: National Institute for Health and Care Excellence, 2014
8. Fabbri A, Parker L, Colombo C, et al. Industry funding of patient and health consumer organisations: systematic review with meta-analysis. *BMJ* 2020;368:l6925. doi: 10.1136/bmj.l6925
9. Rose SL, Highland J, Karafa MT, et al. Patient Advocacy Organizations, Industry Funding, and Conflicts of Interest. *JAMA Intern Med* 2017;177(3):344-50. doi: 10.1001/jamainternmed.2016.8443
10. Mulinari S, Vilhelmsson A, Rickard E, et al. Five years of pharmaceutical industry funding of patient organisations in Sweden: Cross-sectional study of companies, patient organisations and drugs. *PLoS One* 2020;15(6):e0235021. doi: 10.1371/journal.pone.0235021 [published Online First: 20200624]
11. Ozieranski P, Pitter JG, Rickard E, et al. A 'patient-industry complex'? Investigating the financial dependency of UK patient organisations on drug company funding. *Sociol Health Illn* 2022;44(1):188-210. doi: 10.1111/1467-9566.13409 [published Online First: 20211207]
12. Rose SL. Patient advocacy organizations: institutional conflicts of interest, trust, and trustworthiness. 2014(1748-720X (Electronic))
13. Lexchin J, Batt S, Goldberg D, et al. National patient groups in Canada and their disclosure of relationships with pharmaceutical companies: a cross-sectional study. *BMJ Open* 2022;12(3):e055287. doi: 10.1136/bmjopen-2021-055287
14. Parker L, Fabbri A, Grundy Q, et al. "Asset exchange"—interactions between patient groups and pharmaceutical industry: Australian qualitative study. *BMJ* 2019;367:l6694. doi: 10.1136/bmj.l6694
15. Mulinari S, Pashley D, Ozieranski P. Advancing international comparison of pharmaceutical industry funding of patient advocacy: Focus on Denmark. *Health Policy* 2022;126(12):1256-62. doi: https://doi.org/10.1016/j.healthpol.2022.11.003
16. Wouters OJ, McKee M, Luyten J. Estimated Research and Development Investment Needed to Bring a New Medicine to Market, 2009-2018. 2020(1538-3598 (Electronic))
17. Aymé S, Kole A, Groft S. Empowerment of patients: lessons from the rare diseases community. *Lancet* 2008;371(9629):2048-51. doi: 10.1016/s0140-6736(08)60875-2
18. Gamba S, Magazzini L, Pertile P. R&D and market size: Who benefits from orphan drug legislation? *Journal of Health Economics* 2021;80:102522. doi: https://doi.org/10.1016/j.jhealeco.2021.102522
19. Waxman HA. The Waxman Report : How Congress Really Works: First edition. New York : Twelve, 2009. 2009.

For peer review only - http://bmjopen.bmj.com/site/about/guidelines.xhtml

20. Office of the Federal Register NAaRA. Orphan Drug Act - 6 Stat. 2049. In: Office. USGP, ed., 1983.
21. European Commission. Regulation (EC) No 141/2000 of the European Parliament and of the Council of 16 December 1999 on Orphan Medicinal Products, 2000.
22. Halley MC. From "Ought" to "Is": Surfacing Values in Patient and Family Advocacy in Rare Diseases. 2021(1536-0075 (Electronic))
23. Dunkle M, Pines W, Saltonstall PL. Advocacy Groups and Their Role in Rare Diseases Research. In: Posada de la Paz M, Groft SC, eds. Rare Diseases Epidemiology. Dordrecht: Springer Netherlands 2010:515-25.
24. Scottish Medicines Consortium. Patient and Clinician Engagement (PACE) process [Available from: https://www.scottishmedicines.org.uk/how-we-decide/pace/2023.
25. Disclosure UK. ABPI Patient Organisations database 2021 [Available from: https://search.disclosureuk.org.uk/.
26. PMCPA. ABPI Code of Practice 2021 [Available from: https://www.pmcpa.org.uk/the-code/2021-interactive-abpi-code-of-practice/.
27. ONS. Consumer price inflation time series: Office for National Statistics; 2022 [Available from: https://www.ons.gov.uk/economy/inflationandpriceindices/timeseries/l55o/mm23.
28. HMRC. HMRC yearly average and spot rates: HM Revenue and Customs; [Available from: https://www.gov.uk/government/publications/exchange-rates-for-customs-and-vat-yearly.
29. HMRC. HMRC yearly average and spot rates: HM Revenue and Customs; 2022 [Available from: https://www.gov.uk/government/publications/exchange-rates-for-customs-and-vat-yearly.
30. Orphanet. The portal for rare diseases and orphan drugs 2022 [Available from: https://www.orpha.net/consor/cgi-bin/Disease_Search_Simple.php?lng=EN.
31. Rothman SM, Raveis VH, Friedman A, et al. Health Advocacy Organizations and the Pharmaceutical Industry: An Analysis of Disclosure Practices. *American Journal of Public Health* 2011;101(4):602-09. doi: 10.2105/AJPH.2010.300027
32. European Medicines Agency. Criteria to be fulfilled by patient, consumer and healthcare professional organisations involved in European Medicines Agency (EMA) activities 2018 [Available from: https://www.ema.europa.eu/en/documents/regulatory-procedural-guideline/criteria-be-fulfilled-patient-consumer-healthcare-professional-organisations-involved-european_en.pdf.
33. NICE. Policy on declaring and managing interests for NICE advisory committees, 2018.
34. WHO. ICD-11 for Mortality and Morbidity Statistics 2022 [Available from: https://icd.who.int/browse11/l-m/en#/http://id.who.int/icd/entity/465177735?view=G0.
35. NIH U.S. National Library of Medicine. ClinicalTrials.gov [Available from: https://clinicaltrials.gov/2022.
36. Ozieranski P, Csanadi M, Rickard E, et al. Analysis of Pharmaceutical Industry Payments to UK Health Care Organizations in 2015. 2019(2574-3805 (Electronic))
37. Hughes DA, Poletti-Hughes J. Profitability and Market Value of Orphan Drug Companies: A Retrospective, Propensity-Matched Case-Control Study. 2016(1932-6203 (Electronic))
38. McCoy MS, Carniol M, Chockley K, et al. Conflicts of Interest for Patient-Advocacy Organizations. *New England Journal of Medicine* 2017;376(9):880-85. doi: 10.1056/NEJMsr1610625
39. Jones K. In whose interest? Relationships between health consumer groups and the pharmaceutical industry in the UK. 2008(1467-9566 (Electronic))
40. Mandeville KL, Barker R, Packham A, et al. Financial interests of patient organisations contributing to technology assessment at England's National Institute for Health and Care Excellence: policy review. *BMJ* 2019;364:k5300. doi: 10.1136/bmj.k5300
41. Mavris M, Le Cam Y. Involvement of patient organisations in research and development of orphan drugs for rare diseases in europe. 2012(1661-8769 (Print))
42. Bedlington N, Geissler J, Houyez F, et al. Role of Patient Organisations. In: Facey KM, Ploug Hansen H, Single ANV, eds. Patient Involvement in Health Technology Assessment. Singapore: Springer Singapore 2017:401-10.

For peer review only - http://bmjopen.bmj.com/site/about/guidelines.xhtml

43. European Medicines Agency. European public assessment reports (EPAR), 2022.
44. Baggott R, Jones K. The Big Society in an age of austerity: threats and opportunities for Health Consumer and Patients' Organizations in England. 2015(1369-7625 (Electronic))
45. Lexchin J. Association between commercial funding of Canadian patient groups and their views about funding of medicines: An observational study. *PLOS ONE* 2019;14(2):e0212399. doi: 10.1371/journal.pone.0212399
46. Ozieranski P, Martinon L, Jachiet P-A, et al. Accessibility and quality of drug company disclosures of payments to healthcare professionals and organisations in 37 countries: a European policy review. *BMJ Open* 2021;11(12):e053138. doi: 10.1136/bmjopen-2021-053138
47. Vertex Pharmaceuticals Incorporated. Our Science [Available from: https://www.vrtx.com/our-science/2023.
48. Ozieranski P, Csanádi M, Rickard E, et al. Under-reported relationship: a comparative study of pharmaceutical industry and patient organisation payment disclosures in the UK (2012–2016). *BMJ Open* 2020;10(9):e037351. doi: 10.1136/bmjopen-2020-037351
49. Colombo C, Mosconi P, Villani W, et al. Patient organizations' funding from pharmaceutical companies: is disclosure clear, complete and accessible to the public? An Italian survey. *PLoS One* 2012;7(5):e34974. doi: 10.1371/journal.pone.0034974 [published Online First: 20120509]

For peer review only - http://bmjopen.bmj.com/site/about/guidelines.xhtml

Figure legend

Figure 1. Classification model to determine company interests in patient organisation funding Note: An interest is when there is, or could be perceived to be, an opportunity for a pharmaceutical company to benefit in the disease area where the patient organisation operates.

Figure 2. Cumulative value of payments by receiving patient organisation and funding company from 2018-2020

Figure 3. Cumulative value of payments by patient organisation type and therapeutic area from 2018-2020

For peer review only

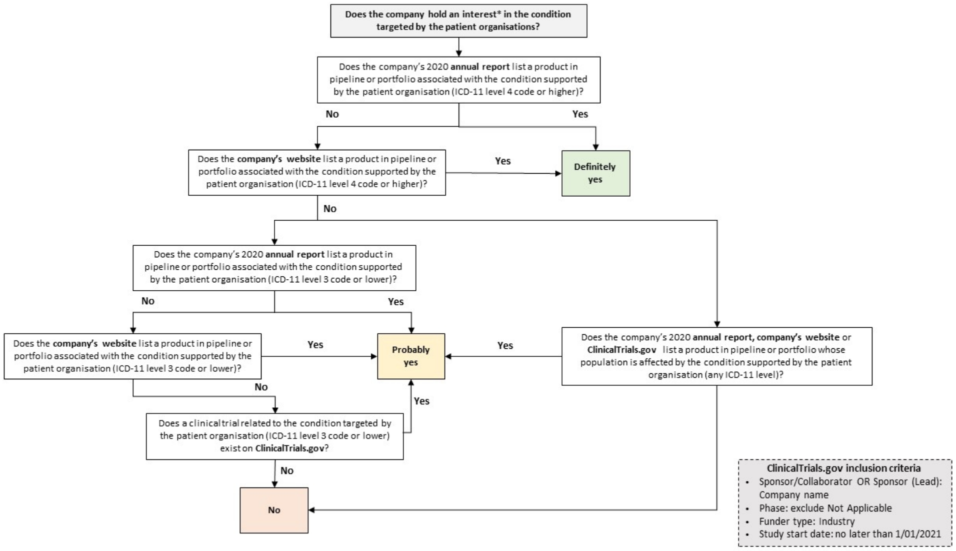

Caption: Classification model to determine company interests in patient organisation funding

Notes: An interest is when there is, or could be perceived to be, an opportunity for a pharmaceutical company to benefit in the disease area where the patient organisation operates.

338x190mm (96 x 96 DPI)

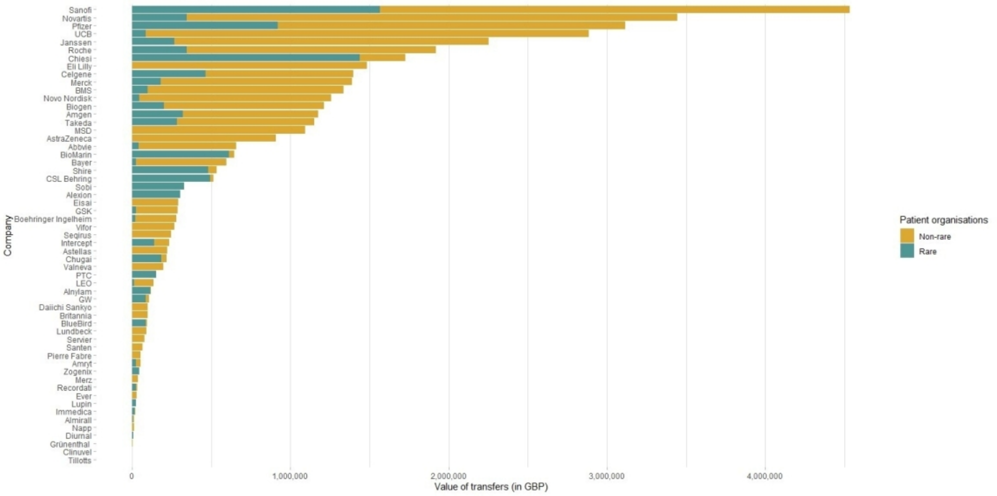

Caption: Cumulative value of payments by receiving patient organisation and funding company from 2018-2020

130x64mm (300 x 300 DPI)

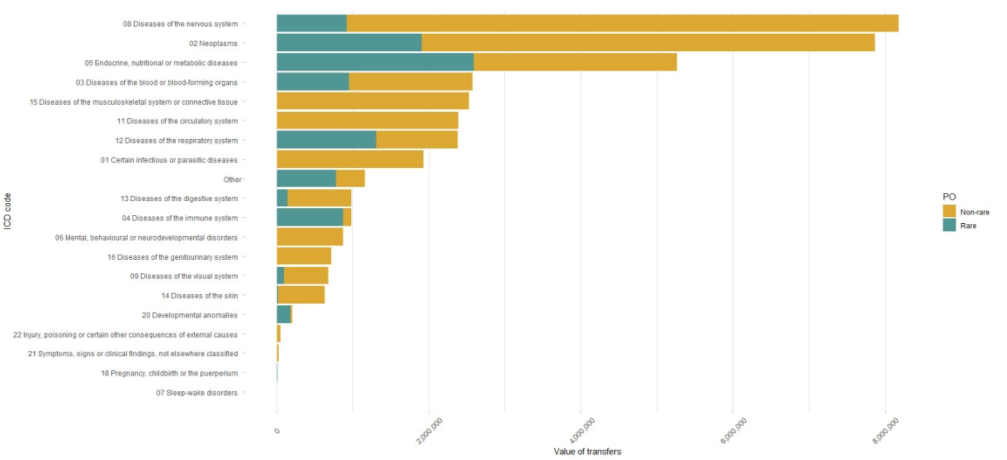

Caption: Cumulative value of payments by patient organisation type and therapeutic area from 2018-2020

1161x525mm (37 x 37 DPI)

Supplemental Material

Data collection

Payments

Data on payments from pharmaceutical companies to POs from 2018 to 2020 were retrieved in January 2022 from the Disclosure UK patient organisation gateway.1 The gateway was launched in 2020 and is a collection of hyperlinks to companies' disclosure of payments to patient organisations. Disclosing financial payments to patient organisations is a requirement of Clause 29 of the Association of British Pharmaceutical Industry (ABPI) Code of Practice.2 However, companies signed up to abide by the ABPI Code, accepting the jurisdiction of the Prescription Medicines Code of Practice Authority (code regulator) extends beyond those who are ABPI members and is expected to include most pharmaceutical companies operative in the UK. We screened the websites of all pharmaceutical companies abiding by the ABPI Code, most of which provided a link in listed in the Disclosure UK database, and retrieved payments information companies' websites to ensure all payments were captured. If payments were not disclosed in Disclosure UK nor in the company's website, the company was assumed not to have made any payment to patient organisations in that year(s).

AG extracted payment disclosures from companies' websites, comprising of the name of the patient organisation, the year in which the payment was made, the reason why it was made and its value. Given that a consolidated database of payments was not available and payments needed to be manually compiled from each individual company's website, IP randomly checked 30% of payments to validate the data collection process and minimise the risk of reporting errors.

All payments were first adjusted for inflation using the ONS Consumer Price Index [3] and then converted to British Pounds (GBP), using the ONS historical yearly conversion rates.[4 5] All payments are in 2020 GBP. Data on pharmaceutical companies' portfolio and pipeline were retrieved from their latest annual report, company website and ClinicalTrials.gov,[6] in order of screening.

Therapeutic areas

Patient organisations' websites were also screened to understand the condition(s) they focused on. For example, in the case of *Blood Cancer UK*, their mission is to "*beat blood cancer*", therefore, the condition supported was coded as blood cancer.

After being identified as described above, conditions were further classified into rare and non-rare.

Conditions were considered rare if they appeared in the Orphanet database of rare diseases regardless of their classification level (group of disorders, disorders or subtypes of disorders).[9]

For example, multiple myeloma appears in the Orphanet database of rare diseases, therefore a patient organisation focusing this condition would be categorised as rare-focused. When condition sub-types appeared in the Orphanet database, the patient organisation's website was

For peer review only - http://bmjopen.bmj.com/site/about/guidelines.xhtml

screened to check whether its focus was on rare conditions. For example, *Metabolic Support UK's* motto is "*Your rare condition. Our common fight*" and was therefore assumed to be rare disease-focused. Conversely, should a patient organisation focus on a broader condition such as blood cancer with no sole focus on rare conditions, the organisation would be conservatively considered non-rare. While this approach was preferred as it did not require further assumptions, it entails that only more specialised patient organisation are considered as rare. Such approach might have led to the number and overall value of payments from pharmaceutical companies to rare diseases-focused patient organisations being underestimated, as these organisaitons are expected to get less payments than more generalist ones (e.g. multiple myeloma vs blood cancer).

A third category (*unclear)* was created for non-disease-specific patient organisations, such as coalition of charities or organisations focused on palliative care for terminally ill patients. This category was excluded from the main analyses, but sub-group analyses are reported at the end of the Supplemental Material.

<u>Companies' interest</u>

We developed a methodology to assess the extent to which a pharmaceutical company holds an interest in the disease supported by a patient organisation. For the purpose of this analysis, we adapted the definition of interest provided by NICE.[11] An interest is when there is, or could be perceived to be, an opportunity for a pharmaceutical company to benefit in the disease area where the patient organisation operates. This could include situations where the pharmaceutical company has a drug developed or in development for a condition supported by the patient organisation, or where a drug in the company's portfolio or pipeline is restricted to a specific population affected by the disease supported by the patient organisation.

As first step, the conditions supported by patient organisations were translated into ICD-11 codes using the online ICD-11 database.[12]

ICD-11 codes are mutually exclusive, exhaustive and are arranged as a single hierarchical tree. This means that specific diseases are nested within broader classifications. An example for multiple myeloma is shown in Table 1 below.

Table 1. Example of ICD-11 classification, Multiple myeloma

Hierarchy level	Condition	ICD-11 code
Level 1	Neoplasms	2
Level 2	Neoplasms of haematopoietic or lymphoid tissues	2A
Level 3	Mature B-cell neoplasms	2A8
Level 4	Plasma cell neoplasms	2A83
Level 5	Plasma cell myeloma	2A83.1

In this example, multiple myeloma is nested within *Plasma cell myeloma*, who is in its turn nested within *Plasma cell neoplasms* and so on up to *Neoplasms*.

For peer review only - http://bmjopen.bmj.com/site/about/guidelines.xhtml

Subsequently, companies' annual reports, website and the ClinicalTrials.gov database were searched to assess whether the each company had an interest in the condition supported by the patient organisation receiving the payment. The diagram in the main document (Figure 1) schematically illustrates the approach taken to understand whether the company definitely, probably or did not have an interest in the condition. Figure 1 below illustrates the source of companies' interests.

For example, if *Company X* reports in its annual report having a drug in development for multiple myeloma and transferred a sum of money to *Blood Cancer UK*, this would be coded as *probably yes*, as the company has a product in its pipeline or portfolio associated with a condition supported by the patient organisation. In this case, the ICD-11 level would be 2, *Neoplasms of haematopoietic or lymphoid tissue*, under which multiple myeloma is nested. Conversely, should *Company X* have made a payment to *Myeloma UK*, this would have been coded as *definitely yes*, as there is perfect alignment between the condition supported by the patient organisation and by *Company X's* drug.

Situations where a company's interest in a certain condition could not be identified indicate an impossibility of identifying such link, rather than the lack thereof.

Figure 1. Source of companies interests

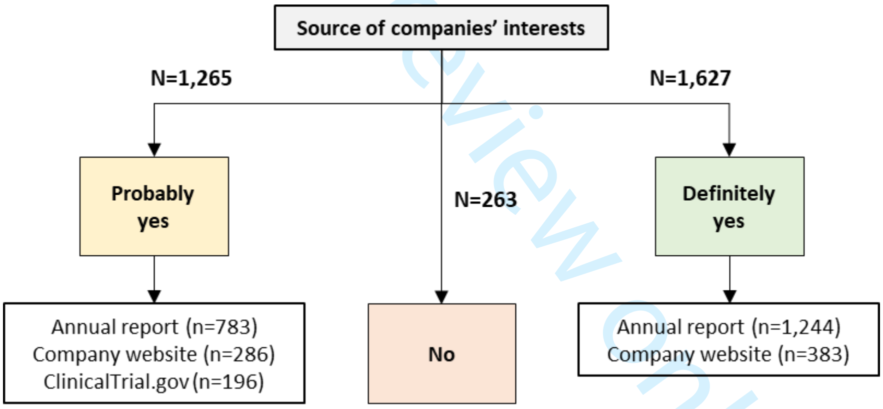

For peer review only

Variables cleaning and coding

Table 2. Description of key variables in payment database

Variables name	Description	Details
**Company**	Standardised company name	Company name as reported on company website.<br>During our study period (2018-2020), two mergers were observed among the companies included in the analysis: BMS and Celgene, and Takeda and Shire. Although these companies had merged, we treated them as separate entities because their disclosures were reported separately even after the acquisition.
**ABPI member**	ABPI membership of company; *source: ABPI full members list*	0 = not ABPI member, 1 = ABPI member
**Company_condition**	Concatenation of company name and disease area targeted by the patient organisation	Concatenation used for coding and analysis purposes
**Company interest**	Whether the company hold an interest* in the condition targeted by the patient organsiation	- **Definitely yes**: the company's annual report or website list a product for the condition targeted by the patient organisation in its portfolio/pipeline (ICD-11 level 4 or above)<br>- **Probably yes**: the company's annual report or website list a product for the condition targeted by the patient organisation in its portfolio/pipeline OR a clinical trial for which the company is sponsor is listed for the disease targeted by the patient organisation OR a drug in the company's pipeline/portfolio is restricted to a specific population affected by the disease targeted by the patient organisation (ICD-11 level 3 or below)<br>- **No** : None of the above
**Source**	Source of company interest variable	Annual report, company website, ClinicalTrials.gov, none
**Name of PO**	Name of patient organization as reported by companies in disclosure report	-
**Standardised PO name**	Standardised name of patient organization to avoid duplicates and inconsistencies	For coding purposes, names of patient organisations were standardised. The following steps were taken:<br>1. Patient organisations' names for typos, abbreviations, spelling mistakes and duplicated within the companies' disclosures (e.g. Crohn's & Colitis UK and CCUK would both be standardized to Crohn's and Colitis UK);<br>2. If the patient organisation changed name over time, the latest name on record was used;

		3. If the two patient organisations merged over the study period, the name of the merged entity was used (e.g. the British Lung Foundation and Asthma UK merged into Asthma + Lung UK);<br>4. Separate entries were made for patient organsiations under the same umbrella but focusing on different geographical entities (e.g. Alzheimer UK vs Alzheimer Scotland)
**Reason for exclusion**	Reason why the organisation was excluded from the analysis	- Not UK organisation (not aligned with geographical scope e.g. Irish, US-based);<br>- For profit company (not aligned with definition of patient organization used in the study);<br>- Missing information (organisations for whose nature is unclear i.e. patient organisation website could not be identified)
**ICD-11**	Classification of disease targeted by the patient organisation according to the WHO ICD-11; *source: ICD WHO website*	General classification (ICD-11 chapters)<br>*See Excel file, Inputs tab*
**Condition**	Condition targeted by patient organisation as reported on website	e.g. Blood Cancer UK would fall under ICD-11 code 02 Neoplasms, with *blood cancer* being the condition
**Charity number (if any)**	Charity number as reported in the organization website or as reported in the England and Wales Charity Commission website	When both England/Wales and Scotland or Northern Ireland charity numbers were provided, the former was chosen. Scotland and Northern Ireland charity numbers were reported only when those for England/Wales were missing
**Company number (if charity number missing)**	Company number as reported in the organization website or as reported in the Government Company Information Service wesbite if the patient organization cannot be found in the charity commission database (e.g. limited by guarantee company)	When both England/Wales and Scotland or Northern Ireland charity numbers were provided, the former was chosen. Scotland and Northern Ireland charity numbers were reported only when those for England/Wales were missing
**Link**	Link of patient organisation website	-
**Rare disease**	Whether the condition or one of the conditions targeted by the patient organisation is considered as rare	A condition was considered as rare if it under at least one of the following criteria:<br>1. The condition is listed in Orphanet list of rare diseases regardless of its ICD-11 level classification;<br>2. In their website, the patient organisation explicitly describe the disease they target as rare (e.g. *Metabolic Support UK's* motto is "*Your rare condition. Our common fight"* and was therefore assumed to be rare disease-focused)

For peer review only - http://bmjopen.bmj.com/site/about/guidelines.xhtml

**Details of payment**	Details of payment as reported by companies in disclosure report	-
**Country**	Country of payment	The country considered for the entire database is the UK
**Year**	Year of payment	2018, 2019, 2020
**Currency**	Currency of payment	Currency the payment is reported in the disclosure reports (i.e. EUR, GBP, USD, CHF, SEK, NKK)
**Currency_year**	Concatenation of currency and year of payment for conversion purposes	-
**Value of payment**	Value of payment in original currency as reported by companies in disclosure report	In-kind payments were removed from the analysis as no monetary value could be associated to such payments
**Value in 2020 pounds**	GBP converted and inflation adjusted value of payment	See details in *Inputs* sheet

*An interest is when there is, or could be perceived to be, an opportunity for a pharmaceutical company to benefit in the disease area where the patient organisation operates.

For peer review only - http://bmjopen.bmj.com/site/about/guidelines.xhtml

Disclosure details

Table 3. Disclosure details for companies disclosing at least one payment between 2018-2020

Company	2018	2019	2020	Years of complete data
AbbVie	0	1	1	2
Alexion	1	1	1	3
ALK-Abello	0	0	1	1
Amirall	0	1	1	2
Alnylam	1	1	1	3
Amgen	1	1	1	3
Amicus Therapeutics	1	1	1	3
Amryt	0	1	1	2
Astellas	1	1	1	3
AstraZeneca	1	1	1	3
Bayer	1	1	1	3
Biogen	1	1	1	3
BioMarin	1	1	1	3
BlueBirdBio	0	0	1	1
Boehringer Ingelheim	1	1	1	3
BMS	1	1	1	3
Britannia	1	1	1	3
Camurus	0	0	1	1
Celgene	1	1	1	3
Chiesi	1	1	1	3
Chugai	1	1	1	3
Clinuvel	0	0	1	1
CSL Behring	1	1	1	3
Daiichi Sankyo	1	1	1	3
Diurnal	0	0	1	1
Dr Falk Pharma UK	1	1	1	3
Eisai	1	1	1	3
EliLilly	1	1	1	3
Ever Pharma	0	1	1	2
GSK	0	0	1	1
Grünenthal	0	0	1	1
GW Pharma	0	1	1	2
Immedica	0	0	1	1
Indivior	0	0	1	1
Intercept Pharma	1	1	1	3
Janssen	1	1	1	3
Jazz Pharma	0	0	1	1
LEO Pharma	1	1	1	3
Lundbeck	0	1	1	2
Lupin Healthcare	0	0	1	1
Merck	1	1	1	3
MSD	1	1	1	3

For peer review only

**Merz Pharma**	1	1	1	3
**Napp Pharma**	1	1	1	3
**Norgine**	1	1	1	3
**Novartis**	1	1	1	3
**Novo Nordisk**	1	1	1	3
**Otsuka Pharma**	1	1	1	3
**Pfizer**	1	1	1	3
**Pierre Fabre**	0	1	1	2
**PTC Therapeutics**	0	0	1	1
**Recordati**	1	1	1	3
**Rosemont Pharma**	0	0	1	1
**Sanofi Aventis**	1	1	1	3
**Santen**	1	1	1	3
**Seqirus**	1	1	1	3
**Servier Laboratories**	1	1	1	3
**Shire**	0	1	1	2
**Sobi**	0	1	1	2
**Takeda**	1	1	1	3
**Tillotts**	0	0	1	1
**UCB Pharma**	1	1	1	3
**Valneva**	1	1	1	3
**Vifor**	1	1	1	3
**Zogenix**	0	0	1	1

Notes: Please note that the table above includes the list of all companies whose disclosure reports were analysed, regardless of whether their payments were included in the analysis or not.

Table 4. Value of included payments by company and year

Company	2018	2019	2020	3 years of complete data
**Abbvie**		£441,596.70	£371,502.90	0
**Alexion**	£82,861.81	£58,253.76	£168,925.00	1
**Almirall**		£2,034.00	£9,775.00	0
**Alnylam**	£12,565.37	£55,858.20	£51,559.00	1
**Amgen**	£477,826.70	£420,997.30	£347,757.00	1
**Amryt**		£6,635.93	£45,412.77	0
**Astellas**	£54,440.01	£74,241.00	£94,583.00	1
**AstraZeneca**	£234,564.10	£431,878.80	£326,201.00	1
**BMS**	£373,025.40	£497,369.10	£517,081.80	1
**Bayer**	£263,950.80	£182,510.80	£171,758.00	1
**BioMarin**		£246,543.20	£411,912.00	0
**Biogen**	£366,326.70	£181,532.60	£663,141.80	1
**BlueBird**			£94,000.00	0
**Boehringer Ingelheim**	£141,615.90	£98,230.17	£79,762.15	1
**Britannia**	£47,763.40	£15,683.16	£35,000.00	1
**CSL Behring**	£107,455.30	£253,944.90	£152,192.00	1
**Camurus**			£13,168.40	0
**Celgene**	£683,943.50	£403,683.40	£310,329.00	1

For peer review only - http://bmjopen.bmj.com/site/about/guidelines.xhtml

**Chiesi**	£574,635.90	£600,908.70	£602,259.00	1
**Chugai**	£27,476.80	£127,523.70	£62,092.00	1
**Clinuvel**			£1,000.00	0
**Daiichi Sankyo**	£11,298.87	£29,427.36	£57,879.46	1
**Diurnal**			£6,000.00	0
**Eisai**	£13,333.69	£89,826.69	£476,271.00	1
**Eli Lilly**	£313,108.10	£299,519.70	£874,288.00	1
**Ever**		£13,195.87	£18,933.61	0
**GSK**			£325,410.00	0
**GW**		£8,898.75	£98,788.00	0
**Grünenthal**			£4,200.00	0
**Immedica**			£19,954.00	0
**Indivior**			£1,200.00	0
**Intercept**	£80,498.28	£79,988.07	£71,711.50	1
**Janssen**	£383,157.00	£780,235.30	£1,170,768.00	1
**LEO**	£48,362.25	£69,635.01	£78,633.00	1
**Lundbeck**		£325.44	£89,400.00	0
**Lupin**			£24,000.00	0
**MSD**	£455,992.00	£296,647.70	£537,631.80	1
**Merck**	£386,664.70	£306,852.60	£763,885.00	1
**Merz**	£6,091.12	£1,645.51	£31,114.00	1
**Napp**	£19,644.63	£4,240.89	£8,000.00	1
**Novartis**	£1,096,753.00	£983,145.00	£1,442,037.00	1
**Novo Nordisk**	£379,440.70	£569,074.40	£452,113.20	1
**PTC**			£151,433.00	0
**Pfizer**	£1,007,704.00	£1,092,337.00	£1,360,510.00	1
**Pierre Fabre**		£4,652.02	£50,010.00	0
**Recordati**	£2,567.93	£13,932.90	£14,500.00	1
**Roche**	£602,260.60	£368,736.60	£1,169,578.00	1
**Rosemont**			£200.00	0
**Sanofi**	£1,426,376.00	£1,939,009.00	£1,262,802.00	1
**Santen**	£14,736.81	£13,800.69	£38,170.00	1
**Seqirus**	£162,049.20	£157,635.00	£105,000.00	1
**Servier**	£7,098.03	£55,834.20	£17,162.87	1
**Shire**		£23,970.69	£555,244.40	0
**Sobi**		£194,693.30	£132,988.00	0
**Takeda**	£412,112.60	£361,158.90	£420,548.50	1
**Tillotts**			£830.00	0
**UCB**	£493,715.70	£912,466.50	£1,493,896.00	1
**Valneva**	£56,573.44	£82,380.05	£59,512.00	1
**Vifor**	£105,724.50	£193,389.20	£58,082.50	1
**Zogenix**			£43,625.00	0
**N**	37	47	60	37
**Payment Reporting Companies - All Years/At Least One Payment (%)**	61% (37/60)	78% (47/60)	100% (60/60)	61% (37/60)

For peer review only - http://bmjopen.bmj.com/site/about/guidelines.xhtml

**Value of Companies Payments - All Years/At Least One Payment (%)**	100% (£10.9 /£10.9 mln)	93% (£12.1/13 mln)	86% (£15.5/£18 mln)	100% (£10.9 /£10.9 mln)

Notes: This table displays the total included payments by company in 2020 GBP. Empty cells indicate a company/year for which no disclosure report was found. The *N* row indicates the number of companies reporting payment in each year included in the analysis. The row *Payment Reporting Companies - All Years/At Least One Payment (%)* shows the percentage of companies that disclosed payments in a given year out of the total number of companies that disclosed at least one payment across all years. For example, in 2019, 47 out of 60 companies disclosed a payment (78%). The final row, *Value of Companies Payments - All Years/At Least One Payment (%)*, indicates the percentage of the value of payments from companies reporting payment consistently across all years over the value of payments from companies reporting at least one payment. For example, in 2019, payments from companies that disclosed consistently across the study period amounted to £12,103,534 compared to £13,046,079 when any payment disclosed in that year is considered (93%).

For peer review only

Table 5. Companies' commercial interests by ICD-11 codes

	ICD-11																				
**Company**	**01**	**02**	**03**	**04**	**05**	**06**	**07**	**08**	**09**	**11**	**12**	**13**	**14**	**15**	**16**	**18**	**19**	**20**	**21**	**22**	**Other**
**Abbvie**	1	1	1	0	0	1	0	1	0	0	0	1	1	1	0	0	1	1	0	0	0
**Alexion**	0	0	1	0	1	0	0	1	0	0	0	0	0	0	0	0	0	0	0	0	1
**Almirall**	0	0	0	0	0	0	0	0	0	0	0	0	1	0	0	0	0	0	0	0	0
**Alnylam**	0	0	0	0	1	0	0	0	0	1	0	0	0	0	0	0	0	0	0	0	1
**Amgen**	0	1	1	0	1	0	0	0	0	1	0	1	1	1	1	0	0	0	0	0	1
**Amryt**	0	0	0	0	1	0	0	0	0	1	0	0	1	0	0	0	0	0	0	0	1
**Astellas**	0	1	1	0	0	0	0	0	0	0	0	0	0	0	1	0	0	0	0	0	1
**AstraZeneca**	0	1	1	0	1	0	0	0	0	1	1	0	0	0	1	0	0	0	0	0	0
**BMS**	0	1	1	0	0	1	0	1	0	1	1	1	0	1	1	0	0	0	0	0	0
**Bayer**	0	1	1	0	0	0	0	0	1	1	0	1	0	0	1	0	0	0	0	0	0
**BioMarin**	0	0	1	1	1	1	0	0	0	0	0	0	0	0	0	0	0	0	0	0	1
**Biogen**	0	0	0	0	0	0	0	1	1	0	0	0	0	1	0	0	0	0	0	0	1
**BlueBird**	0	0	1	0	1	0	0	1	0	0	0	0	0	0	0	0	0	0	0	0	1
**Boehringer Ingelheim**	0	1	0	1	1	0	0	0	0	1	1	0	0	0	0	0	0	0	0	0	0
**Britannia**	0	0	0	0	0	1	0	1	0	0	0	0	0	0	0	0	0	0	0	0	1
**CSL Behring**	1	0	1	1	0	0	0	0	0	0	0	0	0	0	0	0	0	0	0	0	0
**Camurus**	0	0	0	0	0	1	0	0	0	0	0	0	0	0	0	0	0	0	0	0	1
**Celgene**	0	1	1	0	0	0	0	1	0	0	0	1	1	1	0	0	0	0	0	0	0
**Chiesi**	0	0	1	0	1	0	0	0	1	1	1	0	0	0	1	0	1	0	0	0	1
**Chugai**	0	0	1	0	0	0	0	1	0	0	0	0	0	1	0	0	0	0	0	0	0
**Clinuvel**	0	0	0	0	1	0	0	0	0	0	0	0	0	0	0	0	0	0	0	0	0
**Daiichi Sankyo**	0	0	1	0	0	0	0	0	0	1	0	0	0	0	0	0	0	0	0	0	0
**Diurnal**	0	0	0	0	1	0	0	0	0	0	0	0	0	0	0	0	0	0	0	0	0
**Eisai**	0	1	0	0	0	1	0	1	0	0	0	1	0	0	0	0	0	0	0	0	1
**Eli Lilly**	0	1	0	0	1	0	0	1	1	0	0	1	1	1	0	0	0	0	0	0	0

For peer review only - http://bmjopen.bmj.com/site/about/guidelines.xhtml

**Ever**	0	0	0	0	0	1	0	1	0	0	0	0	0	0	0	0	0	0	0	0	1
**GSK**	1	1	1	1	0	1	0	1	0	1	1	0	0	0	0	0	0	0	0	0	0
**GW**	0	1	0	0	0	0	0	1	0	0	0	0	0	0	0	0	0	1	0	0	0
**Grünenthal**	0	0	0	0	0	0	0	0	0	0	0	0	0	1	0	0	0	0	0	0	1
**Immedica**	0	1	0	0	1	0	0	0	0	0	0	0	0	0	0	0	0	0	0	0	0
**Indivior**	0	0	0	0	0	0	0	0	0	0	0	0	0	0	0	0	0	0	0	0	1
**Intercept**	0	0	0	0	0	0	0	0	0	0	0	1	0	0	0	0	0	0	0	0	0
**Janssen**	1	1	1	1	1	1	0	0	0	1	1	1	1	1	1	0	0	0	0	0	1
**LEO**	0	0	1	0	0	0	0	0	0	1	0	0	1	0	0	0	0	1	0	0	0
**Lundbeck**	0	0	0	0	0	1	0	1	0	0	0	0	0	0	0	0	0	0	0	0	0
**Lupin**	0	0	0	0	0	0	0	1	0	0	0	0	0	0	0	0	0	0	0	0	1
**MSD**	1	1	0	0	0	0	0	1	1	1	0	0	0	0	0	0	0	0	0	0	1
**Merck**	0	1	0	0	1	0	0	1	0	0	0	0	0	0	1	1	0	1	0	0	1
**Merz**	0	0	0	0	0	0	0	1	0	1	0	0	0	0	0	0	0	0	0	0	1
**Napp**	0	1	0	0	0	0	0	0	0	0	0	1	0	0	0	0	0	0	0	0	1
**Novartis**	0	1	1	1	1	0	0	1	1	1	1	0	1	1	1	0	0	1	0	0	1
**Novo Nordisk**	0	0	1	0	1	0	0	0	0	1	0	0	0	0	0	0	0	1	0	0	0
**PTC**	0	0	0	0	0	0	0	1	0	0	0	0	0	0	0	0	0	0	0	0	1
**Pfizer**	1	1	1	1	1	0	0	1	1	1	1	1	1	1	1	0	0	0	1	0	1
**Pierre Fabre**	0	1	0	0	0	0	0	0	0	0	0	0	1	0	0	0	0	0	0	0	0
**Recordati**	0	1	0	0	1	0	0	0	0	0	0	0	0	0	0	0	0	0	0	0	0
**Roche**	0	1	1	1	0	1	0	1	1	1	1	1	0	1	0	1	0	1	0	0	1
**Rosemont**	0	0	0	0	0	0	0	0	0	0	0	0	0	0	0	0	0	0	0	0	0
**Sanofi**	1	1	1	1	1	1	0	1	0	1	1	0	1	1	1	0	0	1	0	0	1
**Santen**	0	0	0	1	0	0	0	0	1	0	0	0	0	0	0	0	0	0	0	0	0
**Seqirus**	0	0	0	0	0	0	0	0	0	0	0	0	0	0	0	0	0	0	0	0	1
**Servier**	0	1	1	0	0	0	0	0	0	1	0	0	0	0	0	0	0	0	0	0	0
**Shire**	0	0	1	1	1	1	0	0	0	0	0	0	0	0	0	0	0	0	0	0	1
**Sobi**	0	1	1	0	1	0	0	0	0	0	0	0	0	0	0	0	0	0	0	0	1
**Takeda**	0	1	1	0	1	1	0	0	0	0	0	1	1	0	0	0	0	0	0	0	0

For peer review only

**Tillotts**	0	0	0	0	0	0	0	0	0	0	0	1	0	0	0	0	0	0	0	0	0
**UCB**	0	0	1	0	0	0	0	1	0	0	0	0	1	1	1	0	0	0	0	0	1
**Valneva**	1	0	0	0	0	0	0	0	0	0	0	0	0	0	0	0	0	0	0	0	0
**Vifor**	0	0	0	1	0	0	0	0	0	1	0	0	1	0	1	0	0	0	0	0	0
**Zogenix**	0	0	0	0	0	0	0	1	0	0	0	0	0	0	0	0	0	0	0	0	0

Notes: This table reflects whether companies had a definite or probable interest in the ICD-11 code based on their pipeline or portfolio (1 = yes, 0 = no). Please note that companies' interests were opportunistically screened only in disease areas where they made a payment to a specific patient organisation, and therefore this table should not be considered exhaustive. The table refers to all years included in the analysis (2018-2020).

Legend: 01 Certain infectious or parasitic diseases; 02 Neoplasms; 03 Diseases of the blood or blood-forming organs; 04 Diseases of the immune system; 05 Endocrine, nutritional or metabolic diseases; 06 Mental, behavioural or neurodevelopmental disorders; 07 Sleep-wake disorders; 08 Diseases of the nervous system; 09 Diseases of the visual system; 11 Diseases of the circulatory system; 12 Diseases of the respiratory system; 13 Diseases of the digestive system; 14 Diseases of the skin; 15 Diseases of the musculoskeletal system or connective tissue; 16 Diseases of the genitourinary system; 18 Pregnancy, childbirth or the puerperium; 19 Certain conditions originating in the perinatal period; 20 Developmental anomalies; 21 Symptoms, signs or clinical findings, not elsewhere classified; 22 Injury, poisoning or certain other consequences of external causes; Other. Other indicates disease areas where patient organisations operate that could not be classified as any ICD-11 codes.

For peer review only

Table 6. List of patient organisations receiving payments between 2018-2020

Standardised name	Charity number	Link
Acacia Mews Care Home	1174346	https://www.nhs.uk/services/Careproviders/Overview/DefaultView.aspx?id=47011
Action Bladder Cancer UK	1164374	https://actionbladdercanceruk.org/
Action for Myalgic Encephalomyelitis	1036419	https://www.actionforme.org.uk/
Action for Pulmonary Fibrosis	1152399	https://www.actionpf.org/
Action On Pre-Eclampsia	1013557	https://action-on-pre-eclampsia.org.uk/
Action on Smoking and Health - Wales	1120834	https://ash.wales/
Action Duchenne	1101971	https://www.actionduchenne.org/
Adfam	1067428	https://adfam.org.uk/
ADHD Foundation	1120898	https://adhdfoundation.org.uk/
ADHD Norfolk	1177126	https://www.adhdnorfolk.org.uk/
Africa Advocacy Foundation	1164778	https://www.africadvocacy.org/
African-Caribbean Leukaemia Trust	1119516	https://aclt.org/
Age UK	1128267	https://www.ageuk.org.uk/
Alex - The Leukodystrophy Charity	1106008	https://www.alextlc.org/
Alex's Wish	1148845	https://alexswish.co.uk/
ALK Positive Lung Cancer	1181171	https://www.alkpositive.org.uk/
Alkaptonuria Society	1101052	https://akusociety.org/
Allergy UK	1094231	https://www.allergyuk.org/
Alport UK	1154774	http://www.alportuk.org/
Alzheimer’s Society	296645	https://www.alzheimers.org.uk/
Alzheimer’s Support	1048314	https://www.alzheimerswiltshire.org.uk/
Alzheimer's Research UK	1077089	https://www.alzheimersresearchuk.org/
Alzheimer's Society	296645	https://www.alzheimers.org.uk/
Anaemia Nurse Specialist Association	1183384	https://anaemianurse.org/home/about/
Anglo Dutch Migraine Association	1044398	https://www.anglodutchmigraine.org/
Anthony Nolan	803716	https://www.anthonynolan.org/
Anticoagulation UK	1090250	https://register-of-charities.charitycommission.gov.uk/charity-details/?regid=1090250&subid=0
AOFAC Foundation	1162155	https://aofacfoundation.org/
Aplastic Anaemia Trust	1107539	https://www.theaat.org.uk/
APS Support UK	1138116	https://aps-support.org.uk/
Arrhythmia Alliance	1107496	https://www.heartrhythmalliance.org/aa/uk
Arthritis and Musculoskeletal Alliance	1108851	http://arma.uk.net/
Aspens	1171446	https://www.aspens.org.uk/
Association for Glycogen Storage Disease	1132271	https://agsd.org.uk/
Association for Multiple Endocrine Neoplasia Disorders	1153890	https://www.amend.org.uk/

For peer review only - http://bmjopen.bmj.com/site/about/guidelines.xhtml

Asthma + Lung UK	326730	https://www.asthma.org.uk/
Astriid	1176645	https://astriid.org/
Atrial Fibrillation Association	1122442	https://www.heartrhythmalliance.org/afa/uk/
Autistica	1107350	https://www.autistica.org.uk/
Axial Spondylitis International Federation	1173902	https://asif.info/
Baby Lifeline	1006457	https://www.babylifeline.org.uk/
Barrett's Oesophagus UK	1127495	https://register-of-charities.charitycommission.gov.uk/charity-search/-/charity-details/4043374
Bath Institute for Rheumatic Diseases	1040650	https://www.birdbath.org.uk/
Batten Disease Family Association	1084908	http://www.bdfa-uk.org.uk/
Bike the UK for MS	1172717	https://biketheukforms.org/
Bipolar UK	293340	https://www.bipolaruk.org/
Bladder Health UK	1149973	https://bladderhealthuk.org/
Bliss	1002973	https://www.bliss.org.uk/
Blood Cancer UK	216032	https://bloodcancer.org.uk/
BME Cancer Communities	1182806	https://www.bmecancer.com/
Bone Cancer Research Trust	1159590	https://www.bcrt.org.uk/
Bowel Cancer UK	1071038	https://www.bowelcanceruk.org.uk/
Brain Tumour Charity	1150054	https://www.thebraintumourcharity.org/
Brain Tumour Research	1153487	https://www.braintumourresearch.org/
Brain Tumour Support	1163856	https://www.braintumoursupport.co.uk/
Brains Trust	1114634	https://brainstrust.org.uk/
Breast Cancer Haven (The Haven)	1061726	https://www.breastcancerhaven.org.uk/
Breast Cancer Now	1160558	https://breastcancernow.org/
Bristol & Weston Hospitals Charity	1170973	https://www.bwhospitalscharity.org.uk/
British Association for Sexual Health & HIV	1148196	https://www.bashh.org/
British Association for the Study of the Liver	1106320	https://www.basl.org.uk/
British Geriatric Society	268762	https://www.bgs.org.uk/
British Heart Foundation	225971	https://www.bhf.org.uk/
British Infertility Counselling Association	803743	https://www.bica.net/
British Inherited Metabolic Disease Group	1184024	https://www.bimdg.org.uk/site/index.asp
British Liver Trust	298858	https://britishlivertrust.org.uk/
British Paediatric Neurology Association	1159115	https://bpna.org.uk/
British Porphyria Association	1089609	http://porphyria.org.uk/
British Sarcoma Group	1154928	https://britishsarcomagroup.org.uk/
British Skin Foundation	1171373	https://www.britishskinfoundation.org.uk/
British Society for Heart Failure	1075720	https://www.bsh.org.uk/

For peer review only - http://bmjopen.bmj.com/site/about/guidelines.xhtml

British Society of Echocardiography	1093808	https://www.bsecho.org/
British Thyroid Foundation	1006391	https://www.btf-thyroid.org/
British Voice Association	1078709	http://www.britishvoiceassociation.org.uk/
Brittle Bone Society	272100	https://www.brittlebone.org/
Cambridge Rare Disease Network	1166365	https://www.camraredisease.org/
Cancer 52	1174569	https://www.cancer52.org.uk/
Cancer Black Care	1086465	https://www.cancerblackcare.org.uk/
Cancer Research UK	1089464	https://www.cancerresearchuk.org/
Cancer Support UK	1105703	https://cancersupportuk.org/
CancerCare	1120048	https://cancercare.org.uk/
Cara Trust	328124	https://www.madtrust.org.uk/project/the-cara-trust/
Cardiac Risk in the Young	1050845	https://www.c-r-y.org.uk/
Cardiomyopathy UK	1164263	https://www.cardiomyopathy.org/
Carers Trust	1145181	https://carers.org/
Carers Worldwide	1150214	https://carersworldwide.org/
Changing Faces	1011222	https://www.changingfaces.org.uk/
Child Growth Foundation	1172807	https://childgrowthfoundation.org/
Childhood Trust	1154032	https://www.childhoodtrust.org.uk/
Children’s Cancer and Leukaemia Group	1182637	https://www.cclg.org.uk/
Children’s HIV Association	1122356	https://www.chiva.org.uk/
Children’s Trust	288018	https://www.thechildrenstrust.org.uk/
Children's Burns Trust	1082084	https://www.cbtrust.org.uk/
Cholangiocarcinoma Charity	1091915	https://ammf.org.uk/
Chronic Lymphocytic Leukaemia Support Association	1178482	https://www.cllsupport.org.uk/
Chronic Myeloid Leukaemia Support Group	1114037	https://cmlsupport.org.uk/
Coalition for Life-Course Immunisation	1182662	https://www.cl-ci.org/
Confederation of Meningitis Organisations	1091105	https://www.comomeningitis.org/
Congenital Adrenal Hyperplasia Support Group	1178951	https://geneticalliance.org.uk/member/congenital-adrenal-hyperplasia-support-group/
Contact a Family	284912	https://contact.org.uk/
Crohn's and Colitis UK	1117148	https://www.crohnsandcolitis.org.uk/
Crohn's In Childhood Research Association	278212	https://www.cicra.org/
Cure Leukaemia	1100154	https://www.cureleukaemia.co.uk/
Cystic Fibrosis Trust	1079049	https://www.cysticfibrosis.org.uk/
Delete Blood Cancer	1150056	https://www.dkms.org.uk/
Dementia UK	1039404	https://www.dementiauk.org/
Dementia Club UK	1168397	https://dementiaclubuk.org.uk/
Diabetes UK	215199	https://www.diabetes.org.uk/

For peer review only - http://bmjopen.bmj.com/site/about/guidelines.xhtml

Diabetes UK - Northern Ireland	215199	https://www.diabetes.org.uk/in_your_area/n_ireland
Diana Award	1117288	https://diana-award.org.uk/
Different Strokes	1092168	https://differentstrokes.co.uk/
Disasters Emergency Committee	1062638	https://www.dec.org.uk/
DMD Pathfinders	1155884	https://www.pathfindersalliance.org.uk/
Donor Conception Network	1041297	https://www.dcnetwork.org/
Down Syndrome International	1091843	https://www.ds-int.org/
Downs Syndrome Association	1061474	https://www.downs-syndrome.org.uk/
Dravet Syndrome UK	1128289	https://www.dravet.org.uk/
DrugFAM	1123316	https://www.drugfam.co.uk/#
Duchenne UK	1147094	https://www.duchenneuk.org/
Dystonia UK	1062595	https://www.dystonia.org.uk/
East North Hertfordshire NHS Trust	1053338	https://www.enherts-tr.nhs.uk/
East Sussex Healthcare NHS Trust	1058599	https://www.esht.nhs.uk/
Ecancer	1176307	https://ecancer.org/en/
Encephalitis Society	1087843	https://www.encephalitis.info/
Endometriosis UK	1035810	https://www.endometriosis-uk.org/
Epilepsy Action	234343	https://www.epilepsy.org.uk/
Epilepsy Research UK	1100394	https://epilepsyresearch.org.uk/
Epilepsy Society	206186	https://epilepsysociety.org.uk/
Errol Mckellar Foundation	1181574	https://www.theerrolmckellarfoundation.com/
European Association for the Study of Obesity	1111288	https://easo.org/
European Parkinson’s Disease Association	1163211	https://www.epda.eu.com/
Eve Appeal	1091708	https://eveappeal.org.uk/
Familial Hypercholesterolaemia Network	1170731	https://fheurope.org/
FareShare	1100051	https://fareshare.org.uk/
Fertility Network UK	1099960	https://fertilitynetworkuk.org/
Fight Bladder Cancer	1157763	https://www.fightbladdercancer.co.uk/
Fight for Sight UK	1111438	https://www.fightforsight.org.uk/
Findacure / Beacon for rare diseases LTD	1149646	https://www.rarebeacon.org/about-us/our-journey/
Fungal Infection Trust	1147658	https://fungalinfectiontrust.org/
Gauchers Association	1095657	https://www.gaucher.org.uk/
Gene People	1141583	https://genepeople.org.uk/
Genetic Alliance UK	1114195	https://geneticalliance.org.uk/
GIST Cancer UK	1129219	https://www.gistcancer.org.uk/
GIST Support UK	1129219	https://geneticalliance.org.uk/member/gist-support-uk/
Global Action on Men's Health	1183428	https://gamh.org/
GO Girls	1179108	https://www.gogirlssupport.org/

For peer review only - http://bmjopen.bmj.com/site/about/guidelines.xhtml

Gorlin Syndrome Group	1197282	https://gorlingroup.org/
Guts UK	1137029	https://gutscharity.org.uk/
Haemachromatosis UK	1001307	https://www.haemochromatosis.org.uk/
Haemophilia Society	288260	https://haemophilia.org.uk/
Haemophilia Wales	1158941	https://haemophiliawales.org/
Harefield Hamsters Transplant Club	1060656	https://harefieldhamsters.org/
Head & Neck Cancer UK	1175181	https://hancuk.org/
Headway East London	1083910	https://headwayeastlondon.org/
Heart UK	1003904	https://www.heartuk.org.uk/
Heartburn Cancer UK	1136413	https://www.heartburncanceruk.org/
Helen & Douglas House	1085951	https://www.helenanddouglas.org.uk/
Helping Overcome Obesity Problems	1150683	http://hoopuk.org.uk/
Hepatitis C Trust	1104279	http://hepctrust.org.uk/
Hereditary Angioedema UK	1152591	https://www.haeuk.org/
Hidradenitis Suppurativa Trust	1177819	https://painuk.org/members/charities/hidradenitis-suppurativa-trust/
Histiocytosis UK	1158789	https://www.histiouk.org/
HIV i-Base	1081905	https://i-base.info/
Home-Start Hampshire	1144661	https://home-starthampshire.org.uk/
Hope for Tomorrow	1094677	https://hopefortomorrow.org.uk/
Human Story Theatre	1173504	https://humanstorytheatre.com/about-us/
Huntington's Disease Association	296453	https://www.hda.org.uk/
Huntington's Disease Youth Organization	1145781	https://en.hdyo.org/
IBD Passport	1171268	https://www.ibdpassport.com/
Ichthyosis Support Group	1142457	https://www.ichthyosis.org.uk/
Immune Thrombocytopenia Support Association	1064480	https://www.itpsupport.org.uk/index.php/en/
Independent Cancer Patients' Voice	1138456	http://www.independentcancerpatientsvoice.org.uk/
Intensive Care Society	1039236	https://www.ics.ac.uk/
International Alliance of Patients' Organizations	1155577	https://www.iapo.org.uk/
International Chronic Myeloid Leukemia Foundation	1132984	https://www.cml-foundation.org/
International Gaucher Alliance	1192011	https://gaucheralliance.org/home
International Headache Society	1042574	https://ihs-headache.org/en/
International Longevity Centre UK	1080496	https://ilcuk.org.uk/
International Niemann-Pick Disease Alliance	1150256	https://www.inpda.org/
International Niemann-Pick Disease Registry	1175311	https://inpdr.org/
International Patient Organisation for Primary Immunodeficiencies	1058005	https://ipopi.org/
Isabel Hospice Limited	1046826	https://www.isabelhospice.org.uk/

For peer review only - http://bmjopen.bmj.com/site/about/guidelines.xhtml

Isle of Wight Diabetic Fund	298933	https://www.charitychoice.co.uk/isle-of-wight-diabetic-fund-142014
Jo's Cervical Cancer Trust	1133542	https://www.jostrust.org.uk/
Juvenile Diabetes Research Foundation	295716	https://jdrf.org.uk/
Karen Clifford Skcin cancer charity	1150048	https://www.skcin.org/
Katie Piper Foundation	1133313	https://katiepiperfoundation.org.uk/
Kent Autistic Trust	801965	https://www.kentautistictrust.org/
Kent MS Therapy Centre	801382	https://kentmstc.org.uk/
Kidney Cancer Support Network	1164238	https://actionkidneycancer.org/
Kidney Cancer UK	1120146	https://www.kcuk.org.uk/
Kidney Care UK	270288	https://www.kidneycareuk.org/
Kidney Research UK	252892	https://www.kidneyresearchuk.org/
Legs Matter	1180844	https://legsmatter.org/
Leukaemia CARE	1183890	https://www.leukaemiacare.org.uk/
Leukaemia UK	1154856	https://www.leukaemiauk.org.uk/
Lipodystrophy UK	1175462	https://register-of-charities.charitycommission.gov.uk/charity-search/-/charity-details/5111931
Liver4Life	1152618	https://www.liver4life.org.uk/
LIVErNORTH	1087226	http://www.livernorth.org.uk/
Lupus UK	1051610	https://www.lupusuk.org.uk/
Lymphoma Action	1068395	https://lymphoma-action.org.uk/about-us
Macmillan Cancer Support	261017	https://www.macmillan.org.uk/
Marie Curie Cancer Care	207994	https://www.mariecurie.org.uk/
Mavis Nye Foundation	1172765	http://www.mavisnyefoundation.com/
Maypole Project	1120163	https://www.themaypoleproject.co.uk/
MDS UK Support Group	1145214	https://mdspatientsupport.org.uk/
Meath Epilepsy Charity	200359	https://www.meath.org.uk/
Medics 4 Rare Diseases	1183996	https://www.m4rd.org/history/
Melanoma Focus	1124716	https://melanomafocus.org/
Melanoma Fund	1085969	https://www.melanoma-fund.co.uk/
Melanoma UK	1157635	https://www.melanomauk.org.uk/
Memorylane Eastbourne	1163541	https://www.memorylaneeastbourne.co.uk/
Men’s Health Forum	1087375	https://www.menshealthforum.org.uk/
Meningitis Now	803016	https://www.meningitisnow.org/
Meningitis Research Foundation	1091105	https://www.meningitis.org/
Mental Health Foundation	801130	https://www.mentalhealth.org.uk/
Mental Health UK	1170815	https://mentalhealth-uk.org/
Mersey Region Epilepsy Association	504366	https://www.epilepsymersey.org.uk/
Mesothelioma UK	1177039	https://www.mesothelioma.uk.com/
Metabolic Support UK	1089588	https://www.metabolicsupportuk.org/
Migraine Trust	1081300	https://migrainetrust.org/
MLD Support Association UK	1150542	https://www.mldsupportuk.org.uk/

For peer review only - http://bmjopen.bmj.com/site/about/guidelines.xhtml

Motor Neurone Disease Association	294354	https://www.mndassociation.org/
Mouth Cancer Foundation	1109298	https://www.mouthcancerfoundation.org/
Multiple Sclerosis International Federation	1105321	https://www.msif.org/
Multiple Sclerosis Society UK	1139257	https://www.mssociety.org.uk/
Multiple Sclerosis Therapy Centres	1031690	https://www.msntc.org.uk/
Multiple Sclerosis Trust	1088353	https://mstrust.org.uk/
Muscular Dystrophy UK	205395	https://www.musculardystrophyuk.org/
MYFANWY TOWNSEND	1085969	https://doit.life/organisation/myfanwy-townsend-melanoma-research-fund
Narcolepsy UK	1144342	https://www.narcolepsy.org.uk/
National AIDS Map	1011220	https://www.aidsmap.com/
National AIDS Trust	297977	https://www.nat.org.uk/
National Axial Spondyloarthritis Society	1183175	https://nass.co.uk/
National Cancer Research Institute	1160609	https://www.ncri.org.uk/
National Eczema Society	1009671	https://eczema.org/
National Federation of Prostate Cancer Support Groups	1163152	https://tackleprostate.org/
National Kidney Federation	1106735	https://www.kidney.org.uk/
National Migraine Centre	1115935	https://www.nationalmigrainecentre.org.uk/
National Rheumatoid Arthritis Society	1134859	https://nras.org.uk/
National Voices	1057711	https://www.nationalvoices.org.uk/
NAZ	1014056	https://www.naz.org.uk/
Neuroendocrine Cancer UK	1092386	https://www.neuroendocrinecancer.org.uk/
Neurological Alliance	1039034	https://www.neural.org.uk/
NHS Charities Together	1186569	https://nhscharitiestogether.co.uk/
Niemann-Pick UK	1144406	https://www.npuk.org/
NMO Spectrum UK	1158104	http://www.nmouk.nhs.uk/resources-useful-links/nmo-spectrum-uk
North Bristol NHS Trust	1055900	https://www.nbt.nhs.uk/
Oliver King Foundation	1144485	https://theoliverkingfoundation.co.uk/
Oral Health Foundation	263198	https://www.dentalhealth.org/
Orchid	1080540	https://orchid-cancer.org.uk/
Osteoporosis Dorset	1023507	https://www.osteodorset.org.uk/
Ovacome	1159682	https://www.ovacome.org.uk/
Ovarian Cancer Action	1109743	https://ovarian.org.uk/
Over the Wall	1075361	https://www.otw.org.uk/
Pain UK	1191657	https://painuk.org/
Pancreatic Cancer Action	1137689	https://pancreaticcanceraction.org/
Pancreatic Cancer UK	1112708	https://www.pancreaticcancer.org.uk/
Parkinson's UK	258197	https://www.parkinsons.org.uk/
Paroxysmal Nocturnal Haemoglobinuria Support	1161518	https://pnhuk.org/

For peer review only - http://bmjopen.bmj.com/site/about/guidelines.xhtml

Patients Association	1006733	https://www.patients-association.org.uk/
Patients On Intravenous and Nasogastric Nutrition Therapy	1157655	https://pinnt.com/Home.aspx
Paula Carr Diabetes Trust	801596	https://www.paulacarrdiabetestrust.co.uk/
Pelican Cancer Foundation	1141911	https://www.pelicancancer.org/?doing_wp_cron=1645539531.1477270126342773437500
Phyllis Tuckwell Hospice	264501	https://www.pth.org.uk/
Pilgrims Hospice	293968	https://www.pilgrimshospices.org/
Pink Ribbon Foundation	1080839	https://www.pinkribbonfoundation.org.uk/
Pituitary Foundation	1058968	https://www.pituitary.org.uk/
Platelet Society	1172202	https://plateletsociety.co.uk/
Point Of Care Foundation	1151628	https://www.pointofcarefoundation.org.uk/
Polycystic Kidney Disease Charity	1160970	https://pkdcharity.org.uk/
Pompe Support Network	1186383	https://pompe.uk/
Positively UK	1007685	https://positivelyuk.org/
Prevent Breast Cancer Charity UK	1109839	https://preventbreastcancer.org.uk/
Primary Immunodeficiency UK	1193166	http://www.immunodeficiencyuk.org/
Progress Educational Trust	1139856	https://www.progress.org.uk/
Progressive Supranuclear Palsy Association	1037087	https://pspassociation.org.uk/
Prostate Cancer UK	1005541	https://prostatecanceruk.org/
Psoriasis Association	1180666	https://www.psoriasis-association.org.uk/
Pulmonary Fibrosis Trust	1149901	https://pulmonaryfibrosistrust.org/
Pulmonary Hypertension Association UK	1120756	https://www.phauk.org/
Pumping Marvellous Foundation	1151848	https://www.pumpingmarvellous.org/
Rainbow Trust Children's Charity	1070532	https://www.rainbowtrust.org.uk/
Rapid Effective Assistance For Children With Potentially Terminal Illness	802440	https://reactcharity.org/
Rare Autoinflammatory Conditions Community	1184846	https://raccuk.com/
Red Rose Recovery	1152474	https://redroserecovery.org.uk/
Reform	1103739	https://reform.uk/
Release	801118	https://www.release.org.uk/
Rethink Mental Illness	271028	https://www.rethink.org/
Retina UK	1153851	https://retinauk.org.uk/about/
Reverse Rett	1136809	https://www.reverserett.org.uk/
Ring 20 Research & Support UK	1165651	https://ring20researchsupport.co.uk/
Roald Dahl's Marvellous Children's	1137409	https://www.roalddahlcharity.org/
Roy Castle Lung Cancer Foundation	1046854	https://roycastle.org/

For peer review only - http://bmjopen.bmj.com/site/about/guidelines.xhtml

Royal Free Charity	1165672	https://royalfreecharity.org/
Royal Manchester Children's Hospital	1049274	https://mft.nhs.uk/rmch/
Royal National Institute of Blind People	226227	https://www.rnib.org.uk/
Royal Osteoporosis Society	1102712	https://theros.org.uk/
Ruth Strauss Foundation	1183221	https://ruthstraussfoundation.com/
Salivary Gland Cancer UK	1182762	https://www.salivaryglandcancer.uk/
SANE	296572	https://www.sane.org.uk/
Sarcoma UK	1139869	https://sarcoma.org.uk/
Scleroderma and Raynauds UK	1161828	https://www.sruk.co.uk/
Sexual Advice Association	1104691	https://sexualadviceassociation.co.uk/
Shift.MS	1117194	https://shift.ms/
Shine Cancer Support	1146902	https://shinecancersupport.org/
Sickle Cell and Young Stroke Survivor	1120902	http://www.scyss.org/
Sickle Cell Society	1046631	https://www.sicklecellsociety.org/
Sightsavers India	207544	https://www.sightsaversindia.in/
Skinship UK	1047108	https://skinshipuk.org/
Society for Mucopolysaccharide Diseases	1143472	https://www.mpssociety.org.uk/
Solving Kids' Cancer	1135601	https://www.solvingkidscancer.org.uk/
Somerville Foundation	1138088	https://sfhearts.org.uk/
Sophia Forum	1131629	https://sophiaforum.net/
South Asian Health Foundation	1073178	https://www.sahf.org.uk/
South of England Neuroscience Association	1198001	https://www.sena.org.uk/
Spinal Injuries Association	1054097	https://www.spinal.co.uk/
Spinal Muscular Atrophy Support UK	1106815	https://smauk.org.uk/
St Elizabeths Centre	1176777	https://www.stelizabeths.org.uk/
Stroke Association	211015	https://www.stroke.org.uk/
Swallows Head and Neck Cancer Charity	1149794	https://www.theswallows.org.uk/
Target Ovarian Cancer	1125038	https://targetovariancancer.org.uk/
Teenage Cancer Trust	1062559	https://www.teenagecancertrust.org/
Tenovus Cancer Care	1054015	https://www.tenovuscancercare.org.uk/
Terrence Higgins Trust	288527	https://www.tht.org.uk/
THE MACULAR DISEASE SOCIETY	1001198	
THE NATIONAL ASSOCIATION FOR THE RELIEF OF PAGET'S DISEASE	266071	https://paget.org.uk/
Theodora Children's Charity	1094532	https://uk.theodora.org/
Thrombosis UK	1090540	https://thrombosisuk.org/news/post.php?s=2021-10-11-thrombosis-uk-winner-of-activity-of-the-year-award-2021
Tiny Tickers	1078114	https://www.tinytickers.org/

For peer review only - http://bmjopen.bmj.com/site/about/guidelines.xhtml

Together for Short Lives		
Trekstock	1132421	https://www.trekstock.com/
Trevi	1075433	https://trevi.org.uk/
Tuberous Sclerosis Association	1039549	https://tuberous-sclerosis.org/
Turner Syndrome Support Society	1080507	https://tss.org.uk/
Twins Trust	1076478	https://twinstrust.org/
UK ATTR AMYLOIDOSIS PATIENTS' ASSOCIATION (UKATPA)	1183624	https://register-of-charities.charitycommission.gov.uk/charity-details/?regid=1183624&subid=0
UK Breast Cancer Group	1177296	https://ukbcg.org/
UK Mastocytosis Support Group	1154007	https://ukmasto.org/#gsc.tab=0
UK National Intrathecal Baclofen Trust	1129812	https://register-of-charities.charitycommission.gov.uk/charity-search/-/charity-details/4043971/full-print
UK Primary Immune-deficiency Patient Support	1148789	https://ukpips.org.uk/
UK Thalassaemia Society	275107	https://ukts.org/
University College London Hospitals Charity	1165398	https://www.uclhcharity.org.uk/
University Hospitals Coventry and Warwickshire	1165393	https://www.uhcw.nhs.uk/
Urology Cancer Research and Education	1120887	http://www.ucare-oxford.org.uk/
Vascular Society of Britain and Ireland	1102769	https://www.vascularsociety.org.uk/default.aspx
Vasculitis UK	1180473	https://www.vasculitis.org.uk/
Versus Arthritis	207711	https://www.versusarthritis.org/
Versus Arthritis UK	207711	https://www.versusarthritis.org/
Virginia Keiley Benefaction	1038091	https://givesuper.co.uk/charityDetails/1038091
Visionary	1135360	https://www.visionary.org.uk/
Waldenstrom's Macroglobulinaemia UK	1187121	https://wmuk.org.uk/
Walton Centre	1050050	https://www.thewaltoncentre.nhs.uk/
White Chapel Mission	227905	https://whitechapel.org.uk/
World Cancer Research Fund	1000739	https://www.wcrf-uk.org/
World Child Cancer	1084729	https://worldchildcancer.org/
Yorkshire Cancer Research	516898	https://yorkshirecancerresearch.org.uk/
Young Epilepsy	311877	https://www.youngepilepsy.org.uk/
Young Lives vs Cancer	1107328	https://www.younglivesvscancer.org.uk/

For peer review only - http://bmjopen.bmj.com/site/about/guidelines.xhtml

Inclusion/exclusion of patient organisations

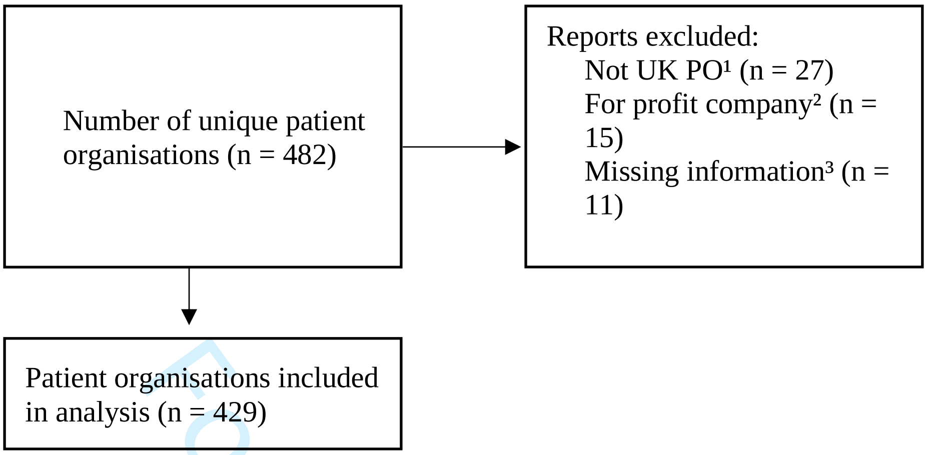

[1]Not aligned with geographical scope e.g. Irish, US-based
[2]Not aligned with EFPIA's definition of patient organisation
[3]Organisations for whose nature is unclear i.e. patient organisation website could not be identified

For peer review only

For peer review only - http://bmjopen.bmj.com/site/about/guidelines.xhtml

Additional tables and figures

Table 7. Number of funding companies, top funder and highest payment for the top five receiving patient organisations

Patient organisations	Number of funding companies	Top funder	Overall funding (in 2020 GBP)	Highest payment (in 2020 GBP)	Top funder share of overall funding*	Top funder interest†
**Rare**						
Cystic Fibrosis Trust	1	Chiesi	£ 1,305,512	£ 440,229	100%	*Definitely yes*
Myeloma UK	8	Celgene	£ 1,243,519	£ 112,988	34%	*Definitely yes*
Genetic Alliance UK	15	Alexion	£ 613,006	£ 50,325	25%	*Definitely yes*
International Patient Organisation for Primary Immunodeficiencies	5	Shire	£ 556,357	£ 221,450	40%	*Definitely yes*
Society for Mucopolysaccharide Diseases	6	Sanofi	£ 651,097	£ 91,179	45%	*Definitely yes*
**Non-rare**						
Diabetes UK	9	Novo Nordisk	£ 2,389,423	£ 207,878	45%	*Definitely yes*
Epilepsy Society	2	UCB	£ 1,539,749	£ 946,300	100%	*Definitely yes*
Shift.MS	5	Sanofi	£ 1,315,328	£ 104,607	26%	*Definitely yes*
Multiple Sclerosis International Federation	6	Sanofi	£ 1,279,214	£ 164,347	38%	*Definitely yes*
Asthma + Lung UK	11	Seqirus	£ 994,842	£ 96,759	16%	*Definitely yes*

Notes Please note that all data presented in the table refer to the overall study period, from 2018 to 2020.
*This column indicates the share of overall funding (from 2018-2020) to the relevant patient organisation from their top funder, as indicated in the third column.
†This column the interest (i.e., *Definitely yes*, *Probably yes* or *No*) the top funder of the patient organisation, as indicated in the third column.

For peer review only - http://bmjopen.bmj.com/site/about/guidelines.xhtml

Table 8. Volume and value of payments by company interests broken down rarity of diseases from 2018 to 2020

		**Volume**; n (%)			**Value**: £ (%)		
**PO type**	**Company's interest**	*2018*	*2019*	*2020*	*2018*	*2019*	*2020*
Overall†	Definitely yes	488 (53%)	554 (52%)	585 (50%)	£6,983,350 (64%)	£8,319,177 (64%)	£10,700,000 (59%)
	Probably yes	369 (40%)	425 (40%)	471 (40%)	£3,137,189 (29%)	£3,844,276 (29%)	£5,743,500 (32%)
	No*	67 (7%)	84 (8%)	112 (10%)	£813,176 (7%)	£882,627 (7%)	£1,566,402 (9%)
Rare	Definitely yes	79 (53%)	125 (58%)	136 (54%)	£1,602,340 (69%)	£2,372,533 (72%)	£2,750,425 (66%)
	Probably yes	59 (40%)	79 (38%)	124 (45%)	£635,393 (27%)	£781,688 (24%)	£1,296,449 (31%)
	No*	10 (7%)	11 (5%)	13 (5%)	£91,282 (4%)	£126,779 (4%)	£134,015 (3%)
Non-rare	Definitely yes	408 (56%)	425 (54%)	443 (55%)	£5,350,194 (67%)	£5,921,218 (65%)	£7,850,393 (62%)
	Probably yes	304 (42%)	339 (43%)	334 (41%)	£2,409,093 (31%)	£3,032,911 (33%)	£4,385,282 (35%)
	No*	17 (2%)	24 (3%)	30 (4%)	£231,784 (3%)	£155,331 (2%)	£334,352 (3%)

Notes: *Definitely yes* indicates payments directed to patient organisations that operated in a disease area (ICD-11 level 4 or higher) for which the company has a product in its portfolio or pipeline. *Probably yes* indicates directed to patient organisations that operated in a disease area (ICD-11 level 3 or lower) for which the company has a product in its portfolio or pipeline. *No* refers to directed to patient organisations that operated in a disease area for which no link could be found to the company's portfolio or pipeline. The higher the ICD-11, the more specific the condition. For example, if the ICD-11 level 4 is *Plasma cell neoplasms*, level 2 would be *Neoplasms of hematopoietic or lymphoid tissues*. Further details on how this variable was constructed can be found in the Supplemental Material.

*Please note that the *No* category of interest conservatively includes also interests that were considered as unclear.

†Please note that the *Overall* results are not a sum of the *Rare* and *Non-rare* results, as they also include patient organisations that could not be classified in either group.

For peer review only - http://bmjopen.bmj.com/site/about/guidelines.xhtml

Figure 2 Value of payments by receiving patient organisation and funding company, broken down by year

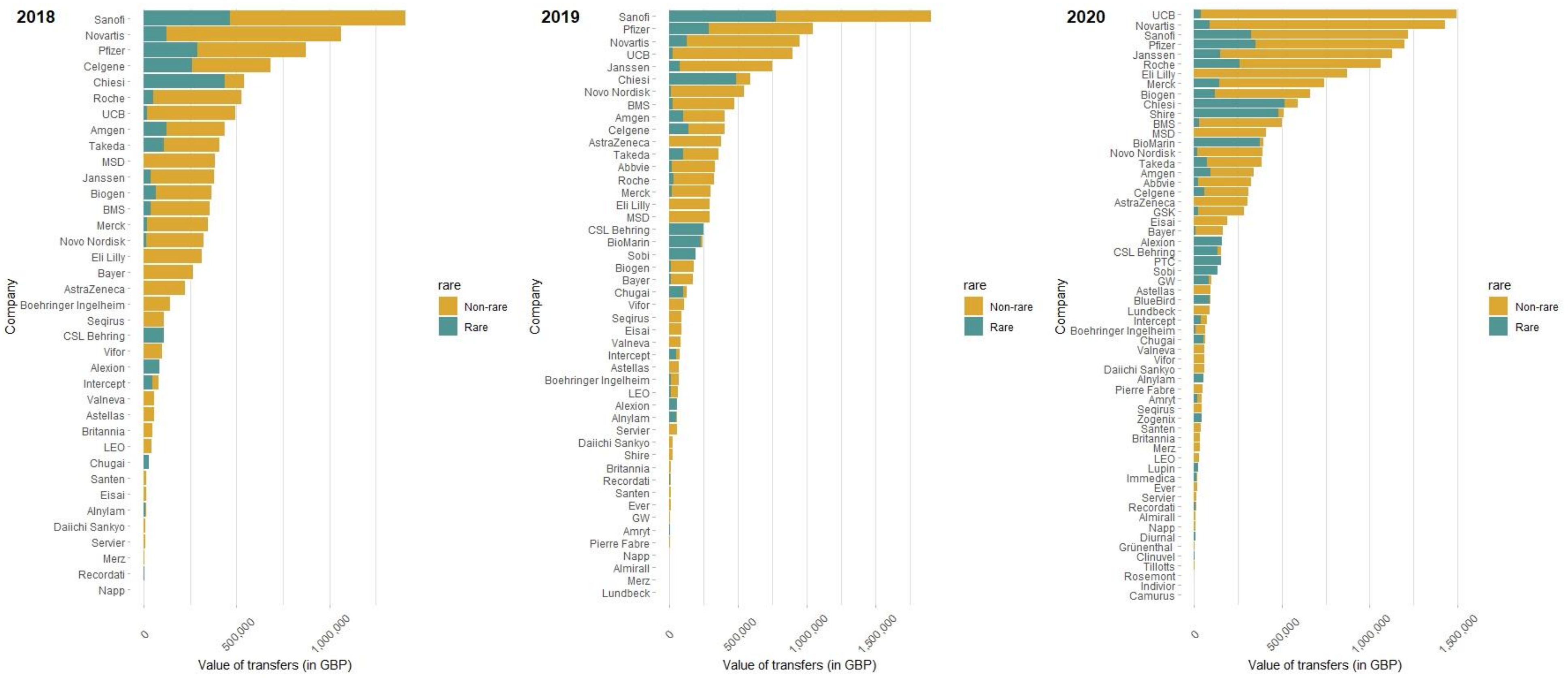

Figure 3. Value of payments by patient organisation type, therapeutic area and year

A)

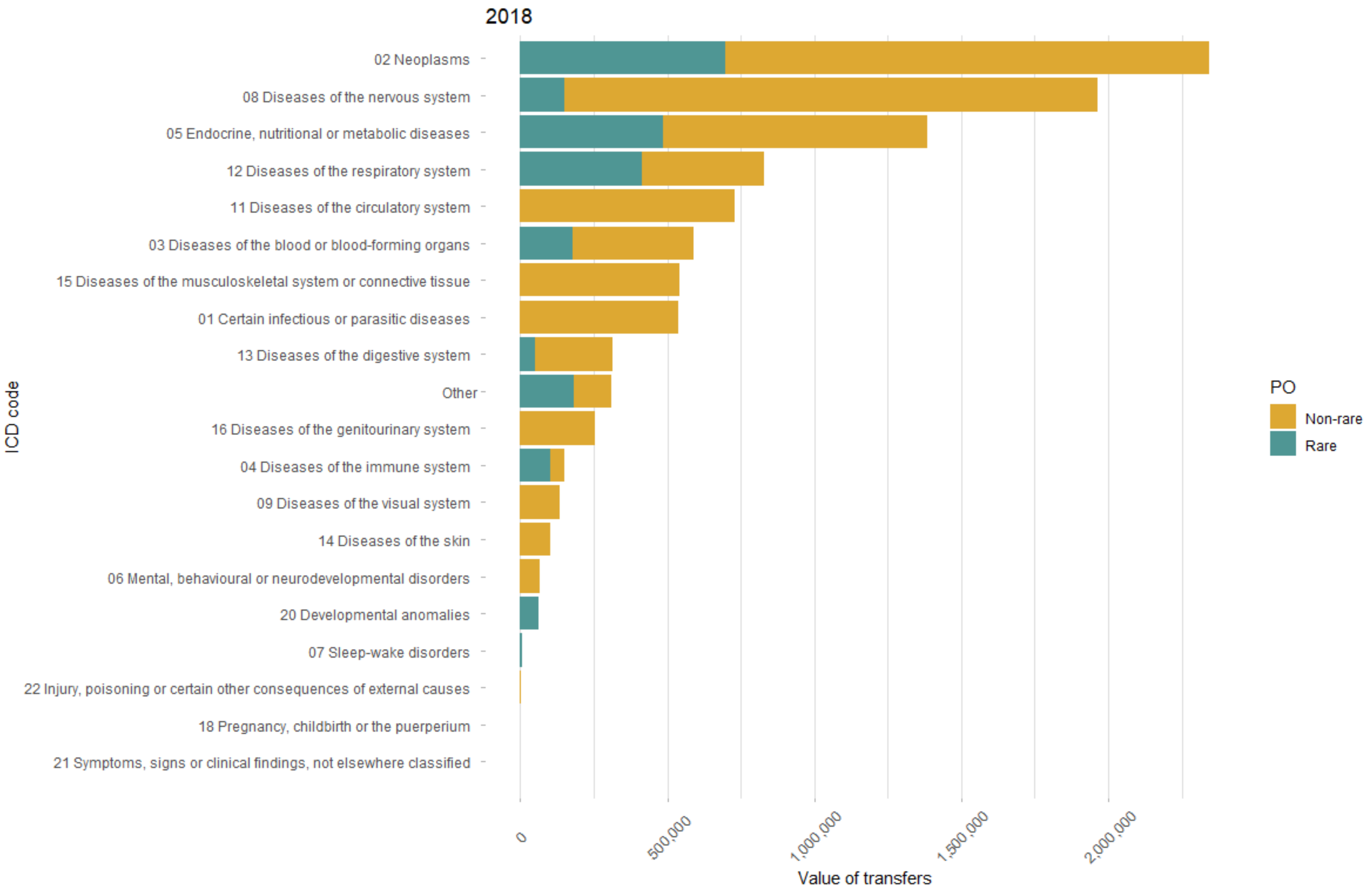

B)

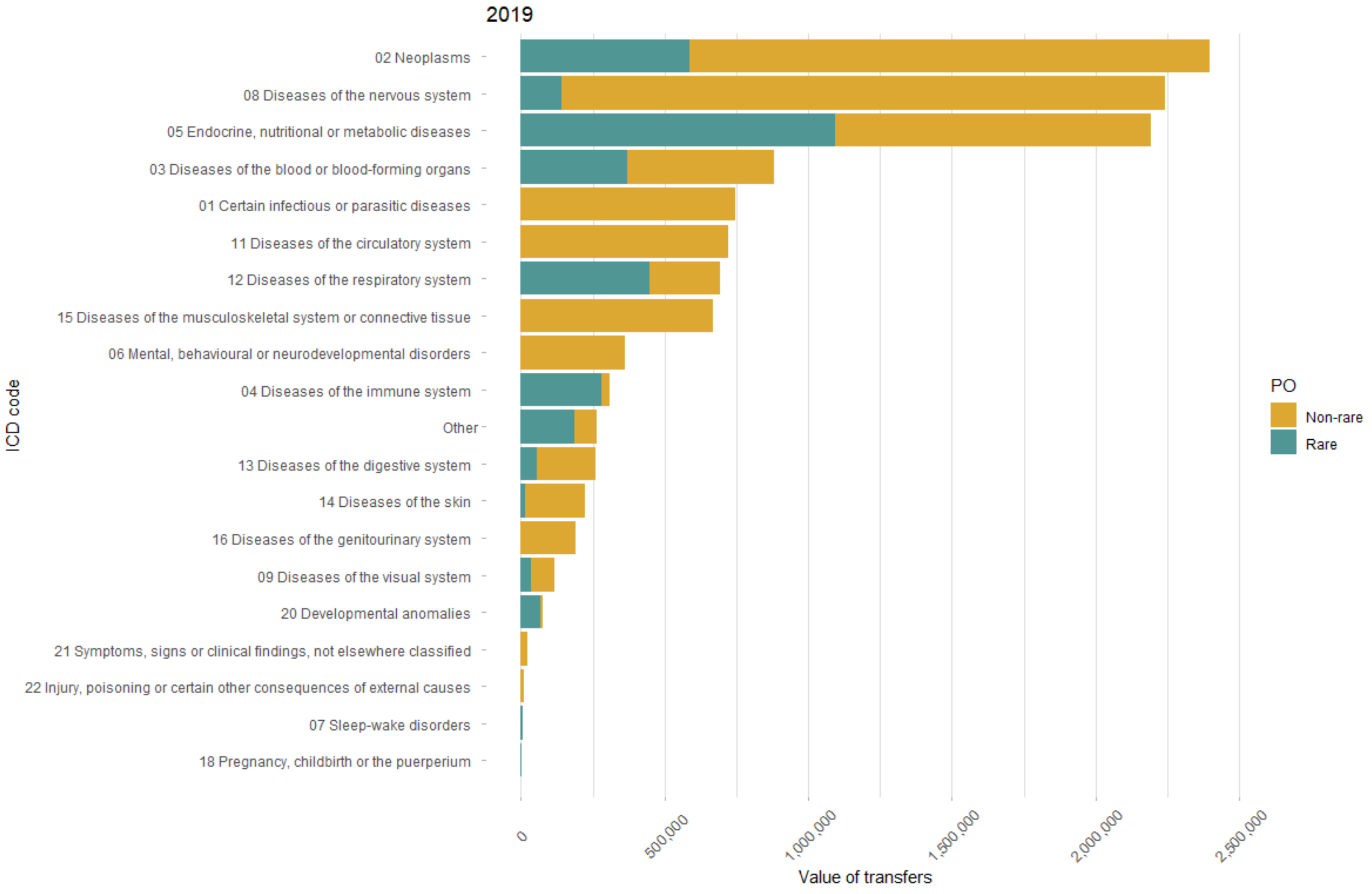
2019
02 Neoplasms
08 Diseases of the nervous system
05 Endocrine, nutritional or metabolic diseases
03 Diseases of the blood or blood-forming organs
01 Certain infectious or parasitic diseases
11 Diseases of the circulatory system
12 Diseases of the respiratory system
15 Diseases of the musculoskeletal system or connective tissue
06 Mental, behavioural or neurodevelopmental disorders
04 Diseases of the immune system
Other
13 Diseases of the digestive system
14 Diseases of the skin
16 Diseases of the genitourinary system
09 Diseases of the visual system
20 Developmental anomalies
21 Symptoms, signs or clinical findings, not elsewhere classified
22 Injury, poisoning or certain other consequences of external causes
07 Sleep-wake disorders
18 Pregnancy, childbirth or the puerperium
ICD code
0
500,000
1,000,000
1,500,000
2,000,000
2,500,000
Value of transfers
PO
Non-rare
Rare

C)

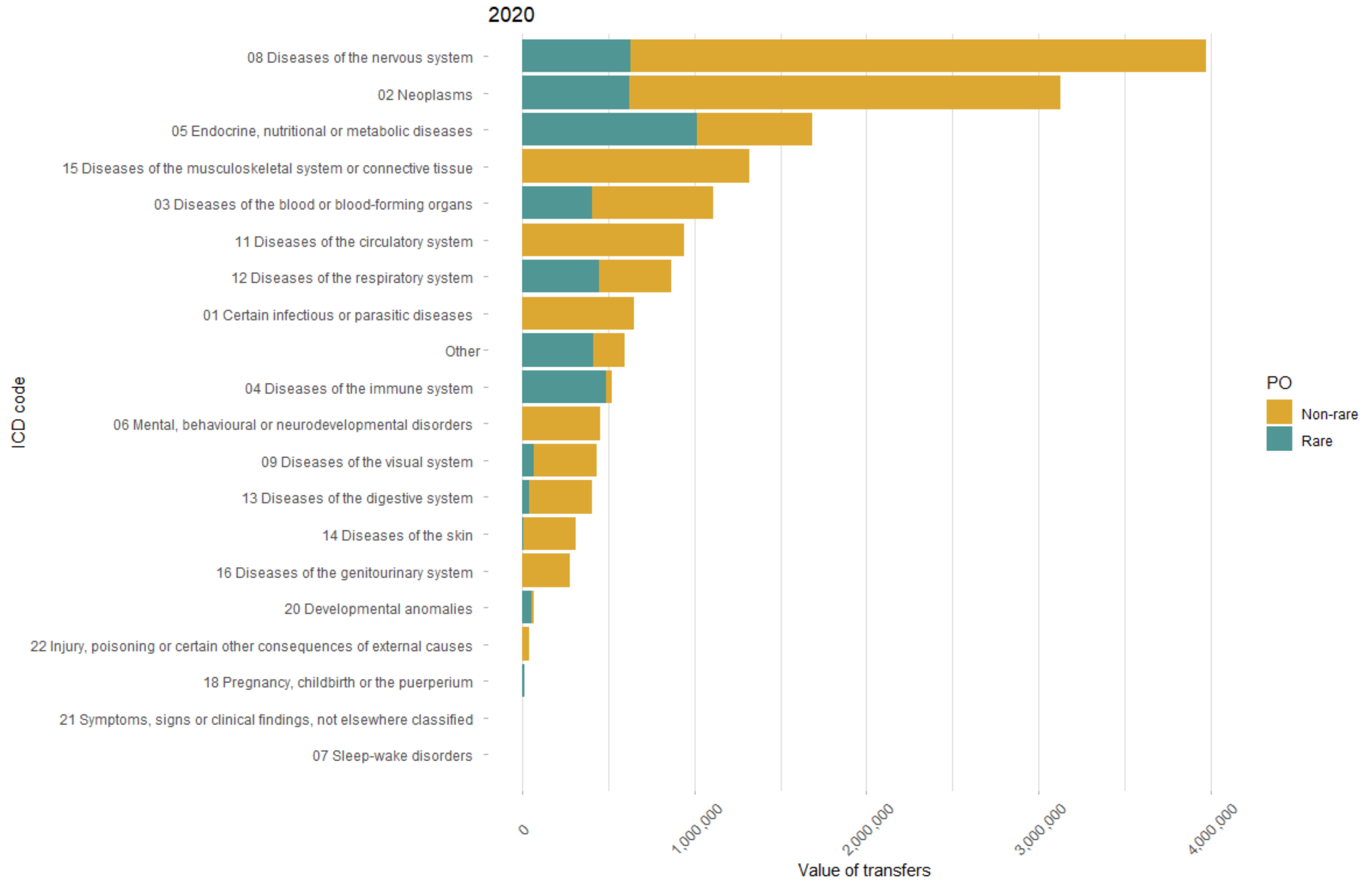
2020
08 Diseases of the nervous system
02 Neoplasms
05 Endocrine, nutritional or metabolic diseases
15 Diseases of the musculoskeletal system or connective tissue
03 Diseases of the blood or blood-forming organs
11 Diseases of the circulatory system
12 Diseases of the respiratory system
01 Certain infectious or parasitic diseases
Other
04 Diseases of the immune system
06 Mental, behavioural or neurodevelopmental disorders
09 Diseases of the visual system
13 Diseases of the digestive system
14 Diseases of the skin
16 Diseases of the genitourinary system
20 Developmental anomalies
22 Injury, poisoning or certain other consequences of external causes
18 Pregnancy, childbirth or the puerperium
21 Symptoms, signs or clinical findings, not elsewhere classified
07 Sleep-wake disorders
ICD code
0
1,000,000
2,000,000
3,000,000
4,000,000
Value of transfers
PO
Non-rare
Rare

Sub-group analyses

Excluded patient organisations

181 payments made 53 to patient organisaitons were excluded from the analysis as they did not match EFPIA's definition of *"not-for-profit organisations, mainly composed of patients and/or caregivers, that represent and/or support the needs of patients and/or caregivers"*.

Figure 4 illustrates the reasons for patient organisations exclusion. Most of the excluded patient organisations were not UK-based (56%; n=101), followed by for profit organisations (36%; n=66) and organisations for which no information could be found online (8%; n=14).

Non-UK patient organisations mostly comprised international alliances of patient organisations, European or Irish organisations. We classified organisations as for-profit if they appeared in the UK government repository of companies[1] as *private limited companies.* Care homes, consultancies and rehabilitation clinics were the most prominent in this category.

Overall, payments to excluded patient organisations amounted to £2,279,445, about 5% of the included payments (Figure 5).

Figure 4. Excluded patient organisations by reason of exclusion

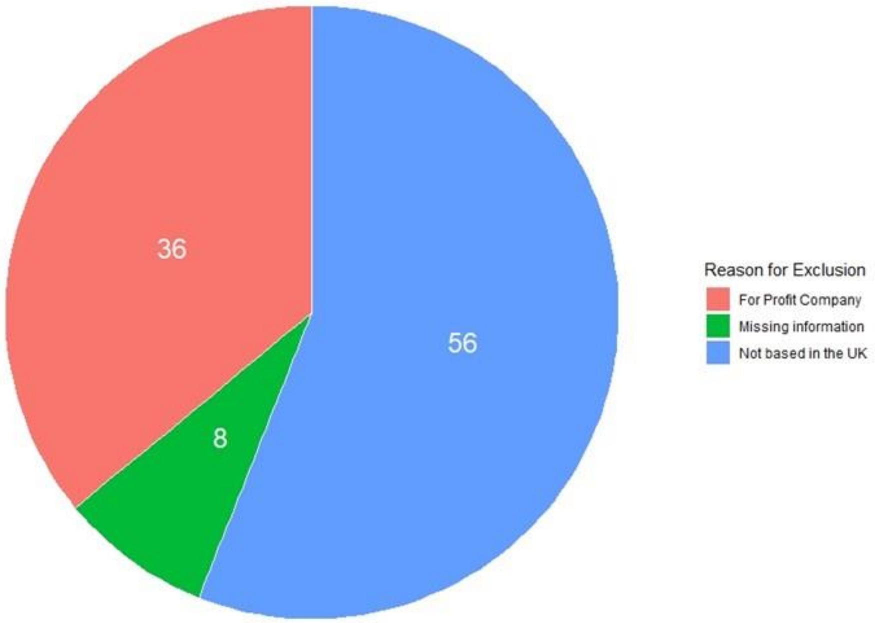

[1] https://find-and-update.company-information.service.gov.uk/

Figure 5. Payments to included and excluded patient organisations

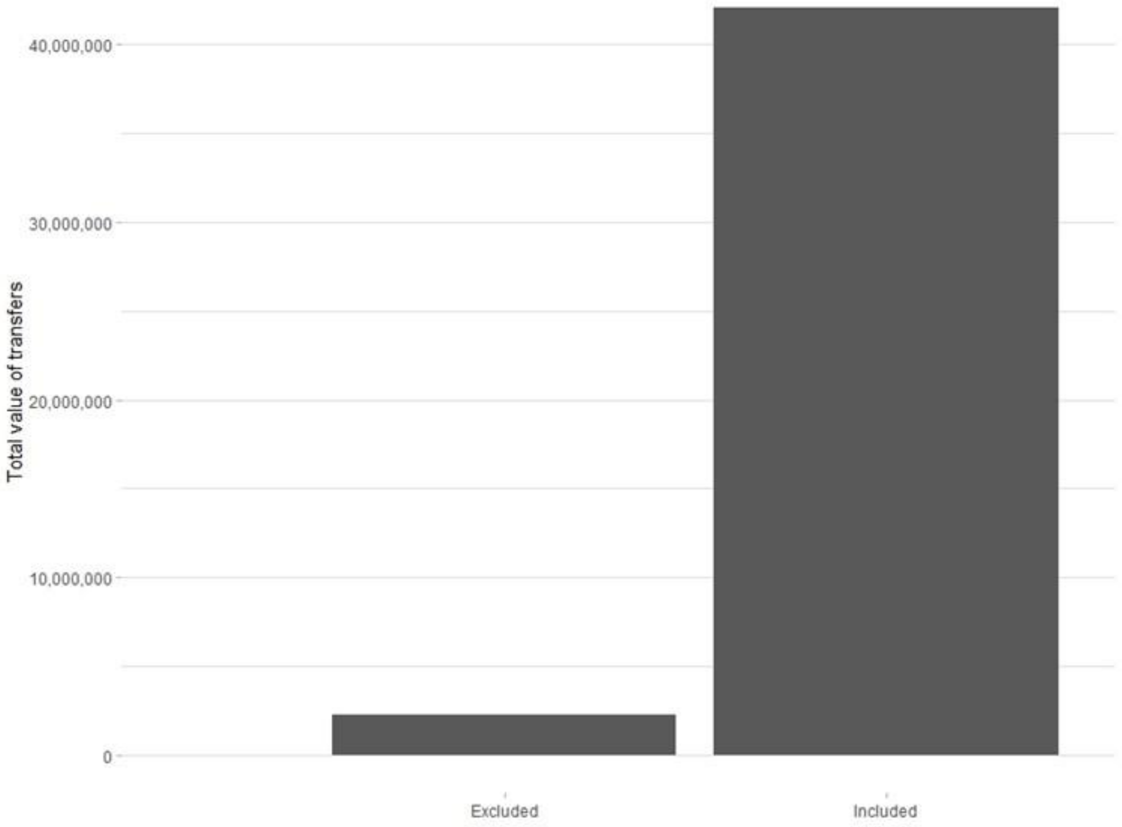

<u>Non-disease-specific organisations</u>

Overall, 378 payments were made to non-disease-specific organisations. Of those, 181 were excluded due to the recipient organisation not meeting the necessary condition to be classified as a patient organisation (as per the analysis presented above). 197 payments were made to 63 non-disease-specific patient organisations. These included hospital charities, carers organisations and hospices.

Payments to non-disease-specific organisations amounted to £ 2,534,044, about 6% of the included disease-specific payments (Figure 6).

For peer review only - http://bmjopen.bmj.com/site/about/guidelines.xhtml

1 **Figure 6. Payments to disease and non-disease-specific patient organisations**

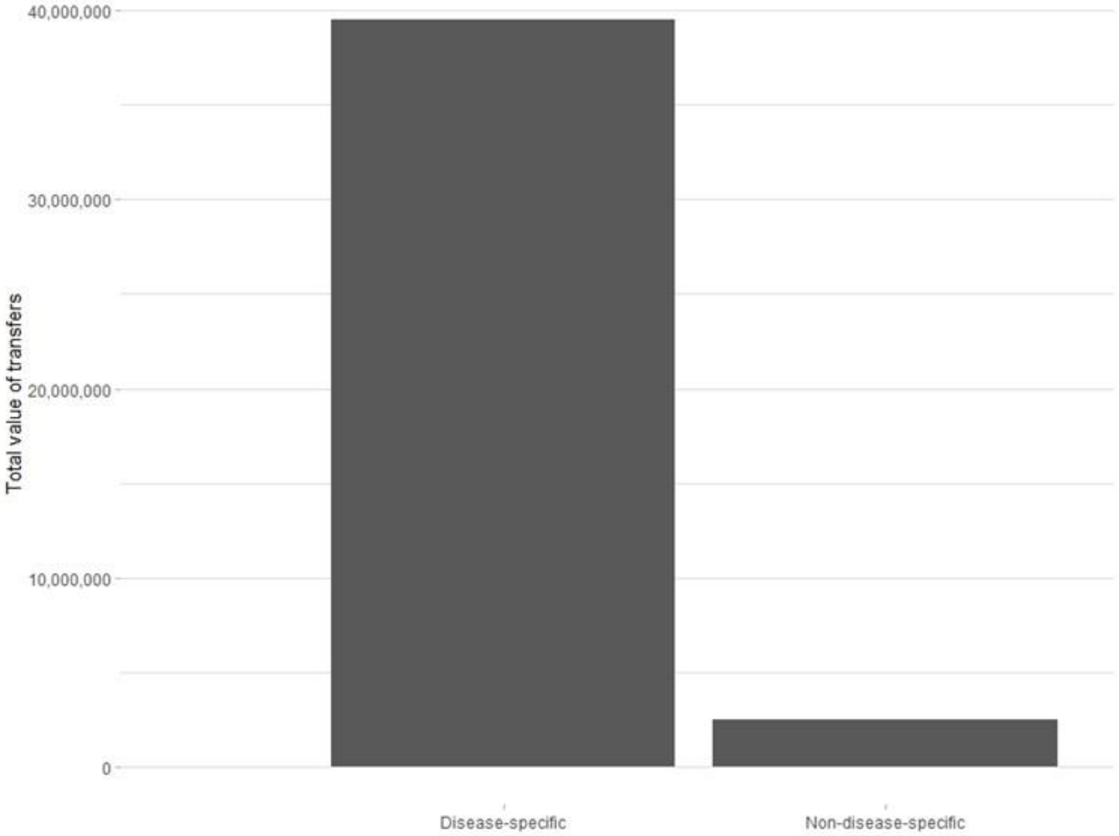

2

3

For peer review only - http://bmjopen.bmj.com/site/about/guidelines.xhtml

References

1. Disclosure UK. ABPI Patient Organisations database 2021 [Available from: https://search.disclosureuk.org.uk/.
2. PMCPA. ABPI Code of Practice 2021 [Available from: https://www.pmcpa.org.uk/the-code/2021-interactive-abpi-code-of-practice/.
3. ONS. Consumer price inflation time series: Office for National Statistics; 2022 [Available from: https://www.ons.gov.uk/economy/inflationandpriceindices/timeseries/l55o/mm23.
4. HMRC. HMRC yearly average and spot rates: HM Revenue and Customs; [Available from: https://www.gov.uk/government/publications/exchange-rates-for-customs-and-vat-yearly.
5. HMRC. HMRC yearly average and spot rates: HM Revenue and Customs; 2022 [Available from: https://www.gov.uk/government/publications/exchange-rates-for-customs-and-vat-yearly.
6. NIH U.S. National Library of Medicine. ClinicalTrials.gov [Available from: https://clinicaltrials.gov/2022.
7. EFPIA. EFPIA Code of Practice on the Relationships between the Pharmaceutical Industry and Patient Organisations: European Federation of Pharmaceutical Industries and Associations, 2011.
8. Ozieranski P, Rickard E, Mulinari, Shai. Exposing drug industry funding of UK patient organisations. *BMJ* 2019;365:l1806. doi: 10.1136/bmj.l1806
9. Orphanet. The portal for rare diseases and orphan drugs 2022 [Available from: https://www.orpha.net/consor/cgi-bin/Disease_Search_Simple.php?lng=EN.
10. Mulinari S, Vilhelmsson A, Rickard E, et al. Five years of pharmaceutical industry funding of patient organisations in Sweden: Cross-sectional study of companies, patient organisations and drugs. *PLoS One* 2020;15(6):e0235021. doi: 10.1371/journal.pone.0235021 [published Online First: 20200624]
11. NICE. Policy on declaring and managing interests for NICE advisory committees, 2018.
12. WHO. ICD-11 for Mortality and Morbidity Statistics 2022 [Available from: https://icd.who.int/browse11/l-m/en#/http://id.who.int/icd/entity/465177735?view=G0.

For peer review only - http://bmjopen.bmj.com/site/about/guidelines.xhtml

CHEERS 2022 Checklist

Topic	No.	Item	Location where item is reported
**Title**			
	1	Identify the study as an economic evaluation and specify the interventions being compared.	p. 1, lines 1-3
**Abstract**			
	2	Provide a structured summary that highlights context, key methods, results, and alternative analyses.	p. 2, lines 4-33
**Introduction**			
**Background and objectives**	3	Give the context for the study, the study question, and its practical relevance for decision making in policy or practice.	p. 4, 5, 6 (all lines)
**Methods**			
**Health economic analysis plan**	4	Indicate whether a health economic analysis plan was developed and where available.	N/A
**Study population**	5	Describe characteristics of the study population (such as age range, demographics, socioeconomic, or clinical characteristics).	p. 7, lines 3-5
**Setting and location**	6	Provide relevant contextual information that may influence findings.	p. 7, line 4
**Comparators**	7	Describe the interventions or strategies being compared and why chosen.	N/A
**Perspective**	8	State the perspective(s) adopted by the study and why chosen.	p. 7, line 4
**Time horizon**	9	State the time horizon for the study and why appropriate.	p. 7, line 5
**Discount rate**	10	Report the discount rate(s) and reason chosen.	N/A
**Selection of outcomes**	11	Describe what outcomes were used as the measure(s) of benefit(s) and harm(s).	p. 7, 8, 9 (all lines)
**Measurement of outcomes**	12	Describe how outcomes used to capture benefit(s) and harm(s) were measured.	p. 7, 8, 9 (all lines)
**Valuation of outcomes**	13	Describe the population and methods used to measure and value outcomes.	p. 8, lines 37-40
**Measurement and valuation of resources and costs**	14	Describe how costs were valued.	N/A
**Currency, price date, and conversion**	15	Report the dates of the estimated resource quantities and unit costs, plus the currency and year of conversion.	p. 7, lines 20-25

For peer review only - http://bmjopen.bmj.com/site/about/guidelines.xhtml

Topic	No.	Item	Location where item is reported
**Rationale and description of model**	16	If modelling is used, describe in detail and why used. Report if the model is publicly available and where it can be accessed.	p. 8, lines 22-36
**Analytics and assumptions**	17	Describe any methods for analysing or statistically transforming data, any extrapolation methods, and approaches for validating any model used.	p. 7, lines 15-17
**Characterising heterogeneity**	18	Describe any methods used for estimating how the results of the study vary for subgroups.	N/A
**Characterising distributional effects**	19	Describe how impacts are distributed across different individuals or adjustments made to reflect priority populations.	N/A
**Characterising uncertainty**	20	Describe methods to characterise any sources of uncertainty in the analysis.	N/A
**Approach to engagement with patients and others affected by the study**	21	Describe any approaches to engage patients or service recipients, the general public, communities, or stakeholders (such as clinicians or payers) in the design of the study.	p. 9, lines 17-20
**Results**			
**Study parameters**	22	Report all analytic inputs (such as values, ranges, references) including uncertainty or distributional assumptions.	N/A
**Summary of main results**	23	Report the mean values for the main categories of costs and outcomes of interest and summarise them in the most appropriate overall measure.	p. 10, 13, 14 (all lines)
**Effect of uncertainty**	24	Describe how uncertainty about analytic judgments, inputs, or projections affect findings. Report the effect of choice of discount rate and time horizon, if applicable.	N/A
**Effect of engagement with patients and others affected by the study**	25	Report on any difference patient/service recipient, general public, community, or stakeholder involvement made to the approach or findings of the study	p. 9, lines 17-20
**Discussion**			
**Study findings, limitations, generalisability, and current knowledge**	26	Report key findings, limitations, ethical or equity considerations not captured, and how these could affect patients, policy, or practice.	p. 15-17 (all lines)
**Other relevant information**			
**Source of funding**	27	Describe how the study was funded and any role of the funder in the identification, design, conduct, and reporting of the analysis	p. 18, lines 11-15
**Conflicts of interest**	28	Report authors conflicts of interest according to journal or International Committee of Medical Journal Editors requirements.	p. 18, lines 16-20

For peer review only - http://bmjopen.bmj.com/site/about/guidelines.xhtml

From: Husereau D, Drummond M, Augustovski F, et al. Consolidated Health Economic Evaluation Reporting Standards 2022 (CHEERS 2022) Explanation and Elaboration: A Report of the ISPOR CHEERS II Good Practices Task Force. Value Health 2022;25. doi:10.1016/j.jval.2021.10.008

For peer review only

For peer review only - http://bmjopen.bmj.com/site/about/guidelines.xhtml

BMJ Open

Industry funding of patient organisations in the United Kingdom: A retrospective study of commercial determinants, funding concentration and disease prevalence

Journal:	*BMJ Open*
Manuscript ID	bmjopen-2022-071138.R2
Article Type:	Original research
Date Submitted by the Author:	09-May-2023
Complete List of Authors:	Gentilini, Arianna; The London School of Economics and Political Science, Department of Health Policy<br>Parvanova, Iva; The London School of Economics and Political Science, Department of Health Policy
**Primary Subject Heading**:	Health policy
Secondary Subject Heading:	Health policy
Keywords:	Health policy < HEALTH SERVICES ADMINISTRATION & MANAGEMENT, PUBLIC HEALTH, Health Equity

SCHOLARONE™
Manuscripts

I, the Submitting Author has the right to grant and does grant on behalf of all authors of the Work (as defined in the below author licence), an exclusive licence and/or a non-exclusive licence for contributions from authors who are: i) UK Crown employees; ii) where BMJ has agreed a CC-BY licence shall apply, and/or iii) in accordance with the terms applicable for US Federal Government officers or employees acting as part of their official duties; on a worldwide, perpetual, irrevocable, royalty-free basis to BMJ Publishing Group Ltd ("BMJ") its licensees and where the relevant Journal is co-owned by BMJ to the co-owners of the Journal, to publish the Work in this journal and any other BMJ products and to exploit all rights, as set out in our licence.

The Submitting Author accepts and understands that any supply made under these terms is made by BMJ to the Submitting Author unless you are acting as an employee on behalf of your employer or a postgraduate student of an affiliated institution which is paying any applicable article publishing charge ("APC") for Open Access articles. Where the Submitting Author wishes to make the Work available on an Open Access basis (and intends to pay the relevant APC), the terms of reuse of such Open Access shall be governed by a Creative Commons licence – details of these licences and which Creative Commons licence will apply to this Work are set out in our licence referred to above.

Other than as permitted in any relevant BMJ Author's Self Archiving Policies, I confirm this Work has not been accepted for publication elsewhere, is not being considered for publication elsewhere and does not duplicate material already published. I confirm all authors consent to publication of this Work and authorise the granting of this licence.

For peer review only

Industry funding of patient organisations in the United Kingdom: A retrospective study of commercial determinants, funding concentration and disease prevalence

Arianna Gentilini and Iva Parvanova

Department of Health Policy, London School of Economics and Political Science, London, UK

For peer review only

Corresponding author

Arianna Gentilini

Department of Health Policy
Cowdray House, COW.4.09
London School of Economics
Houghton street
London WC2A 2AE
England
Email: a.gentilini@lse.ac.uk

For peer review only - http://bmjopen.bmj.com/site/about/guidelines.xhtml

Abstract

Objectives – To assess the relationship between UK-based patient organisation funding and companies' commercial interests in rare and non-rare diseases in 2020.

Design – Retrospective analysis of the value and volume of payments from pharmaceutical companies to patient organisations in the UK matched with data on the conditions supported by patient organisations and drugs in companies' approved portfolios and research and development pipelines.

Setting – UK.

Participants – 74 pharmaceutical companies making payments to 341 UK-based patient organisations.

Main outcome measures – Alignment between the commercial interests of pharmaceutical companies and the disease area focus of patient organisations; difference in the volume and value of payments to patient organisations broken down by prevalence of conditions; industry funding concentration, measured as the number of companies funding each patient organisations, the share of overall industry funding coming from each contributing company and the share of industry funding of each organisation comprised by the single highest payments.

Results – 1,422 payments were made by 74 companies to 341 patient organisations. Almost all funds (90%) from pharmaceutical companies were directed to patient organisations that are aligned with companies' approved drug portfolios and research and development pipelines. Despite rare diseases affecting less than 5% of the UK population, more than 20% of all payments were directed to patient organisations which target such conditions. Patient organisations focusing on rare diseases relied on payments from fewer companies (*p-value* = 0.0031) compared to organisations focusing on non-rare diseases.

Conclusions – Companies predominantly funded patient organisations operating in therapeutic areas relevant to companies' portfolio or drug development pipeline. Patient organisations focusing on rare diseases received more funding relative to the number of patients affected by these conditions and relied more heavily on payments from fewer companies compared to organisations targeting non-rare diseases. Increased independence of patient organisations could help avoiding conflicts of interest.

For peer review only - http://bmjopen.bmj.com/site/about/guidelines.xhtml

Strengths and limitations of this study

- We develop a methodology to determine the concordance between commercial interests of pharmaceutical companies and disease areas supported by patient organisations.
- We present a comparative analysis of industry funding to patient organisations depending on the prevalence of the disease(s) they support.
- Our analysis focuses on a recent time period which might differ from historical trends.

For peer review only

Introduction

Patient organisations – not-for-profit organisations mainly composed of patients and/or caregivers that represent and support the needs of patients or caregivers [1 2] – play an important role in the development, regulatory review, and adoption of new drugs.

During research and development, patient organisations effectively advocate for resources to be directed to conditions where unmet need is highest.[3 4] Patient organisations support research design and planning, helping to identify patient-relevant study endpoints.[4] Patient organisations also represent patient views and preferences at the time of regulatory review and health technology assessment of new drugs.[5 6] For example, during technology appraisals conducted by the National Institute for Health and Care Excellence (NICE), which makes funding recommendations for the English National Health Service (NHS), patients, and organisations representing the interests of patients, provide testimonies of their first-hand experiences on how the disease affects them and those around them.[7] Finally, when drugs are launched, patient organisations contribute to dissemination of research results to patient community and clinicians, and offer support and information on therapies available.[4 8]

Given the increasingly important role of patient organisations it is vital to understand their financial ties with pharmaceutical companies. Previous studies documented the large number and high value of payments from pharmaceutical companies to patient organisations, [2 8-10] the uneven distribution between and within therapeutic areas,[2 10] and the concentration of payments coming from a small number of pharmaceutical firms across multiple jurisdictions.[2 8-16]

What remains unknown is the alignment between the commercial interests of pharmaceutical companies and UK patient organisations' activities. Prior research has demonstrated that industry tends to prioritize commercially attractive conditions, and there is evidence to suggest that the marketing of a drug for a particular disease is associated with increased industry funding to patient organisations operating in that area. [2 10] However, such studies have typically been conducted in different geographic settings and have focused solely on marketed drugs, rather than examining the entire research and development pipeline of pharmaceutical companies. This is especially important given the lengthy timeline for drugs to reach the market,[17] as failure to consider drugs currently undergoing clinical trials may result in an incomplete picture.

Another gap in the literature relates to the dynamics between the pharmaceutical industry and patient organisations supporting rare vs. non-rare conditions. In the UK, diseases are defined rare if they affect up to 5 people in 10,000.[18 19] The low prevalence of rare diseases and their different aetiology, coupled with the lack of interest from policymakers and manufacturers, who often prioritise more profitable and prevalent diseases, has necessitated the formation of patient organisations to advocate for the needs of rare disease patients.[20 21] The National Organisation for Rare Disorders (NORD), serves as the umbrella organisation for rare disease patients in the United States (US) and has been instrumental in lobbying for scientific support and economic incentives to stimulate innovation in rare diseases.[22] This advocacy ultimately led to the passing of the Orphan Drug Act in 1983 in the USA and the EU Regulation on Orphan Medicinal Products in Europe in 2000.[18 23]

For peer review only - http://bmjopen.bmj.com/site/about/guidelines.xhtml

Moreover, the limited availability and complexity of medical knowledge regarding rare diseases have also fostered patients and families affected by these conditions to come together to provide each other with support and medical expertise.[20 24] Patient organisations, which are primarily composed of patients and their caregivers, are in a unique position to share first-hand experiences that can inform research and regulatory decisions.[25] While this is true also for non-rare conditions, patient organisations' input in regulatory and health technology appraisals is particularly important in the context of rare diseases due to scarce evidence. For example, the Scottish Medicines Consortium (SMC) provides opportunities for patient groups and clinicians to have a stronger voice in the decision-making process for drugs used to treat rare and end-of-life conditions.[26] Similarly, three members of patient organisations sit in the Committee for Orphan Medicinal Products (COMP) within the European Medicines Agency (EMA), the body responsible for granting orphan designations to drugs. Patient organisation-led registries that collect real-world data on disease progression can de-risk drug development for rare diseases.[20] While observational studies are common in non-rare diseases, they usually do not require the support of patient organisations' networks as patients are easier to identify and recruit.[3]

Finally, there has been limited exploration of the concentration of industry funding for patient organisations. A recent study by Mulinari and colleagues (2022) examined the average number of pharmaceutical companies making payments to Danish patient organisations,[15] while only one study has investigated the share of industry funding and the top drug company donor's share in UK patient organisations' income.[11] However, no study has specifically focused on the number of companies funding UK patient organisations, nor have they explored whether organisations' industry funding differs based on disease rarity.

Our paper aims to contribute to and expand on existing literature by examining the concordance between the commercial interests of pharmaceutical companies and patient organisations' activities in the UK. Using publicly available data on 2020 payments, we analysed the volume, value of payments to patient organisations according to their disease area of interest, with the objective of examining whether there are differences in funding patterns between rare and non-rare diseases. Lastly, we examined the concentration of industry funding, namely how many companies funded each patient organisation and the extent to which organisations might have been reliant on funding from a single company. Based on the reviewed literature, we formulated the following hypotheses:

- *Hypothesis 1*: Regarding the concordance between the commercial interests of pharmaceutical companies and patient organisations' activities, we expect no difference between rare and non-rare patient organisations, under the assumption that companies are unlikely to fund organisations out of altruistic motives;
- *Hypothesis 2*: Furthermore, we hypothesise that patient organisations targeting rare diseases would receive less overall funding due to their low prevalence. However, the existing incentives, high costs and consequent profitability of some orphan-designated drugs might affect the proportion of funding directed towards these organisations.[27 28]
- *Hypothesis 3*: Considering the limited availability of drugs for rare diseases from a handful of manufacturers, we expect organisations focusing on these conditions to rely

For peer review only - http://bmjopen.bmj.com/site/about/guidelines.xhtml

on payments of higher value and from fewer companies compared to those targeting more prevalent conditions.

For peer review only

For peer review only - http://bmjopen.bmj.com/site/about/guidelines.xhtml

Methods

Data on industry payments

Disclosure reports on pharmaceutical companies' websites were our primary data source on payments from the pharmaceutical industry to UK patient organisations in 2020.[29] Disclosing payments to patient organisations is a requirement of Clause 29 of the Association of British Pharmaceutical Industry (ABPI) Code of Practice.[30] Specifically, the ABPI requires companies to keep a public record of any payment made to patient organisations on their website for a minimum of three years following the payment.[30] Companies that sign up to abide by the ABPI Code accept the jurisdiction of the Prescription Medicines Code of Practice Authority (PMCPA, code regulator), which also affects non-ABPI members operating in the UK.[30] Companies may be sanctioned by the PMCPA if they do not disclose their payments.[30] In an effort to increase transparency, Disclosure UK, an industry-led platform showing payments from pharmaceutical companies to healthcare professionals and organisations, launched a gateway in 2020 that collects hyperlinks to companies' disclosures of payments to patient organisations.[31]

First, we screened the websites of all pharmaceutical companies abiding by the ABPI Code, aided by the Disclosure UK patient organisations gateway. We retrieved payments information from the companies' websites to ensure that all payments were captured. Second, in light of a recent study unveiling that payments to patient organisations were misreported in the Disclosure UK database of payments to healthcare organisations (HCOs),[16] we screened the 2020 Disclosure UK HCOs database for payments to patient organisations.

If payments were not disclosed in the company's website nor in the Disclosure UK HCOs database, we assumed that the company did not make any payments to patient organisations in 2020, as commonly assumed in the literature.[2]

One investigator (AG) extracted payment disclosures from the companies' websites. These comprised the name of the patient organisation, the year when the payment was made, the reason for the payment and its value in the currency reported by the disclosing company. The 2020 Disclosure UK HCOs database was also screened, and recipients were matched to standardised patient organisations names. To ensure the data's accuracy, the final database was scanned for duplicates, but no such instances were found. All payments were first adjusted for inflation using the ONS Consumer Price Index.[32] When reported in different currencies, such as United States Dollars (USD), Swiss Franc (CHF), Swedish Krona (SEK), Norwegian Krone (NKK) and Danish Krone (DKK), the value of the payment was converted to Great British Pounds (GBP), using the ONS historical yearly conversion rates. [33] [34] Two in-kind payments with a monetary value of zero were excluded from the analysis. Further details on variables' cleaning and coding can be found in the Supplemental Material.

Data on patient organisations

We retrieved data on patient organisations from their websites. Details on the therapeutic area they advocated for – proxied by International Classification of Diseases Version 11 (ICD-11)

For peer review only - http://bmjopen.bmj.com/site/about/guidelines.xhtml

codes – and whether the condition(s) was rare or non-rare were also extracted. Conditions were considered rare if they appeared in the Orphanet database of rare diseases, [35] which is platform and repository of data on rare diseases and orphan drugs. Patient organisations that did not match the European Federation of Pharmaceutical Industries and Associations (EFPIA) definition of what constitutes a patient organisation were excluded from the analysis. We chose the EFPIA's definition for the following reasons. First, this corresponds the definition used in the wider peer-reviewed literature.[2 36] Second, other commonly used definitions, such as the one from the EMA, refer to the structure of patient organisations' governing bodies, which have to consist of over 50% patients.[37] Considering the high number of patient organisations included in our analysis, this requirement was challenging – if not impossible – to verify. Second, EFPIA's definition indicates what the pharmaceutical industry considers to be a patient organisation. Therefore, it helped us minimize selection bias issues as it includes a wide range of organisations. We excluded 66 payments to patient organisations that did not match EFPIA's definition. Sub-group analyses on excluded organisations can be found in the Supplemental Material.

Determining commercial interests

We assessed whether – and the extent to which – a pharmaceutical company holds an interest in the disease supported by a patient organisation. We adapted the definition of 'interest' provided by NICE [38]. An interest is when there is, or could be perceived to be, an opportunity for a pharmaceutical company to benefit in the disease area where the patient organisation operates. This could include cases where the pharmaceutical company has a drug developed or in development for a condition targeted by the patient organisation, or where a drug in the company's portfolio or pipeline is restricted to a specific population affected by the disease supported by the patient organisation. We define portfolio as a group of drugs that a pharmaceutical company has already developed, gained regulatory approval for, and is actively marketing or selling. Conversely, pipeline refers to the collection of drug candidates being developed by a pharmaceutical company, at various stages of development, from preclinical research to clinical trials.

To establish whether an interest existed or not, we first classified the conditions targeted by patient organisations to ICD-11 codes using the online ICD-11 database.[39] ICD-11 codes are mutually exclusive, exhaustive and are arranged as a single hierarchical tree, from level one (most general e.g., *neoplasms*) to five (most specific, e.g. *plasma cell myeloma*). This means that specific diseases are nested within broader classifications. Although some patient organisations, such as hospital charities, carers organisations, and hospices, could not be matched to specific ICD-11 codes, they were included in the analysis to provide a comprehensive overview. As a result, the analysis presented results for both disease-specific and non-disease-specific organisations.

We then searched companies' annual reports, websites and the ClinicalTrials.gov registry to determine whether each company had an interest in the condition targeted by the patient organisation receiving the payment. Figure 1 schematically illustrates the approach taken to understand whether – and the degree to which – a company has an interest in the conditions

For peer review only - http://bmjopen.bmj.com/site/about/guidelines.xhtml

(*definitely yes, probably yes, no*). For example, if *Company X* declares in its annual report having a drug in development for multiple myeloma and made a payment to *Blood Cancer UK*, this would be coded as *probably yes,* as the company has a product in its pipeline or portfolio nested within a broader class of conditions targeted by the patient organisation. Conversely, should *Company X* have made a payment to *Myeloma UK,* this would have been coded as *definitely yes,* as there is perfect alignment between the condition targeted by the patient organisation and by *Company X's* drug. Cases in which a company's interest in a certain condition could not be identified were coded as *no.* However, these instances might be due to limitations in data availability and therefore do not necessarily indicate that there was no company interest. Data on pharmaceutical companies' portfolio and pipeline were retrieved from their latest annual reports, company websites and ClinicalTrials.gov.[40]

One investigator (AG) initially coded all data, while the other (IP) blindly re-coded a 30% random sample of payments to validate the data collection process and minimise the risk of reporting errors. We followed this process when validating all data sources described above. Any disagreement was discussed until consensus was reached.

Analysis of industry funding concentration

We assessed the concentration of industry funding received by patient organisations. In a prior study, Ozieranski and colleagues examined funding disparities among healthcare organisations in the UK in 2015, using the Gini coefficient to assess the distribution of funding.[41] However, the authors acknowledged that the data preparation process presented challenges, limiting the analysis to payments from a single year. While this methodology has its advantages, we found that the time-consuming process of reshaping the data outweighed the benefits over using descriptive statistics. In particular, we calculated (1) the number of companies funding each patient organisations, (2) the share of overall industry funding to each patient organisations coming from each contributing company and (3) the share of industry funding of each organisation comprised by the single highest payment.

The Supplemental Material provides further details on the data collection and how the outcomes were constructed. Descriptive statistics and tests, such as ranges and Mann–Whitney U tests, were presented in the analysis. These statistics were preferred over the mean due to the skewed distribution of the data analysed. All analyses and data visualisations were performed using Stata 17 and RStudio (*ggplot2* package), respectively.

Patient and public involvement

Patients were not involved in this study as our analyses focused on patient organisations as institutional actors rather than single patients with specific conditions. We plan to disseminate key findings from our analysis to patients and members of the public.

For peer review only

For peer review only - http://bmjopen.bmj.com/site/about/guidelines.xhtml

Results

In 2020, 74 companies made 1,422 payments to 341 patient organisations, amounting to £22.6 million. Out of the total of 1,422 payments made by pharmaceutical companies to patient organisations in 2020, 82% (1,168 payments) with a value of £18 million were accurately disclosed on the companies' websites. The remaining 18% (254 payments) with a value of £4.6 million were reported in the Disclosure UK HCOs database. Among the companies, 24 out of 74 reported payments only on their websites, while 14 reported payments only in the Disclosure UK HCOs database, and 36 reported payments in both.

Overall, *diseases of the nervous system* (£4.3 million) was the most funded therapeutic area over time, followed by *neoplasms* (£3.2 million) and *endocrine, nutritional or metabolic diseases* (£3.4 million). The conditions that received more funding in 2020 were multiple sclerosis (£1.7 million), followed by obesity (£1.4 million) and epilepsy (£1 million). Pfizer, Novo Nordisk, UCB, Novartis and Roche were the top five funders over the study period (Figure 2). These companies contributed to more than a third (36%) of all payments.

Table 1 summarises the number and value of payments to patient organisations.

Companies' interest in payments to patient organisations

In 2020, 85% of all payments were directed to patient organisations that were judged to be aligned with their portfolio or pipeline. Only 15% of payments were made to organisations that focused on conditions that could not be linked to a product in the funder's portfolio or pipeline. Table 2 shows the volume and value of payments, broken down by the company's interest variable, overall and whether patient organisations targeted a rare or non-rare disease. Payments to patient organisations targeting a disease for which the company has a product developed or in development (*definitely yes*) made up 56% and 54% for patient organisations targeting rare and non-rare conditions, respectively. However, this difference was not statistically significant as anticipated in *Hypothesis 1* ($\chi^2 =$ 1.049, *p-value* = 0.592).

The monetary value of payments coded as *definitely yes* accounted for 55% of the overall payment value. However, this was as high as 67% for patient organisations targeting rare diseases, versus 59% for organisations focusing on non-rare conditions. This difference was found to be statistically significant ($\chi^2 =$ 370.163, *p-value* = 0. 058). When payments coded as *probably yes* were included, the share increased to 90% and 97% for all patient organisations and disease-specific organisations only, respectively.

For peer review only - http://bmjopen.bmj.com/site/about/guidelines.xhtml

Table 1. Number and value of payments from the pharmaceutical industry to UK patient organisations broken down by year and rarity of diseases

**Payment statistics**	
**Number of payments**	1,422
**Median payment (IQR; overall)**	£7,943 (£1,200 - £15,000)
**Median payment (IQR; rare)**	£8,775 (£2,500 - £15,965)
**Median payment (IQR; non-rare)**	£9,060 (£1,520 - £16,850)
**Value of payments (£; overall)**	£22,577,314
**Value of payments (£; rare)**	£4,629,779
**Value of payments (£; non-rare)**	£15,875,662
**Number of pharmaceutical companies**	74
**Number of patient organisations**	341

Abbreviations: IQR (Interquartile range).
Notes: All payments are expressed in 2020 GBP. The Supplemental Materials detail the inflation multipliers and conversion rates used, both retrieved from the Office of National Statistics (ONS) website. Further details on how patient organisation data were cleaned and coded, please see the Supplemental Materials . Please note that the number of pharmaceutical companies and patient organisations making and receiving payments across the study period refers to companies and organisations that made or received at least one payment, respectively.

Table 2. Volume and value of payments by company interests in 2020

**PO type**	**Company's interest**	**Volume**; n (%)	**Value**: £
Overall†	Definitely yes	678 (48%)	£12,529,514 (56%)
	Probably yes	525 (37%)	£7,700,069 (34%)
	No*	219 (15%)	£2,347,732 (10%)
Rare	Definitely yes	161 (56%)	£3,119,217 (67%)
	Probably yes	115 (40%)	£1,388,545 (30%)
	No*	10 (4%)	£122,017 (3%)
Non-rare	Definitely yes	517 (54%)	£9,410,297 (59%)
	Probably yes	389 (41%)	£6,056,915 (38%)
	No*	46 (5%)	£408,449 (3%)

For peer review only

Notes: *Definitely yes* indicates payments directed to patient organisations that operated in a disease area (ICD-11 level 4 or higher) for which the company has a product in its portfolio or pipeline. *Probably yes* indicates directed to patient organisations that operated in a disease area (ICD-11 level 3 or lower) for which the company has a product in its portfolio or pipeline. *No* refers to directed to patient organisations that operated in a disease area for which no link could be found to the company's portfolio or pipeline. The higher the ICD-11, the more specific the condition. For example, if the ICD-11 level 4 is *Plasma cell neoplasms,* level 2 would be *Neoplasms of hematopoietic or lymphoid tissues.* Further details on how this variable was constructed can be found in the Supplemental Material.

*Please note that the *No* category of interest conservatively includes also interests that were considered as unclear.

†Please note that the *Overall* results are not a sum of the *Rare* and *Non-rare* results, as they also include patient organisations that could not be classified in either group and are non-disease-specific.

For peer review only

Industry funding of patient organisations focusing on rare vs. non-rare conditions

Of the £22.6 million in payments from industry to patient organisations, £4.6 million (21%; n=286) were directed to organisations focusing on rare diseases while £15.9 million (70%; n=952) to organisations supporting non-rare conditions. The remaining 9% was directed to non-disease-specific patient organisations, which were excluded from this analysis. Linking these results to *Hypothesis 2*, we observe that patient organisations supporting rare diseases received less but still substantial funding.

The most funded patient organisation overall in 2020 was the European Association for the Study of Obesity, receiving almost £1.5 million, followed by Epilepsy Society (£955,600) and Shift.MS (£588,451). Among the top ten recipients overall in 2020, only one focused on rare diseases (Cystic Fibrosis Trust). However, it is worth noting that Blood Cancer UK, which focuses on malignant haematological malignancies including rare cancers, ranked seventh on the list.[42] The Cystic Fibrosis Trust (£445,229), The Society for Mucopolysaccharide Diseases (£358,037), and the International Patient Organisation for Primary Immunodeficiencies (£345,914) were the top three recipients focusing on rare diseases, followed by Myeloma UK with a slightly lower amount (£340,604).

Figure 3 shows therapeutic areas in order from most to least funded, broken down by rarity of disease targeted. In the case of organisations focusing on rare diseases, *endocrine, nutritional or metabolic disease*, *neoplasms* and *diseases of the nervous system* received most funds. Together, the top three most funded disease areas represented about half of overall funding (57%). When looking at the non-rare conditions that attracted most funding, multiple sclerosis was first (£1.7 million), followed by diabetes (£1.4 million) and epilepsy (£1 million). Cystic fibrosis, primary immunodeficiencies, and lysosomal storage diseases, which include rare metabolic disorders such as Fabry and Gaucher diseases, received the highest funding overall, attracting £445,229, £363,998 and £358,037, respectively.

For peer review only - http://bmjopen.bmj.com/site/about/guidelines.xhtml

Industry funding concentration

Each patient organisation received payments from a median of approximately one unique company, with 1 (IRQ:1-2) and 2 (IQR:1-3) companies funding patient organisations targeting rare and non-rare diseases, respectively. However, this difference was not statistically significant (z = 1.582, *p-value* = 0.114). Overall, the range of unique companies making payments to a unique patient organisation spanned from a minimum of 1 to a maximum of 13. The latter was recorded for Genetic Alliance UK, a national charity and an alliance of over 200 patient organisations, supporting those affected by rare genetic conditions.

In our sample, the median yearly payment of a company to a patient organisation comprised 24% of the its overall industry payments (IQR: 9.5%-74%). When looking at patient organisations focusing on rare diseases, the median company contribution was as high as 30% (IQR: 11.6%-93%) versus 23% (IQR: 9.4%-65.8%) for non-rare conditions (z = -2.164, *p-value* = 0.031).

Finally, the share of industry funding comprised by the single highest payment per organisation amounted to an average of 67.5% (SD: 0.30) for all years, ranging from a minimum of 8.5% to a maximum of 100%. The highest value payment in the case of patient organisations targeting rare diseases made up a larger share of the overall industry funding (median: 71%, IQR: 43.5%-100%), despite not significant, compared to those focusing on more prevalent conditions (median: 62.5%, IQR: 34.7%-100%). While there was not a significant difference in the number of funding companies between patient organisations supporting rare and non-rare diseases (z = -1.087, *p-value* = 0.277) as stated in *Hypothesis 3*, the former relied on larger payments. Histograms illustrating the distribution of the statistics explored in this analysis can be found in the Supplemental Materials.

For peer review only - http://bmjopen.bmj.com/site/about/guidelines.xhtml

Discussion

In this study, we evaluated the financial links between the pharmaceutical industry and patient organisations in the UK in 2020. This is the first study to document the almost-perfect concordance of pharmaceutical company interests and patient organisation funding in the UK. Almost all industry payments during our study period – in terms of both volume (85%) and value (90%) – were to patient organisations aligned with pharmaceutical companies' portfolios and pipelines. This share was even higher when considering only disease-specific patient organisations (97%). Despite rare diseases affecting less than 5% of the UK population, more than 20% of industry funding to patient organisations in 2020 was directed towards organisations focusing on such conditions (£4.6 million / £22.6 million). Finally, we found that patient organisations targeting rare diseases relied on payments from fewer companies but of higher value compared to organisations focusing on non-rare diseases.

The almost-perfect concordance between industry interests and patient organisation activities likely reflect the commercial attractiveness of conditions targeted by pharmaceutical companies.[2 43] Such close alignment between the interests of companies and patient organisations might undermine the credibility of patient organisations as perceived by the general public and might raise questions about patient organisations' inputs in regulatory and health technology appraisals.[9 44 45] Similarly, a study found that during NICE appraisal meetings fewer than 25% of all relevant financial ties between patient organisations and pharmaceutical companies were disclosed.[46] As discussed by the Mandeville and colleagues, this lack of transparency increases the risk of conflicts of interest not being properly detected and managed.

Our findings make an important contribution to the existing body of literature on industry funding of patient organisations. Ozieranski et al found that industry donated over £57 million to UK patient organisations from 2012 to 2016, an average of £11.5 million per year.[2] The authors also observed that payments were concentrated in commercially attractive therapeutic areas, with organisations focusing on cancer receiving more than 36% of overall payments.[2] However, the study did not examine whether companies were more likely to fund organisations that target diseases for which they have already developed or are currently developing products. Another earlier study examined payments to Swedish patient organisations and found an association between drug commercialisation and industry funding.[10] The authors did not take into account products in the companies' pipelines nor drugs that might had not yet launched in Sweden. Considering that patient organisations have an important role not only in the post-commercialisation phase but also in the R&D and approval stages. We therefore developed a replicable classification model to determine whether payments from companies were directed at organisations that were aligned with their portfolios and pipelines.

Patient organisations focusing on rare diseases can drive both supply of and demand for medicinal products due to their research, advocacy and education role. [4 47] As a result of their close ties with patients, these organisations have the credibility and power to educate patient communities, advocate for access to available therapies and raise awareness on the unmet need of certain conditions.[4 20 48] Although a large share of both the value and number of payments were directed to patient organisations focusing on rare diseases, most funds targeted

For peer review only

commercially attractive rare conditions, such as multiple myeloma and cystic fibrosis, where the unmet need is relatively low compared to other rare conditions. These are diseases that have relatively high prevalence and for which 10 and 29 treatments, respectively, are currently approved for use in Europe.[35 49] Furthermore, rare diseases have proved a lucrative asset for pharmaceutical companies.[43] The additional market protection granted to orphan-designated product and the often higher willingness to pay from payers has led companies to increasingly focus on these medicines, which can offer a high return on investment.[27 28] This poses the risk of widening already existing health inequities, where severe and debilitating rare conditions that affect a small number of patients do not receive the resources they need and have to rely on limited public grants.[50]

Finally, our analysis showed that patient organisations focusing on rare diseases are funded by very few companies, relying on a single payment for over 70% of their industry-reported income. Despite the share of industry contributions among the overall patient organisation's income was found to be low in the literature,[11] this increases the risk of pursuing the company's commercial interests rather than objectively representing a patient body.[12] In this study we find that patient organisation received payments from a median of approximately one unique company (IRQ:1-3), ranging from 1 to a maximum of 13. This corresponds to an average of 2.6 (SD:2.3) funding companies per patient organisation. This is consistent with findings from a recent study investigating the distribution of payments from industry to Danish patient organisations, which found that on average, most organisations were funded by 2.6 (SD:2.1) on average.[15]

These findings have important implications for policy and practice. To minimise conflicts of interests and maintain the integrity of patient organisations, particular attention should be paid to funding from companies in the immediate period before or after a patient organisation has endorsed this company's product.[46] One way of avoiding potential conflicts of interest is through increased transparency. Despite considerable progress on this front, especially in terms of reporting the monetary value of industry payments, there are still gaps in reporting.[51]

As highlighted in this and other studies, several companies misreport their payments to patient organisations.[16] Our study found that only 32% of companies disclose all of their payments correctly (i.e., on their website), while the rest report them on both their websites and the Disclosure UK HCOs database (49%) or solely on the latter (19%). This duplication of reporting efforts makes it harder to achieve transparency and obtain a comprehensive overview of the financial relationships between companies and patient organisations. Therefore, efforts should be made to establish a unique repository for payments to patient organisations, similar to the one currently in place for physicians and healthcare organisations.

Furthermore, the financial independence of patient organisations is fundamental to ensure that patients' interests are at the forefront of the organisations' agenda.[52] Compromising this independence can have a detrimental effect and distort public health priorities. For example, AbbVie-sponsored patient organisations were found to strongly oppose switching to biosimilars for Humira, the company's blockbuster drug, in various countries.[15] Similarly, a recent investigation uncovered strong financial connections between Novo Nordisk and UK-based patient organisations that supported the approval of the company's latest obesity drug.

For peer review only - http://bmjopen.bmj.com/site/about/guidelines.xhtml

This, alongside other ongoing investigations, culminated in the suspension of the company from ABPI.[53] The strong financial ties between Novo Nordisk and patient organisations, contributing to the NICE appraisal of the company's drug, raises serious concerns about these groups' independence and might ultimately harm patients. Notably, our analysis found Novo Nordisk to be the second highest funder of patient organisations in term of value in 2020 for an amount of more than £1.8 million. In the long term, policymakers should make sure that patient organisations receive adequate public funding regardless of whether they focus on conditions that are profitable for the industry. Such public funding is particularly important for patient organisations supporting rare diseases, as relatively few companies have financial links with patient organisations focusing on rare diseases, potentially creating high reliance on few high-value payments.

This study had limitations. First, the lack of mandatory reporting of payments to patient organisations by companies that do not comply with the ABPI Code is a major limitation of our analysis.[54] For example, our dataset does not include payments by Vertex, a company with a rare-focused portfolio and a strong presence in cystic fibrosis.[55] Even for companies that are signatories of the ABPI Code, underreporting of payments to patient organisations and removal of disclosure reports from the public domain has been observed.[13 56 57] Second, in our assessment of company interests, we made a conservative assumption that only patient organisations which target relatively narrow conditions were eligible to be coded as *definitely yes*. Despite this assumption, we concluded that more than half of payments were in therapeutic areas in which companies had a clear interest. Finally, our analysis focused on a recent though limited time period. While previous publications show similar trends in terms of the most funded diseases and absolute value of payments,[2 10] lending credibility to our analysis and underlying data, it is still unclear whether these trends hold over time and their generalisability to other periods.

There are several avenues which can be explored further to build on this analysis. While some of the previous literature on the topic has focused on the financial dependency of patient organisations' budgets from pharmaceutical funding,[11] whether this differs depending on the rarity of the disease targeted has not been explored. Due to the small number of patients affected by rare conditions, patient organisations that target such conditions may be less well-equipped to finance their activities via charitable events and may rely more heavily on contributions from pharmaceutical companies. Lastly, while our analysis did not evaluate the effect of Covid-19 on the financial dynamics between pharmaceutical companies and patient organisations, we expect that the pandemic had a substantial effect on the type, value and distribution of payments. Future research should examine the impact of Covid-19 on industry funding of patient organisations.

Conclusions

Almost all industry funding of UK patient organisations in 2020 was in areas that were aligned with companies' approved drug portfolios and research and development pipelines. Pharmaceutical companies spent a larger amount on patient organisations focusing on rare diseases and these organisations relied on a small of companies for their funding.

For peer review only - http://bmjopen.bmj.com/site/about/guidelines.xhtml

Acknowledgements: We thank Dr Huseyin Naci and Dr Olivier Wouters, both from the Department of Health Policy at the London School of Economics and Political Science, for providing comments on earlier drafts of this paper. We also acknowledge Dr Mylene Lagarde and Dr Panos Kanavos for the support provided when finalising the manuscript.

Contributors: AG developed a preliminary version of the study and developed it further with IP. AG collected the data. AG and IP did the analysis, wrote and reviewed the manuscript. Both authors had full access to all of the data (including statistical reports and tables) in the study and can take responsibility for the integrity of the data and the accuracy of the data analysis. The corresponding author attests that all listed authors meet authorship criteria and that no others meeting the criteria have been omitted. AG is the guarantor.

Funding: This research received no specific grant from any funding agency in the public, commercial or not-for-profit sectors. AG is funded by the ESRC for her doctoral research (Project reference: 2480077) and is employed by Imperial College London. IP is funded by the ESRC for her doctoral research (Project reference: 2311285) and is employed by Imperial College London.

Competing interest statement: No competing interest.

Ethical approval: This study does not involve human participants and ethical approval was not required.

Data sharing: A dataset of all publicly available data used in the study is available from the corresponding author at a.gentilini@lse.ac.uk.

Transparency declaration: The lead author affirms that the manuscript is an honest, accurate, and transparent account of the study being reported; that no important aspects of the study have been omitted; and that any discrepancies from the study as planned (and, if relevant, registered) have been explained.

The Corresponding Author has the right to grant on behalf of all authors and does grant on behalf of all authors, an exclusive licence (or non-exclusive for government employees) on a worldwide basis to the BMJ Publishing Group Ltd to permit this article (if accepted) to be published in BMJ editions and any other BMJPGL products and sublicences such use and exploit all subsidiary rights, as set out in our licence.

For peer review only

References

1. EFPIA. EFPIA Code of Practice on the Relationships between the Pharmaceutical Industry and Patient Organisations: European Federation of Pharmaceutical Industries and Associations, 2011.
2. Ozieranski P, Rickard E, Mulinari, Shai. Exposing drug industry funding of UK patient organisations. *BMJ* 2019;365:l1806. doi: 10.1136/bmj.l1806
3. Polich GR. Rare disease patient groups as clinical researchers. *Drug Discovery Today* 2012;17(3):167-72. doi: https://doi.org/10.1016/j.drudis.2011.09.020
4. Geissler J, Ryll B, di Priolo SL, et al. Improving Patient Involvement in Medicines Research and Development::A Practical Roadmap. *Therapeutic Innovation & Regulatory Science* 2017;51(5):612-19. doi: 10.1177/2168479017706405
5. MHRA. Patient Involvement Strategy 2021-25: Medicines and Healthcare products Regulatory Agency 2020.
6. MHRA. Putting patients first: A new era for our agency. Delivery Plan 2021-2023: Medicines and Healthcare products Regulatory Agency 2020.
7. NICE. Public Involvement Programme - Overview of technology appraisals: A factsheet for patient and carer organisations: National Institute for Health and Care Excellence, 2014
8. Fabbri A, Parker L, Colombo C, et al. Industry funding of patient and health consumer organisations: systematic review with meta-analysis. *BMJ* 2020;368:l6925. doi: 10.1136/bmj.l6925
9. Rose SL, Highland J, Karafa MT, et al. Patient Advocacy Organizations, Industry Funding, and Conflicts of Interest. *JAMA Intern Med* 2017;177(3):344-50. doi: 10.1001/jamainternmed.2016.8443
10. Mulinari S, Vilhelmsson A, Rickard E, et al. Five years of pharmaceutical industry funding of patient organisations in Sweden: Cross-sectional study of companies, patient organisations and drugs. *PLoS One* 2020;15(6):e0235021. doi: 10.1371/journal.pone.0235021 [published Online First: 20200624]
11. Ozieranski P, Pitter JG, Rickard E, et al. A 'patient-industry complex'? Investigating the financial dependency of UK patient organisations on drug company funding. *Sociol Health Illn* 2022;44(1):188-210. doi: 10.1111/1467-9566.13409 [published Online First: 20211207]
12. Rose SL. Patient advocacy organizations: institutional conflicts of interest, trust, and trustworthiness. 2014(1748-720X (Electronic))
13. Lexchin J, Batt S, Goldberg D, et al. National patient groups in Canada and their disclosure of relationships with pharmaceutical companies: a cross-sectional study. *BMJ Open* 2022;12(3):e055287. doi: 10.1136/bmjopen-2021-055287
14. Parker L, Fabbri A, Grundy Q, et al. "Asset exchange"—interactions between patient groups and pharmaceutical industry: Australian qualitative study. *BMJ* 2019;367:l6694. doi: 10.1136/bmj.l6694
15. Mulinari S, Pashley D, Ozieranski P. Advancing international comparison of pharmaceutical industry funding of patient advocacy: Focus on Denmark. *Health Policy* 2022;126(12):1256-62. doi: https://doi.org/10.1016/j.healthpol.2022.11.003
16. Rickard E, Carmel E, Ozieranski P. Comparing pharmaceutical company payments in the four UK countries: a cross-sectional and social network analysis. *BMJ Open* 2023;13(3):e061591. doi: 10.1136/bmjopen-2022-061591
17. Wouters OJ, McKee M, Luyten J. Estimated Research and Development Investment Needed to Bring a New Medicine to Market, 2009-2018. 2020(1538-3598 (Electronic))
18. European Commission. Regulation (EC) No 141/2000 of the European Parliament and of the Council of 16 December 1999 on Orphan Medicinal Products, 2000.
19. Department of Health & Social Care. Policy Paper - The UK Rare Diseases Framework 2021 [Available from: https://www.gov.uk/government/publications/uk-rare-diseases-framework/the-uk-rare-diseases-framework.
20. Aymé S, Kole A, Groft S. Empowerment of patients: lessons from the rare diseases community. *Lancet* 2008;371(9629):2048-51. doi: 10.1016/s0140-6736(08)60875-2

For peer review only - http://bmjopen.bmj.com/site/about/guidelines.xhtml

21. Gamba S, Magazzini L, Pertile P. R&D and market size: Who benefits from orphan drug legislation? *Journal of Health Economics* 2021;80:102522. doi: https://doi.org/10.1016/j.jhealeco.2021.102522
22. Waxman HA. The Waxman Report : How Congress Really Works: First edition. New York : Twelve, 2009. 2009.
23. Office of the Federal Register NAaRA. Orphan Drug Act - 6 Stat. 2049. In: Office. USGP, ed., 1983.
24. Halley MC. From "Ought" to "Is": Surfacing Values in Patient and Family Advocacy in Rare Diseases. 2021(1536-0075 (Electronic))
25. Dunkle M, Pines W, Saltonstall PL. Advocacy Groups and Their Role in Rare Diseases Research. In: Posada de la Paz M, Groft SC, eds. Rare Diseases Epidemiology. Dordrecht: Springer Netherlands 2010:515-25.
26. Scottish Medicines Consortium. Patient and Clinician Engagement (PACE) process [Available from: https://www.scottishmedicines.org.uk/how-we-decide/pace/2023.
27. Côté A, Keating B. What Is Wrong with Orphan Drug Policies? *Value in Health* 2012;15(8):1185-91. doi: https://doi.org/10.1016/j.jval.2012.09.004
28. Igho JO, Elizabeth AS, Matthew JT, et al. Effectiveness, safety and costs of orphan drugs: an evidence-based review. *BMJ Open* 2015;5(6):e007199. doi: 10.1136/bmjopen-2014-007199
29. Disclosure UK. ABPI Patient Organisations database 2021 [Available from: https://search.disclosureuk.org.uk/.
30. PMCPA. ABPI Code of Practice 2021 [Available from: https://www.pmcpa.org.uk/the-code/2021-interactive-abpi-code-of-practice/.
31. Disclosure UK. ABPI Patient Organisations database 2021 [Available from: https://search.disclosureuk.org.uk/2020.
32. ONS. Consumer price inflation time series: Office for National Statistics; 2022 [Available from: https://www.ons.gov.uk/economy/inflationandpriceindices/timeseries/l55o/mm23.
33. HMRC. HMRC yearly average and spot rates: HM Revenue and Customs; [Available from: https://www.gov.uk/government/publications/exchange-rates-for-customs-and-vat-yearly.
34. HMRC. HMRC yearly average and spot rates: HM Revenue and Customs; 2022 [Available from: https://www.gov.uk/government/publications/exchange-rates-for-customs-and-vat-yearly.
35. Orphanet. The portal for rare diseases and orphan drugs 2022 [Available from: https://www.orpha.net/consor/cgi-bin/Disease_Search_Simple.php?lng=EN.
36. Rothman SM, Raveis VH, Friedman A, et al. Health Advocacy Organizations and the Pharmaceutical Industry: An Analysis of Disclosure Practices. *American Journal of Public Health* 2011;101(4):602-09. doi: 10.2105/AJPH.2010.300027
37. European Medicines Agency. Criteria to be fulfilled by patient, consumer and healthcare professional organisations involved in European Medicines Agency (EMA) activities 2018 [Available from: https://www.ema.europa.eu/en/documents/regulatory-procedural-guideline/criteria-be-fulfilled-patient-consumer-healthcare-professional-organisations-involved-european_en.pdf.
38. NICE. Policy on declaring and managing interests for NICE advisory committees, 2018.
39. WHO. ICD-11 for Mortality and Morbidity Statistics 2022 [Available from: https://icd.who.int/browse11/l-m/en#/http://id.who.int/icd/entity/465177735?view=G0.
40. NIH U.S. National Library of Medicine. ClinicalTrials.gov [Available from: https://clinicaltrials.gov/2022.
41. Ozieranski P, Csanadi M, Rickard E, et al. Analysis of Pharmaceutical Industry Payments to UK Health Care Organizations in 2015. 2019(2574-3805 (Electronic))
42. Blood Cancer UK. Blood cancer types [Available from: https://bloodcancer.org.uk/understanding-blood-cancer/blood-cancer-types/2023.
43. Hughes DA, Poletti-Hughes J. Profitability and Market Value of Orphan Drug Companies: A Retrospective, Propensity-Matched Case-Control Study. 2016(1932-6203 (Electronic))
44. McCoy MS, Carniol M, Chockley K, et al. Conflicts of Interest for Patient-Advocacy Organizations. *New England Journal of Medicine* 2017;376(9):880-85. doi: 10.1056/NEJMsr1610625

For peer review only - http://bmjopen.bmj.com/site/about/guidelines.xhtml

45. Jones K. In whose interest? Relationships between health consumer groups and the pharmaceutical industry in the UK. 2008(1467-9566 (Electronic))
46. Mandeville KL, Barker R, Packham A, et al. Financial interests of patient organisations contributing to technology assessment at England's National Institute for Health and Care Excellence: policy review. *BMJ* 2019;364:k5300. doi: 10.1136/bmj.k5300
47. Mavris M, Le Cam Y. Involvement of patient organisations in research and development of orphan drugs for rare diseases in europe. 2012(1661-8769 (Print))
48. Bedlington N, Geissler J, Houyez F, et al. Role of Patient Organisations. In: Facey KM, Ploug Hansen H, Single ANV, eds. Patient Involvement in Health Technology Assessment. Singapore: Springer Singapore 2017:401-10.
49. European Medicines Agency. European public assessment reports (EPAR), 2022.
50. Baggott R, Jones K. The Big Society in an age of austerity: threats and opportunities for Health Consumer and Patients' Organizations in England. 2015(1369-7625 (Electronic))
51. Lexchin J. Association between commercial funding of Canadian patient groups and their views about funding of medicines: An observational study. *PLOS ONE* 2019;14(2):e0212399. doi: 10.1371/journal.pone.0212399
52. McCoy MS, Emanuel EJ. Why There Are No "Potential" Conflicts of Interest. *JAMA* 2017;317(17):1721-22. doi: 10.1001/jama.2017.2308
53. Das S, Ungoed-Thomas J. Revealed: experts who praised new 'skinny jab' received payments from drug maker. *The Guardian* 2023.
54. Ozieranski P, Martinon L, Jachiet P-A, et al. Accessibility and quality of drug company disclosures of payments to healthcare professionals and organisations in 37 countries: a European policy review. *BMJ Open* 2021;11(12):e053138. doi: 10.1136/bmjopen-2021-053138
55. Vertex Pharmaceuticals Incorporated. Our Science [Available from: https://www.vrtx.com/our-science/2023.
56. Ozieranski P, Csanádi M, Rickard E, et al. Under-reported relationship: a comparative study of pharmaceutical industry and patient organisation payment disclosures in the UK (2012–2016). *BMJ Open* 2020;10(9):e037351. doi: 10.1136/bmjopen-2020-037351
57. Colombo C, Mosconi P, Villani W, et al. Patient organizations' funding from pharmaceutical companies: is disclosure clear, complete and accessible to the public? An Italian survey. *PLoS One* 2012;7(5):e34974. doi: 10.1371/journal.pone.0034974 [published Online First: 20120509]

For peer review only - http://bmjopen.bmj.com/site/about/guidelines.xhtml

Figure legend

Figure 1. Classification model to determine company interests in patient organisation funding
Note: An interest is when there is, or could be perceived to be, an opportunity for a pharmaceutical company to benefit in the disease area where the patient organisation operates.

Figure 2. Cumulative value of payments by receiving patient organisation type and funding company in 2020
Note: Non-disease-specific patient organisations include organisations that could not be matched to specific ICD-11 codes or could not be classified as rare or non-rare, such as hospital charities, carers organisations, and hospices.

Figure 3. Cumulative value of payments by patient organisation type and therapeutic area from in 2020
Note: Non-disease-specific patient organisations include organisations that could not be matched to specific ICD-11 codes or could not be classified as rare or non-rare, such as hospital charities, carers organisations, and hospices.

For peer review only

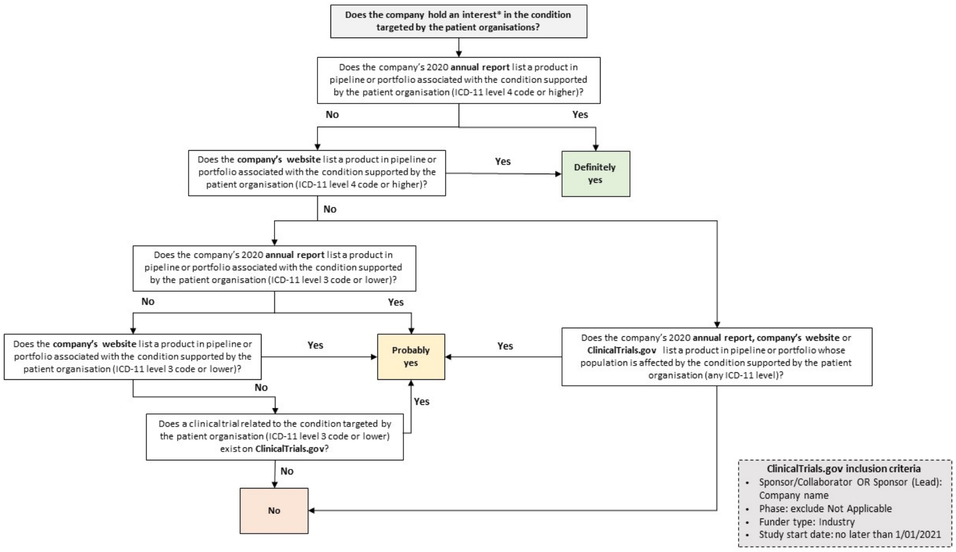

Caption: Classification model to determine company interests in patient organisation funding

Notes: An interest is when there is, or could be perceived to be, an opportunity for a pharmaceutical company to benefit in the disease area where the patient organisation operates.

338x190mm (96 x 96 DPI)

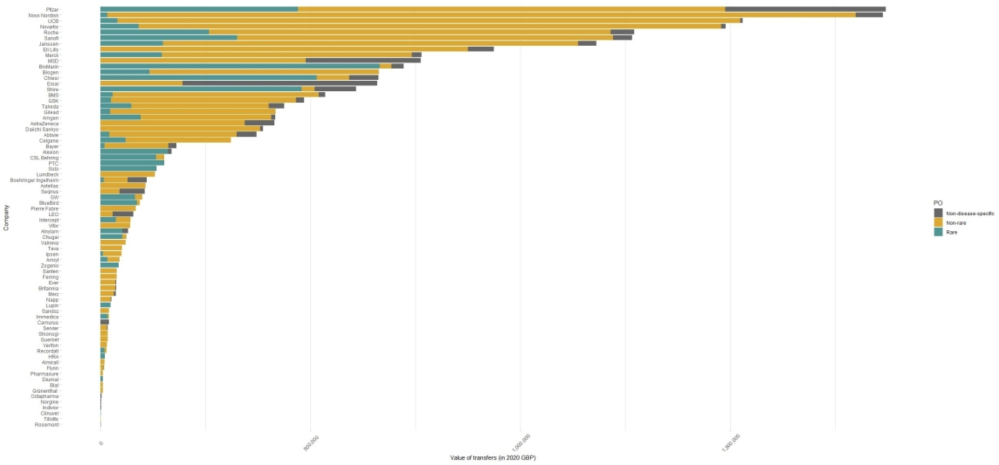

Cumulative value of payments by receiving patient organisation type and funding company in 2020

Note: Non-disease-specific patient organisations include organisations that could not be matched to specific ICD-11 codes or could not be classified as rare or non-rare, such as hospital charities, carers organisations, and hospices.

650x299mm (96 x 96 DPI)

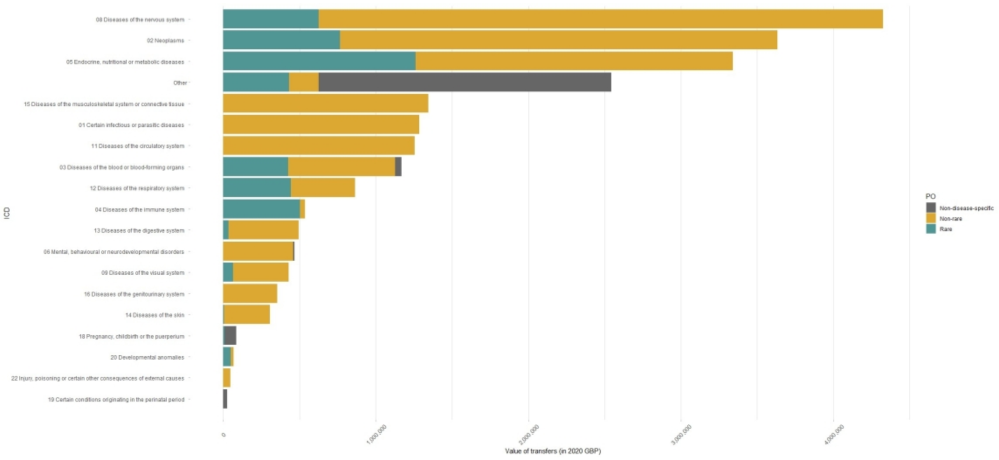

Cumulative value of payments by patient organisation type and therapeutic area from in 2020

Note: Non-disease-specific patient organisations include organisations that could not be matched to specific ICD-11 codes or could not be classified as rare or non-rare, such as hospital charities, carers organisations, and hospices.

581x264mm (96 x 96 DPI)

Supplemental Material

Data collection

Payments

We retrieved data on 2020 payments from pharmaceutical companies to patient organisations from the following sources:

1) **Companies' websites**. Disclosing payments to patient organisations is a requirement of Clause 29 of the Association of British Pharmaceutical Industry (ABPI) Code of Practice.[1] Specifically, the ABPI requires companies to keep a public record of any payment made to patient organisations on their website for a minimum of three years following the payment.[1] Therefore, companies' website were our primary data source on payments to patient organisations.
2) **Disclosure UK HCOs database**. In light of a recent study unveiling that payments to patient organisations were misreported in the Disclosure UK database of payments to healthcare organisations (HCOs),[2] we also screened the 2020 Disclosure UK HCOs database for payments to patient organisations.

If payments were not disclosed in the company's website nor in the Disclosure UK HCOs database, we assumed that the company did not make any payments to patient organisations in 2020, as commonly assumed in the literature.[3]

One investigator (AG) extracted payment disclosures from the companies' websites. These comprised the name of the patient organisation, the year when the payment was made, the reason for the payment and its value in the currency reported by the disclosing company. The 2020 Disclosure UK HCOs database was also screened, and recipients were matched to standardised patient organisations names. To ensure the data's accuracy, the final database was scanned for duplicates, but no such instances were found. All payments were first adjusted for inflation using the ONS Consumer Price Index.[4] When reported in different currencies, such as United States Dollars (USD), Swiss Franc (CHF), Swedish Krona (SEK), Norwegian Krone (NKK) and Danish Krone (DKK), the value of the payment was converted to Great British Pounds (GBP), using the ONS historical yearly conversion rates. [5 6] Two in-kind payments with a monetary value of zero were excluded from the analysis. Further details on variables' cleaning and coding can be found in the Supplemental Material.

Therapeutic areas

Patient organisations' websites were also screened to understand the condition(s) they focused on. For example, in the case of *Blood Cancer UK*, their mission is to "*beat blood cancer*", therefore, the condition supported was coded as blood cancer.

After being identified as described above, conditions were further classified into rare and non-rare.

For peer review only - http://bmjopen.bmj.com/site/about/guidelines.xhtml

Conditions were considered rare if they appeared in the Orphanet database of rare diseases regardless of their classification level (group of disorders, disorders or subtypes of disorders).[7] For example, multiple myeloma appears in the Orphanet database of rare diseases, therefore a patient organisation focusing this condition would be categorised as rare-focused. When condition sub-types appeared in the Orphanet database, the patient organisation's website was screened to check whether its focus was on rare conditions. For example, *Metabolic Support UK's* motto is "*Your rare condition. Our common fight"* and was therefore assumed to be rare disease-focused. Conversely, should a patient organisation focus on a broader condition such as blood cancer with no sole focus on rare conditions, the organisation would be conservatively considered non-rare. While this approach was preferred as it did not require further assumptions, it entails that only more specialised patient organisation are considered as rare. Such approach might have led to the number and overall value of payments from pharmaceutical companies to rare diseases-focused patient organisations being underestimated, as these organisaitons are expected to get less payments than more generalist ones (e.g. multiple myeloma vs blood cancer).

A third category (*unclear)* was created for non-disease-specific patient organisations, such as coalition of charities or organisations focused on palliative care for terminally ill patients. This category was excluded from the main analyses, but sub-group analyses are reported at the end of the Supplemental Material.

Companies' interest

We developed a methodology to assess the extent to which a pharmaceutical company holds an interest in the disease supported by a patient organisation. For the purpose of this analysis, we adapted the definition of interest provided by NICE.[8] An interest is when there is, or could be perceived to be, an opportunity for a pharmaceutical company to benefit in the disease area where the patient organisation operates. This could include situations where the pharmaceutical company has a drug developed or in development for a condition supported by the patient organisation, or where a drug in the company's portfolio or pipeline is restricted to a specific population affected by the disease supported by the patient organisation.

As first step, the conditions supported by patient organisations were translated into ICD-11 codes using the online ICD-11 database.[9]

ICD-11 codes are mutually exclusive, exhaustive and are arranged as a single hierarchical tree. This means that specific diseases are nested within broader classifications. An example for multiple myeloma is shown in Table 1 below.

Table 1. Example of ICD-11 classification, Multiple myeloma

Hierarchy level	Condition	ICD-11 code
Level 1	Neoplasms	2
Level 2	Neoplasms of haematopoietic or lymphoid tissues	2A
Level 3	Mature B-cell neoplasms	2A8
Level 4	Plasma cell neoplasms	2A83
Level 5	Plasma cell myeloma	2A83.1

For peer review only - http://bmjopen.bmj.com/site/about/guidelines.xhtml

In this example, multiple myeloma is nested within *Plasma cell myeloma*, who is in its turn nested within *Plasma cell neoplasms* and so on up to *Neoplasms*.

Subsequently, companies' annual reports, website and the ClinicalTrials.gov database were searched to assess whether the each company had an interest in the condition supported by the patient organisation receiving the payment. The diagram in the main document (Figure 1) schematically illustrates the approach taken to understand whether the company definitely, probably or did not have an interest in the condition. Figure 1 below illustrates the source of companies' interests.

For example, if *Company X* reports in its annual report having a drug in development for multiple myeloma and transferred a sum of money to *Blood Cancer UK*, this would be coded as *probably yes*, as the company has a product in its pipeline or portfolio associated with a condition supported by the patient organisation. In this case, the ICD-11 level would be 2, *Neoplasms of haematopoietic or lymphoid tissue*, under which multiple myeloma is nested. Conversely, should *Company X* have made a payment to *Myeloma UK*, this would have been coded as *definitely yes*, as there is perfect alignment between the condition supported by the patient organisation and by *Company X's* drug.

Situations where a company's interest in a certain condition could not be identified indicate an impossibility of identifying such link, rather than the lack thereof.

Figure 1. Source of companies interests

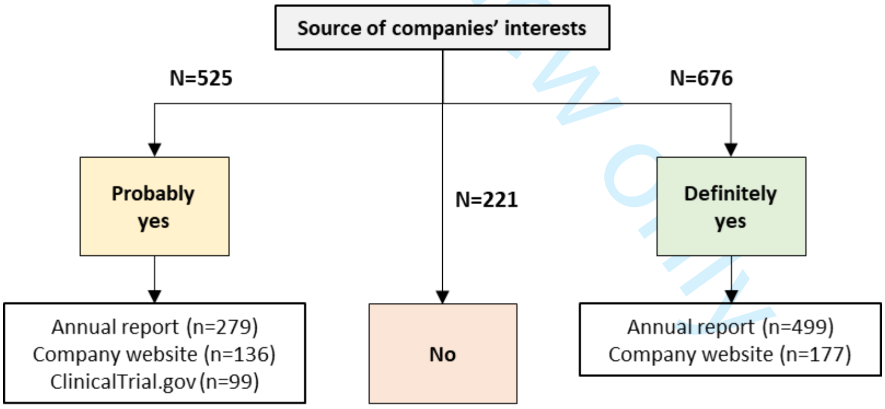

For peer review only - http://bmjopen.bmj.com/site/about/guidelines.xhtml

1 **Variables cleaning and coding**

2 **Table 2. Description of key variables in payment database**

Variables name	Description	Details
**Company**	Standardised company name	Company name as reported on company website and/or on HCOs database.<br>Two mergers involving companies included in our analysis—BMS and Celgene, and Takeda and Shire—were completed prior to 2020. Although these companies had merged, we treated them as separate entities because their disclosures were reported separately even after the acquisition.
**ABPI member**	ABPI membership of company; *source: ABPI full members list*	0 = not ABPI member, 1 = ABPI member
**Company_condition**	Concatenation of company name and disease area targeted by the patient organisation	Concatenation used for coding and analysis purposes
**Company interest**	Whether the company hold an interest* in the condition targeted by the patient organsation	- **Definitely yes**: the company's annual report or website list a product for the condition targeted by the patient organisation in its portfolio/pipeline (ICD-11 level 4 or above)<br>- **Probably yes**: the company's annual report or website list a product for the condition targeted by the patient organisation in its portfolio/pipeline OR a clinical trial for which the company is sponsor is listed for the disease targeted by the patient organisation OR a drug in the company's pipeline/portfolio is restricted to a specific population affected by the disease targeted by the patient organisation (ICD-11 level 3 or below)<br>- **No** : None of the above
**Source**	Source of company interest variable	Annual report, company website, ClinicalTrials.gov, none
**Name of PO**	Name of patient organization as reported by companies in disclosure report	-
**Standardised PO name**	Standardised name of patient organization to avoid duplicates and inconsistencies	For coding purposes, names of patient organisations were standardised. The following steps were taken:<br>1. Patient organisations' names for typos, abbreviations, spelling mistakes and duplicated within the companies' disclosures (e.g. Crohn's & Colitis UK and CCUK would both be standardized to Crohn's and Colitis UK);<br>2. If the patient organisation changed name over time, the latest name on record was used;

For peer review only - http://bmjopen.bmj.com/site/about/guidelines.xhtml

		3. If the two patient organisations merged over the study period, the name of the merged entity was used (e.g. the British Lung Foundation and Asthma UK merged into Asthma + Lung UK);<br>4. Separate entries were made for patient organsiations under the same umbrella but focusing on different geographical entities (e.g. Alzheimer UK vs Alzheimer Scotland)
**Reason for exclusion**	Reason why the organisation was excluded from the analysis	- Not UK organisation (not aligned with geographical scope e.g. Irish, US-based);<br>- For profit company (not aligned with definition of patient organization used in the study);<br>- Missing information (organisations for whose nature is unclear i.e. patient organisation website could not be identified)
**ICD-11**	Classification of disease targeted by the patient organisation according to the WHO ICD-11; *source: ICD WHO website*	General classification (ICD-11 chapters)<br>*See Excel file, Inputs tab*
**Condition**	Condition targeted by patient organisation as reported on website	e.g. Blood Cancer UK would fall under ICD-11 code 02 Neoplasms, with *blood cancer* being the condition
**Charity number (if any)**	Charity number as reported in the organization website or as reported in the England and Wales Charity Commission website	When both England/Wales and Scotland or Northern Ireland charity numbers were provided, the former was chosen. Scotland and Northern Ireland charity numbers were reported only when those for England/Wales were missing
**Company number (if charity number missing)**	Company number as reported in the organization website or as reported in the Government Company Information Service wesbite if the patient organization cannot be found in the charity commission database (e.g. limited by guarantee company)	When both England/Wales and Scotland or Northern Ireland charity numbers were provided, the former was chosen. Scotland and Northern Ireland charity numbers were reported only when those for England/Wales were missing
**Link**	Link of patient organisation website	-
**Rare disease**	Whether the condition or one of the conditions targeted by the patient organisation is considered as rare	A condition was considered as rare if it under at least one of the following criteria:<br>1. The condition is listed in Orphanet list of rare diseases regardless of its ICD-11 level classification;<br>2. In their website, the patient organisation explicitly describe the disease they target as rare (e.g. *Metabolic Support UK's* motto is "*Your rare condition. Our common fight*" and was therefore assumed to be rare disease-focused)

For peer review only - http://bmjopen.bmj.com/site/about/guidelines.xhtml

**Details of payment**	Details of payment as reported by companies in disclosure report	-
**Country**	Country of payment	The country considered for the entire database is the UK
**Year**	Year of payment	2020
**Currency**	Currency of payment	Currency the payment is reported in the disclosure reports (i.e. EUR, GBP, USD, CHF, SEK, NKK)
**Currency_year**	Concatenation of currency and year of payment for conversion purposes	-
**Value of payment**	Value of payment in original currency as reported by companies in disclosure report	In-kind payments were removed from the analysis as no monetary value could be associated to such payments
**Value in 2020 pounds**	GBP converted and inflation adjusted value of payment	See details in *Inputs* sheet

*An interest is when there is, or could be perceived to be, an opportunity for a pharmaceutical company to benefit in the disease area where the patient organisation operates.

For peer review only - http://bmjopen.bmj.com/site/about/guidelines.xhtml

Disclosure details

Table 3. Reporting of payments to patient organizations by pharmaceutical companies: comparison of company websites and Disclosure UK HCOs database

Company	Company website only	HCOs database only	Both
Abbvie	X		
Alexion	X		
Almirall	X		
Alnylam			X
Amgen			X
Amryt	X		
Astellas			X
AstraZeneca			X
BMS			X
Bayer			X
Bial		X	
BioMarin			X
Biogen	X		
BlueBird	X		
Boehringer Ingelheim			X
Britannia			X
CSL Behring	X		
Camurus			X
Celgene			X
Chiesi			X
Chugai	X		
Clinuvel	X		
Daiichi Sankyo			X
Diurnal	X		
Eisai			X
Eli Lilly			X
Ever			X
Ferring		X	
Flynn		X	
GSK			X
GW			X
Gilead		X	
Grünenthal			X
Guerbet		X	
HRA		X	
Immedica	X		
Indivior	X		
Intercept	X		
Ipsen		X	
Janssen			X

For peer review only

**LEO**	X		
**Lundbeck**			X
**Lupin**	X		
**MSD**			X
**Merck**			X
**Merz**			X
**Napp**			X
**Norgine**		X	
**Novartis**			X
**Novo Nordisk**			X
**Octapharma**		X	
**PTC**	X		
**Pfizer**			X
**Pharmasure**		X	
**Pierre Fabre**			X
**Recordati**	X		
**Roche**			X
**Rosemont**			X
**Sandoz**		X	
**Sanofi**			X
**Santen**	X		
**Seqirus**	X		
**Servier**	X		
**Shionogi**		X	
**Shire**			X
**Sobi**	X		
**Takeda**			X
**Teva**		X	
**Tillotts**	X		
**UCB**			X
**Valneva**	X		
**Veriton**		X	
**Vifor**			X
**Zogenix**	X		
**Total (n;%)**	24; 32%	14; 19%	36; 49%

Table 4. Reporting of payments to patient organizations by pharmaceutical companies: payments disclosed on company websites and Disclosure UK HCOs database

Company	Payments reported on company website (£)	Payments reported on HCOs database (£)	Total
**Abbvie**	£ 371,503	£ -	£ 371,503
**Alexion**	£ 168,925	£ -	£ 168,925
**Almirall**	£ 9,775	£ -	£ 9,775
**Alnylam**	£ 51,559	£ 14,050	£ 65,609
**Amgen**	£ 347,757	£ 68,845	£ 416,602

For peer review only - http://bmjopen.bmj.com/site/about/guidelines.xhtml

For peer review only

**Amryt**	£	45,413	£	-	£	45,413
**Astellas**	£	94,583	£	13,071	£	107,654
**AstraZeneca**	£	326,201	£	88,175	£	414,376
**BMS**	£	517,082	£	17,750	£	534,832
**Bayer**	£	171,758	£	9,098	£	180,856
**Bial**	£	-	£	5,500	£	5,500
**BioMarin**	£	411,912	£	310,455	£	722,367
**Biogen**	£	663,142	£	-	£	663,142
**BlueBird**	£	94,000	£	-	£	94,000
**Boehringer Ingelheim**	£	79,762	£	30,000	£	109,762
**Britannia**	£	35,000	£	2,030	£	37,030
**CSL Behring**	£	152,192	£	-	£	152,192
**Camurus**	£	13,168	£	6,500	£	19,668
**Celgene**	£	310,329	£	640	£	310,969
**Chiesi**	£	602,259	£	60,000	£	662,259
**Chugai**	£	62,092	£	-	£	62,092
**Clinuvel**	£	1,000	£	-	£	1,000
**Daiichi Sankyo**	£	57,879	£	329,385	£	387,264
**Diurnal**	£	6,000	£	-	£	6,000
**Eisai**	£	476,271	£	183,207	£	659,478
**Eli Lilly**	£	874,288	£	62,690	£	936,978
**Ever**	£	18,934	£	18,934	£	37,867
**Ferring**	£	-	£	38,000	£	38,000
**Flynn**	£	-	£	8,002	£	8,002
**GSK**	£	325,410	£	159,064	£	484,474
**GW**	£	98,788	£	303	£	99,091
**Gilead**	£	-	£	417,448	£	417,448
**Grünenthal**	£	4,200	£	1,000	£	5,200
**Guerbet**	£	-	£	17,000	£	17,000
**HRA**	£	-	£	10,000	£	10,000
**Immedica**	£	19,954	£	-	£	19,954
**Indivior**	£	1,200	£	-	£	1,200
**Intercept**	£	71,712	£	-	£	71,712
**Ipsen**	£	-	£	50,050	£	50,050
**Janssen**	£	1,170,768	£	10,000	£	1,180,768
**LEO**	£	78,633	£	-	£	78,633
**Lundbeck**	£	89,400	£	40,309	£	129,709
**Lupin**	£	24,000	£	-	£	24,000
**MSD**	£	537,632	£	225,287	£	762,919
**Merck**	£	763,885	£	1,000	£	764,885
**Merz**	£	31,114	£	5,789	£	36,903
**Napp**	£	8,000	£	18,020	£	26,020
**Norgine**	£	-	£	1,240	£	1,240
**Novartis**	£	1,442,037	£	46,812	£	1,488,849
**Novo Nordisk**	£	452,113	£	1,411,598	£	1,863,711

For peer review only - http://bmjopen.bmj.com/site/about/guidelines.xhtml

**Octapharma**	£ -	£ 2,995	£ 2,995
**PTC**	£ 151,433	£ -	£ 151,433
**Pfizer**	£ 1,360,510	£ 509,793	£ 1,870,303
**Pharmasure**	£ -	£ 6,000	£ 6,000
**Pierre Fabre**	£ 50,010	£ 34,096	£ 84,106
**Recordati**	£ 14,500	£ -	£ 14,500
**Roche**	£ 1,169,578	£ 101,395	£ 1,270,973
**Rosemont**	£ 200	£ 200	£ 400
**Sandoz**	£ -	£ 20,000	£ 20,000
**Sanofi**	£ 1,262,802	£ 3,825	£ 1,266,627
**Santen**	£ 38,170	£ -	£ 38,170
**Seqirus**	£ 105,000	£ -	£ 105,000
**Servier**	£ 17,163	£ -	£ 17,163
**Shionogi**	£ -	£ 17,000	£ 17,000
**Shire**	£ 555,244	£ 53,980	£ 609,224
**Sobi**	£ 132,988	£ -	£ 132,988
**Takeda**	£ 420,549	£ 17,270	£ 437,819
**Teva**	£ -	£ 51,410	£ 51,410
**Tillotts**	£ 830	£ -	£ 830
**UCB**	£ 1,493,896	£ 35,378	£ 1,529,274
**Valneva**	£ 59,512	£ -	£ 59,512
**Veriton**	£ -	£ 15,000	£ 15,000
**Vifor**	£ 58,083	£ 12,000	£ 70,083
**Zogenix**	£ 43,625	£ -	£ 43,625
**Total (£;%)**	£18,015,722; 80%	£4,561,593; 20%	£22,577,314; 100%

For peer review only - http://bmjopen.bmj.com/site/about/guidelines.xhtml

Table 5. Companies' commercial interests by ICD-11 codes according to 2020 payments

	ICD-11																		
**Company**	**01**	**02**	**03**	**04**	**05**	**06**	**08**	**09**	**11**	**12**	**13**	**14**	**15**	**16**	**18**	**19**	**20**	**22**	**Other**
**Abbvie**	1	1	0	0	0	0	1	0	0	0	1	1	1	0	0	0	0	0	0
**Alexion**	0	0	1	0	1	0	0	0	0	0	0	0	0	0	0	0	0	0	0
**Almirall**	0	0	0	0	0	0	0	0	0	0	0	1	0	0	0	0	0	0	0
**Alnylam**	0	0	0	0	1	0	0	0	0	0	0	0	0	0	0	0	0	0	0
**Amgen**	0	1	1	0	0	0	0	0	0	0	1	1	1	0	0	0	0	0	0
**Amryt**	0	0	0	0	1	0	0	0	0	0	0	0	0	0	0	0	0	0	0
**Astellas**	0	1	0	0	0	0	0	0	0	0	0	0	0	0	0	0	0	0	0
**AstraZeneca**	0	1	0	0	1	0	0	0	1	0	0	0	0	1	0	0	0	0	0
**BMS**	0	1	0	0	0	0	1	0	1	0	0	0	1	0	0	0	0	0	0
**Bayer**	0	1	0	0	0	0	0	1	1	0	0	0	0	0	0	0	0	0	0
**Bial**	0	0	0	0	0	0	1	0	0	0	0	0	0	0	0	0	0	0	0
**BioMarin**	0	0	1	0	1	0	0	0	0	0	0	0	0	0	0	0	0	0	0
**Biogen**	0	0	0	0	0	0	1	1	0	0	0	0	1	0	0	0	0	0	0
**BlueBird**	0	0	1	0	0	0	1	0	0	0	0	0	0	0	0	0	0	0	0
**Boehringer Ingelheim**	0	0	0	1	0	0	0	0	0	1	0	0	0	0	0	0	0	0	0
**Britannia**	0	0	0	0	0	0	1	0	0	0	0	0	0	0	0	0	0	0	0
**CSL Behring**	1	0	1	1	0	0	0	0	0	0	0	0	0	0	0	0	0	0	0
**Camurus**	0	0	0	0	0	0	0	0	0	0	0	0	0	0	0	0	0	0	0
**Celgene**	0	1	0	0	0	0	0	0	0	0	0	0	0	0	0	0	0	0	0
**Chiesi**	0	0	1	0	1	0	0	0	0	1	0	0	0	0	0	0	0	0	0
**Chugai**	0	0	1	0	0	0	0	0	0	0	0	0	1	0	0	0	0	0	0
**Clinuvel**	0	0	0	0	1	0	0	0	0	0	0	0	0	0	0	0	0	0	0
**Daiichi Sankyo**	0	1	0	0	0	0	0	0	1	0	0	0	0	0	0	0	0	0	0
**Diurnal**	0	0	0	0	1	0	0	0	0	0	0	0	0	0	0	0	0	0	0

For peer review only

**Eisai**	0	1	0	0	0	1	1	0	0	0	0	0	0	0	0	0	0	0	0
**Eli Lilly**	0	1	0	0	1	0	1	0	0	0	0	1	1	0	0	0	0	0	0
**Ever**	0	0	0	0	0	0	1	0	0	0	0	0	0	0	0	0	0	0	0
**Ferring**	0	1	0	0	0	0	0	0	0	0	0	0	0	1	0	0	0	0	0
**Flynn**	0	0	0	0	0	1	0	0	0	0	0	0	0	0	0	0	0	0	0
**GSK**	1	1	0	1	0	0	0	0	1	0	0	0	0	0	0	0	0	0	0
**GW**	0	0	0	0	0	0	0	0	0	0	0	0	0	0	0	0	0	0	0
**Gilead**	1	1	0	0	0	0	0	0	0	0	1	0	0	0	0	0	0	0	0
**Grünenthal**	0	0	0	0	0	0	0	0	0	0	0	0	0	0	0	0	0	0	0
**Guerbet**	0	0	0	0	0	0	0	0	0	0	0	0	0	1	0	0	0	0	0
**HRA**	0	0	0	0	0	0	0	0	0	0	0	0	0	0	0	0	0	0	0
**Immedica**	0	0	0	0	0	0	0	0	0	0	0	0	0	0	0	0	0	0	0
**Indivior**	0	0	0	0	0	0	0	0	0	0	0	0	0	0	0	0	0	0	0
**Intercept**	0	0	0	0	0	0	0	0	0	0	1	0	0	0	0	0	0	0	0
**Ipsen**	0	1	0	0	0	0	1	0	0	0	0	0	0	0	0	0	0	0	0
**Janssen**	1	1	0	0	0	0	0	0	1	0	1	1	0	0	0	0	0	0	0
**LEO**	0	0	0	0	0	0	0	0	1	0	0	1	0	0	0	0	0	0	0
**Lundbeck**	0	0	0	0	0	1	1	0	0	0	0	0	0	0	0	0	0	0	0
**Lupin**	0	0	0	0	0	0	1	0	0	0	0	0	0	0	0	0	0	0	0
**MSD**	1	1	0	0	0	0	0	0	0	0	0	0	0	0	0	0	0	0	0
**Merck**	0	1	0	0	0	0	1	0	0	0	0	0	0	1	0	0	0	0	0
**Merz**	0	0	0	0	0	0	0	0	0	0	0	0	0	0	0	0	0	0	0
**Napp**	0	0	0	0	1	0	0	0	0	0	0	0	0	0	0	0	0	0	0
**Norgine**	0	0	0	0	0	0	0	0	0	0	0	0	0	0	0	0	0	0	0
**Novartis**	0	1	1	0	0	0	1	1	1	0	0	1	1	0	0	0	0	0	0
**Novo Nordisk**	0	0	1	0	1	0	0	0	0	0	0	0	0	0	0	0	1	0	0
**Octapharma**	0	0	0	0	0	0	0	0	0	0	0	0	0	0	0	0	0	0	0
**PTC**	0	0	0	0	0	0	1	0	0	0	0	0	0	0	0	0	0	0	0
**Pfizer**	1	1	1	0	1	0	1	0	1	0	1	0	1	0	0	0	1	0	0
**Pharmasure**	0	0	0	0	0	0	0	0	0	0	0	0	0	1	0	0	0	0	0

For peer review only - http://bmjopen.bmj.com/site/about/guidelines.xhtml

**Pierre Fabre**	0	1	0	0	0	0	0	0	0	0	0	1	0	0	0	0	0	0	0
**Recordati**	0	0	0	0	1	0	0	0	0	0	0	0	0	0	0	0	0	0	0
**Roche**	0	1	0	0	0	0	1	0	0	1	1	0	0	0	1	0	0	0	0
**Rosemont**	0	0	0	0	0	0	0	0	0	0	0	0	0	0	0	0	0	0	0
**Sandoz**	0	0	0	0	0	0	0	0	0	0	0	0	0	0	0	0	0	0	0
**Sanofi**	1	1	1	1	1	0	1	0	1	0	0	1	1	1	0	0	0	0	0
**Santen**	0	0	0	1	0	0	0	1	0	0	0	0	0	0	0	0	0	0	0
**Seqirus**	0	0	0	0	0	0	0	0	0	0	0	0	0	0	0	0	0	0	0
**Servier**	0	1	0	0	0	0	0	0	0	0	0	0	0	0	0	0	0	0	0
**Shionogi**	0	0	0	0	0	0	0	0	0	0	0	0	0	0	0	0	0	0	0
**Shire**	0	0	1	1	1	1	0	0	0	0	0	0	0	0	0	0	0	0	0
**Sobi**	0	1	1	0	1	0	0	0	0	0	0	0	0	0	0	0	0	0	0
**Takeda**	0	1	0	0	1	0	0	0	0	0	1	0	0	0	0	0	0	0	0
**Teva**	0	0	0	0	0	0	0	0	0	0	0	0	0	0	0	0	0	0	0
**Tillotts**	0	0	0	0	0	0	0	0	0	0	1	0	0	0	0	0	0	0	0
**UCB**	0	0	1	0	0	0	1	0	0	0	0	1	1	0	0	0	0	0	0
**Valneva**	1	0	0	0	0	0	0	0	0	0	0	0	0	0	0	0	0	0	0
**Veriton**	0	0	0	0	0	0	0	0	0	0	0	0	0	0	0	0	0	0	0
**Vifor**	0	0	0	0	0	0	0	0	0	0	0	0	0	0	0	0	0	0	0
**Zogenix**	0	0	0	0	0	0	1	0	0	0	0	0	0	0	0	0	0	0	0

Notes: This table reflects whether companies had a definite or probable interest in the ICD-11 code based on their pipeline or portfolio (1 = yes, 0 = no). Please note that companies' interests were opportunistically screened only in disease areas where they made a payment to a specific patient organisation, and therefore this table should not be considered exhaustive. The table refers payments made in 2020 only.

Legend: 01 Certain infectious or parasitic diseases; 02 Neoplasms; 03 Diseases of the blood or blood-forming organs; 04 Diseases of the immune system; 05 Endocrine, nutritional or metabolic diseases; 06 Mental, behavioural or neurodevelopmental disorders; 08 Diseases of the nervous system; 09 Diseases of the visual system; 11 Diseases of the circulatory system; 12 Diseases of the respiratory system; 13 Diseases of the digestive system; 14 Diseases of the skin; 15 Diseases of the musculoskeletal system or connective tissue; 16 Diseases of the genitourinary system; 18 Pregnancy, childbirth or the puerperium; 19 Certain conditions originating in the perinatal period; 20 Developmental anomalies; 22 Injury, poisoning or certain other consequences of external causes; Other. Other indicates disease areas where patient organisations operate that could not be classified as any ICD-11 codes.

For peer review only - http://bmjopen.bmj.com/site/about/guidelines.xhtml

Table 6. List of patient organisations receiving payments in 2020

Standardised name	Charity number	Link
Acacia Mews Care Home	1174346	https://www.nhs.uk/services/Careproviders/Overview/DefaultView.aspx?id=47011
Action Bladder Cancer UK	1164374	https://actionbladdercanceruk.org/
Action for Pulmonary Fibrosis	1152399	https://www.actionpf.org/
Action On Pre-Eclampsia	1013557	https://action-on-pre-eclampsia.org.uk/
Action on Smoking and Health - Wales	1120834	https://ash.wales/
Action Duchenne	1101971	https://www.actionduchenne.org/
Adfam	1067428	https://adfam.org.uk/
Africa Advocacy Foundation	1164778	https://www.africadvocacy.org/
African-Caribbean Leukaemia Trust	1119516	https://aclt.org/
Age UK	1128267	https://www.ageuk.org.uk/
Alex - The Leukodystrophy Charity	1106008	https://www.alextlc.org/
ALK Positive Lung Cancer	1181171	https://www.alkpositive.org.uk/
Alkaptonuria Society	1101052	https://akusociety.org/
Allergy UK	1094231	https://www.allergyuk.org/
Alliance for Heart Failure	N/A	https://allianceforheartfailure.org/
Alzheimer Scotland	SC022315	https://www.alzscot.org/
Alzheimer's Support	1048314	https://www.alzheimerswiltshire.org.uk/
Alzheimer's Research UK	1077089	https://www.alzheimersresearchuk.org/
Alzheimer's Society	296645	https://www.alzheimers.org.uk/
Amyloidosis Patients Association	1183624	https://register-of-charities.charitycommission.gov.uk/charity-details/?regid=1183624&subid=0
Anthony Nolan	803716	https://www.anthonynolan.org/
Anticoagulation UK	1090250	https://register-of-charities.charitycommission.gov.uk/charity-details/?regid=1090250&subid=0
AOFAC Foundation	1162155	https://aofacfoundation.org/
Aplastic Anaemia Trust	1107539	https://www.theaat.org.uk/
APS Support UK	1138116	https://aps-support.org.uk/
Arthritis and Musculoskeletal Alliance	1108851	http://arma.uk.net/
Aspens	1171446	https://www.aspens.org.uk/
Association for Glycogen Storage Disease	1132271	https://agsd.org.uk/
Asthma + Lung UK	326730	https://www.asthma.org.uk/
Astriid	1176645	https://astriid.org/
Atrial Fibrillation Association	1122442	Supporting children terminally ill
Axial Spondylitis International Federation	1173902	https://asif.info/
Baby Lifeline	1006457	https://www.babylifeline.org.uk/
Bath Institute for Rheumatic Diseases	1040650	https://www.birdbath.org.uk/

For peer review only - http://bmjopen.bmj.com/site/about/guidelines.xhtml

Batten Disease Family Association	1084908	http://www.bdfa-uk.org.uk/
Bipolar UK	293340	https://www.bipolaruk.org/
Bladder Health UK	1149973	https://bladderhealthuk.org/
Bliss	1002973	https://www.bliss.org.uk/
Blood Cancer Alliance	N/A	https://www.bloodcanceralliance.org/
Blood Cancer UK	216032	https://bloodcancer.org.uk/
BME Cancer Communities	1182806	https://www.bmecancer.com/
Bowel Cancer UK	1071038	https://www.bowelcanceruk.org.uk/
Brains Trust	1114634	https://brainstrust.org.uk/
Breast Cancer Haven (The Haven)	3291851	https://www.breastcancerhaven.org.uk/
Breast Cancer Now	1160558	https://breastcancernow.org/
British Association ofr the Study of the Liver	1106320	https://www.basl.org.uk/
British Heart Foundation	225971	https://www.bhf.org.uk/
British Inherited Metabolic Disease Group	1184024	https://www.bimdg.org.uk/site/index.asp
British Liver Trust	298858	https://britishlivertrust.org.uk/
British Paediatric Neurology Association	1159115	https://bpna.org.uk/
British Porphyria Association	1089609	http://porphyria.org.uk/
British Skin Foundation	1171373	https://www.britishskinfoundation.org.uk/
British Society for Heart Failure	1075720	https://www.bsh.org.uk/
British Society of Echocardiography	1093808	https://www.bsecho.org/
British Thyroid Foundation	1006391	https://www.btf-thyroid.org/
Cambridge Rare Disease Network	1166365	https://www.camraredisease.org/
Cancer 52	7994413	https://www.cancer52.org.uk/
Cancer Black Care	1086465	https://www.cancerblackcare.org.uk/
Cancer Focus Northern Ireland	101307	https://cancerfocusni.org/
Cancer Research UK	1089464	https://www.cancerresearchuk.org/
Cancer Support Scotland	SC012867	https://www.cancersupportscotland.org/
Cancer Support UK	1105703	https://cancersupportuk.org/
CancerCare	1120048	https://cancercare.org.uk/
Cara Trust	328124	https://www.madtrust.org.uk/project/the-cara-trust/
Cardiomyopathy UK	1164263	https://www.cardiomyopathy.org/
Carers UK	N/A	https://www.carersuk.org/
Changing Faces	1011222	https://www.changingfaces.org.uk/
Child Growth Foundation	1172807	https://childgrowthfoundation.org/
Childhood Trust	1154032	https://www.childhoodtrust.org.uk/
Children’s Cancer and Leukaemia Group	1182637	https://www.cclg.org.uk/
Children’s HIV Association	1122356	https://www.chiva.org.uk/
Children’s Trust	288018	https://www.thechildrenstrust.org.uk/
Children's Burns Trust	1082084	https://www.cbtrust.org.uk/

For peer review only - http://bmjopen.bmj.com/site/about/guidelines.xhtml

Cholangiocarcinoma Charity	1091915	https://ammf.org.uk/
Chronic Lymphocytic Leukaemia Support Association	1178482	https://www.cllsupport.org.uk/
Coalition for Life-Course Immunisation	1182662	https://www.cl-ci.org/
Confederation of Meningitis Organisations	1091105	https://www.comomeningitis.org/
Contact a Family	284912	https://contact.org.uk/
Crohn's and Colitis UK	1117148	https://www.crohnsandcolitis.org.uk/
Cystic Fibrosis Trust	1079049	https://www.cysticfibrosis.org.uk/
Dementia UK	1039404	https://www.dementiauk.org/
Dementia Club UK	1168397	https://dementiaclubuk.org.uk/
Diabetes UK	215199	https://www.diabetes.org.uk/
Diana Award	1117288	https://diana-award.org.uk/
DMD Pathfinders	1155884	https://www.pathfindersalliance.org.uk/
Down Syndrome International	1091843	https://www.ds-int.org/
Downs Syndrome Association	1061474	https://www.downs-syndrome.org.uk/
Dravet Syndrome UK	1128289	https://www.dravet.org.uk/
DrugFAM	1123316	https://www.drugfam.co.uk/#
Duchenne UK	1147094	https://www.duchenneuk.org/
Dystonia UK	1062595	https://www.dystonia.org.uk/
East North Hertfordshire NHS Trust	1053338	https://www.enherts-tr.nhs.uk/
East Sussex Healthcare NHS Trust	1058599	https://www.esht.nhs.uk/
Ecancer	1176307	https://ecancer.org/en/
Eczema Outreach Support	SC042392	https://www.eos.org.uk/
Encephalitis Society	1087843	https://www.encephalitis.info/
Epilepsy Action	234343	https://www.epilepsy.org.uk/?gclid=CjwKCAiAsNKQBhAPEiwAB-I5zXsMWEMg1x_J-blYzK3HQGZujp-zoejjkEA_sYpKqYxct5LuE_sV6hoC1t8QAvD_BwE
Epilepsy Consortium Scotland	N/A	http://www.epilepsyconsortiumscotland.co.uk/
Epilepsy Research UK	1100394	https://epilepsyresearch.org.uk/
Epilepsy Scotland	SC000067	https://www.epilepsyscotland.org.uk/
Epilepsy Society	206186	https://epilepsysociety.org.uk/
Errol Mckellar Foundation	1181574	https://www.theerrolmckellarfoundation.com/
European Parkinson’s Disease Association	1163211	https://www.epda.eu.com/
Eve Appeal	1091708	https://eveappeal.org.uk/
Familial Hypercholesterolaemia Network	1170731	https://fheurope.org/
FareShare	1100051	https://fareshare.org.uk/
Favor UK	N/A	https://www.facesandvoicesofrecoveryuk.org/

For peer review only - http://bmjopen.bmj.com/site/about/guidelines.xhtml

Fertility Network UK	1099960	https://fertilitynetworkuk.org/
Fight Bladder Cancer	1157763	https://www.fightbladdercancer.co.uk/
Fight for Sight UK	1111438	https://www.fightforsight.org.uk/
Findacure	1149646	https://www.rarebeacon.org/about-us/our-journey/
Gauchers Association	1095657	https://www.gaucher.org.uk/
Gene People	1141583	https://genepeople.org.uk/
Genetic Alliance UK	1114195	https://geneticalliance.org.uk/
GetYourBellyOut	11276246	https://getyourbellyout.org.uk/
GIST Cancer UK	1129219	https://www.gistcancer.org.uk/
Global Action on Men's Health	1183428	https://gamh.org/
GO Girls	1179108	https://www.gogirlssupport.org/
Gorlin Syndrome Group	1197282	https://gorlingroup.org/
Guts UK	1137029	https://gutscharity.org.uk/
Haemachromatosis UK	1001307	https://www.haemochromatosis.org.uk/
Haemophilia Scotland	SC044298	https://haemophilia.scot/
Haemophilia Society	288260	https://haemophilia.org.uk/
Headway East London	1083910	https://headwayeastlondon.org/
Heart UK	1003904	https://www.heartuk.org.uk/
Heartburn Cancer UK	1136413	https://www.heartburncanceruk.org/
Helen & Douglas House	1085951	https://www.helenanddouglas.org.uk/
Hepatitis C Coalition	N/A	http://www.hepc-coalition.uk/
Hepatitis C Trust	1104279	http://hepctrust.org.uk/
Hereditary Angioedema UK	1152591	https://www.haeuk.org/
Hidradenitis Suppurativa Trust	1177819	https://painuk.org/members/charities/hidradenitis-suppurativa-trust/
Histiocytosis UK	1158789	https://www.histiouk.org/
HIV i-Base	1081905	https://i-base.info/
HIV Scotland	SC033951	https://www.hiv.scot/
Human Story Theatre	1173504	https://humanstorytheatre.com/about-us/
Huntington's Disease Association	296453	https://www.hda.org.uk/
Huntington's Disease Youth Organization	1145781	https://en.hdyo.org/
Immune Deficiency Patient Group of Wales	N/A	https://www.facebook.com/tommy.browne.idpgw/
Immune Thrombocytopenia Support Association	1064480	https://www.itpsupport.org.uk/index.php/en/
Independent Cancer Patients' Voice	1138456	http://www.independentcancerpatientsvoice.org.uk/
Intensive Care Society	1039236	https://www.ics.ac.uk/
International Alliance of Patients' Organizations	1155577	https://www.iapo.org.uk/
International Brain Tumour Alliance	N/A	https://theibta.org/
International Gaucher Alliance	6653373	https://gaucheralliance.org/home
International Headache Society	1042574	https://ihs-headache.org/en/
International Longevity Centre UK	1080496	https://ilcuk.org.uk/

For peer review only - http://bmjopen.bmj.com/site/about/guidelines.xhtml

International Niemann-Pick Disease Alliance	1150256	https://www.inpda.org/
International Patient Organisation for Primary Immunodeficiencies	1058005	https://ipopi.org/
Invisible Cafe	N/A	https://theinvisiblecafe.co.uk/
Isabel Hospice Limited	1046826	https://www.isabelhospice.org.uk/
Jo's Cervical Cancer Trust	1133542	https://www.jostrust.org.uk/
Juvenile Diabetes Research Foundation	295716	https://jdrf.org.uk/
Karen Clifford Skcin cancer charity	1150048	https://www.skcin.org/
Kent Autistic Trust	801965	https://www.kentautistictrust.org/
Kent MS Therapy Centre	801382	https://kentmstc.org.uk/
Kidney Cancer Support Network	1164238	https://actionkidneycancer.org/
Kidney Cancer UK	1120146	https://www.kcuk.org.uk/
Kidney Care UK	270288	https://www.kidneycareuk.org/
Kidney Research UK	252892	https://www.kidneyresearchuk.org/
Leukaemia CARE	1183890	https://www.leukaemiacare.org.uk/
Leukaemia UK	1154856	https://www.leukaemiauk.org.uk/
Liver4Life	1152618	https://www.liver4life.org.uk/
Lupus UK	1051610	https://www.lupusuk.org.uk/
Lymphoma Action	1068395	https://lymphoma-action.org.uk/about-us
Macmillan Cancer Support	261017	https://www.macmillan.org.uk/
Macular Society	2177039	https://www.macularsociety.org/
Maggie's Centres	SC024414	https://www.maggies.org/
Maypole Project	1120163	https://www.themaypoleproject.co.uk/
MDS UK Support Group	1145214	https://mdspatientsupport.org.uk/
Meath Epilepsy Charity	200359	https://www.meath.org.uk/
Medics 4 Rare Diseases	1183996	https://www.m4rd.org/history/
Melanoma Focus	1124716	https://melanomafocus.org/
Melanoma Fund	1085969	https://www.melanoma-fund.co.uk/
Melanoma UK	1157635	https://www.melanomauk.org.uk/
Memorylane Eastbourne	1163541	https://www.memorylaneeastbourne.co.uk/
Meningitis Now	803016	https://www.meningitisnow.org/
Meningitis Research Foundation	1091105	https://www.meningitis.org/
Menopause Support	N/A	https://menopausesupport.co.uk/
Mental Health UK	1170815	https://mentalhealth-uk.org/
Mersey Region Epilepsy Association	504366	https://www.epilepsymersey.org.uk/
Mesothelioma UK	1177039	https://www.mesothelioma.uk.com/
Metabolic Support UK	1089588	https://www.metabolicsupportuk.org/
Migraine Trust	1081300	https://migrainetrust.org/
Motor Neurone Disease Association	294354	https://www.mndassociation.org/
Mouth Cancer Foundation	1109298	https://www.mouthcancerfoundation.org/
MPN Voice	1160316	https://www.mpnvoice.org.uk/

For peer review only - http://bmjopen.bmj.com/site/about/guidelines.xhtml

Multiple Sclerosis International Federation	1105321	https://www.msif.org/
Multiple Sclerosis Society UK	1139257	https://www.mssociety.org.uk/
Multiple Sclerosis Therapy Centres	1031690	https://www.msntc.org.uk/
Multiple Sclerosis Trust	1088353	https://mstrust.org.uk/
Muscular Dystrophy UK	205395	https://www.musculardystrophyuk.org/
My Name'5 Doddie Foundation	SC047871	https://www.myname5doddie.co.uk/
Myeloma UK	SC026116	https://www.myeloma.org.uk/
National AIDS Map	1011220	https://www.aidsmap.com/
National AIDS Trust	297977	https://www.nat.org.uk/
National Attention Deficit Disorder Information and Support Service	N/A	https://www.nhs.uk/services/service-directory/the-national-attention-deficit-disorder-information-and-support-service-addiss/N10498901
National Axial Spondyloarthritis Society	1183175	https://nass.co.uk/
National Cancer Research Institute	1160609	https://www.ncri.org.uk/
National Eczema Society	1009671	https://eczema.org/
National Federation of Prostate Cancer Support Groups	1163152	https://tackleprostate.org/
National Kidney Federation	1106735	https://www.kidney.org.uk/
National Rheumatoid Arthritis Society	1134859	https://nras.org.uk/
National Voices	1057711	https://www.nationalvoices.org.uk/
NAZ	1014056	https://www.naz.org.uk/
Neuroendocrine Cancer UK	1092386	https://www.neuroendocrinecancer.org.uk/
Neurological Alliance	1039034	https://www.neural.org.uk/
New Life Counselling	NI005568	https://www.amh.org.uk/
NHS Charities Together	1186569	https://nhscharitiestogether.co.uk/
Nicole & Jessica Rich Foundation	N/A	https://thenicolerichfoundation.org.uk/
Niemann-Pick UK	1144406	https://www.npuk.org/
North Bristol NHS Trust	1055900	https://www.nbt.nhs.uk/
Oral Health Foundation	263198	https://www.dentalhealth.org/
Orchid	1080540	https://orchid-cancer.org.uk/
Osteoporosis Dorse	1023507	https://www.osteodorset.org.uk/
Ovacome	1159682	https://www.ovacome.org.uk/
Ovarian Cancer Action	1109743	https://ovarian.org.uk/
Over the Wall	1075361	https://www.otw.org.uk/
Pain Concern	SC023559	https://painconcern.org.uk/
Pancreatic Cancer Action	1137689	https://pancreaticcanceraction.org/
Pancreatic Cancer UK	1112708	https://www.pancreaticcancer.org.uk/
Parathyroid UK	N/A	https://parathyroiduk.org/
Parkinson’s UK	258197	https://www.parkinsons.org.uk/
Patient Information Forum	N/A	https://pifonline.org.uk/
Patients Association	1006733	https://www.patients-association.org.uk/

For peer review only - http://bmjopen.bmj.com/site/about/guidelines.xhtml

Patients On Intravenous and Nasogastric Nutrition Therapy	1157655	https://pinnt.com/Home.aspx
Paula Carr Diabetes Trust	801596	https://www.paulacarrdiabetestrust.co.uk/
PBC Foundation UK	SC025619	https://www.pbcfoundation.org.uk/
Pilgrims Hospice	293968	https://www.pilgrimshospices.org/
Pituitary Foundation	1058968	https://www.pituitary.org.uk/
Platelet Society	1172202	https://plateletsociety.co.uk/
Police Community Clubs of Great Britain	N/A	https://www.policecommunityclubs.org/
Polycystic Kidney Disease Charity	1160970	https://pkdcharity.org.uk/
Pompe Support Network	1186383	https://pompe.uk/
Positively UK	1007685	https://positivelyuk.org/
Primary Immunodeficiency UK	1193166	http://www.immunodeficiencyuk.org/
Progress Educational Trust	1139856	https://www.progress.org.uk/
Progressive Supranuclear Palsy Association	1037087	https://pspassociation.org.uk/
Prostate Cancer UK	1005541	https://prostatecanceruk.org/
Psoriasis Association	1180666	https://www.psoriasis-association.org.uk/
Pulmonary Hypertension Association UK	1120756	https://www.phauk.org/
Pumping Marvellous Foundation	1151848	https://www.pumpingmarvellous.org/
Rain Trust	N/A	https://www.nhs.uk/services/service-directory/rain-trust/N10972097
Rainbow Trust Children's Charity	1070532	https://www.rainbowtrust.org.uk/
Rapid Effective Assistance For Children With Potentially Terminal Illness	802440	https://reactcharity.org/
Red Rose Recovery	1152474	https://redroserecovery.org.uk/
Release	801118	https://www.release.org.uk/
Rethink Mental Illness	271028	https://www.rethink.org/
Retina UK	1153851	https://retinauk.org.uk/about/
Revive Multiple Sclerosis Support	SC022886	https://www.revivemssupport.org.uk/
Roy Castle Lung Cancer Foundation	1046854	https://roycastle.org/
Royal Free Charity	1165672	https://royalfreecharity.org/
Royal National Institute of Blind People	226227	https://www.rnib.org.uk/
Royal Osteoporosis Society	1102712	https://theros.org.uk/
Ruth Strauss Foundation	1183221	https://ruthstraussfoundation.com/
Salivary Gland Cancer UK	1182762	https://www.salivaryglandcancer.uk/
SANE	296572	https://www.sane.org.uk/
Sarcoma UK	1139869	https://sarcoma.org.uk/
Scleroderma and Raynauds UK	1161828	https://www.sruk.co.uk/
Scottish Drugs Forum	SC008075	https://www.sdf.org.uk/

For peer review only - http://bmjopen.bmj.com/site/about/guidelines.xhtml

Scottish Families Affected by Alcohol & Drugs	N/A	https://www.sfad.org.uk/
Scottish Huntington's Association	SC010985	https://hdscotland.org/
Shift.MS	1117194	https://shift.ms/
Shine Cancer Support	1146902	https://shinecancersupport.org/
Sickle Cell Society	1046631	https://www.sicklecellsociety.org/
Skin Conditions Campaign Sco tland	SC030004	https://www.disabilityscot.org.uk/organisat ion/skin-conditions-campaign-scotland/
Society for Mucopolysaccharide Diseases	1143472	https://www.mpssociety.org.uk/
Somerville Foundation	1138088	https://sfhearts.org.uk/
Sophia Forum	1131629	https://sophiaforum.net/
Spinal Muscular Atrophy Support UK	1106815	https://smauk.org.uk/
St Elizabeths Centre	1176777	https://www.stelizabeths.org.uk/
Stroke Association	211015	https://www.stroke.org.uk/
Swallows Head and Neck Cancer Charity	1149794	https://www.theswallows.org.uk/
Target Ovarian Cancer	1125038	https://targetovariancancer.org.uk/
Tenovus Cancer Care	1054015	https://www.tenovuscancercare.org.uk/
Terrence Higgins Trust	288527	https://www.tht.org.uk/
Thrombosis UK	1090540	https://thrombosisuk.org/news/post.php?s=2021-10-11-thrombosis-uk-winner-of-activity-of-the-year-award-2021
Tiny Tickers	1078114	https://www.tinytickers.org/
Together for Short Lives	1144022	https://www.togetherforshortlives.org.uk/
TRACTion Cancer Support	SCO048145	https://www.tractioncancersupport.org/
Trekstock	1132421	https://www.trekstock.com/
Trevi	1075433	https://trevi.org.uk/
Tuberous Sclerosis Association	1039549	https://tuberous-sclerosis.org/
Turner Syndrome Support Society	1080507	https://tss.org.uk/
Twins Trust	1076478	https://twinstrust.org/
UK Breast Cancer Group	1177296	https://ukbcg.org/
UK Lung Cancer Coalition	N/A	https://www.uklcc.org.uk/
UK Primary Immune-deficiency Patient Support	1148789	https://ukpips.org.uk/
UK Thalassaemia Society	275107	https://ukts.org/
University of Newcastle Institute of Neuroscience	N/A	https://www.ncl.ac.uk/medical-sciences/research/research-themes/neuroscience/
Urology Cancer Research and Education	1120887	http://www.ucare-oxford.org.uk/
Versus Arthritis	207711	https://www.versusarthritis.org/
Waldenstrom's Macroglobulinaemia UK	1187121	https://wmuk.org.uk/
White Chapel Mission	227905	https://whitechapel.org.uk/
Working with Cancer	9092152	https://workingwithcancer.co.uk/
Young Epilepsy	311877	https://www.youngepilepsy.org.uk/

For peer review only - http://bmjopen.bmj.com/site/about/guidelines.xhtml

Inclusion/exclusion of patient organisations

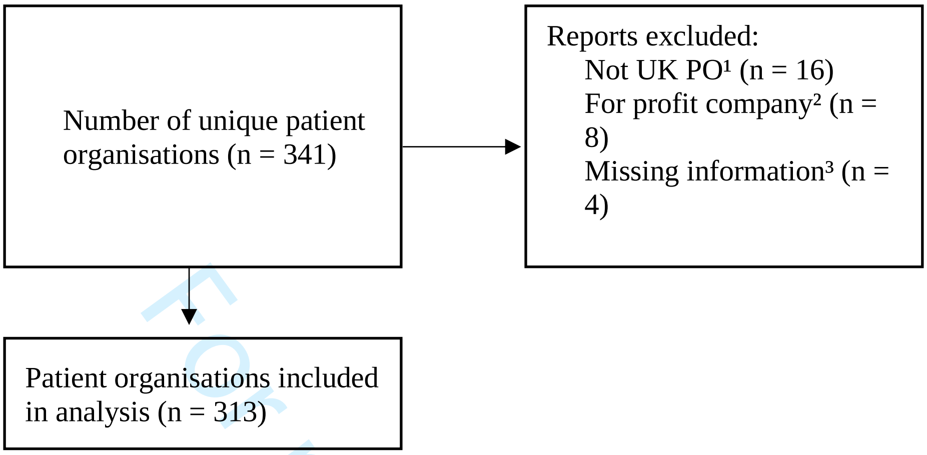

[1]Not aligned with geographical scope e.g. Irish, US-based

[2]Not aligned with EFPIA's definition of patient organisation

[3]Organisations for whose nature is unclear i.e. patient organisation website could not be identified

For peer review only

Additional tables and figures

Figure 2. Histogram of unique companies funding patient organisations in 2020, broken down by rarity of disease

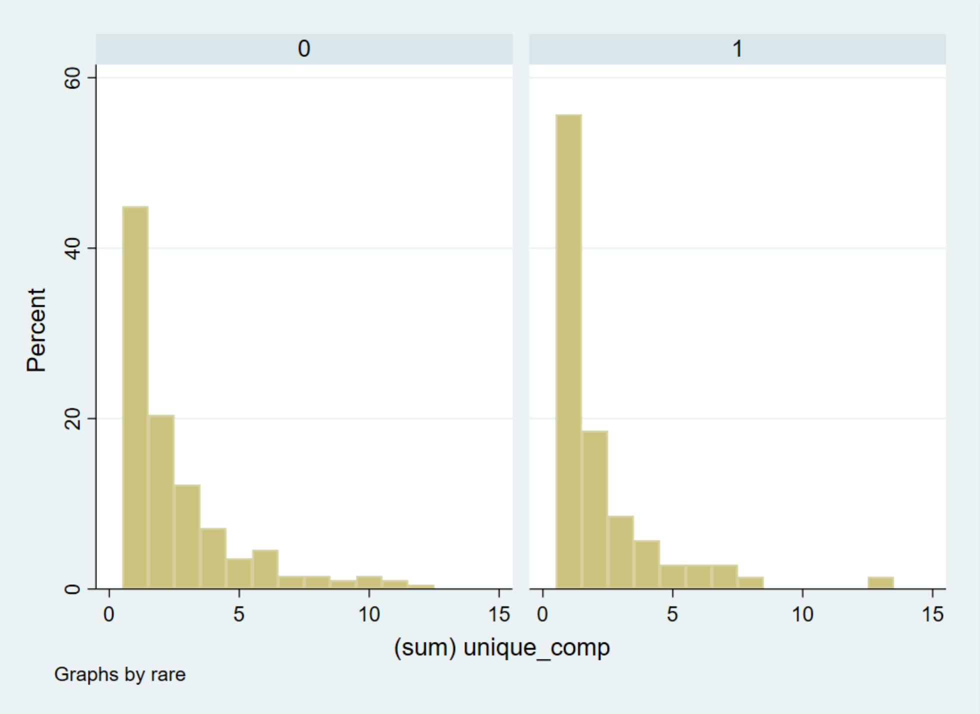

Figure 3. Histogram of share of overall industry funding to patient organisations coming from each contributing company in 2020, broken down by rarity of disease

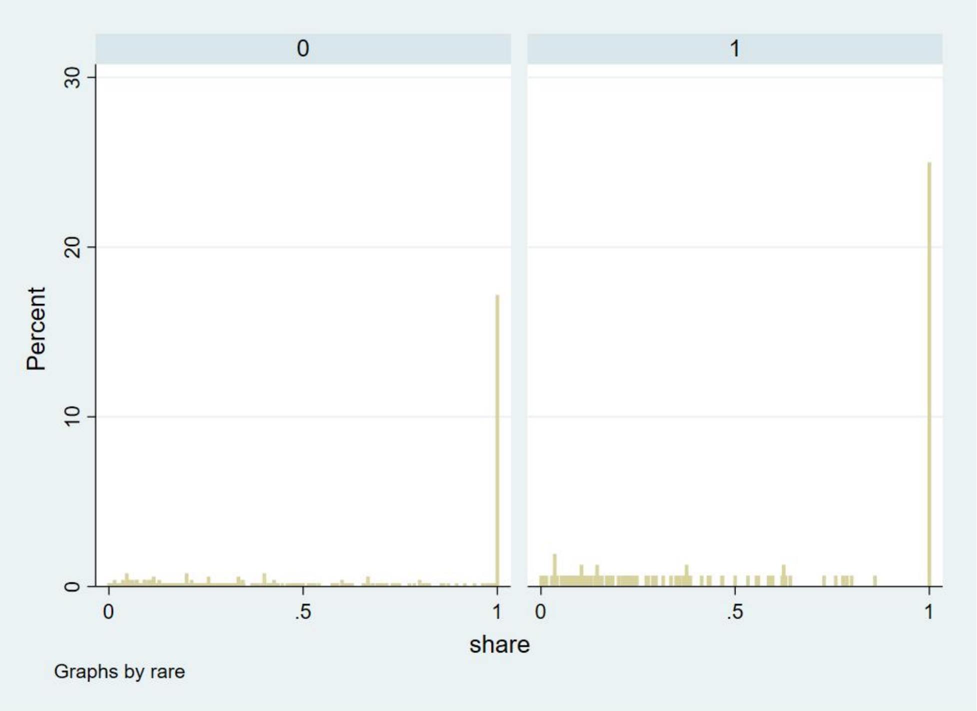

For peer review only - http://bmjopen.bmj.com/site/about/guidelines.xhtml

Figure 4. Histogram of share of industry funding of each organisation comprised by the single highest payment in 2020, broken down by rarity of disease

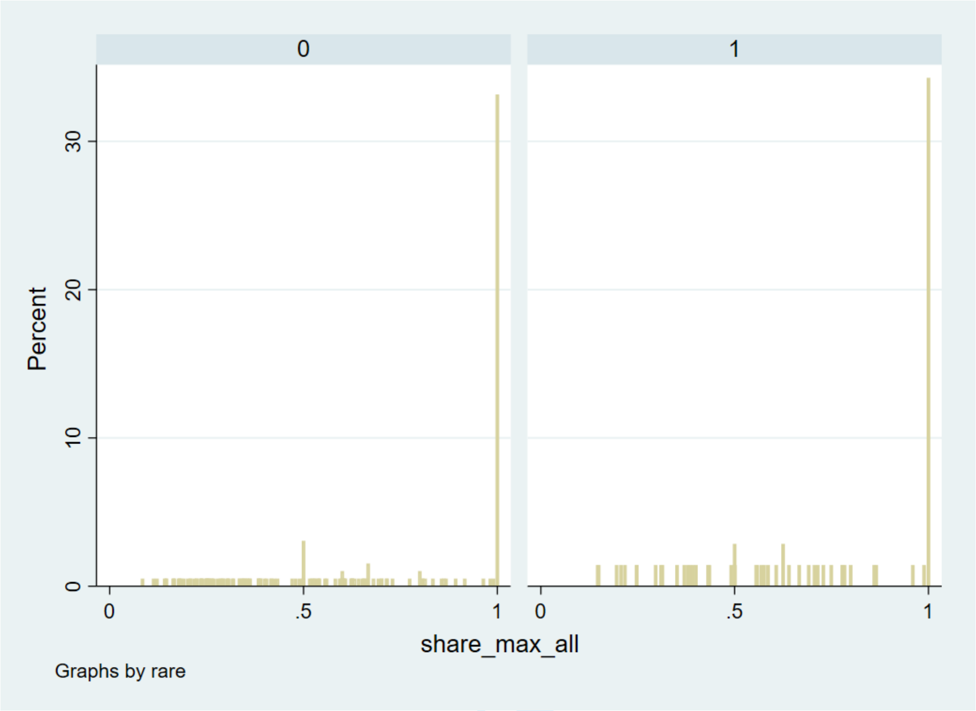

For peer review only - http://bmjopen.bmj.com/site/about/guidelines.xhtml

Sub-group analyses

Excluded patient organisations

66 payments made 28 to patient organisaitons were excluded from the analysis as they did not match EFPIA's definition of *"not-for-profit organisations, mainly composed of patients and/or caregivers, that represent and/or support the needs of patients and/or caregivers"*.

Figure 5 illustrates the reasons for patient organisations exclusion. Most of the excluded patient organisations were for profit organisations (47%; n=31), followed by not UK-based (42%; n=28) and organisations for which no information could be found online (11%; n=7).

Non-UK patient organisations mostly comprised international alliances of patient organisations, European or Irish organisations. We classified organisations as for-profit if they appeared in the UK government repository of companies[1] as *private limited companies.* Care homes, consultancies and rehabilitation clinics were the most prominent in this category.

Overall, payments to excluded patient organisations amounted to £869,677, about 4% of the included payments (Figure 6).

Figure 5. Excluded patient organisations by reason of exclusion

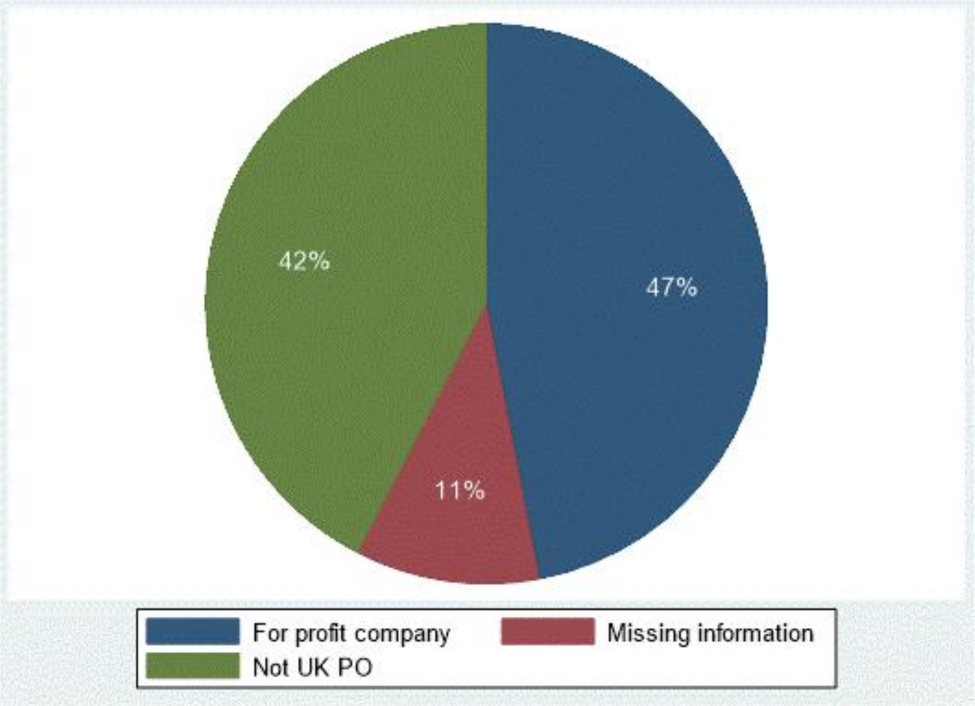

[1] https://find-and-update.company-information.service.gov.uk/

For peer review only - http://bmjopen.bmj.com/site/about/guidelines.xhtml

1 **Figure 6. Payments to included and excluded patient organisations**

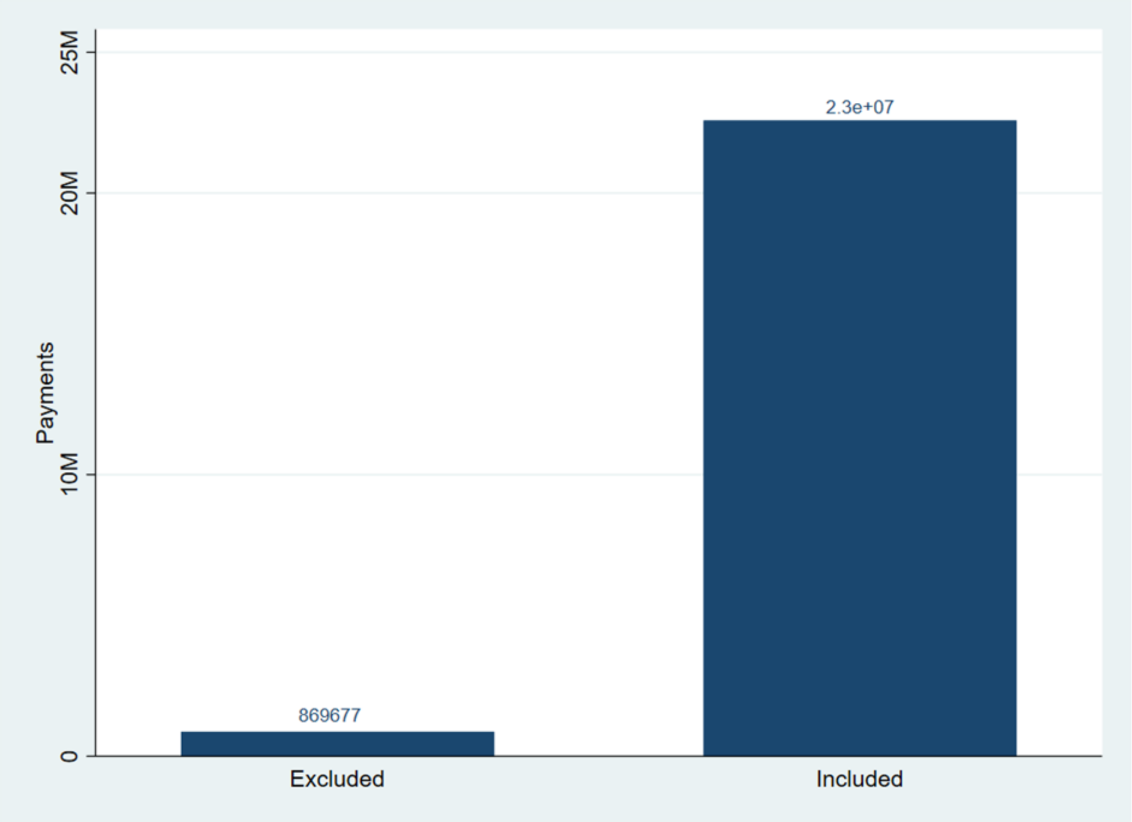

2

3

For peer review only

For peer review only - http://bmjopen.bmj.com/site/about/guidelines.xhtml

References

1. PMCPA. ABPI Code of Practice 2021 [Available from: https://www.pmcpa.org.uk/the-code/2021-interactive-abpi-code-of-practice/.
2. Rickard E, Carmel E, Ozieranski P. Comparing pharmaceutical company payments in the four UK countries: a cross-sectional and social network analysis. *BMJ Open* 2023;13(3):e061591. doi: 10.1136/bmjopen-2022-061591
3. Ozieranski P, Rickard E, Mulinari, Shai. Exposing drug industry funding of UK patient organisations. *BMJ* 2019;365:l1806. doi: 10.1136/bmj.l1806
4. ONS. Consumer price inflation time series: Office for National Statistics; 2022 [Available from: https://www.ons.gov.uk/economy/inflationandpriceindices/timeseries/l55o/mm23.
5. HMRC. HMRC yearly average and spot rates: HM Revenue and Customs; [Available from: https://www.gov.uk/government/publications/exchange-rates-for-customs-and-vat-yearly.
6. HMRC. HMRC yearly average and spot rates: HM Revenue and Customs; 2022 [Available from: https://www.gov.uk/government/publications/exchange-rates-for-customs-and-vat-yearly.
7. Orphanet. The portal for rare diseases and orphan drugs 2022 [Available from: https://www.orpha.net/consor/cgi-bin/Disease_Search_Simple.php?lng=EN.
8. NICE. Policy on declaring and managing interests for NICE advisory committees, 2018.
9. WHO. ICD-11 for Mortality and Morbidity Statistics 2022 [Available from: https://icd.who.int/browse11/l-m/en#/http://id.who.int/icd/entity/465177735?view=G0.

For peer review only

For peer review only - http://bmjopen.bmj.com/site/about/guidelines.xhtml

CHEERS 2022 Checklist

Topic	No.	Item	Location where item is reported
**Title**			
	1	Identify the study as an economic evaluation and specify the interventions being compared.	p. 1, lines 1-3
**Abstract**			
	2	Provide a structured summary that highlights context, key methods, results, and alternative analyses.	p. 2, lines 4-33
**Introduction**			
**Background and objectives**	3	Give the context for the study, the study question, and its practical relevance for decision making in policy or practice.	p. 4, 5, 6 (all lines)
**Methods**			
**Health economic analysis plan**	4	Indicate whether a health economic analysis plan was developed and where available.	N/A
**Study population**	5	Describe characteristics of the study population (such as age range, demographics, socioeconomic, or clinical characteristics).	p. 7, lines 3-4
**Setting and location**	6	Provide relevant contextual information that may influence findings.	p. 7, line 4
**Comparators**	7	Describe the interventions or strategies being compared and why chosen.	N/A
**Perspective**	8	State the perspective(s) adopted by the study and why chosen.	p. 7, line 4
**Time horizon**	9	State the time horizon for the study and why appropriate.	p. 7, line 4
**Discount rate**	10	Report the discount rate(s) and reason chosen.	N/A
**Selection of outcomes**	11	Describe what outcomes were used as the measure(s) of benefit(s) and harm(s).	p. 7, 8, 9 (all lines)
**Measurement of outcomes**	12	Describe how outcomes used to capture benefit(s) and harm(s) were measured.	p. 7, 8, 9 (all lines)

For peer review only - http://bmjopen.bmj.com/site/about/guidelines.xhtml

Topic	No.	Item	Location where item is reported
**Valuation of outcomes**	13	Describe the population and methods used to measure and value outcomes.	p. 9, lines 12-15
**Measurement and valuation of resources and costs**	14	Describe how costs were valued.	N/A
**Currency, price date, and conversion**	15	Report the dates of the estimated resource quantities and unit costs, plus the currency and year of conversion.	p. 7, lines 30-34
**Rationale and description of model**	16	If modelling is used, describe in detail and why used. Report if the model is publicly available and where it can be accessed.	p. 8, lines 17-28
**Analytics and assumptions**	17	Describe any methods for analysing or statistically transforming data, any extrapolation methods, and approaches for validating any model used.	p. 9, lines 27-31
**Characterising heterogeneity**	18	Describe any methods used for estimating how the results of the study vary for subgroups.	N/A
**Characterising distributional effects**	19	Describe how impacts are distributed across different individuals or adjustments made to reflect priority populations.	N/A
**Characterising uncertainty**	20	Describe methods to characterise any sources of uncertainty in the analysis.	N/A
**Approach to engagement with patients and others affected by the study**	21	Describe any approaches to engage patients or service recipients, the general public, communities, or stakeholders (such as clinicians or payers) in the design of the study.	p. 9, lines 32-35
**Results**			
**Study parameters**	22	Report all analytic inputs (such as values, ranges, references) including uncertainty or distributional assumptions.	N/A
**Summary of main results**	23	Report the mean values for the main categories of costs and outcomes of interest and summarise them in the most appropriate overall measure.	p. 10, 11, 12, 13, 14 (all lines)
**Effect of uncertainty**	24	Describe how uncertainty about analytic judgments, inputs, or projections affect findings. Report the effect of choice of discount rate and time horizon, if applicable.	N/A

Topic	No.	Item	Location where item is reported
**Effect of engagement with patients and others affected by the study**	25	Report on any difference patient/service recipient, general public, community, or stakeholder involvement made to the approach or findings of the study	p. 9, lines 32-35
**Discussion**			
**Study findings, limitations, generalisability, and current knowledge**	26	Report key findings, limitations, ethical or equity considerations not captured, and how these could affect patients, policy, or practice.	p. 15-17 (all lines)
**Other relevant information**			
**Source of funding**	27	Describe how the study was funded and any role of the funder in the identification, design, conduct, and reporting of the analysis	p. 18, lines 11-15
**Conflicts of interest**	28	Report authors conflicts of interest according to journal or International Committee of Medical Journal Editors requirements.	p. 18, lines 16-20

From: Husereau D, Drummond M, Augustovski F, et al. Consolidated Health Economic Evaluation Reporting Standards 2022 (CHEERS 2022) Explanation and Elaboration: A Report of the ISPOR CHEERS II Good Practices Task Force. Value Health 2022;25. doi:10.1016/j.jval.2021.10.008

For peer review only - http://bmjopen.bmj.com/site/about/guidelines.xhtml

BMJ Open

Industry funding of patient organisations in the United Kingdom: A retrospective study of commercial determinants, funding concentration and disease prevalence

Journal:	*BMJ Open*
Manuscript ID	bmjopen-2022-071138.R3
Article Type:	Original research
Date Submitted by the Author:	01-Jun-2023
Complete List of Authors:	Gentilini, Arianna; The London School of Economics and Political Science, Department of Health Policy<br>Parvanova, Iva; The London School of Economics and Political Science, Department of Health Policy
**Primary Subject Heading**:	Health policy
Secondary Subject Heading:	Health policy
Keywords:	Health policy < HEALTH SERVICES ADMINISTRATION & MANAGEMENT, PUBLIC HEALTH, Health Equity

SCHOLARONE™
Manuscripts

I, the Submitting Author has the right to grant and does grant on behalf of all authors of the Work (as defined in the below author licence), an exclusive licence and/or a non-exclusive licence for contributions from authors who are: i) UK Crown employees; ii) where BMJ has agreed a CC-BY licence shall apply, and/or iii) in accordance with the terms applicable for US Federal Government officers or employees acting as part of their official duties; on a worldwide, perpetual, irrevocable, royalty-free basis to BMJ Publishing Group Ltd ("BMJ") its licensees and where the relevant Journal is co-owned by BMJ to the co-owners of the Journal, to publish the Work in this journal and any other BMJ products and to exploit all rights, as set out in our licence.

The Submitting Author accepts and understands that any supply made under these terms is made by BMJ to the Submitting Author unless you are acting as an employee on behalf of your employer or a postgraduate student of an affiliated institution which is paying any applicable article publishing charge ("APC") for Open Access articles. Where the Submitting Author wishes to make the Work available on an Open Access basis (and intends to pay the relevant APC), the terms of reuse of such Open Access shall be governed by a Creative Commons licence – details of these licences and which Creative Commons licence will apply to this Work are set out in our licence referred to above.

Other than as permitted in any relevant BMJ Author's Self Archiving Policies, I confirm this Work has not been accepted for publication elsewhere, is not being considered for publication elsewhere and does not duplicate material already published. I confirm all authors consent to publication of this Work and authorise the granting of this licence.

For peer review only

Industry funding of patient organisations in the United Kingdom: A retrospective study of commercial determinants, funding concentration and disease prevalence

Arianna Gentilini and Iva Parvanova

Department of Health Policy, London School of Economics and Political Science, London, UK

Corresponding author

Arianna Gentilini

Department of Health Policy
Cowdray House, COW.4.09
London School of Economics
Houghton street
London WC2A 2AE
England
Email: a.gentilini@lse.ac.uk

For peer review only

For peer review only - http://bmjopen.bmj.com/site/about/guidelines.xhtml

Abstract

Objectives – To assess the relationship between UK-based patient organisation funding and companies' commercial interests in rare and non-rare diseases in 2020.

Design – Retrospective analysis of the value and volume of payments from pharmaceutical companies to patient organisations in the UK matched with data on the conditions supported by patient organisations and drugs in companies' approved portfolios and research and development pipelines.

Setting – UK.

Participants – 74 pharmaceutical companies making payments to 341 UK-based patient organisations.

Main outcome measures – Alignment between the commercial interests of pharmaceutical companies and the disease area focus of patient organisations; difference in the volume and value of payments to patient organisations broken down by prevalence of conditions; industry funding concentration, measured as the number of companies funding each patient organisations, the share of overall industry funding coming from each contributing company and the share of industry funding of each organisation comprised by the single highest payments.

Results – 1,422 payments were made by 74 companies to 341 patient organisations. Almost all funds (90%) from pharmaceutical companies were directed to patient organisations that are aligned with companies' approved drug portfolios and research and development pipelines. Despite rare diseases affecting less than 5% of the UK population, more than 20% of all payments were directed to patient organisations which target such conditions. Patient organisations focusing on rare diseases relied on payments from fewer companies (*p-value* = 0.0031) compared to organisations focusing on non-rare diseases.

Conclusions – Companies predominantly funded patient organisations operating in therapeutic areas relevant to companies' portfolio or drug development pipeline. Patient organisations focusing on rare diseases received more funding relative to the number of patients affected by these conditions and relied more heavily on payments from fewer companies compared to organisations targeting non-rare diseases. Increased independence of patient organisations could help avoiding conflicts of interest.

For peer review only - http://bmjopen.bmj.com/site/about/guidelines.xhtml

Strengths and limitations of this study

- We develop a methodology to determine the concordance between commercial interests of pharmaceutical companies and disease areas supported by patient organisations.
- We present a comparative analysis of industry funding to patient organisations depending on the prevalence of the disease(s) they support.
- Our analysis focuses on a recent time period which might differ from historical trends.

For peer review only

Introduction

Patient organisations – not-for-profit organisations mainly composed of patients and/or caregivers that represent and support the needs of patients or caregivers [1 2] – play an important role in the development, regulatory review, and adoption of new drugs.

During research and development, patient organisations effectively advocate for resources to be directed to conditions where unmet need is highest.[3 4] Patient organisations support research design and planning, helping to identify patient-relevant study endpoints.[4] Patient organisations also represent patient views and preferences at the time of regulatory review and health technology assessment of new drugs.[5 6] For example, during technology appraisals conducted by the National Institute for Health and Care Excellence (NICE), which makes funding recommendations for the English National Health Service (NHS), patients, and organisations representing the interests of patients, provide testimonies of their first-hand experiences on how the disease affects them and those around them.[7] Finally, when drugs are launched, patient organisations contribute to dissemination of research results to patient community and clinicians, and offer support and information on therapies available.[4 8]

Given the increasingly important role of patient organisations it is vital to understand their financial ties with pharmaceutical companies. Previous studies documented the large number and high value of payments from pharmaceutical companies to patient organisations, [2 8-10] the uneven distribution between and within therapeutic areas,[2 10] and the concentration of payments coming from a small number of pharmaceutical firms across multiple jurisdictions.[2 8-16]

What remains unknown is the alignment between the commercial interests of pharmaceutical companies and UK patient organisations' activities. Prior research has demonstrated that industry tends to prioritize commercially attractive conditions, and there is evidence to suggest that the marketing of a drug for a particular disease is associated with increased industry funding to patient organisations operating in that area. [2 10] However, such studies have typically been conducted in different geographic settings and have focused solely on marketed drugs, rather than examining the entire research and development pipeline of pharmaceutical companies. This is especially important given the lengthy timeline for drugs to reach the market,[17] as failure to consider drugs currently undergoing clinical trials may result in an incomplete picture.

Another gap in the literature relates to the dynamics between the pharmaceutical industry and patient organisations supporting rare vs. non-rare conditions. In the UK, diseases are defined rare if they affect up to 5 people in 10,000.[18 19] The low prevalence of rare diseases and their different aetiology, coupled with the lack of interest from policymakers and manufacturers, who often prioritise more profitable and prevalent diseases, has necessitated the formation of patient organisations to advocate for the needs of rare disease patients.[20 21] The National Organisation for Rare Disorders (NORD), serves as the umbrella organisation for rare disease patients in the United States (US) and has been instrumental in lobbying for scientific support and economic incentives to stimulate innovation in rare diseases.[22] This advocacy ultimately led to the passing of the Orphan Drug Act in 1983 in the USA and the EU Regulation on Orphan Medicinal Products in Europe in 2000.[18 23]

For peer review only - http://bmjopen.bmj.com/site/about/guidelines.xhtml

Moreover, the limited availability and complexity of medical knowledge regarding rare diseases have also fostered patients and families affected by these conditions to come together to provide each other with support and medical expertise.[20 24] Patient organisations, which are primarily composed of patients and their caregivers, are in a unique position to share first-hand experiences that can inform research and regulatory decisions.[25] While this is true also for non-rare conditions, patient organisations' input in regulatory and health technology appraisals is particularly important in the context of rare diseases due to scarce evidence. For example, the Scottish Medicines Consortium (SMC) provides opportunities for patient groups and clinicians to have a stronger voice in the decision-making process for drugs used to treat rare and end-of-life conditions.[26] Similarly, three members of patient organisations sit in the Committee for Orphan Medicinal Products (COMP) within the European Medicines Agency (EMA), the body responsible for granting orphan designations to drugs. Patient organisation-led registries that collect real-world data on disease progression can de-risk drug development for rare diseases.[20] While observational studies are common in non-rare diseases, they usually do not require the support of patient organisations' networks as patients are easier to identify and recruit.[3]

Finally, there has been limited exploration of the concentration of industry funding for patient organisations. A recent study by Mulinari and colleagues (2022) examined the average number of pharmaceutical companies making payments to Danish patient organisations,[15] while only one study has investigated the share of industry funding and the top drug company donor's share in UK patient organisations' income.[11] However, no study has specifically focused on the number of companies funding UK patient organisations, nor have they explored whether organisations' industry funding differs based on disease rarity.

Our paper aims to contribute to and expand on existing literature by examining the concordance between the commercial interests of pharmaceutical companies and patient organisations' activities in the UK. Using publicly available data on 2020 payments, we analysed the volume, value of payments to patient organisations according to their disease area of interest, with the objective of examining whether there are differences in funding patterns between rare and non-rare diseases. Lastly, we examined the concentration of industry funding, namely how many companies funded each patient organisation and the extent to which organisations might have been reliant on funding from a single company. Based on the reviewed literature, we formulated the following hypotheses:

- *Hypothesis 1*: Regarding the concordance between the commercial interests of pharmaceutical companies and patient organisations' activities, we expect no difference between rare and non-rare patient organisations, under the assumption that companies are unlikely to fund organisations out of altruistic motives;
- *Hypothesis 2*: Furthermore, we hypothesise that patient organisations targeting rare diseases would receive less overall funding due to their low prevalence. However, the existing incentives, high costs and consequent profitability of some orphan-designated drugs might affect the proportion of funding directed towards these organisations.[27 28]
- *Hypothesis 3*: Considering the limited availability of drugs for rare diseases from a handful of manufacturers, we expect organisations focusing on these conditions to rely

For peer review only - http://bmjopen.bmj.com/site/about/guidelines.xhtml

on payments of higher value and from fewer companies compared to those targeting more prevalent conditions.

For peer review only

For peer review only - http://bmjopen.bmj.com/site/about/guidelines.xhtml

Methods

Data on industry payments

Disclosure reports on pharmaceutical companies' websites were our primary data source on payments from the pharmaceutical industry to UK patient organisations in 2020.[29] Disclosing payments to patient organisations is a requirement of Clause 29 of the Association of British Pharmaceutical Industry (ABPI) Code of Practice.[30] Specifically, the ABPI requires companies to keep a public record of any payment made to patient organisations on their website for a minimum of three years following the payment.[30] Companies that sign up to abide by the ABPI Code accept the jurisdiction of the Prescription Medicines Code of Practice Authority (PMCPA, code regulator), which also affects non-ABPI members operating in the UK.[30] Companies may be sanctioned by the PMCPA if they do not disclose their payments.[30] In an effort to increase transparency, Disclosure UK, an industry-led platform showing payments from pharmaceutical companies to healthcare professionals and organisations, launched a gateway in 2020 that collects hyperlinks to companies' disclosures of payments to patient organisations.[31]

First, we screened the websites of all pharmaceutical companies abiding by the ABPI Code, aided by the Disclosure UK patient organisations gateway. We retrieved payments information from the companies' websites to ensure that all payments were captured. Second, in light of a recent study unveiling that payments to patient organisations were misreported in the Disclosure UK database of payments to healthcare organisations (HCOs),[16] we screened the 2020 Disclosure UK HCOs database for payments to patient organisations.

If payments were not disclosed in the company's website nor in the Disclosure UK HCOs database, we assumed that the company did not make any payments to patient organisations in 2020, as commonly assumed in the literature.[2]

One investigator (AG) extracted payment disclosures from the companies' websites. These comprised the name of the patient organisation, the year when the payment was made, the reason for the payment and its value in the currency reported by the disclosing company. The 2020 Disclosure UK HCOs database was also screened, and recipients were matched to standardised patient organisations names. To ensure the data's accuracy, the final database was scanned for duplicates, but no such instances were found. When reported in different currencies, such as United States Dollars (USD), Swiss Franc (CHF), Swedish Krona (SEK), Norwegian Krone (NKK) and Danish Krone (DKK), the value of the payment was converted to Great British Pounds (GBP), using the ONS historical yearly conversion rates. [32 33] All payments are reported in 2020 GBP. Two in-kind payments with a monetary value of zero were excluded from the analysis. Further details on variables' cleaning and coding can be found in the Supplemental Material.

Data on patient organisations

We retrieved data on patient organisations from their websites. Details on the therapeutic area they advocated for – proxied by International Classification of Diseases Version 11 (ICD-11)

For peer review only - http://bmjopen.bmj.com/site/about/guidelines.xhtml

codes – and whether the condition(s) was rare or non-rare were also extracted. Conditions were considered rare if they appeared in the Orphanet database of rare diseases, [34] which is platform and repository of data on rare diseases and orphan drugs. Patient organisations that did not match the European Federation of Pharmaceutical Industries and Associations (EFPIA) definition of what constitutes a patient organisation were excluded from the analysis. We chose the EFPIA's definition for the following reasons. First, this corresponds the definition used in the wider peer-reviewed literature.[2 35] Second, other commonly used definitions, such as the one from the EMA, refer to the structure of patient organisations' governing bodies, which have to consist of over 50% patients.[36] Considering the high number of patient organisations included in our analysis, this requirement was challenging – if not impossible – to verify. Second, EFPIA's definition indicates what the pharmaceutical industry considers to be a patient organisation. Therefore, it helped us minimize selection bias issues as it includes a wide range of organisations. We excluded 66 payments to patient organisations that did not match EFPIA's definition. Sub-group analyses on excluded organisations can be found in the Supplemental Material.

Determining commercial interests

We assessed whether – and the extent to which – a pharmaceutical company holds an interest in the disease supported by a patient organisation. We adapted the definition of 'interest' provided by NICE [37]. An interest is when there is, or could be perceived to be, an opportunity for a pharmaceutical company to benefit in the disease area where the patient organisation operates. This could include cases where the pharmaceutical company has a drug developed or in development for a condition targeted by the patient organisation, or where a drug in the company's portfolio or pipeline is restricted to a specific population affected by the disease supported by the patient organisation. We define portfolio as a group of drugs that a pharmaceutical company has already developed, gained regulatory approval for, and is actively marketing or selling. Conversely, pipeline refers to the collection of drug candidates being developed by a pharmaceutical company, at various stages of development, from preclinical research to clinical trials.

To establish whether an interest existed or not, we first classified the conditions targeted by patient organisations to ICD-11 codes using the online ICD-11 database.[38] ICD-11 codes are mutually exclusive, exhaustive and are arranged as a single hierarchical tree, from level one (most general e.g., *neoplasms*) to five (most specific, e.g. *plasma cell myeloma*). This means that specific diseases are nested within broader classifications. Although some patient organisations, such as hospital charities, carers organisations, and hospices, could not be matched to specific ICD-11 codes, they were included in the analysis to provide a comprehensive overview. As a result, the analysis presented results for both disease-specific and non-disease-specific organisations.

We then searched companies' annual reports, websites and the ClinicalTrials.gov registry to determine whether each company had an interest in the condition targeted by the patient organisation receiving the payment. Figure 1 schematically illustrates the approach taken to understand whether – and the degree to which – a company has an interest in the conditions

For peer review only - http://bmjopen.bmj.com/site/about/guidelines.xhtml

(*definitely yes, probably yes, no*). For example, if *Company X* declares in its annual report having a drug in development for multiple myeloma and made a payment to *Blood Cancer UK*, this would be coded as *probably yes,* as the company has a product in its pipeline or portfolio nested within a broader class of conditions targeted by the patient organisation. Conversely, should *Company X* have made a payment to *Myeloma UK,* this would have been coded as *definitely yes,* as there is perfect alignment between the condition targeted by the patient organisation and by *Company X's* drug. Cases in which a company's interest in a certain condition could not be identified were coded as *no.* However, these instances might be due to limitations in data availability and therefore do not necessarily indicate that there was no company interest. Data on pharmaceutical companies' portfolio and pipeline were retrieved from their latest annual reports, company websites and ClinicalTrials.gov.[39]

One investigator (AG) initially coded all data, while the other (IP) blindly re-coded a 30% random sample of payments to validate the data collection process and minimise the risk of reporting errors. We followed this process when validating all data sources described above. Any disagreement was discussed until consensus was reached.

Analysis of industry funding concentration

We assessed the concentration of industry funding received by patient organisations. In a prior study, Ozieranski and colleagues examined funding disparities among healthcare organisations in the UK in 2015, using the Gini coefficient to assess the distribution of funding.[40] However, the authors acknowledged that the data preparation process presented challenges, limiting the analysis to payments from a single year. While this methodology has its advantages, we found that the time-consuming process of reshaping the data outweighed the benefits over using descriptive statistics. In particular, we calculated (1) the number of companies funding each patient organisations, (2) the share of overall industry funding to each patient organisations coming from each contributing company and (3) the share of industry funding of each organisation comprised by the single highest payment.

The Supplemental Material provides further details on the data collection and how the outcomes were constructed. Descriptive statistics and tests, such as ranges and Mann–Whitney U tests, were presented in the analysis. These statistics were preferred over the mean due to the skewed distribution of the data analysed. All analyses and data visualisations were performed using Stata 17 and RStudio (*ggplot2* package), respectively.

Patient and public involvement

Patients were not involved in this study as our analyses focused on patient organisations as institutional actors rather than single patients with specific conditions. We plan to disseminate key findings from our analysis to patients and members of the public.

For peer review only - http://bmjopen.bmj.com/site/about/guidelines.xhtml

Results

In 2020, 74 companies made 1,422 payments to 341 patient organisations, amounting to £22.6 million. Out of the total of 1,422 payments made by pharmaceutical companies to patient organisations in 2020, 82% (1,168 payments) with a value of £18 million were accurately disclosed on the companies' websites. The remaining 18% (254 payments) with a value of £4.6 million were reported in the Disclosure UK HCOs database. Among the companies, 24 out of 74 reported payments only on their websites, while 14 reported payments only in the Disclosure UK HCOs database, and 36 reported payments in both.

Overall, *diseases of the nervous system* (£4.3 million) was the most funded therapeutic area over time, followed by *neoplasms* (£3.2 million) and *endocrine, nutritional or metabolic diseases* (£3.4 million). The conditions that received more funding in 2020 were multiple sclerosis (£1.7 million), followed by obesity (£1.4 million) and epilepsy (£1 million). Pfizer, Novo Nordisk, UCB, Novartis and Roche were the top five funders over the study period (Figure 2). These companies contributed to more than a third (36%) of all payments.

Table 1 summarises the number and value of payments to patient organisations.

Companies' interest in payments to patient organisations

In 2020, 85% of all payments were directed to patient organisations that were judged to be aligned with their portfolio or pipeline. Only 15% of payments were made to organisations that focused on conditions that could not be linked to a product in the funder's portfolio or pipeline. Table 2 shows the volume and value of payments, broken down by the company's interest variable, overall and whether patient organisations targeted a rare or non-rare disease. Payments to patient organisations targeting a disease for which the company has a product developed or in development (*definitely yes*) made up 56% and 54% for patient organisations targeting rare and non-rare conditions, respectively. However, this difference was not statistically significant as anticipated in *Hypothesis 1* ($\chi^2 =$ 1.049, *p-value* = 0.592).

The monetary value of payments coded as *definitely yes* accounted for 55% of the overall payment value. However, this was as high as 67% for patient organisations targeting rare diseases, versus 59% for organisations focusing on non-rare conditions. This difference was found to be statistically significant ($\chi^2 =$ 370.163, *p-value* = 0. 058). When payments coded as *probably yes* were included, the share increased to 90% and 97% for all patient organisations and disease-specific organisations only, respectively.

For peer review only - http://bmjopen.bmj.com/site/about/guidelines.xhtml

Table 1. Number and value of payments from the pharmaceutical industry to UK patient organisations broken down by year and rarity of diseases

**Payment statistics**	
**Number of payments**	1,422
**Median payment (IQR; overall)**	£7,943 (£1,200 - £15,000)
**Median payment (IQR; rare)**	£8,775 (£2,500 - £15,965)
**Median payment (IQR; non-rare)**	£9,060 (£1,520 - £16,850)
**Value of payments (£; overall)**	£22,577,314
**Value of payments (£; rare)**	£4,629,779
**Value of payments (£; non-rare)**	£15,875,662
**Number of pharmaceutical companies**	74
**Number of patient organisations**	341

Abbreviations: IQR (Interquartile range).
Notes: All payments are expressed in 2020 GBP. The Supplemental Materials detail the conversion rates used, which were retrieved from the Office of National Statistics (ONS) website. Further details on how patient organisation data were cleaned and coded, please see the Supplemental Materials . Please note that the number of pharmaceutical companies and patient organisations making and receiving payments across the study period refers to companies and organisations that made or received at least one payment, respectively.

Table 2. Volume and value of payments by company interests in 2020

**PO type**	**Company's interest**	**Volume**; n (%)	**Value**: £
Overall†	Definitely yes	678 (48%)	£12,529,514 (56%)
	Probably yes	525 (37%)	£7,700,069 (34%)
	No*	219 (15%)	£2,347,732 (10%)
Rare	Definitely yes	161 (56%)	£3,119,217 (67%)
	Probably yes	115 (40%)	£1,388,545 (30%)
	No*	10 (4%)	£122,017 (3%)
Non-rare	Definitely yes	517 (54%)	£9,410,297 (59%)
	Probably yes	389 (41%)	£6,056,915 (38%)
	No*	46 (5%)	£408,449 (3%)

For peer review only - http://bmjopen.bmj.com/site/about/guidelines.xhtml

Notes: *Definitely yes* indicates payments directed to patient organisations that operated in a disease area (ICD-11 level 4 or higher) for which the company has a product in its portfolio or pipeline. *Probably yes* indicates directed to patient organisations that operated in a disease area (ICD-11 level 3 or lower) for which the company has a product in its portfolio or pipeline. *No* refers to directed to patient organisations that operated in a disease area for which no link could be found to the company's portfolio or pipeline. The higher the ICD-11, the more specific the condition. For example, if the ICD-11 level 4 is *Plasma cell neoplasms,* level 2 would be *Neoplasms of hematopoietic or lymphoid tissues.* Further details on how this variable was constructed can be found in the Supplemental Material.

*Please note that the *No* category of interest conservatively includes also interests that were considered as unclear.

†Please note that the *Overall* results are not a sum of the *Rare* and *Non-rare* results, as they also include patient organisations that could not be classified in either group and are non-disease-specific.

For peer review only

For peer review only - http://bmjopen.bmj.com/site/about/guidelines.xhtml

Industry funding of patient organisations focusing on rare vs. non-rare conditions

Of the £22.6 million in payments from industry to patient organisations, £4.6 million (21%; n=286) were directed to organisations focusing on rare diseases while £15.9 million (70%; n=952) to organisations supporting non-rare conditions. The remaining 9% was directed to non-disease-specific patient organisations, which were excluded from this analysis. Linking these results to *Hypothesis 2*, we observe that patient organisations supporting rare diseases received less but still substantial funding.

The most funded patient organisation overall in 2020 was the European Association for the Study of Obesity, receiving almost £1.5 million, followed by Epilepsy Society (£955,600) and Shift.MS (£588,451). Among the top ten recipients overall in 2020, only one focused on rare diseases (Cystic Fibrosis Trust). However, it is worth noting that Blood Cancer UK, which focuses on malignant haematological malignancies including rare cancers, ranked seventh on the list.[41] The Cystic Fibrosis Trust (£445,229), The Society for Mucopolysaccharide Diseases (£358,037), and the International Patient Organisation for Primary Immunodeficiencies (£345,914) were the top three recipients focusing on rare diseases, followed by Myeloma UK with a slightly lower amount (£340,604).

Figure 3 shows therapeutic areas in order from most to least funded, broken down by rarity of disease targeted. In the case of organisations focusing on rare diseases, *endocrine, nutritional or metabolic disease*, *neoplasms* and *diseases of the nervous system* received most funds. Together, the top three most funded disease areas represented about half of overall funding (57%). When looking at the non-rare conditions that attracted most funding, multiple sclerosis was first (£1.7 million), followed by diabetes (£1.4 million) and epilepsy (£1 million). Cystic fibrosis, primary immunodeficiencies, and lysosomal storage diseases, which include rare metabolic disorders such as Fabry and Gaucher diseases, received the highest funding overall, attracting £445,229, £363,998 and £358,037, respectively.

For peer review only

Industry funding concentration

Each patient organisation received payments from a median of approximately one unique company, with 1 (IRQ:1-2) and 2 (IQR:1-3) companies funding patient organisations targeting rare and non-rare diseases, respectively. However, this difference was not statistically significant (z = 1.582, *p-value* = 0.114). Overall, the range of unique companies making payments to a unique patient organisation spanned from a minimum of 1 to a maximum of 13. The latter was recorded for Genetic Alliance UK, a national charity and an alliance of over 200 patient organisations, supporting those affected by rare genetic conditions.

In our sample, the median yearly payment of a company to a patient organisation comprised 24% of the its overall industry payments (IQR: 9.5%-74%). When looking at patient organisations focusing on rare diseases, the median company contribution was as high as 30% (IQR: 11.6%-93%) versus 23% (IQR: 9.4%-65.8%) for non-rare conditions (z = -2.164, *p-value* = 0.031).

Finally, the share of industry funding comprised by the single highest payment per organisation amounted to an average of 67.5% (SD: 0.30) for all years, ranging from a minimum of 8.5% to a maximum of 100%. The highest value payment in the case of patient organisations targeting rare diseases made up a larger share of the overall industry funding (median: 71%, IQR: 43.5%-100%), despite not significant, compared to those focusing on more prevalent conditions (median: 62.5%, IQR: 34.7%-100%). While there was not a significant difference in the number of funding companies between patient organisations supporting rare and non-rare diseases (z = -1.087, *p-value* = 0.277) as stated in *Hypothesis 3*, the former relied on larger payments. Histograms illustrating the distribution of the statistics explored in this analysis can be found in the Supplemental Materials.

For peer review only - http://bmjopen.bmj.com/site/about/guidelines.xhtml

Discussion

In this study, we evaluated the financial links between the pharmaceutical industry and patient organisations in the UK in 2020. This is the first study to document the almost-perfect concordance of pharmaceutical company interests and patient organisation funding in the UK. Almost all industry payments during our study period – in terms of both volume (85%) and value (90%) – were to patient organisations aligned with pharmaceutical companies' portfolios and pipelines. This share was even higher when considering only disease-specific patient organisations (97%). Despite rare diseases affecting less than 5% of the UK population, more than 20% of industry funding to patient organisations in 2020 was directed towards organisations focusing on such conditions (£4.6 million / £22.6 million). Finally, we found that patient organisations targeting rare diseases relied on payments from fewer companies but of higher value compared to organisations focusing on non-rare diseases.

The almost-perfect concordance between industry interests and patient organisation activities likely reflect the commercial attractiveness of conditions targeted by pharmaceutical companies.[2 42] Such close alignment between the interests of companies and patient organisations might undermine the credibility of patient organisations as perceived by the general public and might raise questions about patient organisations' inputs in regulatory and health technology appraisals.[9 43 44] Similarly, a study found that during NICE appraisal meetings fewer than 25% of all relevant financial ties between patient organisations and pharmaceutical companies were disclosed.[45] As discussed by the Mandeville and colleagues, this lack of transparency increases the risk of conflicts of interest not being properly detected and managed.

Our findings make an important contribution to the existing body of literature on industry funding of patient organisations. Ozieranski et al found that industry donated over £57 million to UK patient organisations from 2012 to 2016, an average of £11.5 million per year.[2] The authors also observed that payments were concentrated in commercially attractive therapeutic areas, with organisations focusing on cancer receiving more than 36% of overall payments.[2] However, the study did not examine whether companies were more likely to fund organisations that target diseases for which they have already developed or are currently developing products. Another earlier study examined payments to Swedish patient organisations and found an association between drug commercialisation and industry funding.[10] The authors did not take into account products in the companies' pipelines nor drugs that might had not yet launched in Sweden. Considering that patient organisations have an important role not only in the post-commercialisation phase but also in the R&D and approval stages. We therefore developed a replicable classification model to determine whether payments from companies were directed at organisations that were aligned with their portfolios and pipelines.

Patient organisations focusing on rare diseases can drive both supply of and demand for medicinal products due to their research, advocacy and education role. [4 46] As a result of their close ties with patients, these organisations have the credibility and power to educate patient communities, advocate for access to available therapies and raise awareness on the unmet need of certain conditions.[4 20 47] Although a large share of both the value and number of payments were directed to patient organisations focusing on rare diseases, most funds targeted

For peer review only - http://bmjopen.bmj.com/site/about/guidelines.xhtml

commercially attractive rare conditions, such as multiple myeloma and cystic fibrosis, where the unmet need is relatively low compared to other rare conditions. These are diseases that have relatively high prevalence and for which 10 and 29 treatments, respectively, are currently approved for use in Europe.[34 48] Furthermore, rare diseases have proved a lucrative asset for pharmaceutical companies.[42] The additional market protection granted to orphan-designated product and the often higher willingness to pay from payers has led companies to increasingly focus on these medicines, which can offer a high return on investment.[27 28] This poses the risk of widening already existing health inequities, where severe and debilitating rare conditions that affect a small number of patients do not receive the resources they need and have to rely on limited public grants.[49]

Finally, our analysis showed that patient organisations focusing on rare diseases are funded by very few companies, relying on a single payment for over 70% of their industry-reported income. Despite the share of industry contributions among the overall patient organisation's income was found to be low in the literature,[11] this increases the risk of pursuing the company's commercial interests rather than objectively representing a patient body.[12] In this study we find that patient organisation received payments from a median of approximately one unique company (IRQ:1-3), ranging from 1 to a maximum of 13. This corresponds to an average of 2.6 (SD:2.3) funding companies per patient organisation. This is consistent with findings from a recent study investigating the distribution of payments from industry to Danish patient organisations, which found that on average, most organisations were funded by 2.6 (SD:2.1) on average.[15]

These findings have important implications for policy and practice. To minimise conflicts of interests and maintain the integrity of patient organisations, particular attention should be paid to funding from companies in the period before or after a patient organisation has endorsed this company's product.[45] However, the duration of this period should be carefully evaluated to avoid overlooking more historical commercial ties.[50] One way of avoiding potential conflicts of interest is through increased transparency. Despite considerable progress on this front, especially in terms of reporting the monetary value of industry payments, there are still gaps in reporting.[51]

As highlighted in this and other studies, several companies misreport their payments to patient organisations.[16] Our study found that only 32% of companies disclose all of their payments correctly (i.e., on their website), while the rest report them on both their websites and the Disclosure UK HCOs database (49%) or solely on the latter (19%). This duplication of reporting efforts makes it harder to achieve transparency and obtain a comprehensive overview of the financial relationships between companies and patient organisations. Therefore, efforts should be made to establish a unique repository for payments to patient organisations, similar to the one currently in place for physicians and healthcare organisations.

Furthermore, the financial independence of patient organisations is fundamental to ensure that patients' interests are at the forefront of the organisations' agenda.[52] Compromising this independence can have a detrimental effect and distort public health priorities. For example, AbbVie-sponsored patient organisations were found to strongly oppose switching to biosimilars for Humira, the company's blockbuster drug, in various countries.[15] Similarly, a

For peer review only - http://bmjopen.bmj.com/site/about/guidelines.xhtml

recent investigation uncovered strong financial connections between Novo Nordisk and UK-based patient organisations that supported the approval of the company's latest obesity drug. This, alongside other ongoing investigations, culminated in the suspension of the company from ABPI.[53] The strong financial ties between Novo Nordisk and patient organisations, contributing to the NICE appraisal of the company's drug, raises serious concerns about these groups' independence and might ultimately harm patients.[50] Notably, our analysis found Novo Nordisk to be the second highest funder of patient organisations in term of value in 2020 for an amount of more than £1.8 million. In the long term, policymakers should make sure that patient organisations receive adequate public funding regardless of whether they focus on conditions that are profitable for the industry. Such public funding is particularly important for patient organisations supporting rare diseases, as relatively few companies have financial links with patient organisations focusing on rare diseases, potentially creating high reliance on few high-value payments.

This study had limitations. First, the lack of mandatory reporting of payments to patient organisations by companies that do not comply with the ABPI Code is a major limitation of our analysis.[54] For example, our dataset does not include payments by Vertex, a company with a rare-focused portfolio and a strong presence in cystic fibrosis.[55] Even for companies that are signatories of the ABPI Code, underreporting of payments to patient organisations and removal of disclosure reports from the public domain has been observed.[13 56 57] Second, in our assessment of company interests, we made a conservative assumption that only patient organisations which target relatively narrow conditions were eligible to be coded as *definitely yes*. Despite this assumption, we concluded that more than half of payments were in therapeutic areas in which companies had a clear interest. Finally, our analysis focused on a recent though limited time period. While previous publications show similar trends in terms of the most funded diseases and absolute value of payments,[2 10] lending credibility to our analysis and underlying data, it is still unclear whether these trends hold over time and their generalisability to other periods.

There are several avenues which can be explored further to build on this analysis. While some of the previous literature on the topic has focused on the financial dependency of patient organisations' budgets from pharmaceutical funding,[11] whether this differs depending on the rarity of the disease targeted has not been explored. Due to the small number of patients affected by rare conditions, patient organisations that target such conditions may be less well-equipped to finance their activities via charitable events and may rely more heavily on contributions from pharmaceutical companies. Lastly, while our analysis did not evaluate the effect of Covid-19 on the financial dynamics between pharmaceutical companies and patient organisations, we expect that the pandemic had a substantial effect on the type, value and distribution of payments. Future research should examine the impact of Covid-19 on industry funding of patient organisations.

Conclusions

Almost all industry funding of UK patient organisations in 2020 was in areas that were aligned with companies' approved drug portfolios and research and development pipelines.

For peer review only - http://bmjopen.bmj.com/site/about/guidelines.xhtml

Pharmaceutical companies spent a larger amount on patient organisations focusing on rare diseases and these organisations relied on a small of companies for their funding.

For peer review only

For peer review only - http://bmjopen.bmj.com/site/about/guidelines.xhtml

Acknowledgements: We thank Dr Huseyin Naci and Dr Olivier Wouters, both from the Department of Health Policy at the London School of Economics and Political Science, for providing comments on earlier drafts of this paper. We also acknowledge Dr Mylene Lagarde and Dr Panos Kanavos for the support provided when finalising the manuscript.

Contributors: AG developed a preliminary version of the study and developed it further with IP. AG collected the data. AG and IP did the analysis, wrote and reviewed the manuscript. Both authors had full access to all of the data (including statistical reports and tables) in the study and can take responsibility for the integrity of the data and the accuracy of the data analysis. The corresponding author attests that all listed authors meet authorship criteria and that no others meeting the criteria have been omitted. AG is the guarantor.

Funding: This research received no specific grant from any funding agency in the public, commercial or not-for-profit sectors. AG is funded by the ESRC for her doctoral research (Project reference: 2480077) and is employed by Imperial College London. IP is funded by the ESRC for her doctoral research (Project reference: 2311285) and is employed by Imperial College London.

Competing interest statement: No competing interest.

Ethical approval: This study does not involve human participants and ethical approval was not required.

Data sharing: Extra data can be accessed via the Dryad data repository at http://datadryad.org/ with the doi: 10.5061/dryad.fqz612jxd

Transparency declaration: The lead author affirms that the manuscript is an honest, accurate, and transparent account of the study being reported; that no important aspects of the study have been omitted; and that any discrepancies from the study as planned (and, if relevant, registered) have been explained.

The Corresponding Author has the right to grant on behalf of all authors and does grant on behalf of all authors, an exclusive licence (or non-exclusive for government employees) on a worldwide basis to the BMJ Publishing Group Ltd to permit this article (if accepted) to be published in BMJ editions and any other BMJPGL products and sublicences such use and exploit all subsidiary rights, as set out in our licence.

For peer review only

References

1. EFPIA. EFPIA Code of Practice on the Relationships between the Pharmaceutical Industry and Patient Organisations: European Federation of Pharmaceutical Industries and Associations, 2011.
2. Ozieranski P, Rickard E, Mulinari, Shai. Exposing drug industry funding of UK patient organisations. *BMJ* 2019;365:l1806. doi: 10.1136/bmj.l1806
3. Polich GR. Rare disease patient groups as clinical researchers. *Drug Discovery Today* 2012;17(3):167-72. doi: https://doi.org/10.1016/j.drudis.2011.09.020
4. Geissler J, Ryll B, di Priolo SL, et al. Improving Patient Involvement in Medicines Research and Development::A Practical Roadmap. *Therapeutic Innovation & Regulatory Science* 2017;51(5):612-19. doi: 10.1177/2168479017706405
5. MHRA. Patient Involvement Strategy 2021-25: Medicines and Healthcare products Regulatory Agency 2020.
6. MHRA. Putting patients first: A new era for our agency. Delivery Plan 2021-2023: Medicines and Healthcare products Regulatory Agency 2020.
7. NICE. Public Involvement Programme - Overview of technology appraisals: A factsheet for patient and carer organisations: National Institute for Health and Care Excellence, 2014
8. Fabbri A, Parker L, Colombo C, et al. Industry funding of patient and health consumer organisations: systematic review with meta-analysis. *BMJ* 2020;368:l6925. doi: 10.1136/bmj.l6925
9. Rose SL, Highland J, Karafa MT, et al. Patient Advocacy Organizations, Industry Funding, and Conflicts of Interest. *JAMA Intern Med* 2017;177(3):344-50. doi: 10.1001/jamainternmed.2016.8443
10. Mulinari S, Vilhelmsson A, Rickard E, et al. Five years of pharmaceutical industry funding of patient organisations in Sweden: Cross-sectional study of companies, patient organisations and drugs. *PLoS One* 2020;15(6):e0235021. doi: 10.1371/journal.pone.0235021 [published Online First: 20200624]
11. Ozieranski P, Pitter JG, Rickard E, et al. A 'patient-industry complex'? Investigating the financial dependency of UK patient organisations on drug company funding. *Sociol Health Illn* 2022;44(1):188-210. doi: 10.1111/1467-9566.13409 [published Online First: 20211207]
12. Rose SL. Patient advocacy organizations: institutional conflicts of interest, trust, and trustworthiness. 2014(1748-720X (Electronic))
13. Lexchin J, Batt S, Goldberg D, et al. National patient groups in Canada and their disclosure of relationships with pharmaceutical companies: a cross-sectional study. *BMJ Open* 2022;12(3):e055287. doi: 10.1136/bmjopen-2021-055287
14. Parker L, Fabbri A, Grundy Q, et al. "Asset exchange"—interactions between patient groups and pharmaceutical industry: Australian qualitative study. *BMJ* 2019;367:l6694. doi: 10.1136/bmj.l6694
15. Mulinari S, Pashley D, Ozieranski P. Advancing international comparison of pharmaceutical industry funding of patient advocacy: Focus on Denmark. *Health Policy* 2022;126(12):1256-62. doi: https://doi.org/10.1016/j.healthpol.2022.11.003
16. Rickard E, Carmel E, Ozieranski P. Comparing pharmaceutical company payments in the four UK countries: a cross-sectional and social network analysis. *BMJ Open* 2023;13(3):e061591. doi: 10.1136/bmjopen-2022-061591
17. Wouters OJ, McKee M, Luyten J. Estimated Research and Development Investment Needed to Bring a New Medicine to Market, 2009-2018. 2020(1538-3598 (Electronic))
18. European Commission. Regulation (EC) No 141/2000 of the European Parliament and of the Council of 16 December 1999 on Orphan Medicinal Products, 2000.
19. Department of Health & Social Care. Policy Paper - The UK Rare Diseases Framework 2021 [Available from: https://www.gov.uk/government/publications/uk-rare-diseases-framework/the-uk-rare-diseases-framework.
20. Aymé S, Kole A, Groft S. Empowerment of patients: lessons from the rare diseases community. *Lancet* 2008;371(9629):2048-51. doi: 10.1016/s0140-6736(08)60875-2

For peer review only - http://bmjopen.bmj.com/site/about/guidelines.xhtml

21. Gamba S, Magazzini L, Pertile P. R&D and market size: Who benefits from orphan drug legislation? *Journal of Health Economics* 2021;80:102522. doi: https://doi.org/10.1016/j.jhealeco.2021.102522
22. Waxman HA. The Waxman Report : How Congress Really Works: First edition. New York : Twelve, 2009. 2009.
23. Office of the Federal Register NAaRA. Orphan Drug Act - 6 Stat. 2049. In: Office. USGP, ed., 1983.
24. Halley MC. From "Ought" to "Is": Surfacing Values in Patient and Family Advocacy in Rare Diseases. 2021(1536-0075 (Electronic))
25. Dunkle M, Pines W, Saltonstall PL. Advocacy Groups and Their Role in Rare Diseases Research. In: Posada de la Paz M, Groft SC, eds. Rare Diseases Epidemiology. Dordrecht: Springer Netherlands 2010:515-25.
26. Scottish Medicines Consortium. Patient and Clinician Engagement (PACE) process [Available from: https://www.scottishmedicines.org.uk/how-we-decide/pace/2023.
27. Côté A, Keating B. What Is Wrong with Orphan Drug Policies? *Value in Health* 2012;15(8):1185-91. doi: https://doi.org/10.1016/j.jval.2012.09.004
28. Igho JO, Elizabeth AS, Matthew JT, et al. Effectiveness, safety and costs of orphan drugs: an evidence-based review. *BMJ Open* 2015;5(6):e007199. doi: 10.1136/bmjopen-2014-007199
29. Disclosure UK. ABPI Patient Organisations database 2021 [Available from: https://search.disclosureuk.org.uk/.
30. PMCPA. ABPI Code of Practice 2021 [Available from: https://www.pmcpa.org.uk/the-code/2021-interactive-abpi-code-of-practice/.
31. Disclosure UK. ABPI Patient Organisations database 2021 [Available from: https://search.disclosureuk.org.uk/2020.
32. HMRC. HMRC yearly average and spot rates: HM Revenue and Customs; [Available from: https://www.gov.uk/government/publications/exchange-rates-for-customs-and-vat-yearly.
33. HMRC. HMRC yearly average and spot rates: HM Revenue and Customs; 2022 [Available from: https://www.gov.uk/government/publications/exchange-rates-for-customs-and-vat-yearly.
34. Orphanet. The portal for rare diseases and orphan drugs 2022 [Available from: https://www.orpha.net/consor/cgi-bin/Disease_Search_Simple.php?lng=EN.
35. Rothman SM, Raveis VH, Friedman A, et al. Health Advocacy Organizations and the Pharmaceutical Industry: An Analysis of Disclosure Practices. *American Journal of Public Health* 2011;101(4):602-09. doi: 10.2105/AJPH.2010.300027
36. European Medicines Agency. Criteria to be fulfilled by patient, consumer and healthcare professional organisations involved in European Medicines Agency (EMA) activities 2018 [Available from: https://www.ema.europa.eu/en/documents/regulatory-procedural-guideline/criteria-be-fulfilled-patient-consumer-healthcare-professional-organisations-involved-european_en.pdf.
37. NICE. Policy on declaring and managing interests for NICE advisory committees, 2018.
38. WHO. ICD-11 for Mortality and Morbidity Statistics 2022 [Available from: https://icd.who.int/browse11/l-m/en#/http://id.who.int/icd/entity/465177735?view=G0.
39. NIH U.S. National Library of Medicine. ClinicalTrials.gov [Available from: https://clinicaltrials.gov/2022.
40. Ozieranski P, Csanadi M, Rickard E, et al. Analysis of Pharmaceutical Industry Payments to UK Health Care Organizations in 2015. 2019(2574-3805 (Electronic))
41. Blood Cancer UK. Blood cancer types [Available from: https://bloodcancer.org.uk/understanding-blood-cancer/blood-cancer-types/2023.
42. Hughes DA, Poletti-Hughes J. Profitability and Market Value of Orphan Drug Companies: A Retrospective, Propensity-Matched Case-Control Study. 2016(1932-6203 (Electronic))
43. McCoy MS, Carniol M, Chockley K, et al. Conflicts of Interest for Patient-Advocacy Organizations. *New England Journal of Medicine* 2017;376(9):880-85. doi: 10.1056/NEJMsr1610625
44. Jones K. In whose interest? Relationships between health consumer groups and the pharmaceutical industry in the UK. 2008(1467-9566 (Electronic))

For peer review only - http://bmjopen.bmj.com/site/about/guidelines.xhtml

45. Mandeville KL, Barker R, Packham A, et al. Financial interests of patient organisations contributing to technology assessment at England's National Institute for Health and Care Excellence: policy review. *BMJ* 2019;364:k5300. doi: 10.1136/bmj.k5300
46. Mavris M, Le Cam Y. Involvement of patient organisations in research and development of orphan drugs for rare diseases in europe. 2012(1661-8769 (Print))
47. Bedlington N, Geissler J, Houyez F, et al. Role of Patient Organisations. In: Facey KM, Ploug Hansen H, Single ANV, eds. Patient Involvement in Health Technology Assessment. Singapore: Springer Singapore 2017:401-10.
48. European Medicines Agency. European public assessment reports (EPAR), 2022.
49. Baggott R, Jones K. The Big Society in an age of austerity: threats and opportunities for Health Consumer and Patients' Organizations in England. 2015(1369-7625 (Electronic))
50. Parvanova I, Gentilini A, Cushing J, et al. Safeguarding NICE from patient groups' conflicts of interest. *BMJ* 2023;381:p1243. doi: 10.1136/bmj.p1243
51. Lexchin J. Association between commercial funding of Canadian patient groups and their views about funding of medicines: An observational study. *PLOS ONE* 2019;14(2):e0212399. doi: 10.1371/journal.pone.0212399
52. McCoy MS, Emanuel EJ. Why There Are No "Potential" Conflicts of Interest. *JAMA* 2017;317(17):1721-22. doi: 10.1001/jama.2017.2308
53. Das S, Ungoed-Thomas J. Revealed: experts who praised new 'skinny jab' received payments from drug maker. *The Guardian* 2023.
54. Ozieranski P, Martinon L, Jachiet P-A, et al. Accessibility and quality of drug company disclosures of payments to healthcare professionals and organisations in 37 countries: a European policy review. *BMJ Open* 2021;11(12):e053138. doi: 10.1136/bmjopen-2021-053138
55. Vertex Pharmaceuticals Incorporated. Our Science [Available from: https://www.vrtx.com/our-science/2023.
56. Ozieranski P, Csanádi M, Rickard E, et al. Under-reported relationship: a comparative study of pharmaceutical industry and patient organisation payment disclosures in the UK (2012–2016). *BMJ Open* 2020;10(9):e037351. doi: 10.1136/bmjopen-2020-037351
57. Colombo C, Mosconi P, Villani W, et al. Patient organizations' funding from pharmaceutical companies: is disclosure clear, complete and accessible to the public? An Italian survey. *PLoS One* 2012;7(5):e34974. doi: 10.1371/journal.pone.0034974 [published Online First: 20120509]

For peer review only - http://bmjopen.bmj.com/site/about/guidelines.xhtml

Figure legend

Figure 1. Classification model to determine company interests in patient organisation funding Note: An interest is when there is, or could be perceived to be, an opportunity for a pharmaceutical company to benefit in the disease area where the patient organisation operates.

Figure 2. Cumulative value of payments by receiving patient organisation type and funding company in 2020
Note: Non-disease-specific patient organisations include organisations that could not be matched to specific ICD-11 codes or could not be classified as rare or non-rare, such as hospital charities, carers organisations, and hospices.

Figure 3. Cumulative value of payments by patient organisation type and therapeutic area from in 2020
Note: Non-disease-specific patient organisations include organisations that could not be matched to specific ICD-11 codes or could not be classified as rare or non-rare, such as hospital charities, carers organisations, and hospices.

For peer review only

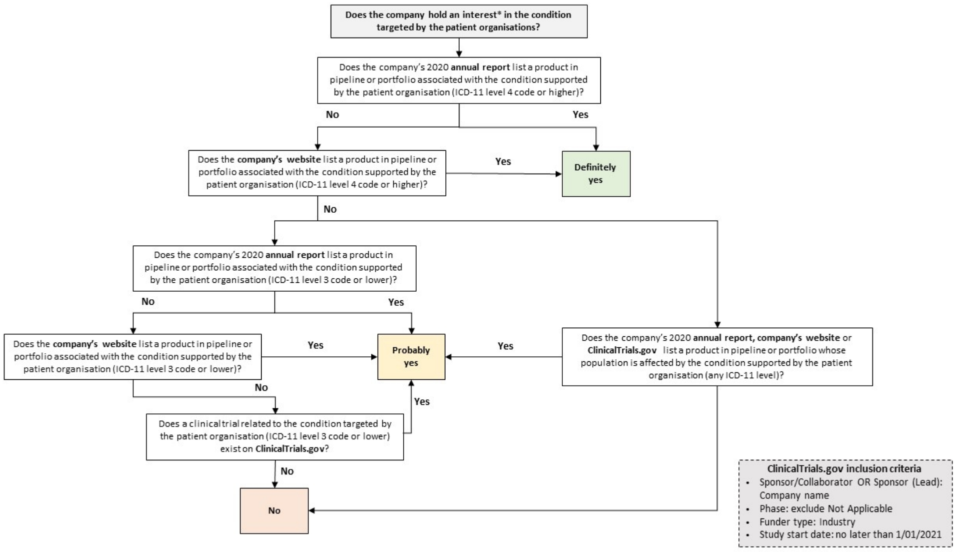

Caption: Classification model to determine company interests in patient organisation funding

Notes: An interest is when there is, or could be perceived to be, an opportunity for a pharmaceutical company to benefit in the disease area where the patient organisation operates.

338x190mm (96 x 96 DPI)

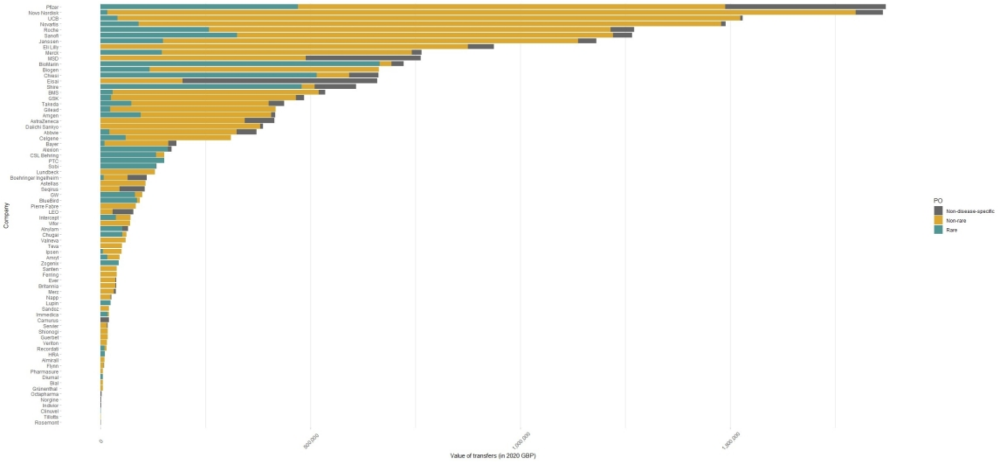

Cumulative value of payments by receiving patient organisation type and funding company in 2020

Note: Non-disease-specific patient organisations include organisations that could not be matched to specific ICD-11 codes or could not be classified as rare or non-rare, such as hospital charities, carers organisations, and hospices.

650x299mm (96 x 96 DPI)

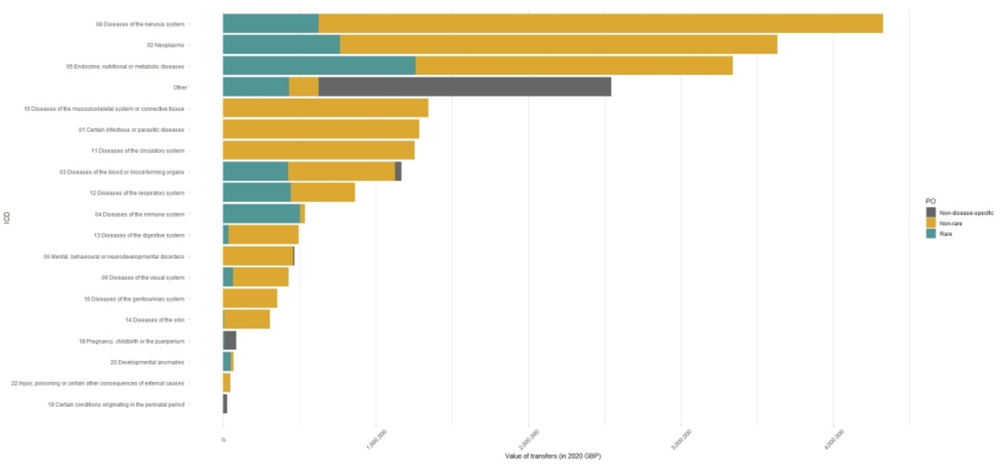

Cumulative value of payments by patient organisation type and therapeutic area from in 2020

Note: Non-disease-specific patient organisations include organisations that could not be matched to specific ICD-11 codes or could not be classified as rare or non-rare, such as hospital charities, carers organisations, and hospices.

581x264mm (96 x 96 DPI)

Supplemental Material

Data collection

Payments

We retrieved data on 2020 payments from pharmaceutical companies to patient organisations from the following sources:

1) **Companies' websites**. Disclosing payments to patient organisations is a requirement of Clause 29 of the Association of British Pharmaceutical Industry (ABPI) Code of Practice.[1] Specifically, the ABPI requires companies to keep a public record of any payment made to patient organisations on their website for a minimum of three years following the payment.[1] Therefore, companies' website were our primary data source on payments to patient organisations.
2) **Disclosure UK HCOs database**. In light of a recent study unveiling that payments to patient organisations were misreported in the Disclosure UK database of payments to healthcare organisations (HCOs),[2] we also screened the 2020 Disclosure UK HCOs database for payments to patient organisations.

If payments were not disclosed in the company's website nor in the Disclosure UK HCOs database, we assumed that the company did not make any payments to patient organisations in 2020, as commonly assumed in the literature.[3]

One investigator (AG) extracted payment disclosures from the companies' websites. These comprised the name of the patient organisation, the year when the payment was made, the reason for the payment and its value in the currency reported by the disclosing company. The 2020 Disclosure UK HCOs database was also screened, and recipients were matched to standardised patient organisations names. To ensure the data's accuracy, the final database was scanned for duplicates, but no such instances were found. When reported in different currencies, such as United States Dollars (USD), Swiss Franc (CHF), Swedish Krona (SEK), Norwegian Krone (NKK) and Danish Krone (DKK), the value of the payment was converted to Great British Pounds (GBP), using the ONS historical yearly conversion rates. [4 5] Two in-kind payments with a monetary value of zero were excluded from the analysis. Further details on variables' cleaning and coding can be found in the Supplemental Material.

Therapeutic areas

Patient organisations' websites were also screened to understand the condition(s) they focused on. For example, in the case of *Blood Cancer UK*, their mission is to "*beat blood cancer*", therefore, the condition supported was coded as blood cancer.

After being identified as described above, conditions were further classified into rare and non-rare.

For peer review only - http://bmjopen.bmj.com/site/about/guidelines.xhtml

Conditions were considered rare if they appeared in the Orphanet database of rare diseases regardless of their classification level (group of disorders, disorders or subtypes of disorders).[6] For example, multiple myeloma appears in the Orphanet database of rare diseases, therefore a patient organisation focusing this condition would be categorised as rare-focused. When condition sub-types appeared in the Orphanet database, the patient organisation's website was screened to check whether its focus was on rare conditions. For example, *Metabolic Support UK's* motto is "*Your rare condition. Our common fight"* and was therefore assumed to be rare disease-focused. Conversely, should a patient organisation focus on a broader condition such as blood cancer with no sole focus on rare conditions, the organisation would be conservatively considered non-rare. While this approach was preferred as it did not require further assumptions, it entails that only more specialised patient organisation are considered as rare. Such approach might have led to the number and overall value of payments from pharmaceutical companies to rare diseases-focused patient organisations being underestimated, as these organisaitons are expected to get less payments than more generalist ones (e.g. multiple myeloma vs blood cancer).

A third category (*unclear)* was created for non-disease-specific patient organisations, such as coalition of charities or organisations focused on palliative care for terminally ill patients. This category was excluded from the main analyses, but sub-group analyses are reported at the end of the Supplemental Material.

Companies' interest

We developed a methodology to assess the extent to which a pharmaceutical company holds an interest in the disease supported by a patient organisation. For the purpose of this analysis, we adapted the definition of interest provided by NICE.[7] An interest is when there is, or could be perceived to be, an opportunity for a pharmaceutical company to benefit in the disease area where the patient organisation operates. This could include situations where the pharmaceutical company has a drug developed or in development for a condition supported by the patient organisation, or where a drug in the company's portfolio or pipeline is restricted to a specific population affected by the disease supported by the patient organisation.

As first step, the conditions supported by patient organisations were translated into ICD-11 codes using the online ICD-11 database.[8]

ICD-11 codes are mutually exclusive, exhaustive and are arranged as a single hierarchical tree. This means that specific diseases are nested within broader classifications. An example for multiple myeloma is shown in Table 1 below.

Table 1. Example of ICD-11 classification, Multiple myeloma

Hierarchy level	Condition	ICD-11 code
Level 1	Neoplasms	2
Level 2	Neoplasms of haematopoietic or lymphoid tissues	2A
Level 3	Mature B-cell neoplasms	2A8
Level 4	Plasma cell neoplasms	2A83
Level 5	Plasma cell myeloma	2A83.1

For peer review only - http://bmjopen.bmj.com/site/about/guidelines.xhtml

In this example, multiple myeloma is nested within *Plasma cell myeloma*, who is in its turn nested within *Plasma cell neoplasms* and so on up to *Neoplasms*.

Subsequently, companies' annual reports, website and the ClinicalTrials.gov database were searched to assess whether the each company had an interest in the condition supported by the patient organisation receiving the payment. The diagram in the main document (Figure 1) schematically illustrates the approach taken to understand whether the company definitely, probably or did not have an interest in the condition. Figure 1 below illustrates the source of companies' interests.

For example, if *Company X* reports in its annual report having a drug in development for multiple myeloma and transferred a sum of money to *Blood Cancer UK*, this would be coded as *probably yes*, as the company has a product in its pipeline or portfolio associated with a condition supported by the patient organisation. In this case, the ICD-11 level would be 2, *Neoplasms of haematopoietic or lymphoid tissue*, under which multiple myeloma is nested. Conversely, should *Company X* have made a payment to *Myeloma UK*, this would have been coded as *definitely yes*, as there is perfect alignment between the condition supported by the patient organisation and by *Company X's* drug.

Situations where a company's interest in a certain condition could not be identified indicate an impossibility of identifying such link, rather than the lack thereof.

Figure 1. Source of companies interests

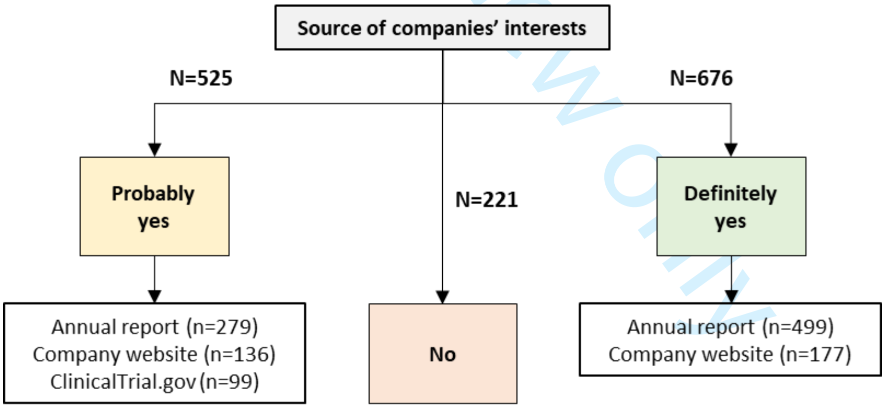

For peer review only - http://bmjopen.bmj.com/site/about/guidelines.xhtml

Variables cleaning and coding

Table 2. Description of key variables in payment database

Variables name	Description	Details
**Company**	Standardised company name	Company name as reported on company website and/or on HCOs database.<br>Two mergers involving companies included in our analysis—BMS and Celgene, and Takeda and Shire—were completed prior to 2020. Although these companies had merged, we treated them as separate entities because their disclosures were reported separately even after the acquisition.
**ABPI member**	ABPI membership of company; *source: ABPI full members list*	0 = not ABPI member, 1 = ABPI member
**Company_condition**	Concatenation of company name and disease area targeted by the patient organisation	Concatenation used for coding and analysis purposes
**Company interest**	Whether the company hold an interest* in the condition targeted by the patient organsiation	- **Definitely yes**: the company's annual report or website list a product for the condition targeted by the patient organisation in its portfolio/pipeline (ICD-11 level 4 or above)<br>- **Probably yes**: the company's annual report or website list a product for the condition targeted by the patient organisation in its portfolio/pipeline OR a clinical trial for which the company is sponsor is listed for the disease targeted by the patient organisation OR a drug in the company's pipeline/portfolio is restricted to a specific population affected by the disease targeted by the patient organisation (ICD-11 level 3 or below)<br>- **No** : None of the above
**Source**	Source of company interest variable	Annual report, company website, ClinicalTrials.gov, none
**Name of PO**	Name of patient organization as reported by companies in disclosure report	-
**Standardised PO name**	Standardised name of patient organization to avoid duplicates and inconsistencies	For coding purposes, names of patient organisations were standardised. The following steps were taken:<br>1. Patient organisations' names for typos, abbreviations, spelling mistakes and duplicated within the companies' disclosures (e.g. Crohn's & Colitis UK and CCUK would both be standardized to Crohn's and Colitis UK);<br>2. If the patient organisation changed name over time, the latest name on record was used;

For peer review only - http://bmjopen.bmj.com/site/about/guidelines.xhtml

		3. If the two patient organisations merged over the study period, the name of the merged entity was used (e.g. the British Lung Foundation and Asthma UK merged into Asthma + Lung UK);<br>4. Separate entries were made for patient organsiations under the same umbrella but focusing on different geographical entities (e.g. Alzheimer UK vs Alzheimer Scotland)
**Reason for exclusion**	Reason why the organisation was excluded from the analysis	- Not UK organisation (not aligned with geographical scope e.g. Irish, US-based);<br>- For profit company (not aligned with definition of patient organization used in the study);<br>- Missing information (organisations for whose nature is unclear i.e. patient organisation website could not be identified)
**ICD-11**	Classification of disease targeted by the patient organisation according to the WHO ICD-11; *source: ICD WHO website*	General classification (ICD-11 chapters)<br>*See Excel file, Inputs tab*
**Condition**	Condition targeted by patient organisation as reported on website	e.g. Blood Cancer UK would fall under ICD-11 code 02 Neoplasms, with *blood cancer* being the condition
**Charity number (if any)**	Charity number as reported in the organization website or as reported in the England and Wales Charity Commission website	When both England/Wales and Scotland or Northern Ireland charity numbers were provided, the former was chosen. Scotland and Northern Ireland charity numbers were reported only when those for England/Wales were missing
**Company number (if charity number missing)**	Company number as reported in the organization website or as reported in the Government Company Information Service wesbite if the patient organization cannot be found in the charity commission database (e.g. limited by guarantee company)	When both England/Wales and Scotland or Northern Ireland charity numbers were provided, the former was chosen. Scotland and Northern Ireland charity numbers were reported only when those for England/Wales were missing
**Link**	Link of patient organisation website	-
**Rare disease**	Whether the condition or one of the conditions targeted by the patient organisation is considered as rare	A condition was considered as rare if it under at least one of the following criteria:<br>1. The condition is listed in Orphanet list of rare diseases regardless of its ICD-11 level classification;<br>2. In their website, the patient organisation explicitly describe the disease they target as rare (e.g. *Metabolic Support UK's* motto is "*Your rare condition. Our common fight"* and was therefore assumed to be rare disease-focused)

For peer review only - http://bmjopen.bmj.com/site/about/guidelines.xhtml

**Details of payment**	Details of payment as reported by companies in disclosure report	-
**Country**	Country of payment	The country considered for the entire database is the UK
**Year**	Year of payment	2020
**Currency**	Currency of payment	Currency the payment is reported in the disclosure reports (i.e. EUR, GBP, USD, CHF, SEK, NKK)
**Currency_year**	Concatenation of currency and year of payment for conversion purposes	-
**Value of payment**	Value of payment in original currency as reported by companies in disclosure report	In-kind payments were removed from the analysis as no monetary value could be associated to such payments
**Value in 2020 pounds**	GBP converted value of payment	See details in *Inputs* sheet

*An interest is when there is, or could be perceived to be, an opportunity for a pharmaceutical company to benefit in the disease area where the patient organisation operates.

For peer review only

1 **Disclosure details**

2 **Table 3. Reporting of payments to patient organizations by pharmaceutical companies:**
3 **comparison of company websites and Disclosure UK HCOs database**

Company	Company website only	HCOs database only	Both
Abbvie	X		
Alexion	X		
Almirall	X		
Alnylam			X
Amgen			X
Amryt	X		
Astellas			X
AstraZeneca			X
BMS			X
Bayer			X
Bial		X	
BioMarin			X
Biogen	X		
BlueBird	X		
Boehringer Ingelheim			X
Britannia			X
CSL Behring	X		
Camurus			X
Celgene			X
Chiesi			X
Chugai	X		
Clinuvel	X		
Daiichi Sankyo			X
Diurnal	X		
Eisai			X
Eli Lilly			X
Ever			X
Ferring		X	
Flynn		X	
GSK			X
GW			X
Gilead		X	
Grünenthal			X
Guerbet		X	
HRA		X	
Immedica	X		
Indivior	X		
Intercept	X		
Ipsen		X	
Janssen			X

For peer review only - http://bmjopen.bmj.com/site/about/guidelines.xhtml

**LEO**	X		
**Lundbeck**			X
**Lupin**	X		
**MSD**			X
**Merck**			X
**Merz**			X
**Napp**			X
**Norgine**		X	
**Novartis**			X
**Novo Nordisk**			X
**Octapharma**		X	
**PTC**	X		
**Pfizer**			X
**Pharmasure**		X	
**Pierre Fabre**			X
**Recordati**	X		
**Roche**			X
**Rosemont**			X
**Sandoz**		X	
**Sanofi**			X
**Santen**	X		
**Seqirus**	X		
**Servier**	X		
**Shionogi**		X	
**Shire**			X
**Sobi**	X		
**Takeda**			X
**Teva**		X	
**Tillotts**	X		
**UCB**			X
**Valneva**	X		
**Veriton**		X	
**Vifor**			X
**Zogenix**	X		
**Total (n;%)**	24; 32%	14; 19%	36; 49%

Table 4. Reporting of payments to patient organizations by pharmaceutical companies: payments disclosed on company websites and Disclosure UK HCOs database

Company	Payments reported on company website (£)	Payments reported on HCOs database (£)	Total
**Abbvie**	£ 371,503	£ -	£ 371,503
**Alexion**	£ 168,925	£ -	£ 168,925
**Almirall**	£ 9,775	£ -	£ 9,775
**Alnylam**	£ 51,559	£ 14,050	£ 65,609
**Amgen**	£ 347,757	£ 68,845	£ 416,602

For peer review only

**Amryt**	£	45,413	£	-	£	45,413
**Astellas**	£	94,583	£	13,071	£	107,654
**AstraZeneca**	£	326,201	£	88,175	£	414,376
**BMS**	£	517,082	£	17,750	£	534,832
**Bayer**	£	171,758	£	9,098	£	180,856
**Bial**	£	-	£	5,500	£	5,500
**BioMarin**	£	411,912	£	310,455	£	722,367
**Biogen**	£	663,142	£	-	£	663,142
**BlueBird**	£	94,000	£	-	£	94,000
**Boehringer Ingelheim**	£	79,762	£	30,000	£	109,762
**Britannia**	£	35,000	£	2,030	£	37,030
**CSL Behring**	£	152,192	£	-	£	152,192
**Camurus**	£	13,168	£	6,500	£	19,668
**Celgene**	£	310,329	£	640	£	310,969
**Chiesi**	£	602,259	£	60,000	£	662,259
**Chugai**	£	62,092	£	-	£	62,092
**Clinuvel**	£	1,000	£	-	£	1,000
**Daiichi Sankyo**	£	57,879	£	329,385	£	387,264
**Diurnal**	£	6,000	£	-	£	6,000
**Eisai**	£	476,271	£	183,207	£	659,478
**Eli Lilly**	£	874,288	£	62,690	£	936,978
**Ever**	£	18,934	£	18,934	£	37,867
**Ferring**	£	-	£	38,000	£	38,000
**Flynn**	£	-	£	8,002	£	8,002
**GSK**	£	325,410	£	159,064	£	484,474
**GW**	£	98,788	£	303	£	99,091
**Gilead**	£	-	£	417,448	£	417,448
**Grünenthal**	£	4,200	£	1,000	£	5,200
**Guerbet**	£	-	£	17,000	£	17,000
**HRA**	£	-	£	10,000	£	10,000
**Immedica**	£	19,954	£	-	£	19,954
**Indivior**	£	1,200	£	-	£	1,200
**Intercept**	£	71,712	£	-	£	71,712
**Ipsen**	£	-	£	50,050	£	50,050
**Janssen**	£	1,170,768	£	10,000	£	1,180,768
**LEO**	£	78,633	£	-	£	78,633
**Lundbeck**	£	89,400	£	40,309	£	129,709
**Lupin**	£	24,000	£	-	£	24,000
**MSD**	£	537,632	£	225,287	£	762,919
**Merck**	£	763,885	£	1,000	£	764,885
**Merz**	£	31,114	£	5,789	£	36,903
**Napp**	£	8,000	£	18,020	£	26,020
**Norgine**	£	-	£	1,240	£	1,240
**Novartis**	£	1,442,037	£	46,812	£	1,488,849
**Novo Nordisk**	£	452,113	£	1,411,598	£	1,863,711

For peer review only - http://bmjopen.bmj.com/site/about/guidelines.xhtml

**Octapharma**	£ -	£ 2,995	£ 2,995
**PTC**	£ 151,433	£ -	£ 151,433
**Pfizer**	£ 1,360,510	£ 509,793	£ 1,870,303
**Pharmasure**	£ -	£ 6,000	£ 6,000
**Pierre Fabre**	£ 50,010	£ 34,096	£ 84,106
**Recordati**	£ 14,500	£ -	£ 14,500
**Roche**	£ 1,169,578	£ 101,395	£ 1,270,973
**Rosemont**	£ 200	£ 200	£ 400
**Sandoz**	£ -	£ 20,000	£ 20,000
**Sanofi**	£ 1,262,802	£ 3,825	£ 1,266,627
**Santen**	£ 38,170	£ -	£ 38,170
**Seqirus**	£ 105,000	£ -	£ 105,000
**Servier**	£ 17,163	£ -	£ 17,163
**Shionogi**	£ -	£ 17,000	£ 17,000
**Shire**	£ 555,244	£ 53,980	£ 609,224
**Sobi**	£ 132,988	£ -	£ 132,988
**Takeda**	£ 420,549	£ 17,270	£ 437,819
**Teva**	£ -	£ 51,410	£ 51,410
**Tillotts**	£ 830	£ -	£ 830
**UCB**	£ 1,493,896	£ 35,378	£ 1,529,274
**Valneva**	£ 59,512	£ -	£ 59,512
**Veriton**	£ -	£ 15,000	£ 15,000
**Vifor**	£ 58,083	£ 12,000	£ 70,083
**Zogenix**	£ 43,625	£ -	£ 43,625
**Total (£;%)**	£18,015,722; 80%	£4,561,593; 20%	£22,577,314; 100%

1

For peer review only

Table 5. Companies' commercial interests by ICD-11 codes according to 2020 payments

	ICD-11																	
**Company**	**01**	**02**	**03**	**04**	**05**	**06**	**08**	**09**	**11**	**12**	**13**	**14**	**15**	**16**	**18**	**19**	**20**	**22**	**Other**
**Abbvie**	1	1	0	0	0	0	1	0	0	0	1	1	1	0	0	0	0	0	0
**Alexion**	0	0	1	0	1	0	0	0	0	0	0	0	0	0	0	0	0	0	0
**Almirall**	0	0	0	0	0	0	0	0	0	0	0	1	0	0	0	0	0	0	0
**Alnylam**	0	0	0	0	1	0	0	0	0	0	0	0	0	0	0	0	0	0	0
**Amgen**	0	1	1	0	0	0	0	0	0	0	1	1	1	0	0	0	0	0	0
**Amryt**	0	0	0	0	1	0	0	0	0	0	0	0	0	0	0	0	0	0	0
**Astellas**	0	1	0	0	0	0	0	0	0	0	0	0	0	0	0	0	0	0	0
**AstraZeneca**	0	1	0	0	1	0	0	0	1	0	0	0	0	1	0	0	0	0	0
**BMS**	0	1	0	0	0	0	1	0	1	0	0	0	1	0	0	0	0	0	0
**Bayer**	0	1	0	0	0	0	0	1	1	0	0	0	0	0	0	0	0	0	0
**Bial**	0	0	0	0	0	0	1	0	0	0	0	0	0	0	0	0	0	0	0
**BioMarin**	0	0	1	0	1	0	0	0	0	0	0	0	0	0	0	0	0	0	0
**Biogen**	0	0	0	0	0	0	1	1	0	0	0	0	1	0	0	0	0	0	0
**BlueBird**	0	0	1	0	0	0	1	0	0	0	0	0	0	0	0	0	0	0	0
**Boehringer Ingelheim**	0	0	0	1	0	0	0	0	0	1	0	0	0	0	0	0	0	0	0
**Britannia**	0	0	0	0	0	0	1	0	0	0	0	0	0	0	0	0	0	0	0
**CSL Behring**	1	0	1	1	0	0	0	0	0	0	0	0	0	0	0	0	0	0	0
**Camurus**	0	0	0	0	0	0	0	0	0	0	0	0	0	0	0	0	0	0	0
**Celgene**	0	1	0	0	0	0	0	0	0	0	0	0	0	0	0	0	0	0	0
**Chiesi**	0	0	1	0	1	0	0	0	0	1	0	0	0	0	0	0	0	0	0
**Chugai**	0	0	1	0	0	0	0	0	0	0	0	0	1	0	0	0	0	0	0
**Clinuvel**	0	0	0	0	1	0	0	0	0	0	0	0	0	0	0	0	0	0	0
**Daiichi Sankyo**	0	1	0	0	0	0	0	0	1	0	0	0	0	0	0	0	0	0	0
**Diurnal**	0	0	0	0	1	0	0	0	0	0	0	0	0	0	0	0	0	0	0

**Eisai**	0	1	0	0	0	1	1	0	0	0	0	0	0	0	0	0	0	0	0
**Eli Lilly**	0	1	0	0	1	0	1	0	0	0	0	1	1	0	0	0	0	0	0
**Ever**	0	0	0	0	0	0	1	0	0	0	0	0	0	0	0	0	0	0	0
**Ferring**	0	1	0	0	0	0	0	0	0	0	0	0	0	1	0	0	0	0	0
**Flynn**	0	0	0	0	0	1	0	0	0	0	0	0	0	0	0	0	0	0	0
**GSK**	1	1	0	1	0	0	0	0	1	0	0	0	0	0	0	0	0	0	0
**GW**	0	0	0	0	0	0	0	0	0	0	0	0	0	0	0	0	0	0	0
**Gilead**	1	1	0	0	0	0	0	0	0	0	1	0	0	0	0	0	0	0	0
**Grünenthal**	0	0	0	0	0	0	0	0	0	0	0	0	0	0	0	0	0	0	0
**Guerbet**	0	0	0	0	0	0	0	0	0	0	0	0	0	1	0	0	0	0	0
**HRA**	0	0	0	0	0	0	0	0	0	0	0	0	0	0	0	0	0	0	0
**Immedica**	0	0	0	0	0	0	0	0	0	0	0	0	0	0	0	0	0	0	0
**Indivior**	0	0	0	0	0	0	0	0	0	0	0	0	0	0	0	0	0	0	0
**Intercept**	0	0	0	0	0	0	0	0	0	0	1	0	0	0	0	0	0	0	0
**Ipsen**	0	1	0	0	0	0	1	0	0	0	0	0	0	0	0	0	0	0	0
**Janssen**	1	1	0	0	0	0	0	0	1	0	1	1	0	0	0	0	0	0	0
**LEO**	0	0	0	0	0	0	0	0	1	0	0	1	0	0	0	0	0	0	0
**Lundbeck**	0	0	0	0	0	1	1	0	0	0	0	0	0	0	0	0	0	0	0
**Lupin**	0	0	0	0	0	0	1	0	0	0	0	0	0	0	0	0	0	0	0
**MSD**	1	1	0	0	0	0	0	0	0	0	0	0	0	0	0	0	0	0	0
**Merck**	0	1	0	0	0	0	1	0	0	0	0	0	0	1	0	0	0	0	0
**Merz**	0	0	0	0	0	0	0	0	0	0	0	0	0	0	0	0	0	0	0
**Napp**	0	0	0	0	1	0	0	0	0	0	0	0	0	0	0	0	0	0	0
**Norgine**	0	0	0	0	0	0	0	0	0	0	0	0	0	0	0	0	0	0	0
**Novartis**	0	1	1	0	0	0	1	1	1	0	0	1	1	0	0	0	0	0	0
**Novo Nordisk**	0	0	1	0	1	0	0	0	0	0	0	0	0	0	0	0	1	0	0
**Octapharma**	0	0	0	0	0	0	0	0	0	0	0	0	0	0	0	0	0	0	0
**PTC**	0	0	0	0	0	0	1	0	0	0	0	0	0	0	0	0	0	0	0
**Pfizer**	1	1	1	0	1	0	1	0	1	0	1	0	1	0	0	0	1	0	0
**Pharmasure**	0	0	0	0	0	0	0	0	0	0	0	0	0	1	0	0	0	0	0

For peer review only - http://bmjopen.bmj.com/site/about/guidelines.xhtml

**Pierre Fabre**	0	1	0	0	0	0	0	0	0	0	0	1	0	0	0	0	0	0	0
**Recordati**	0	0	0	0	1	0	0	0	0	0	0	0	0	0	0	0	0	0	0
**Roche**	0	1	0	0	0	0	1	0	0	1	1	0	0	0	1	0	0	0	0
**Rosemont**	0	0	0	0	0	0	0	0	0	0	0	0	0	0	0	0	0	0	0
**Sandoz**	0	0	0	0	0	0	0	0	0	0	0	0	0	0	0	0	0	0	0
**Sanofi**	1	1	1	1	1	0	1	0	1	0	0	1	1	1	0	0	0	0	0
**Santen**	0	0	0	1	0	0	0	1	0	0	0	0	0	0	0	0	0	0	0
**Seqirus**	0	0	0	0	0	0	0	0	0	0	0	0	0	0	0	0	0	0	0
**Servier**	0	1	0	0	0	0	0	0	0	0	0	0	0	0	0	0	0	0	0
**Shionogi**	0	0	0	0	0	0	0	0	0	0	0	0	0	0	0	0	0	0	0
**Shire**	0	0	1	1	1	1	0	0	0	0	0	0	0	0	0	0	0	0	0
**Sobi**	0	1	1	0	1	0	0	0	0	0	0	0	0	0	0	0	0	0	0
**Takeda**	0	1	0	0	1	0	0	0	0	0	1	0	0	0	0	0	0	0	0
**Teva**	0	0	0	0	0	0	0	0	0	0	0	0	0	0	0	0	0	0	0
**Tillotts**	0	0	0	0	0	0	0	0	0	0	1	0	0	0	0	0	0	0	0
**UCB**	0	0	1	0	0	0	1	0	0	0	0	1	1	0	0	0	0	0	0
**Valneva**	1	0	0	0	0	0	0	0	0	0	0	0	0	0	0	0	0	0	0
**Veriton**	0	0	0	0	0	0	0	0	0	0	0	0	0	0	0	0	0	0	0
**Vifor**	0	0	0	0	0	0	0	0	0	0	0	0	0	0	0	0	0	0	0
**Zogenix**	0	0	0	0	0	0	1	0	0	0	0	0	0	0	0	0	0	0	0

Notes: This table reflects whether companies had a definite or probable interest in the ICD-11 code based on their pipeline or portfolio (1 = yes, 0 = no). Please note that companies' interests were opportunistically screened only in disease areas where they made a payment to a specific patient organisation, and therefore this table should not be considered exhaustive. The table refers payments made in 2020 only.

Legend: 01 Certain infectious or parasitic diseases; 02 Neoplasms; 03 Diseases of the blood or blood-forming organs; 04 Diseases of the immune system; 05 Endocrine, nutritional or metabolic diseases; 06 Mental, behavioural or neurodevelopmental disorders; 08 Diseases of the nervous system; 09 Diseases of the visual system; 11 Diseases of the circulatory system; 12 Diseases of the respiratory system; 13 Diseases of the digestive system; 14 Diseases of the skin; 15 Diseases of the musculoskeletal system or connective tissue; 16 Diseases of the genitourinary system; 18 Pregnancy, childbirth or the puerperium; 19 Certain conditions originating in the perinatal period; 20 Developmental anomalies; 22 Injury, poisoning or certain other consequences of external causes; Other. Other indicates disease areas where patient organisations operate that could not be classified as any ICD-11 codes.

For peer review only - http://bmjopen.bmj.com/site/about/guidelines.xhtml

Table 6. List of patient organisations receiving payments in 2020

Standardised name	Charity number	Link
Acacia Mews Care Home	1174346	https://www.nhs.uk/services/Careproviders/Overview/DefaultView.aspx?id=47011
Action Bladder Cancer UK	1164374	https://actionbladdercanceruk.org/
Action for Pulmonary Fibrosis	1152399	https://www.actionpf.org/
Action On Pre-Eclampsia	1013557	https://action-on-pre-eclampsia.org.uk/
Action on Smoking and Health - Wales	1120834	https://ash.wales/
Action Duchenne	1101971	https://www.actionduchenne.org/
Adfam	1067428	https://adfam.org.uk/
Africa Advocacy Foundation	1164778	https://www.africadvocacy.org/
African-Caribbean Leukaemia Trust	1119516	https://aclt.org/
Age UK	1128267	https://www.ageuk.org.uk/
Alex - The Leukodystrophy Charity	1106008	https://www.alextlc.org/
ALK Positive Lung Cancer	1181171	https://www.alkpositive.org.uk/
Alkaptonuria Society	1101052	https://akusociety.org/
Allergy UK	1094231	https://www.allergyuk.org/
Alliance for Heart Failure	N/A	https://allianceforheartfailure.org/
Alzheimer Scotland	SC022315	https://www.alzscot.org/
Alzheimer’s Support	1048314	https://www.alzheimerswiltshire.org.uk/
Alzheimer's Research UK	1077089	https://www.alzheimersresearchuk.org/
Alzheimer's Society	296645	https://www.alzheimers.org.uk/
Amyloidosis Patients Association	1183624	https://register-of-charities.charitycommission.gov.uk/charity-details/?regid=1183624&subid=0
Anthony Nolan	803716	https://www.anthonynolan.org/
Anticoagulation UK	1090250	https://register-of-charities.charitycommission.gov.uk/charity-details/?regid=1090250&subid=0
AOFAC Foundation	1162155	https://aofacfoundation.org/
Aplastic Anaemia Trust	1107539	https://www.theaat.org.uk/
APS Support UK	1138116	https://aps-support.org.uk/
Arthritis and Musculoskeletal Alliance	1108851	http://arma.uk.net/
Aspens	1171446	https://www.aspens.org.uk/
Association for Glycogen Storage Disease	1132271	https://agsd.org.uk/
Asthma + Lung UK	326730	https://www.asthma.org.uk/
Astriid	1176645	https://astriid.org/
Atrial Fibrillation Association	1122442	Supporting children terminally ill
Axial Spondylitis International Federation	1173902	https://asif.info/
Baby Lifeline	1006457	https://www.babylifeline.org.uk/
Bath Institute for Rheumatic Diseases	1040650	https://www.birdbath.org.uk/

For peer review only - http://bmjopen.bmj.com/site/about/guidelines.xhtml

Batten Disease Family Association	1084908	http://www.bdfa-uk.org.uk/
Bipolar UK	293340	https://www.bipolaruk.org/
Bladder Health UK	1149973	https://bladderhealthuk.org/
Bliss	1002973	https://www.bliss.org.uk/
Blood Cancer Alliance	N/A	https://www.bloodcanceralliance.org/
Blood Cancer UK	216032	https://bloodcancer.org.uk/
BME Cancer Communities	1182806	https://www.bmecancer.com/
Bowel Cancer UK	1071038	https://www.bowelcanceruk.org.uk/
Brains Trust	1114634	https://brainstrust.org.uk/
Breast Cancer Haven (The Haven)	3291851	https://www.breastcancerhaven.org.uk/
Breast Cancer Now	1160558	https://breastcancernow.org/
British Association ofr the Study of the Liver	1106320	https://www.basl.org.uk/
British Heart Foundation	225971	https://www.bhf.org.uk/
British Inherited Metabolic Disease Group	1184024	https://www.bimdg.org.uk/site/index.asp
British Liver Trust	298858	https://britishlivertrust.org.uk/
British Paediatric Neurology Association	1159115	https://bpna.org.uk/
British Porphyria Association	1089609	http://porphyria.org.uk/
British Skin Foundation	1171373	https://www.britishskinfoundation.org.uk/
British Society for Heart Failure	1075720	https://www.bsh.org.uk/
British Society of Echocardiography	1093808	https://www.bsecho.org/
British Thyroid Foundation	1006391	https://www.btf-thyroid.org/
Cambridge Rare Disease Network	1166365	https://www.camraredisease.org/
Cancer 52	7994413	https://www.cancer52.org.uk/
Cancer Black Care	1086465	https://www.cancerblackcare.org.uk/
Cancer Focus Northern Ireland	101307	https://cancerfocusni.org/
Cancer Research UK	1089464	https://www.cancerresearchuk.org/
Cancer Support Scotland	SC012867	https://www.cancersupportscotland.org/
Cancer Support UK	1105703	https://cancersupportuk.org/
CancerCare	1120048	https://cancercare.org.uk/
Cara Trust	328124	https://www.madtrust.org.uk/project/the-cara-trust/
Cardiomyopathy UK	1164263	https://www.cardiomyopathy.org/
Carers UK	N/A	https://www.carersuk.org/
Changing Faces	1011222	https://www.changingfaces.org.uk/
Child Growth Foundation	1172807	https://childgrowthfoundation.org/
Childhood Trust	1154032	https://www.childhoodtrust.org.uk/
Children’s Cancer and Leukaemia Group	1182637	https://www.cclg.org.uk/
Children’s HIV Association	1122356	https://www.chiva.org.uk/
Children’s Trust	288018	https://www.thechildrenstrust.org.uk/
Children's Burns Trust	1082084	https://www.cbtrust.org.uk/

For peer review only - http://bmjopen.bmj.com/site/about/guidelines.xhtml

Cholangiocarcinoma Charity	1091915	https://ammf.org.uk/
Chronic Lymphocytic Leukaemia Support Association	1178482	https://www.cllsupport.org.uk/
Coalition for Life-Course Immunisation	1182662	https://www.cl-ci.org/
Confederation of Meningitis Organisations	1091105	https://www.comomeningitis.org/
Contact a Family	284912	https://contact.org.uk/
Crohn's and Colitis UK	1117148	https://www.crohnsandcolitis.org.uk/
Cystic Fibrosis Trust	1079049	https://www.cysticfibrosis.org.uk/
Dementia UK	1039404	https://www.dementiauk.org/
Dementia Club UK	1168397	https://dementiaclubuk.org.uk/
Diabetes UK	215199	https://www.diabetes.org.uk/
Diana Award	1117288	https://diana-award.org.uk/
DMD Pathfinders	1155884	https://www.pathfindersalliance.org.uk/
Down Syndrome International	1091843	https://www.ds-int.org/
Downs Syndrome Association	1061474	https://www.downs-syndrome.org.uk/
Dravet Syndrome UK	1128289	https://www.dravet.org.uk/
DrugFAM	1123316	https://www.drugfam.co.uk/#
Duchenne UK	1147094	https://www.duchenneuk.org/
Dystonia UK	1062595	https://www.dystonia.org.uk/
East North Hertfordshire NHS Trust	1053338	https://www.enherts-tr.nhs.uk/
East Sussex Healthcare NHS Trust	1058599	https://www.esht.nhs.uk/
Ecancer	1176307	https://ecancer.org/en/
Eczema Outreach Support	SC042392	https://www.eos.org.uk/
Encephalitis Society	1087843	https://www.encephalitis.info/
Epilepsy Action	234343	https://www.epilepsy.org.uk/?gclid=CjwKCAiAsNKQBhAPEiwAB-I5zXsMWEMg1x_J-blYzK3HQGZujp-zoejjkEA_sYpKqYxct5LuE_sV6hoC1t8QAvD_BwE
Epilepsy Consortium Scotland	N/A	http://www.epilepsyconsortiumscotland.co.uk/
Epilepsy Research UK	1100394	https://epilepsyresearch.org.uk/
Epilepsy Scotland	SC000067	https://www.epilepsyscotland.org.uk/
Epilepsy Society	206186	https://epilepsysociety.org.uk/
Errol Mckellar Foundation	1181574	https://www.theerrolmckellarfoundation.com/
European Parkinson’s Disease Association	1163211	https://www.epda.eu.com/
Eve Appeal	1091708	https://eveappeal.org.uk/
Familial Hypercholesterolaemia Network	1170731	https://fheurope.org/
FareShare	1100051	https://fareshare.org.uk/
Favor UK	N/A	https://www.facesandvoicesofrecoveryuk.org/

For peer review only - http://bmjopen.bmj.com/site/about/guidelines.xhtml

Fertility Network UK	1099960	https://fertilitynetworkuk.org/
Fight Bladder Cancer	1157763	https://www.fightbladdercancer.co.uk/
Fight for Sight UK	1111438	https://www.fightforsight.org.uk/
Findacure	1149646	https://www.rarebeacon.org/about-us/our-journey/
Gauchers Association	1095657	https://www.gaucher.org.uk/
Gene People	1141583	https://genepeople.org.uk/
Genetic Alliance UK	1114195	https://geneticalliance.org.uk/
GetYourBellyOut	11276246	https://getyourbellyout.org.uk/
GIST Cancer UK	1129219	https://www.gistcancer.org.uk/
Global Action on Men's Health	1183428	https://gamh.org/
GO Girls	1179108	https://www.gogirlssupport.org/
Gorlin Syndrome Group	1197282	https://gorlingroup.org/
Guts UK	1137029	https://gutscharity.org.uk/
Haemachromatosis UK	1001307	https://www.haemochromatosis.org.uk/
Haemophilia Scotland	SC044298	https://haemophilia.scot/
Haemophilia Society	288260	https://haemophilia.org.uk/
Headway East London	1083910	https://headwayeastlondon.org/
Heart UK	1003904	https://www.heartuk.org.uk/
Heartburn Cancer UK	1136413	https://www.heartburncanceruk.org/
Helen & Douglas House	1085951	https://www.helenanddouglas.org.uk/
Hepatitis C Coalition	N/A	http://www.hepc-coalition.uk/
Hepatitis C Trust	1104279	http://hepctrust.org.uk/
Hereditary Angioedema UK	1152591	https://www.haeuk.org/
Hidradenitis Suppurativa Trust	1177819	https://painuk.org/members/charities/hidradenitis-suppurativa-trust/
Histiocytosis UK	1158789	https://www.histiouk.org/
HIV i-Base	1081905	https://i-base.info/
HIV Scotland	SC033951	https://www.hiv.scot/
Human Story Theatre	1173504	https://humanstorytheatre.com/about-us/
Huntington's Disease Association	296453	https://www.hda.org.uk/
Huntington's Disease Youth Organization	1145781	https://en.hdyo.org/
Immune Deficiency Patient Group of Wales	N/A	https://www.facebook.com/tommy.browne.idpgw/
Immune Thrombocytopenia Support Association	1064480	https://www.itpsupport.org.uk/index.php/en/
Independent Cancer Patients' Voice	1138456	http://www.independentcancerpatientsvoice.org.uk/
Intensive Care Society	1039236	https://www.ics.ac.uk/
International Alliance of Patients' Organizations	1155577	https://www.iapo.org.uk/
International Brain Tumour Alliance	N/A	https://theibta.org/
International Gaucher Alliance	6653373	https://gaucheralliance.org/home
International Headache Society	1042574	https://ihs-headache.org/en/
International Longevity Centre UK	1080496	https://ilcuk.org.uk/

For peer review only - http://bmjopen.bmj.com/site/about/guidelines.xhtml

International Niemann-Pick Disease Alliance	1150256	https://www.inpda.org/
International Patient Organisation for Primary Immunodeficiencies	1058005	https://ipopi.org/
Invisible Cafe	N/A	https://theinvisiblecafe.co.uk/
Isabel Hospice Limited	1046826	https://www.isabelhospice.org.uk/
Jo's Cervical Cancer Trust	1133542	https://www.jostrust.org.uk/
Juvenile Diabetes Research Foundation	295716	https://jdrf.org.uk/
Karen Clifford Skcin cancer charity	1150048	https://www.skcin.org/
Kent Autistic Trust	801965	https://www.kentautistictrust.org/
Kent MS Therapy Centre	801382	https://kentmstc.org.uk/
Kidney Cancer Support Network	1164238	https://actionkidneycancer.org/
Kidney Cancer UK	1120146	https://www.kcuk.org.uk/
Kidney Care UK	270288	https://www.kidneycareuk.org/
Kidney Research UK	252892	https://www.kidneyresearchuk.org/
Leukaemia CARE	1183890	https://www.leukaemiacare.org.uk/
Leukaemia UK	1154856	https://www.leukaemiauk.org.uk/
Liver4Life	1152618	https://www.liver4life.org.uk/
Lupus UK	1051610	https://www.lupusuk.org.uk/
Lymphoma Action	1068395	https://lymphoma-action.org.uk/about-us
Macmillan Cancer Support	261017	https://www.macmillan.org.uk/
Macular Society	2177039	https://www.macularsociety.org/
Maggie's Centres	SC024414	https://www.maggies.org/
Maypole Project	1120163	https://www.themaypoleproject.co.uk/
MDS UK Support Group	1145214	https://mdspatientsupport.org.uk/
Meath Epilepsy Charity	200359	https://www.meath.org.uk/
Medics 4 Rare Diseases	1183996	https://www.m4rd.org/history/
Melanoma Focus	1124716	https://melanomafocus.org/
Melanoma Fund	1085969	https://www.melanoma-fund.co.uk/
Melanoma UK	1157635	https://www.melanomauk.org.uk/
Memorylane Eastbourne	1163541	https://www.memorylaneeastbourne.co.uk/
Meningitis Now	803016	https://www.meningitisnow.org/
Meningitis Research Foundation	1091105	https://www.meningitis.org/
Menopause Support	N/A	https://menopausesupport.co.uk/
Mental Health UK	1170815	https://mentalhealth-uk.org/
Mersey Region Epilepsy Association	504366	https://www.epilepsymersey.org.uk/
Mesothelioma UK	1177039	https://www.mesothelioma.uk.com/
Metabolic Support UK	1089588	https://www.metabolicsupportuk.org/
Migraine Trust	1081300	https://migrainetrust.org/
Motor Neurone Disease Association	294354	https://www.mndassociation.org/
Mouth Cancer Foundation	1109298	https://www.mouthcancerfoundation.org/
MPN Voice	1160316	https://www.mpnvoice.org.uk/

For peer review only - http://bmjopen.bmj.com/site/about/guidelines.xhtml

Multiple Sclerosis International Federation	1105321	https://www.msif.org/
Multiple Sclerosis Society UK	1139257	https://www.mssociety.org.uk/
Multiple Sclerosis Therapy Centres	1031690	https://www.msntc.org.uk/
Multiple Sclerosis Trust	1088353	https://mstrust.org.uk/
Muscular Dystrophy UK	205395	https://www.musculardystrophyuk.org/
My Name'5 Doddie Foundation	SC047871	https://www.myname5doddie.co.uk/
Myeloma UK	SC026116	https://www.myeloma.org.uk/
National AIDS Map	1011220	https://www.aidsmap.com/
National AIDS Trust	297977	https://www.nat.org.uk/
National Attention Deficit Disorder Information and Support Service	N/A	https://www.nhs.uk/services/service-directory/the-national-attention-deficit-disorder-information-and-support-service-addiss/N10498901
National Axial Spondyloarthritis Society	1183175	https://nass.co.uk/
National Cancer Research Institute	1160609	https://www.ncri.org.uk/
National Eczema Society	1009671	https://eczema.org/
National Federation of Prostate Cancer Support Groups	1163152	https://tackleprostate.org/
National Kidney Federation	1106735	https://www.kidney.org.uk/
National Rheumatoid Arthritis Society	1134859	https://nras.org.uk/
National Voices	1057711	https://www.nationalvoices.org.uk/
NAZ	1014056	https://www.naz.org.uk/
Neuroendocrine Cancer UK	1092386	https://www.neuroendocrinecancer.org.uk/
Neurological Alliance	1039034	https://www.neural.org.uk/
New Life Counselling	NI005568	https://www.amh.org.uk/
NHS Charities Together	1186569	https://nhscharitiestogether.co.uk/
Nicole & Jessica Rich Foundation	N/A	https://thenicolerichfoundation.org.uk/
Niemann-Pick UK	1144406	https://www.npuk.org/
North Bristol NHS Trust	1055900	https://www.nbt.nhs.uk/
Oral Health Foundation	263198	https://www.dentalhealth.org/
Orchid	1080540	https://orchid-cancer.org.uk/
Osteoporosis Dorse	1023507	https://www.osteodorset.org.uk/
Ovacome	1159682	https://www.ovacome.org.uk/
Ovarian Cancer Action	1109743	https://ovarian.org.uk/
Over the Wall	1075361	https://www.otw.org.uk/
Pain Concern	SC023559	https://painconcern.org.uk/
Pancreatic Cancer Action	1137689	https://pancreaticcanceraction.org/
Pancreatic Cancer UK	1112708	https://www.pancreaticcancer.org.uk/
Parathyroid UK	N/A	https://parathyroiduk.org/
Parkinson’s UK	258197	https://www.parkinsons.org.uk/
Patient Information Forum	N/A	https://pifonline.org.uk/
Patients Association	1006733	https://www.patients-association.org.uk/

For peer review only - http://bmjopen.bmj.com/site/about/guidelines.xhtml

Patients On Intravenous and Nasogastric Nutrition Therapy	1157655	https://pinnt.com/Home.aspx
Paula Carr Diabetes Trust	801596	https://www.paulacarrdiabetestrust.co.uk/
PBC Foundation UK	SC025619	https://www.pbcfoundation.org.uk/
Pilgrims Hospice	293968	https://www.pilgrimshospices.org/
Pituitary Foundation	1058968	https://www.pituitary.org.uk/
Platelet Society	1172202	https://plateletsociety.co.uk/
Police Community Clubs of Great Britain	N/A	https://www.policecommunityclubs.org/
Polycystic Kidney Disease Charity	1160970	https://pkdcharity.org.uk/
Pompe Support Network	1186383	https://pompe.uk/
Positively UK	1007685	https://positivelyuk.org/
Primary Immunodeficiency UK	1193166	http://www.immunodeficiencyuk.org/
Progress Educational Trust	1139856	https://www.progress.org.uk/
Progressive Supranuclear Palsy Association	1037087	https://pspassociation.org.uk/
Prostate Cancer UK	1005541	https://prostatecanceruk.org/
Psoriasis Association	1180666	https://www.psoriasis-association.org.uk/
Pulmonary Hypertension Association UK	1120756	https://www.phauk.org/
Pumping Marvellous Foundation	1151848	https://www.pumpingmarvellous.org/
Rain Trust	N/A	https://www.nhs.uk/services/service-directory/rain-trust/N10972097
Rainbow Trust Children's Charity	1070532	https://www.rainbowtrust.org.uk/
Rapid Effective Assistance For Children With Potentially Terminal Illness	802440	https://reactcharity.org/
Red Rose Recovery	1152474	https://redroserecovery.org.uk/
Release	801118	https://www.release.org.uk/
Rethink Mental Illness	271028	https://www.rethink.org/
Retina UK	1153851	https://retinauk.org.uk/about/
Revive Multiple Sclerosis Support	SC022886	https://www.revivemssupport.org.uk/
Roy Castle Lung Cancer Foundation	1046854	https://roycastle.org/
Royal Free Charity	1165672	https://royalfreecharity.org/
Royal National Institute of Blind People	226227	https://www.rnib.org.uk/
Royal Osteoporosis Society	1102712	https://theros.org.uk/
Ruth Strauss Foundation	1183221	https://ruthstraussfoundation.com/
Salivary Gland Cancer UK	1182762	https://www.salivaryglandcancer.uk/
SANE	296572	https://www.sane.org.uk/
Sarcoma UK	1139869	https://sarcoma.org.uk/
Scleroderma and Raynauds UK	1161828	https://www.sruk.co.uk/
Scottish Drugs Forum	SC008075	https://www.sdf.org.uk/

For peer review only - http://bmjopen.bmj.com/site/about/guidelines.xhtml

Scottish Families Affected by Alcohol & Drugs	N/A	https://www.sfad.org.uk/
Scottish Huntington's Association	SC010985	https://hdscotland.org/
Shift.MS	1117194	https://shift.ms/
Shine Cancer Support	1146902	https://shinecancersupport.org/
Sickle Cell Society	1046631	https://www.sicklecellsociety.org/
Skin Conditions Campaign Sco tland	SC030004	https://www.disabilityscot.org.uk/organisat ion/skin-conditions-campaign-scotland/
Society for Mucopolysaccharide Diseases	1143472	https://www.mpssociety.org.uk/
Somerville Foundation	1138088	https://sfhearts.org.uk/
Sophia Forum	1131629	https://sophiaforum.net/
Spinal Muscular Atrophy Support UK	1106815	https://smauk.org.uk/
St Elizabeths Centre	1176777	https://www.stelizabeths.org.uk/
Stroke Association	211015	https://www.stroke.org.uk/
Swallows Head and Neck Cancer Charity	1149794	https://www.theswallows.org.uk/
Target Ovarian Cancer	1125038	https://targetovariancancer.org.uk/
Tenovus Cancer Care	1054015	https://www.tenovuscancercare.org.uk/
Terrence Higgins Trust	288527	https://www.tht.org.uk/
Thrombosis UK	1090540	https://thrombosisuk.org/news/post.php?s=2021-10-11-thrombosis-uk-winner-of-activity-of-the-year-award-2021
Tiny Tickers	1078114	https://www.tinytickers.org/
Together for Short Lives	1144022	https://www.togetherforshortlives.org.uk/
TRACTion Cancer Support	SCO048145	https://www.tractioncancersupport.org/
Trekstock	1132421	https://www.trekstock.com/
Trevi	1075433	https://trevi.org.uk/
Tuberous Sclerosis Association	1039549	https://tuberous-sclerosis.org/
Turner Syndrome Support Society	1080507	https://tss.org.uk/
Twins Trust	1076478	https://twinstrust.org/
UK Breast Cancer Group	1177296	https://ukbcg.org/
UK Lung Cancer Coalition	N/A	https://www.uklcc.org.uk/
UK Primary Immune-deficiency Patient Support	1148789	https://ukpips.org.uk/
UK Thalassaemia Society	275107	https://ukts.org/
University of Newcastle Institute of Neuroscience	N/A	https://www.ncl.ac.uk/medical-sciences/research/research-themes/neuroscience/
Urology Cancer Research and Education	1120887	http://www.ucare-oxford.org.uk/
Versus Arthritis	207711	https://www.versusarthritis.org/
Waldenstrom's Macroglobulinaemia UK	1187121	https://wmuk.org.uk/
White Chapel Mission	227905	https://whitechapel.org.uk/
Working with Cancer	9092152	https://workingwithcancer.co.uk/
Young Epilepsy	311877	https://www.youngepilepsy.org.uk/

For peer review only - http://bmjopen.bmj.com/site/about/guidelines.xhtml

Inclusion/exclusion of patient organisations

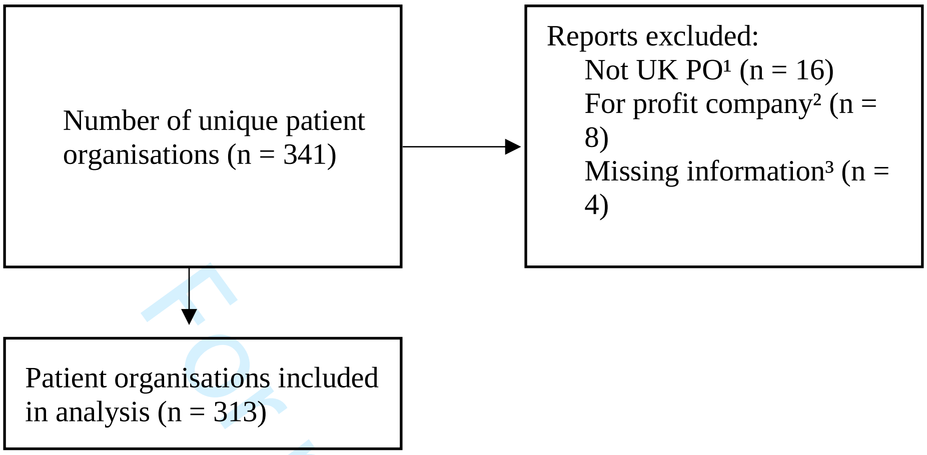

[1]Not aligned with geographical scope e.g. Irish, US-based
[2]Not aligned with EFPIA's definition of patient organisation
[3]Organisations for whose nature is unclear i.e. patient organisation website could not be identified

For peer review only

For peer review only - http://bmjopen.bmj.com/site/about/guidelines.xhtml

Additional tables and figures

Figure 2. Histogram of unique companies funding patient organisations in 2020, broken down by rarity of disease

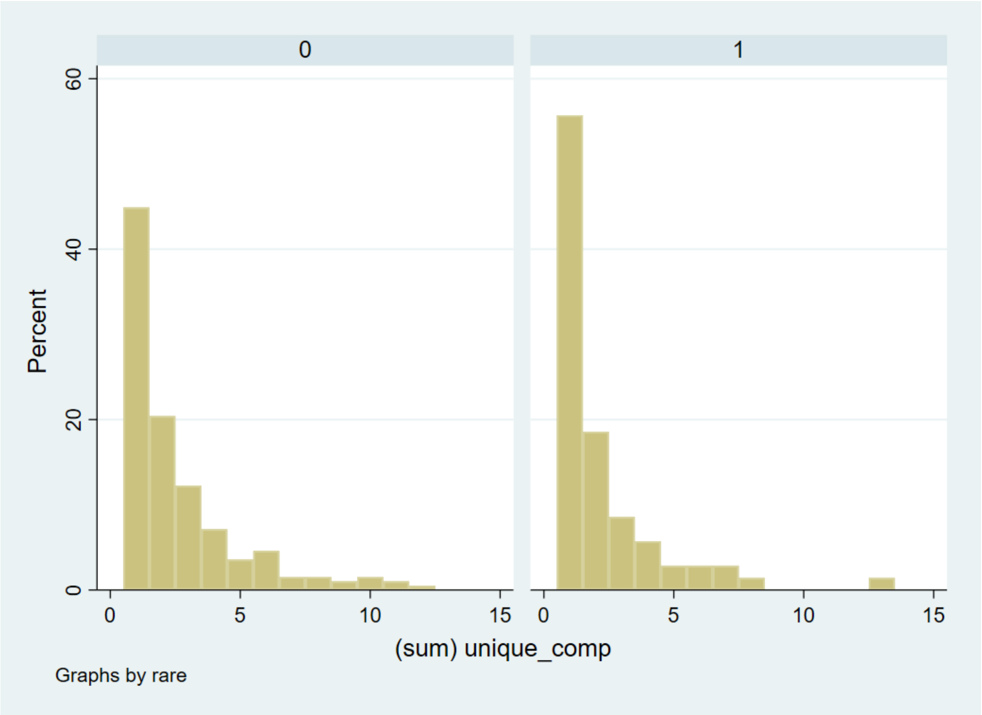

Figure 3. Histogram of share of overall industry funding to patient organisations coming from each contributing company in 2020, broken down by rarity of disease

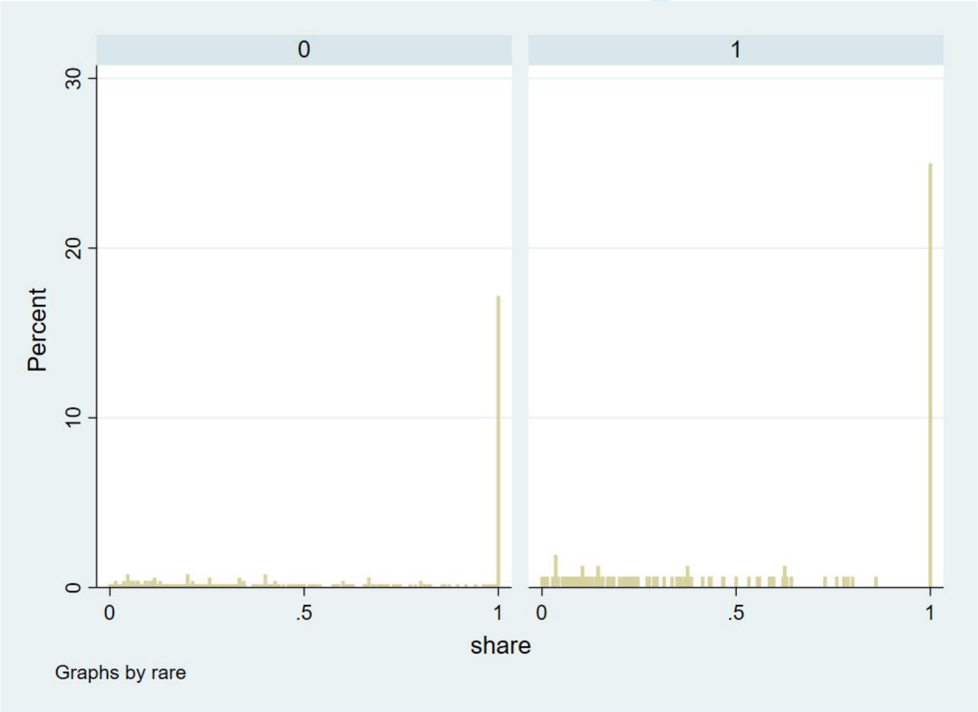

For peer review only - http://bmjopen.bmj.com/site/about/guidelines.xhtml

Figure 4. Histogram of share of industry funding of each organisation comprised by the single highest payment in 2020, broken down by rarity of disease

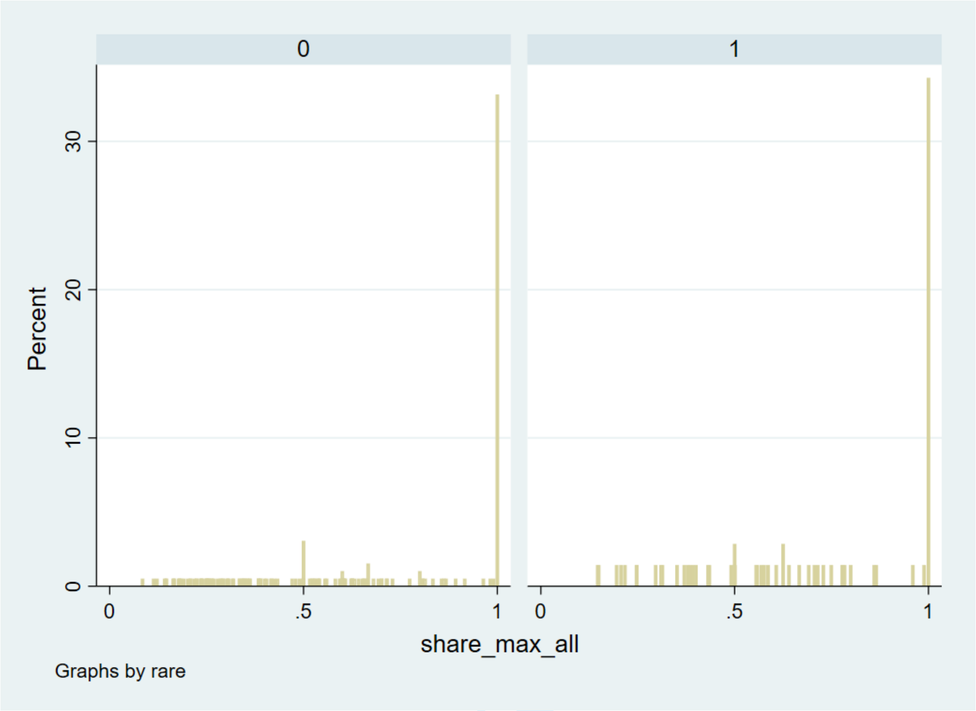

For peer review only

Sub-group analyses

Excluded patient organisations

66 payments made 28 to patient organisaitons were excluded from the analysis as they did not match EFPIA's definition of *"not-for-profit organisations, mainly composed of patients and/or caregivers, that represent and/or support the needs of patients and/or caregivers"*.

Figure 5 illustrates the reasons for patient organisations exclusion. Most of the excluded patient organisations were for profit organisations (47%; n=31), followed by not UK-based (42%; n=28) and organisations for which no information could be found online (11%; n=7).

Non-UK patient organisations mostly comprised international alliances of patient organisations, European or Irish organisations. We classified organisations as for-profit if they appeared in the UK government repository of companies[1] as *private limited companies.* Care homes, consultancies and rehabilitation clinics were the most prominent in this category.

Overall, payments to excluded patient organisations amounted to £869,677, about 4% of the included payments (Figure 6).

Figure 5. Excluded patient organisations by reason of exclusion

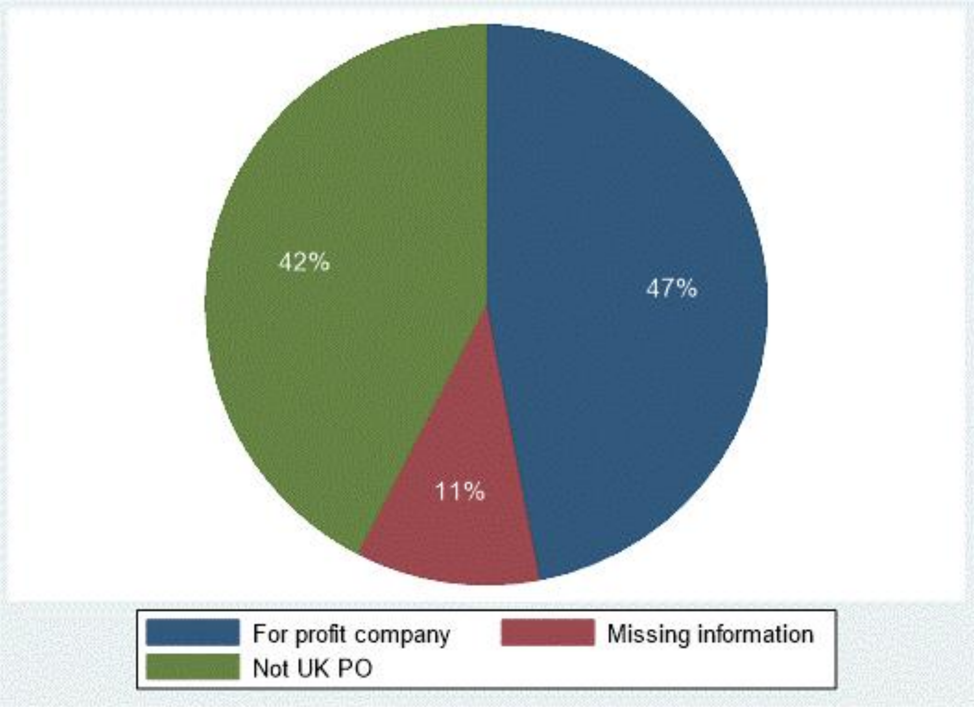

[1] https://find-and-update.company-information.service.gov.uk/

1 **Figure 6. Payments to included and excluded patient organisations**

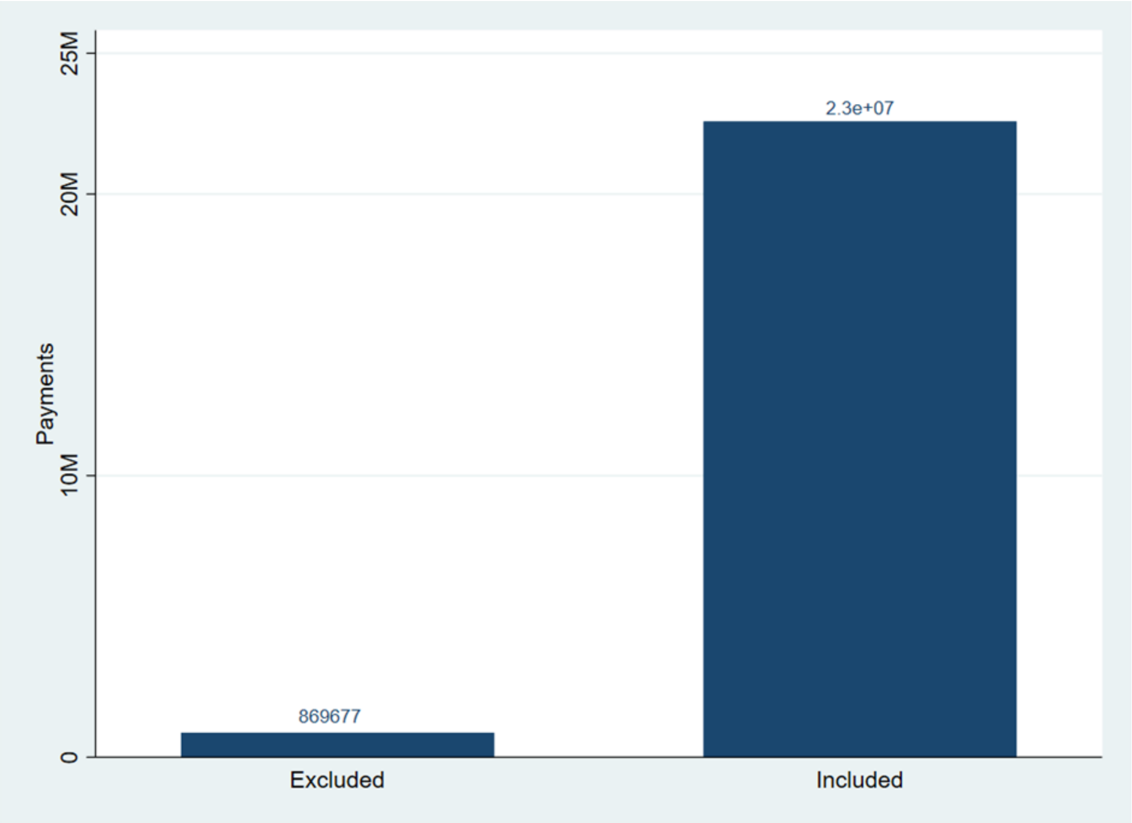

2

3

For peer review only

References

1. PMCPA. ABPI Code of Practice 2021 [Available from: https://www.pmcpa.org.uk/the-code/2021-interactive-abpi-code-of-practice/.
2. Rickard E, Carmel E, Ozieranski P. Comparing pharmaceutical company payments in the four UK countries: a cross-sectional and social network analysis. *BMJ Open* 2023;13(3):e061591. doi: 10.1136/bmjopen-2022-061591
3. Ozieranski P, Rickard E, Mulinari, Shai. Exposing drug industry funding of UK patient organisations. *BMJ* 2019;365:l1806. doi: 10.1136/bmj.l1806
4. HMRC. HMRC yearly average and spot rates: HM Revenue and Customs; [Available from: https://www.gov.uk/government/publications/exchange-rates-for-customs-and-vat-yearly.
5. HMRC. HMRC yearly average and spot rates: HM Revenue and Customs; 2022 [Available from: https://www.gov.uk/government/publications/exchange-rates-for-customs-and-vat-yearly.
6. Orphanet. The portal for rare diseases and orphan drugs 2022 [Available from: https://www.orpha.net/consor/cgi-bin/Disease_Search_Simple.php?lng=EN.
7. NICE. Policy on declaring and managing interests for NICE advisory committees, 2018.
8. WHO. ICD-11 for Mortality and Morbidity Statistics 2022 [Available from: https://icd.who.int/browse11/l-m/en#/http://id.who.int/icd/entity/465177735?view=G0.

For peer review only

CHEERS 2022 Checklist

Topic	No.	Item	Location where item is reported
**Title**			
	1	Identify the study as an economic evaluation and specify the interventions being compared.	p. 1, lines 1-3
**Abstract**			
	2	Provide a structured summary that highlights context, key methods, results, and alternative analyses.	p. 2, lines 4-33
**Introduction**			
**Background and objectives**	3	Give the context for the study, the study question, and its practical relevance for decision making in policy or practice.	p. 4, 5, 6 (all lines)
**Methods**			
**Health economic analysis plan**	4	Indicate whether a health economic analysis plan was developed and where available.	N/A
**Study population**	5	Describe characteristics of the study population (such as age range, demographics, socioeconomic, or clinical characteristics).	p. 7, lines 3-4
**Setting and location**	6	Provide relevant contextual information that may influence findings.	p. 7, line 4
**Comparators**	7	Describe the interventions or strategies being compared and why chosen.	N/A
**Perspective**	8	State the perspective(s) adopted by the study and why chosen.	p. 7, line 4
**Time horizon**	9	State the time horizon for the study and why appropriate.	p. 7, line 4
**Discount rate**	10	Report the discount rate(s) and reason chosen.	N/A
**Selection of outcomes**	11	Describe what outcomes were used as the measure(s) of benefit(s) and harm(s).	p. 7, 8, 9 (all lines)
**Measurement of outcomes**	12	Describe how outcomes used to capture benefit(s) and harm(s) were measured.	p. 7, 8, 9 (all lines)

For peer review only - http://bmjopen.bmj.com/site/about/guidelines.xhtml

Topic	No.	Item	Location where item is reported
**Valuation of outcomes**	13	Describe the population and methods used to measure and value outcomes.	p. 9, lines 12-15
**Measurement and valuation of resources and costs**	14	Describe how costs were valued.	N/A
**Currency, price date, and conversion**	15	Report the dates of the estimated resource quantities and unit costs, plus the currency and year of conversion.	p. 7, lines 30-34
**Rationale and description of model**	16	If modelling is used, describe in detail and why used. Report if the model is publicly available and where it can be accessed.	p. 8, lines 17-28
**Analytics and assumptions**	17	Describe any methods for analysing or statistically transforming data, any extrapolation methods, and approaches for validating any model used.	p. 9, lines 27-31
**Characterising heterogeneity**	18	Describe any methods used for estimating how the results of the study vary for subgroups.	N/A
**Characterising distributional effects**	19	Describe how impacts are distributed across different individuals or adjustments made to reflect priority populations.	N/A
**Characterising uncertainty**	20	Describe methods to characterise any sources of uncertainty in the analysis.	N/A
**Approach to engagement with patients and others affected by the study**	21	Describe any approaches to engage patients or service recipients, the general public, communities, or stakeholders (such as clinicians or payers) in the design of the study.	p. 9, lines 32-35
**Results**			
**Study parameters**	22	Report all analytic inputs (such as values, ranges, references) including uncertainty or distributional assumptions.	N/A
**Summary of main results**	23	Report the mean values for the main categories of costs and outcomes of interest and summarise them in the most appropriate overall measure.	p. 10, 11, 12, 13, 14 (all lines)
**Effect of uncertainty**	24	Describe how uncertainty about analytic judgments, inputs, or projections affect findings. Report the effect of choice of discount rate and time horizon, if applicable.	N/A

For peer review only - http://bmjopen.bmj.com/site/about/guidelines.xhtml

Topic	No.	Item	Location where item is reported
**Effect of engagement with patients and others affected by the study**	25	Report on any difference patient/service recipient, general public, community, or stakeholder involvement made to the approach or findings of the study	p. 9, lines 32-35
**Discussion**			
**Study findings, limitations, generalisability, and current knowledge**	26	Report key findings, limitations, ethical or equity considerations not captured, and how these could affect patients, policy, or practice.	p. 15-17 (all lines)
**Other relevant information**			
**Source of funding**	27	Describe how the study was funded and any role of the funder in the identification, design, conduct, and reporting of the analysis	p. 18, lines 11-15
**Conflicts of interest**	28	Report authors conflicts of interest according to journal or International Committee of Medical Journal Editors requirements.	p. 18, lines 16-20

From: Husereau D, Drummond M, Augustovski F, et al. Consolidated Health Economic Evaluation Reporting Standards 2022 (CHEERS 2022) Explanation and Elaboration: A Report of the ISPOR CHEERS II Good Practices Task Force. Value Health 2022;25. doi:10.1016/j.jval.2021.10.008

For peer review only - http://bmjopen.bmj.com/site/about/guidelines.xhtml
